# Head-to-head comparison of 6 plasma biomarkers in early multiple system atrophy

**DOI:** 10.1038/s41531-023-00481-5

**Published:** 2023-03-15

**Authors:** Yu Guo, Xue-Ning Shen, Shu-Yi Huang, Shu-Fen Chen, Hui-Fu Wang, Wei Zhang, Ya-Ru Zhang, Wei Cheng, Mei Cui, Qiang Dong, Jin-Tai Yu

**Affiliations:** 1 Department of Neurology and Institute of Neurology, Huashan Hospital, State Key Laboratory of Medical Neurobiology and MOE Frontiers Center for Brain Science, Shanghai Medical College, Fudan University, National Center for Neurological Disorders, Shanghai, China; 2 The Institute of Science and Technology for Brain-inspired Intelligence, Fudan University, Shanghai, China; 3 Fudan ISTBI—ZJNU Algorithm Centre for Brain-inspired Intelligence, Zhejiang Normal University, Zhejiang, China

**Keywords:** Movement disorders, Diagnostic markers

## Abstract

There is a dire need for reliable biomarkers to solidify an early and accurate diagnosis of multiple system atrophy (MSA). We sought to compare the ability of emerging plasma markers in distinguishing MSA from its mimics and healthy controls in early disease stages, and to evaluate their performance in detecting disease severity and brain atrophy. Plasma neurofilament light (NfL), glial fibrillary acidic protein (GFAP), phosphorylated tau181, amyloid-β (Aβ)42, and Aβ40 were measured using ultrasensitive Simoa in early-stage patients with MSA (*n* = 73), spinocerebellar ataxia (SCA, *n* = 29), Parkinson’s disease (PD, *n* = 28), and healthy controls (*n* = 100). We observed that elevated NfL outperformed other biomarkers in distinguishing MSA and its subtypes (AUC = 0.9) versus controls. Intriguingly, when separating MSA from its mimics, increased GFAP (AUC = 0.717) in MSA-C and decreased Aβ40 (AUC = 0.807) in MSA-P best discriminated from SCA and PD respectively. Plasma levels were comparable between MSA-C and MSA-P and the differentiation by plasma index alone was poor. Combining plasma markers noticeably improved the discriminatory efficacy. Of note, among MSA patients, higher GFAP and NfL were correlated with the atrophy of brain regions vulnerable to MSA (e.g., cerebellum, pons, or putamen). They could also aggravate the severity of MSA, and this association was partially mediated by cerebral volumes. In contrast, no obvious associations of phosphorylated tau and Aβ with disease severity were observed. Collectively, plasma biomarkers, especially in combination, are useful to facilitate the discriminatory work-up of MSA at early stages. Moreover, NfL and GFAP may be promising biomarkers to monitor the disease severity of MSA.

## Introduction

Multiple system atrophy (MSA) is an orphan, adult-onset, fatal neurodegenerative disorder characterized by a variable phenotypic combination of predominant parkinsonian (MSA-P) or cerebellar (MSA-C) symptoms, autonomic failure, and pyramidal signs. Because of its protean clinical presentations, MSA may be misdiagnosed^1,2^. The parkinsonian subtype often presents with parkinsonism and additional features of dysautonomia, which may be indistinguishable from Parkinson’s disease (PD). The manifestation of late-onset cerebellar ataxia with autonomic involvement can masquerade as symptoms induced by spinocerebellar ataxia (SCA).

MSA is rapidly progressing and curative treatment is not available; the mean survival time is approximately 6 to 10 years, with few patients surviving more than 15 years^2^. Although in our experience MSA can generally be distinguished from PD and SCA by standardized neuroimaging, autonomic or genetic tests, it can be difficult to distinguish during early disease stages or when the manifestation is atypical^1^. Recent clinicopathological studies also found suboptimal accuracy (62–79%) of MSA diagnosis^3,4^. All of these reports emphasize the dire need for reliable biomarkers to solidify an early and accurate diagnosis of MSA^5,6^.

The increased neurofilament light chain (NfL) levels have been recently proposed as a supportive biomarker of MSA given the good discriminatory ability for parkinsonian disorders^6^. Nonetheless, evidence providing the value of NfL in early MSA stages is insufficient. In addition, due to limited availability and lack of validation, supporting biomarkers are still far from meeting the diagnostic requirements of MSA^6^.

Previous biomarker research usually adopted MSA as a part of parkinsonism and observed the performance of several fluid markers such as glial fibrillary acidic protein (GFAP), phosphorylated tau (p-tau), and amyloid-β (Aβ)^7,8^. However, the differentiation in MSA diagnostic categories by treating MSA as an independent entity has been neglected^6,9,10^, especially with less research on distinguishing MSA-C from SCA. The results were not always reproducible and the patient groups were not well-characterized with clinical or imaging features, which limited the application of the emerging or consolidating markers in clinical practice. Moreover, head-to-head studies of the aforementioned plasma biomarkers across MSA and MSA look-alike disorders are lacking.

Herein, we aimed to systematically compare the plasma levels of NfL, GFAP, p-tau181, Aβ40, Aβ42, and Aβ42/40 among patients with early stages of MSA, PD, and SCA and healthy controls (HCs). We then determined the abilities of these markers, individually or in combination, to accurately distinguish across MSA categories. Next, we examined the relations of the plasma markers with comprehensive clinical and neuroimaging features in patients with MSA. Last, we explored whether the influences of plasma indicators on clinical phenotypes were mediated by imaging alterations. We predict that plasma biomarkers may vary widely from disease to disease and some of them may have potential values of clinical application.

## Results

### Sample characteristics

At study entry, we analyzed 73 MSA cases (age, 58.62 ± 7.85 years; male, 56.16%; 58 MSA-C and 15 MSA-P), 29 SCA cases, and 28 PD cases in early disease stages, and 100 HCs. PD patients tended to be older and SCA patients tended to be younger, which is in keeping with the corresponding disease features. There were no significant differences among diagnostic groups in sex and disease duration (Table 1). One measurement of NfL in an MSA participant was considered an outlier and removed from further analyses as it exceeded 5 standard deviations of the mean (NfL = 397.26 pg/mL). We found that age was strongly correlated with NfL, p-tau181, GFAP, and Aβ42/40 concentrations. Sex could affect GFAP levels, and education could affect GFAP and Aβ42/40 levels. However, disease duration was not associated with any of the plasma biomarkers tested (Supplementary Table 1).

**Table 1:** Baseline characteristics in each diagnostic group.

Characteristic	HC	MSA	MSA-C	MSA-P	SCA	PD	*P* value
	*N* = 100	*N* = 73	*N* = 58	*N* = 15	*N* = 29	*N* = 28	
Age, years	59.50 (8.91)	58.62 (7.85)	58.81 (8.06)	57.87 (7.20)	46.55 (11.91)	64.04 (9.33)	<0.001
Male, *n* (%)	44 (44.00%)	41 (56.16%)	33 (56.90%)	8 (53.33%)	16 (55.17%)	16 (57.14%)	0.339
Disease duration, months	–	19.48 (8.69)	20.64 (8.91)	15.00 (6.15)	21.66 (10.97)	19.79 (10.92)	0.729
Education, years	9.69 (4.22)	8.26 (4.24)	8.61 (4.19)	6.93 (4.30)	10.42 (4.22)	7.79 (3.80)	0.042
Plasma NfL, pg/mL	13.74 (8.53)	40.92 (44.89)	43.24 (49.62)	31.95 (15.12)	33.64 (31.31)	27.26 (19.79)	<0.001
Plasma GFAP, pg/mL	69.20 (35.69)	92.44 (58.34)	98.40 (62.50)	69.42 (29.79)	65.74 (42.96)	115.34 (54.70)	<0.001
Plasma *p*-tau181, pg/mL	1.99 (0.84)	1.42 (0.65)	1.38 (0.64)	1.58 (0.68)	1.43 (0.58)	1.75 (0.91)	<0.001
Plasma Aβ40, pg/mL	83.02 (25.78)	64.87 (23.18)	66.93 (24.84)	56.90 (12.89)	71.33 (21.34)	85.71 (29.16)	0.006
Plasma Aβ42, pg/mL	4.96 (1.63)	4.46 (1.37)	4.60 (1.38)	3.93 (1.23)	4.82 (1.37)	5.66 (1.85)	0.043
Plasma Aβ42/40	0.06 (0.02)	0.07 (0.01)	0.07 (0.01)	0.07 (0.01)	0.07 (0.01)	0.07 (0.01)	0.003

*Αβ* amyloid-β, *GFAP* glial fibrillary acidic protein, *HC* healthy control, *MSA* multiple system atrophy, *MSA-C* multiple system atrophy-cerebellar type, *MSA-P* multiple system atrophy-parkinsonian type, *NfL* neurofilament light, *PD* Parkinson’s disease; *p-tau181* phosphorylated tau at threonine 181, *SCA* spinocerebellar ataxia.

### Plasma levels across divergent diagnostic groups

**Fig. 1:**
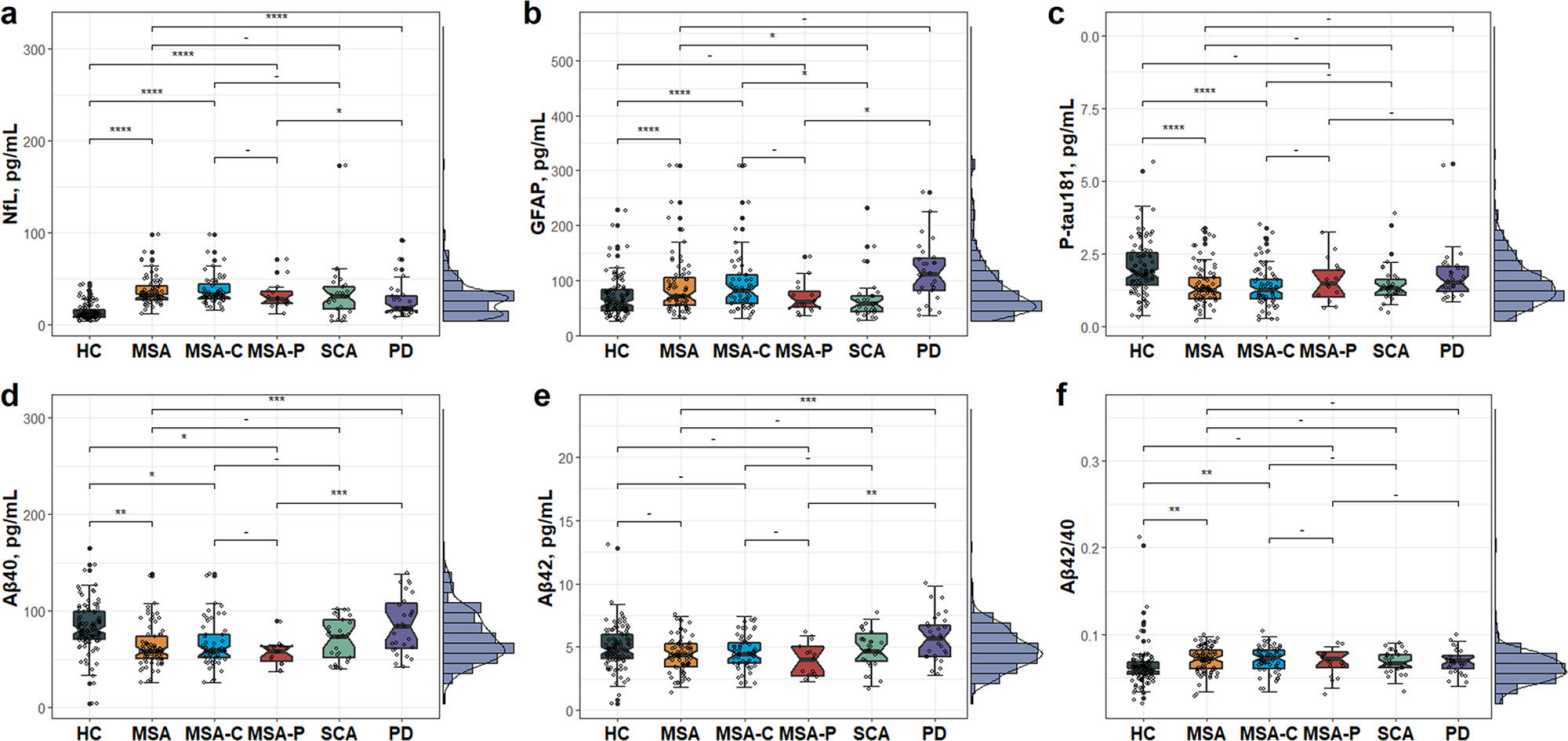
Distributions of the plasma levels across diagnostic groups. Plasma levels of NfL (**a**), GFAP (**b**), p-tau181 (**c**), Αβ40 (**d**), Aβ42 (**e**), and Αβ42/40 (**f**) per diagnostic group were compared using analysis of covariance after controlling for age and sex. In boxplots, the center line indicates the median, and the lower and upper bounds of the box indicate the 25% quartile (Q1) and 75% quartile (Q3), respectively. The lower whisker indicates Q1-1.5*interquartile range (IQR) and the upper whisker indicates Q3 + 1.5*IQR. Significance: ****p < 0.0001, ***p < 0.001, **p < 0.01, *p < 0.05, -*p* ≥ 0.05. Αβ amyloid-β, GFAP glial fibrillary acidic protein, HC healthy control, MSA multiple system atrophy, MSA-C multiple system atrophy-cerebellar type, MSA-P multiple system atrophy-parkinsonian type, NfL neurofilament light, NS non-significant, PD Parkinson’s disease, p-tau181 phosphorylated tau at threonine 181, SCA spinocerebellar ataxia.

Plasma levels were differentially distributed among the diagnostic groups (Fig. 1), while no evident differences were found between MSA-C and MSA-P patients.

### NfL

NfL levels were markedly elevated among MSA and its subtypes when compared with HC and PD groups (all *p* < 0.05). However, the obvious difference vanished between MSA-C (or MSA) and SCA groups.

### GFAP

Plasma GFAP levels were increased in MSA and MSA-C groups (all *p* < 0.0001) but not in the MSA-P group when compared with controls. The concentrations of GFAP in SCA patients were lower than those observed in patients with MSA-C or MSA. Besides, MSA-P patients displayed lower GFAP concentrations relative to PD patients.

### P-tau181

Both MSA and MSA-C groups had decreased plasma p-tau181 levels relative to those controls, while the MSA-P and HC groups did not differ. The levels of p-tau181 were similar among MSA-C (or MSA) versus SCA, MSA-P (or MSA) versus PD, and MSA-C versus MSA-P groups.

### Aβ40

The levels of Aβ40 in MSA and its subtypes were far below those observed in HCs and PD. In contrast, MSA-C (or MSA) and SCA groups displayed comparable Aβ40 concentrations.

### Aβ42 and Aβ42/40

Concentrations of Aβ42 and Aβ42/40 across divergent diagnostic groups were similar, with the exception that MSA (or MSA-P) patients showed decreased Aβ42 levels than PD patients and MSA (or MSA-C) patients had elevated Aβ42/40 levels than those controls.

### ROC analyses

#### MSA/MSA-C/MSA-P versus HC

**Fig. 2:**
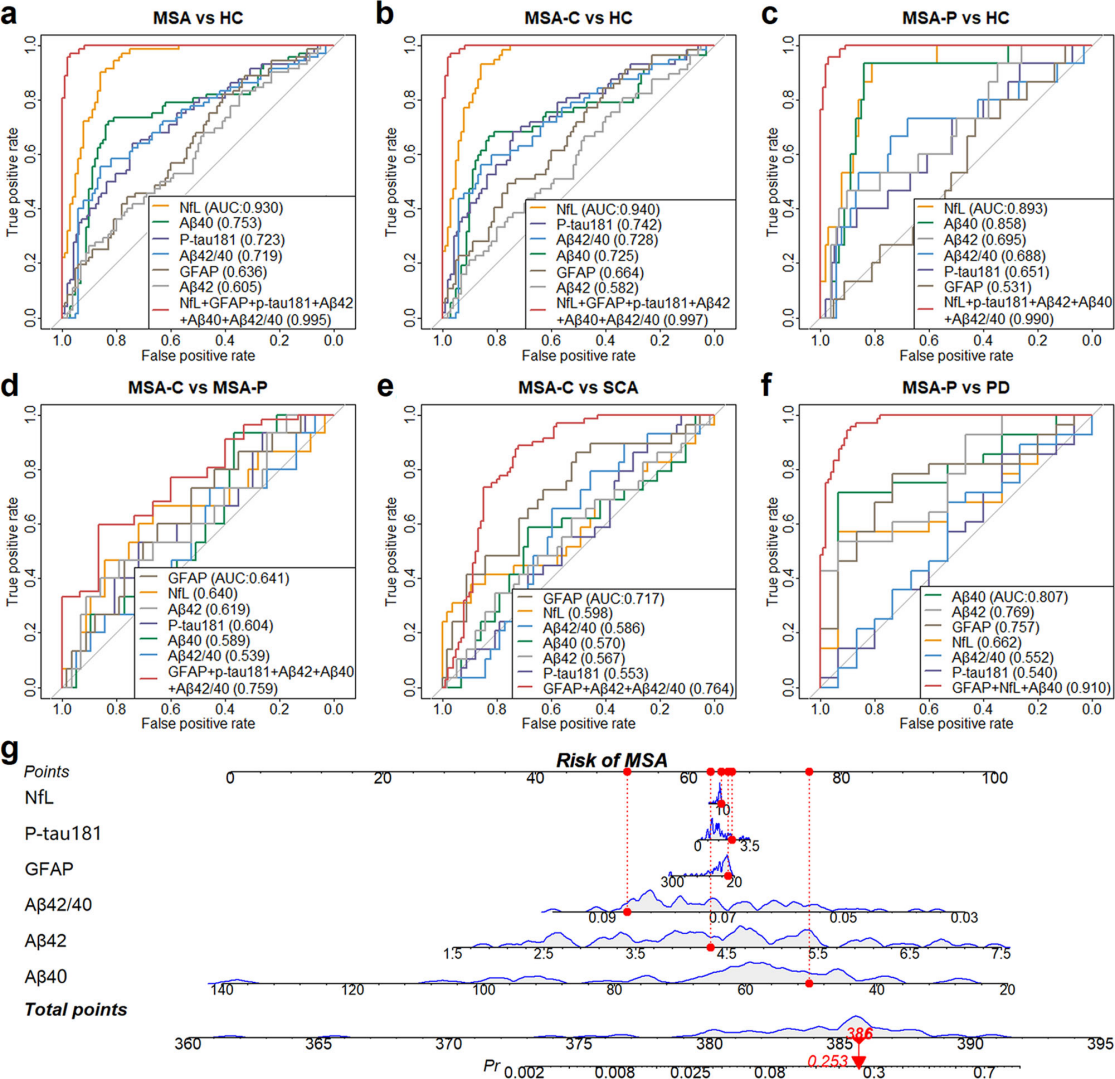
Performance of plasma markers in discrimination diagnosis. The receiving operating characteristic curves were delineated for differentiating MSA, MSA-C, and MSA-P from HC (**a**–**c**), MSA-C from MSA-P (**d**), MSA-C from SCA (**e**), and MSA-P from PD (**f**). **g** The nomogram for predicting the risk of being diagnosed with MSA. The concentration ranges of each plasma biomarker and the corresponding scores are shown. The example here shows a predicted risk of 25.3%. Αβ amyloid-β, AUC area under the curve, GFAP glial fibrillary acidic protein, HC healthy control, MSA multiple system atrophy, MSA-C multiple system atrophy-cerebellar type, MSA-P multiple system atrophy-parkinsonian type, NfL neurofilament light, PD Parkinson’s disease, p-tau181 phosphorylated tau at threonine 181, SCA spinocerebellar ataxia.

ROC analyses were performed to evaluate the ability of plasma markers, alone or in combination, in differential diagnoses (Fig. 2a–f and Supplementary Table 2). When distinguishing MSA versus controls, NfL had high accuracy (AUC = 0.930) with good sensitivity (90%) and specificity (86%). Aβ40 (AUC = 0.753), p-tau181 (AUC = 0.723), and Aβ42/40 (AUC = 0.719) showed moderate accuracy, while GFAP and Aβ42 displayed low accuracy. Similar patterns were observed in the differentiation of MSA-C or MSA-P versus HC, except that plasma p-tau181 showed low accuracy in distinguishing MSA-P from controls. Applying the panel of all six plasma biomarkers yielded nearly perfect accuracy to discriminate MSA (AUC = 0.995) or MSA-C (AUC = 0.997) from HC. When distinguishing between MSA-P and HC, the discriminatory power reached highest for NfL combined with p-tau181, Aβ42, Aβ40, and Aβ42/40 (AUC = 0.990).

#### MSA-C versus MSA-P

When we looked into MSA-C and MSA-P subtypes, we found each single plasma indicator performed poorly, with AUCs reaching about 0.6 or lower. The highest accuracy for separating MSA subtypes was found in the combination of GFAP, p-tau181, Aβ42, Aβ40, and Aβ42/40, where the AUC reached 0.759 (sensitivity = 0.596, specificity = 0.867).

#### MSA-C versus SCA

The AUC of plasma GFAP for separation of MSA-C versus SCA was 0.717 (sensitivity = 0.51, specificity = 0.86). ROC analyses for NfL, Aβ42/40, Aβ40, Aβ42, and p-tau181 only resulted in an AUC of 0.598, 0.586, 0.570, 0.567, and 0.553, respectively. The diagnostic accuracy was enhanced for GFAP in combination with Aβ42 and Aβ42/40 (AUC = 0.764).

#### MSA-P versus PD

MSA-P could be differentiated from PD with an AUC of 0.807 (sensitivity = 0.714, specificity = 0.933) for Aβ40. The discriminatory power weakened for Aβ42, GFAP, NfL, Aβ42/40 and p-tau181 (AUC: 0.769, 0.757, 0.662, 0.552 and 0.540, respectively). The model consisting of NfL, GFAP, and Aβ40 revealed a high AUC of 0.910 (sensitivity = 0.800, specificity = 0.929).

#### Sensitivity analyses for differential diagnosis

To investigate the influence of confounders on the differential diagnosis of diseases, ROC analyses were repeated after adjusting for age and sex. The derived results were essentially unchanged (Supplementary Table 3).

#### Risk of MSA

From the nomogram, we could clearly see the risk of being diagnosed with MSA based on different levels of plasma biomarkers (Fig. 2g).

### Plasma markers correlated with clinical scales

**Fig. 3:**
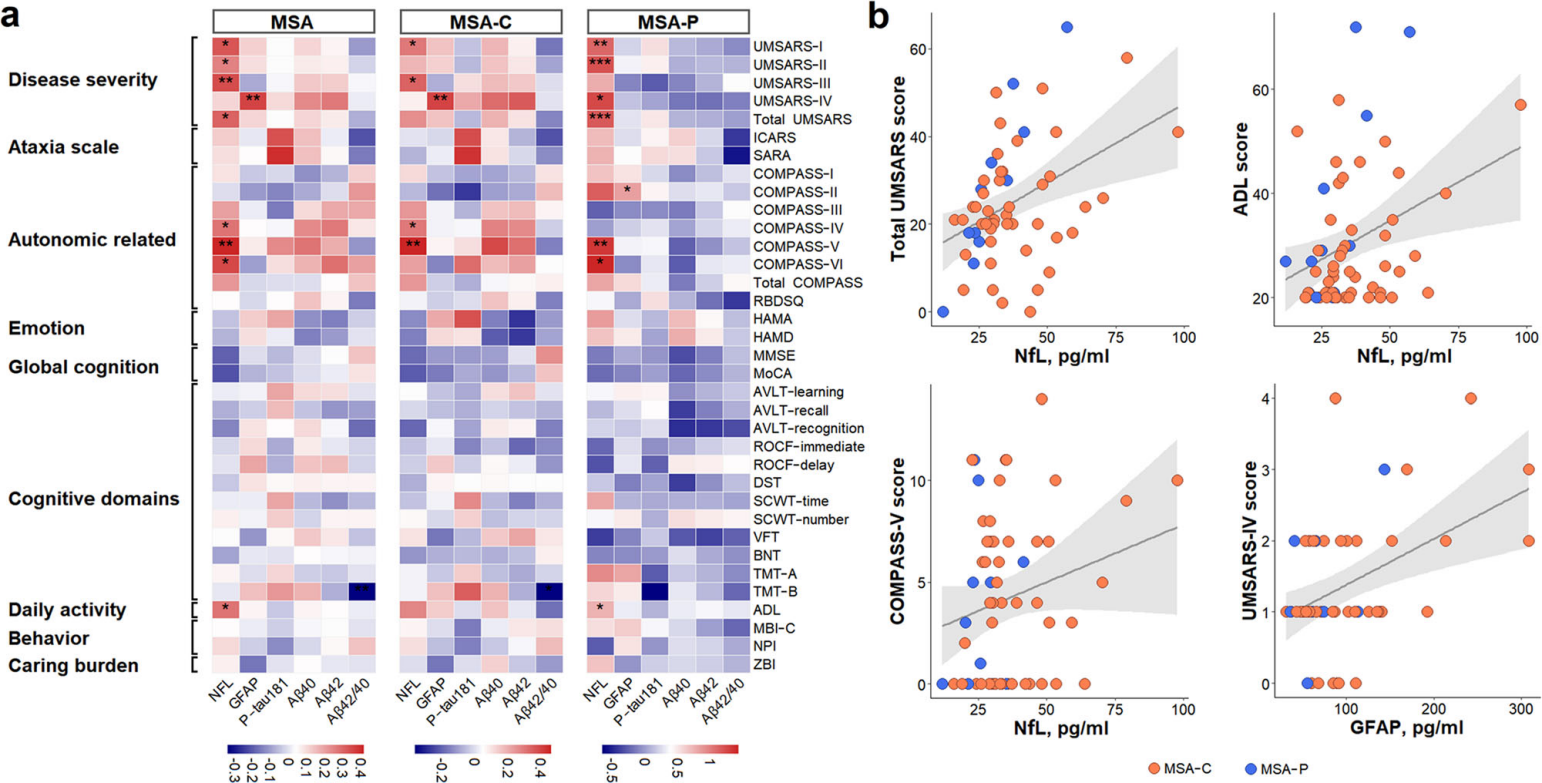
Plasma measures in relation to clinical assessment scales in MSA and its subtypes. **a** The left heat map showed correlations of clinical scales with plasma biomarkers, with colors representing the correlation coefficients (β) of multiple linear regressions. The color bar represents the range of β values. Models were adjusted for age and sex. Significance: ***p < 0.001, **p < 0.01, *p < 0.05, -*p* ≥ 0.05. **b** The right scatter plots displayed significantly positive associations between NfL or GFAP and clinical scores. Αβ amyloid-β, ADL Activity of Daily Living Scale, AVLT Auditory Verbal Learning Test, BNT Boston Naming Test, COMPASS Composite Autonomic Symptom Score, DST Digit Span Test, GFAP glial fibrillary acidic protein, HAMA Hamilton Anxiety Scale, HAMD Hamilton Depression Scale, ICARS International Cooperative Ataxia Rating Scale, MBI-C Mild Behavioral Impairment Checklist, MMSE Mini-Mental State Examination, MoCA Montreal Cognitive Assessment, MSA multiple system atrophy, MSA-C multiple system atrophy-cerebellar type, MSA-P multiple system atrophy-parkinsonian type, NfL neurofilament light, NPI Neuropsychiatric Inventory, p-tau phosphorylated tau, RBDSQ Rapid eye movement sleep behavior disorder screening questionnaire, ROCF Rey-Osterreich Complex Figure, SARA Scale for the Assessment and Rating of Ataxia, SCWT Stroops Color Word Test, TMT Trails Making Test, UMSARS Unified Multiple System Atrophy Rating Scale, VFT Verbal Fluency Test, ZBI Zarit Caregiver Burden Interview.

Next, we comprehensively evaluated the relations of plasma indicators with clinical assessment scales in patients with MSA (Fig. 3). Plasma NfL levels were positively correlated with the disease severity as reflected by UMSARS-I (β = 0.329, *p* = 0.012), UMSARS-II (β = 0.250, *p* = 0.048), UMSARS-III (β = 0.341, *p* = 0.009), and total UMSARS (β = 0.305, *p* = 0.017). Higher NfL levels could worsen autonomic function (COMPASS-IV: β = 0.277, *p* = 0.043; COMPASS-V: β = 0.413, *p* = 0.002; COMPASS-VI: β = 0.352, *p* = 0.045) and impair the ability to perform daily activities (ADL: β = 0.272, *p* = 0.036). Likewise, elevated GFAP levels could exacerbate global disability (UMSARS-IV: β = 0.356, *p* = 0.008). These results were robust in MSA-C and MSA-P subtypes, albeit the significance was weakened after multiple FDR corrections (Supplementary Table 4). Moreover, we did not find significant associations of plasma p-tau181, Aβ40, Aβ42, and Aβ42/40 with multidimensional scales in MSA patients, except the inverse association between Aβ42/40 and cognitive function (as measured by Trails Making Test).

### Associations of plasma measures with neuroimaging indices

**Fig. 4:**
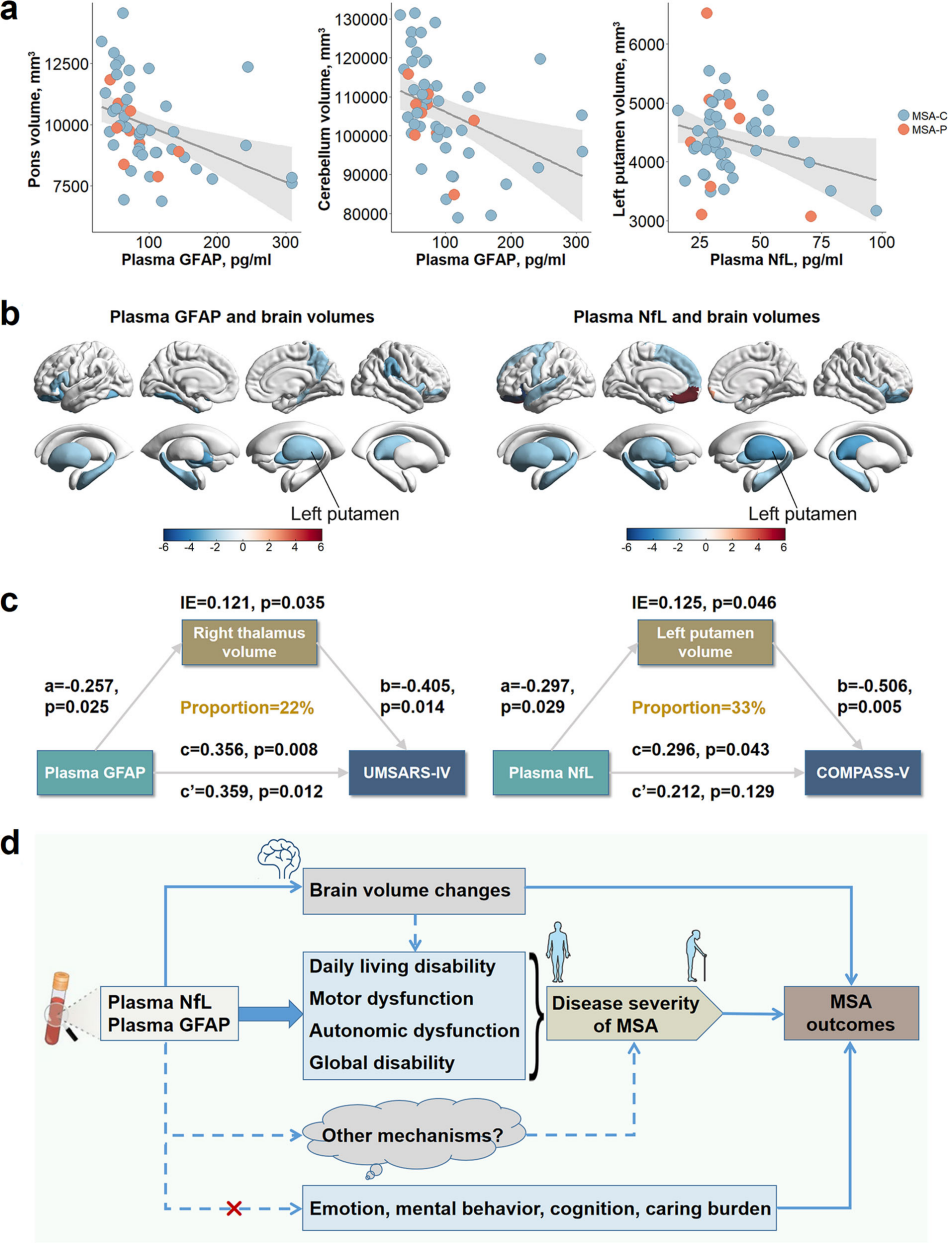
Associations of plasma measures with imaging indices in MSA patients. **a** Associations of plasma GFAP or NfL with MSA-specific brain regions were shown in scatter plots. **b** Relationships of GFAP and NfL levels with cortical and subcortical volumes were delineated, with colors reflecting *t* values in multiple linear regressions. The color bar represents the range of *t* values. **c** Results of mediation analyses. The relationship between plasma markers and clinical scores could be partially mediated by brain volumes. **d** A schematic graph depicting associations among plasma markers, imaging indices, and clinical scales. Changes in brain volumes could partially modulate the associations of plasma NfL and GFAP with the disease severity of MSA. Future studies are warranted to explore whether there are more mediators via which plasma markers contributed to disease severity. Neither NfL nor GFAP could affect emotion, mental behavior, cognition, or caring burden among MSA patients. GFAP glial fibrillary acidic protein, IE indirect effect, MSA multiple system atrophy, NfL neurofilament light.

Brainstem volumes were extracted and further segmented into pons, midbrain, and medulla in the present study. Plasma GFAP levels were significantly correlated with volumetric atrophy in the pons (β = −0.375, *p* = 0.002) and the whole brainstem (β = −0.301, *p* = 0.010) (Fig. 4a), but not with midbrain and medulla volumes. The correlations remained significant after FDR corrections (Supplementary Table 5). Besides, plasma GFAP was associated with cerebellum atrophy, including bilateral cerebellar cortex (left: β = −0.253, *p* = 0.039; right: β = −0.269, *p* = 0.032) and white matter (left: β = −0.292, *p* = 0.025; right: β = −0.387, *p* = 0.002). In contrast, these associations for NfL were substantially attenuated.

Regarding subcortical areas, NfL levels were correlated with volumetric atrophy in the left putamen (β = −0.297, *p* = 0.029), bilateral pallidum, bilateral thalamus, and bilateral hippocampus (Fig. 4b). Likewise, plasma GFAP was associated with atrophy of the left putamen (β = −0.275, *p* = 0.040), bilateral pallidum, right thalamus, and left hippocampus. These associations were slightly weakened after multiple corrections (Supplementary Table 5). As for cortical areas, NfL and GFAP were correlated with atrophy of the left lateral orbitofrontal cortex, right pars orbitalis, and right insula, but many correlations did not survive FDR correction.

No obvious differences were found for plasma NfL (*p* = 0.218) and GFAP (*p* = 0.867) between DAT-PET positive and negative groups (Supplementary Fig. 1), and their associations with quantitative DAT-PET uptake were not significant in all measured cerebral areas (Supplementary Table 6). Besides, we did not find significant associations between glucose metabolism and NfL or GFAP in MSA-susceptible areas. The limited population size available for PET imaging may be partly responsible for these negative associations.

### Validation and complementary analyses

The above results remained essentially unchanged when we expanded the sample size irrespective of the duration of symptoms (Supplementary Table 7–11; Supplementary Fig. 2). The discriminative performance of the plasma biomarkers was also validated in an independent advanced disease cohort, albeit with a somewhat small sample size (Supplementary Table 12–13; Supplementary Fig. 3).

Moreover, we performed ROC analyses for neuroimaging indices (Supplementary Table 14). We found that when distinguishing MSA-C from SCA, plasma GFAP was superior to neuroimaging indices, while other plasma markers performed similarly to neuroimaging indices. When distinguishing MSA-P from PD, plasma Aβ40 was superior to neuroimaging indices, while other plasma markers performed similarly to neuroimaging indices.

### Mediation analyses

Based on the above associations, we then observed that the association between GFAP and UMSARS-IV was partially mediated by right thalamus volume (mediation proportion = 22%), and the relationship between NfL and COMPASS-V could be partially modulated by left putamen volume (mediation proportion = 33%) (Fig. 4c).

## Discussion

Through head-to-head comparisons of plasma biomarkers at early disease stages, we confirmed that NfL was a good discriminator with markedly high values for MSA and its subtypes compared to controls. Besides, this study disclosed the clinical value of GFAP in distinguishing MSA-C from SCA. Both NfL and GFAP could aggravate the disease severity of MSA and contribute to the atrophy of MSA-susceptible areas. And the former association was partially mediated by brain volumes (Fig. 4d). When discriminating MSA-P versus PD, Aβ40 performed best, albeit the accuracy was modest. The separation of MSA subtypes by plasma index alone was poor, and the combination of variables noticeably improved the discriminatory efficacy.

Ascertaining the levels of plasma markers in early stages is imperative for the diagnostic workup of MSA. Our findings are in keeping with the notion that NfL was markedly elevated in MSA and its subtypes with superb discriminatory ability from controls^7,8,11–16^. The increased NfL levels in MSA relative to PD are supported by several publications, although the accuracy of NfL in distinguishing MSA-P from PD is lower than that of previous work for differentiating MSA and PD^11,12,17,18^. Instead, we found the ability of Aβ40 or Aβ42 in classifying a diagnosis of MSA-P versus PD was superior to that of NfL, albeit the sensitivity was relatively low. This is in stark contrast to former Aβ studies, which reported either negative results^7,8^ or similar but worse discriminative ability as NfL^16^. Many studies have looked at Aβ42 in MSA, yet the results have been conflicting, with some describing significantly reduced Aβ42 levels in MSA patients while others observing no obvious difference among MSA, PD, and control groups^8,16,19–25^. The lower concentrations of Aβ40 in MSA than in PD and HCs are in disagreement with original CSF and plasma studies^16,21,22^. Population characteristics (e.g., sample size, distinct disease duration, and motor severity) might be responsible for part of these differences. Another major variability likely comes from biomarker measurement approaches (e.g., whether ELISA or the more sensitive Simoa method was used) and matrix effects (CSF versus blood). Future studies are warranted to confirm the role of Aβ in MSA.

To date, there has been little research on the diagnostic utility of fluid biomarkers to separate MSA-C from SCA, as MSA has traditionally been studied as a part of parkinsonism^9,10,16,26–31^. The high proportion of MSA-C patients in this study, which may relate to the population ethnicity^32^, could well bridge the existing knowledge gaps. Although previous research suggested that NfL could differentiate MSA-C from sporadic adult ataxia^33^, we failed to detect the good performance of NfL in the discrimination of MSA-C versus SCA and the differences in NfL levels between the two groups were not obvious. The non-significant association between NfL and pons or cerebellum volume, while conflicting with the findings in SCA patients^34^, may indirectly support the above results. The insufficient number of patients with sporadic adult ataxia currently included in our cohort limited us to observe the performance of different plasma markers in differentiating these patients from those with MSA-C. This is a direction for our future research. Intriguingly, we identified that the single blood marker offering sufficient diagnostic accuracy was unavailable besides GFAP. Although its sensitivity was relatively low, combining markers improved the discriminatory efficacy. Our results also indicated that plasma GFAP could aggravate global disability in MSA patients, and this association could be partially mediated by thalamus atrophy. Besides, GFAP levels were significantly correlated with atrophy in brain regions (e.g., cerebellum, pons, brainstem) vulnerable to MSA. Former GFAP studies focused only on its poor performance in distinguishing MSA from PD or HCs^7,15^, which was consistent with our results, but ignored its ability to distinguish MSA-C from SCA. Our observations provide tentative hints that GFAP might be a promising peripheral biomarker for the determination of degenerative ataxia, which contributes to the body of evidence pointing towards the involvement of glial degeneration in MSA^2,10^. Pending replication in independent, diverse, and larger cohorts, plasma GFAP might serve as a candidate marker of disease severity in MSA. Given the rapid progression of MSA and its unclear pathogenesis, identifying promising biomarkers and unraveling complicated mechanisms will greatly assist in tailoring therapeutic interventions timely.

As indicated by the results, disease discrimination has its characteristic biomarker pattern, which may help clinicians in improving diagnostic certainty. For example, of the plasma markers we evaluated, NfL performed best at differentiating MSA from HCs, while GFAP performed best at differentiating MSA-C from SCA. Nevertheless, we have to acknowledge that the capability of plasma index alone in separating MSA from its mimics and MSA subtypes was insufficient. Utilizing the biomarker panel noticeably improved the discrimination of MSA-P from PD, but not for the discrimination between MSA-C and SCA or MSA-P. Whether there are more sensitive and specific biological markers needs to be further explored. Concerning p-tau181, original studies yielded contradictory results, with some reporting similar CSF levels in MSA, PD, and HCs, and others reporting decreased CSF p-tau levels in MSA^7,8^. In this study, we present preliminary evidence of plasma p-tau in the identification of MSA, showing reduced levels relative to HC but comparable levels to PD or SCA as well as moderate accuracy in distinguishing MSA from HC but poor accuracy in distinguishing MSA from its mimics. Collectively, our findings enabled a better differential diagnosis for MSA, which might be a desirable complement to the current biomarker panel.

Our findings add to the reliability of NfL in reflecting the disease severity of MSA^13,24,35,36^, which sheds light on its potential to detect therapeutic effects on axonal degeneration in clinical trials. Further, we revealed that NfL might exacerbate disease severity through more complex mechanisms, such as affecting brain atrophy, instead of direct effects. Whether there are more mediators is a direction for future research. Consistent with a recent study^13^, we did not find NfL levels linked to general cognition in patients with MSA. Besides NfL, the relationship between plasma metrics and multidimensional outcomes of MSA has rarely been investigated. Our study makes up for the vacuum of relevant knowledge, albeit most associations were not evident. The biomarkers studied may not be entirely specific for MSA, as similar abnormalities can be observed in other diseases. Despite this, we could not ignore the value of these markers, and more systematic studies in larger, well-defined clinical cohorts are needed to determine their clinical use.

Our study has several strengths. The focus on early disease stages helps ascertain which biomarkers have the greatest clinical utility. Simultaneous comparisons of multiple plasma biomarkers evaluated using the same method greatly minimize the bias due to methodological heterogeneity. The meticulous clinical and imaging phenotyping of subjects was another strength. A caveat, however, is that the disease diagnoses were not confirmed by postmortem neuropathology. Importantly, clinical diagnoses were made by at least two physicians specializing in movement disorders, and patients were followed over time with reassessments at each visit (if any). All of these should improve diagnostic accuracy. In addition, our longitudinal data are insufficient to understand whether and how plasma biomarkers evolve during divergent disease courses. Consequently, enriching the sample size of our cohort is a future research direction. Moreover, the sample size was relatively small. Larger and multicenter studies are needed to further validate and generalize our findings.

Collectively our findings identified some interesting diagnostic markers. Applying them in clinical practice, especially in combination, may aid in overcoming existing diagnostic obstacles and greatly facilitate the discriminatory work-up between early MSA and its mimics, particularly when atypical features are present and the clinical criteria are not met or only partially met. Furthermore, the release of NfL or GFAP into the peripheral blood may represent a significant opportunity for monitoring the disease severity of MSA in a non-invasive manner. These markers may serve as efficacy measures or surrogates of target engagement for future clinical trials.

## Methods

### Study population

Patients with early stages (within 3 years of motor onset) of MSA, PD, and SCA were enrolled between June 2019 and February 2022 from Huashan Hospital, Fudan University. The diagnosis of the diseases was made by movement disorder specialists following the internationally established consensus statement^6,37,38^, and patients were followed over time and reevaluated at each visit (if available). All MSA patients included in this study had their clinical diagnosis verified by at least one long-term follow-up visit. In order to differentiate from MSA-C and MSA-P patients, we randomly selected SCA and PD patients respectively within 3 years of motor onset who visited Huashan Hospital between June 2019 and February 2022, with a matching ratio of approximate 2:1 or 1:2. All SCA patients were genetically diagnosed based on concrete genetic reports and had evidence of progressive cerebellar ataxia, and/or other clinical symptoms. Subjects with central nervous system infections, head trauma, other neurodegenerative disorders (e.g., epilepsy, Alzheimer’s disease), other major neurological disorders, major psychological diseases, severe systemic diseases (e.g., cancer), and family history of genetic diseases other than SCA were excluded. Furthermore, we randomly enrolled 100 healthy subjects matched for age and sex with MSA patients who visited Qingdao Municipal Hospital between June 2019 and February 2022. Regional ethical committees of Qingdao Municipal Hospital and Huashan Hospital, Fudan University approved this study (approval number: KY2020-1161, KY2020-065). Written informed consent was obtained from all participants or authorized representatives. All research procedures adhered to the tenets of the Declaration of Helsinki.

### Clinical assessment

All MSA patients underwent thorough clinical and neurological examinations and were evaluated during face-to-face interviews by neurologists who were unaware of the study design and analytic procedures. Disease duration was uniformly defined as the time interval from the onset of motor symptoms to enrollment. Disease severity was rated with part-I (activities of daily living), part-II (motor examination), part-III (autonomic examination), and part-IV (global disability) of the Unified Multiple System Atrophy Rating Scale (UMSARS)^39^. Total UMSARS score is the sum of parts I and II^13^. Regarding the ataxia assessment, the International Cooperative Ataxia Rating Scale and Scale for the Assessment and Rating of Ataxia were applied. Autonomic function was evaluated using Composite Autonomic Symptom Score (COMPASS) 31^40^ (parts I: orthostatic intolerance, II: vasomotor, III: secretomotor, IV: gastrointestinal, V: bladder, VI: pupillomotor). Rapid eye movement sleep behavior disorder (RBD) was assessed using the RBD screening questionnaire.

Emotion was quantitatively evaluated by Hamilton Anxiety Scale and Hamilton Depression Scale. Global cognition was assessed using Mini-Mental State Examination and Montreal Cognitive Assessment. Five isolated cognitive domains, including memory, visuospatial function, attention, language, and executive function, were measured using Auditory Verbal Learning Test, Rey-Osterreich Complex Figure, Digit Span Test, Stroops Color Word Test, Verbal Fluency Test, Boston Naming Test, and Trails Making Test. In addition, family members of the participants were inquired to achieve assessments of daily living ability (Activity of Daily Living Scale, ADL), mental behavior (Mild Behavioral Impairment Checklist and Neuropsychiatric Inventory), and caring burden (Zarit Caregiver Burden Interview).

### Plasma collection and quantification

For each participant, blood samples were obtained by venipuncture and collected in ethylenediaminetetraacetate anticoagulation tubes after overnight fasting. These samples were rested for 30 min at room temperature and centrifuged at 1800 rpm for 15 min at 4 °C to isolate plasma. Then the supernatant was divided into aliquots and stored at −80 °C immediately until further processing. We measured the concentrations of plasma markers using ultrasensitive single-molecule array (Simoa) technology (Quanterix, Billerica, MA, USA) on the automated Simoa HD-X platform. Plasma NfL, GFAP, Aβ40, and Aβ42 levels were quantified using the Simoa^®^ Neurology 4-Plex E assay (catalog number: 103670), and p-tau181 levels were quantified by Simoa^TM^ pTau-181 Advantage V2 assay (Catalog Number: 103714). All samples were analyzed using the same batch of reagents and all plasma concentrations obtained were within the linear ranges of the assays. The inter- and intra-assay coefficients of variations for all assays were <20% and <10%, respectively. The variations in all specimens were lower than the control standard. The analytical lowest limit of quantification was 0.400 pg/mL for NfL, 2.89 pg/mL for GFAP, 1.02 pg/mL for Aβ40, 0.378 pg/mL for Aβ42, and 0.085 pg/mL for p-tau181. All samples tested exceeded these thresholds. Analyses were performed by board-certified laboratory technicians, blinded to clinical data.

### Image acquisition and processing

The maximum interval between plasma sampling and imaging scans was 10 days. A subgroup of 53 MSA patients received structural magnetic resonance imaging (MRI) on a 3.0 T MRI scanner (Discovery 750, GE Healthcare, Milwaukee, WI, USA). Of them, 26 patients underwent both dopamine transporter (DAT) and metabolic (^18^F-FDG) positron emission tomography (PET) on a Biograph mCT Flow PET/CT scanner (Siemens Healthcare, Erlangen, Germany).

T1-weighted MRI scans were performed with the following parameters: slice orientation, sagittal; slice thickness, 1.0 mm; slices per slab, 184; in-plane resolution, 1.0 × 1.0 mm; matrix, 256 × 256; echo time, 3.2 ms; repetition time, 8.5 ms; inversion time, 400 ms; and flip angle, 12°. The uptake of ^18^F-DTBZ and ^18^F-FDG was measured 90 and 60 min after the intravenous injection respectively and lasted for 20 min. All scans passed the visual quality control check for artifacts before processing. According to the Desikan-Killiany and ASEG atlases, each participant’s T1-weighted magnetization-prepared rapid acquisition gradient echo (MPRAGE) image within one week was segmented and parcellated with FreeSurfer, version 6.0 (Martinos Center for Biomedical Imaging) to obtain brain region volumes. PET images were then coregistered to the corresponding MPRAGE using Statistical Parametric Mapping version 12. The intensity-normalized standard uptake value ratio (SUVR) values were extracted using the cerebellum gray matter as a reference region. Data with partial volume correction under the Geometric Transfer Matrix model was considered. All imaging analyses were carried out by investigators unaware of disease status.

### Statistical analyses

Baseline demographic characteristics were compared by the Mann–Whitney *U* test or *t* test (for continuous variables) and the chi-square test (for categorical variables). Continuous data are described as mean (standard deviations), and categorical variables are presented as numbers (percentages). Raw plasma marker concentrations were nonnormally distributed due to biologically higher or lower levels, and natural logarithm transformation yielded acceptable normal distribution. To clearly convey the distributions of plasma concentrations, the plots delineated raw plasma values, although group comparisons were done using analysis of covariance on natural log-transformed values after adjusting for age and sex. Post-hoc power analyses were further conducted using the ‘pwr’ R package for all plasma biomarkers (Supplementary Table 15).

Receiver operating characteristic (ROC) analyses were used to test the ability of plasma markers in distinguishing MSA and related disorders. The accuracy for differential diagnosis was quantified as the area under the curve (AUC) and the values of sensitivity and specificity. To examine if different multimodal combinations of variables were superior to unimodal metrics, all tested plasma markers were entered into the binary logistic regression models to obtain probabilities for each individual. The desired biomarker panel was determined when the lowest Akaike information criterion value was obtained during a reverse stepwise regression procedure. A nomogram based on logistic regressions was also developed to test the risk of being diagnosed as MSA.

Associations of plasma markers with clinical scales were explored using multiple linear regressions after accounting for age and sex. Next, the relationships between significant markers and imaging features were also analyzed via multiple linear regressions (MRI analyses: adjusting for intracranial volumes; PET analyses: adjusting for age and sex). All variables entering the regression models were standardized by Z-scale beforehand using the “scale” function in R software, where z = (x-u)/s, u represents the sample mean and s represents the sample standard deviation. Furthermore, to examine whether the relationships of plasma markers with clinical phenotypes were mediated by neuroimaging indices, mediation analyses were performed. Mediation analyses were established if: (1) plasma markers were related to cerebral volumes; (2) plasma markers were related to clinical scales; (3) cerebral volumes were related to clinical scales; and (4) the associations of plasma markers with clinical scales were attenuated when cerebral volumes (the mediator) were added in the regression model. The mediation or indirect effect was estimated via the “mediate” R package, with the significance determined using 10,000 bootstrapped iterations, where the path of the model was controlled for age and sex.

All statistical analyses were carried out using R version 4.1.2 (http://www.r-project.org/). A two-tailed *p*-value <0.05 was deemed statistically significant. If corrections for multiple comparisons were considered, a more conservative significance level based on false discovery rate (FDR) correction was applied.

## Supplementary information

Supplementary Material

## Data Availability

The data used and analyzed in this study are available from the corresponding author on reasonable request.
