# Supplementary material for: Head-to-head comparison of 6 plasma biomarkers in early multiple system atrophy
Source: NPJ Parkinsons Dis. 2023 Mar 15;9:40. doi: 10.1038/s41531-023-00481-5 (PMC10017699; doi:10.1038/s41531-023-00481-5)
Supplement: Supplementary file 1 — Supplementary Material [file 41531_2023_481_MOESM1_ESM.docx]

**Supplementary Material**

Content

Supplementary Table 1: Associations of plasma biomarkers with demographic factors………….…………………………………………….………........………....1

Supplementary Table 2: Results of receiver operating characteristic (ROC) analyses…………….……………………………………………………………........………...3

Supplementary Table 3: Results of receiver operating characteristic (ROC) analyses after adjusting for age and sex………………………………………………….…….......7

Supplementary Table 4: Relationship of plasma indicators with clinical assessment scales in MSA and its subtypes…………………………………………………......10

Supplementary Table 5: Associations of plasma measures with MRI indices in MSA patients………………..……………………………………….…….…….38

Supplementary Figure 1: Plasma NfL and GFAP in DAT PET-negative versus DAT PET-positive groups………..….................................................................................….…47

Supplementary Table 6: Associations of plasma measures with PET imaging indices in MSA patients……………………………………………………………….………....48

Supplementary Table 7: Demographic characteristics and plasma patterns per diagnostic group after expanding the sample size………………………………….……..…….63

Supplementary Figure 2: Distributions of the plasma levels across diagnostic groups after expanding the sample size………………………………….……..……...............64

Supplementary Table 8: Results of receiver operating characteristic (ROC) analyses after expanding the sample size………………………………….……..……………....66

Supplementary Table 9: Relationship of plasma indicators with clinical assessment scales in MSA and its subtypes after expanding the sample size……….………..70

Supplementary Table 10: Associations of plasma measures with MRI indices in MSA patients after expanding the sample size………………………………….…….......98

Supplementary Table 11: Associations of plasma measures with PET imaging indices in MSA patients after expanding the sample size…………………………………......107

Supplementary Table 12: Demographic characteristics and plasma patterns per diagnostic group in an independent advanced disease cohort………………………….…122

Supplementary Figure 3: Distributions of the plasma levels across diagnostic groups in an independent advanced disease cohort………………….……..…….…....…..123

Supplementary Table 13: Results of receiver operating characteristic (ROC) analyses in an independent advanced disease cohort………………………………….........125

Supplementary Table 14: Receiver operating characteristic (ROC) analyses of neuroimaging indices…………………………………………….……………..….….…........127

Supplementary Table 15: Results of post-hoc power analysis………………………………………………………………………………………………………………129

**Supplementary Table 1: Associations of plasma biomarkers with demographic factors**

| Plasma measures | Demographic factors | β | Standard error | P value | P value (FDR-corrected) |
| --- | --- | --- | --- | --- | --- |
| NfL | Age | 0.010 | 0.001685411 | 2.45E-08 | 5.88E-07 |
| NfL | Male sex | -0.013 | 0.032732999 | 0.685443857 | 0.822532628 |
| NfL | Education | -0.008 | 0.004014014 | 0.054248245 | 0.130195788 |
| NfL | Disease duration | -0.001 | 0.002290234 | 0.812821603 | 0.873668884 |
| GFAP | Age | 0.008 | 0.001436922 | 2.86E-07 | 3.43E-06 |
| GFAP | Male sex | -0.083 | 0.027060594 | 0.002461619 | 0.016387176 |
| GFAP | Education | -0.009 | 0.003486396 | 0.010917677 | 0.04005384 |
| GFAP | Disease duration | 0.001 | 0.001971283 | 0.452632801 | 0.639011013 |
| P-tau181 | Age | 0.003 | 0.001362993 | 0.01168237 | 0.04005384 |
| P-tau181 | Male sex | 0.012 | 0.02472252 | 0.617393864 | 0.779865933 |
| P-tau181 | Education | -0.004 | 0.002979829 | 0.211066019 | 0.361827461 |
| P-tau181 | Disease duration | 0.004 | 0.001652266 | 0.032685473 | 0.098056419 |
| Aβ40 | Age | 0.002 | 0.00134166 | 0.125699607 | 0.232060813 |
| Aβ40 | Male sex | -0.029 | 0.024366909 | 0.239696561 | 0.383514498 |
| Aβ40 | Education | -0.006 | 0.003030984 | 0.038621718 | 0.102991248 |
| Aβ40 | Disease duration | 0.000 | 0.001325755 | 0.79664784 | 0.873668884 |
| Aβ42 | Age | 0.000 | 0.001160894 | 0.982399275 | 0.982399275 |
| Aβ42 | Male sex | -0.004 | 0.021036561 | 0.837266014 | 0.873668884 |
| Aβ42 | Education | -0.001 | 0.002650944 | 0.578065892 | 0.770754523 |
| Aβ42 | Disease duration | 0.001 | 0.001269457 | 0.400740737 | 0.601111106 |
| Aβ42/40 | Age | -0.002087798 | 0.00070549 | 0.003413995 | 0.016387176 |
| Aβ42/40 | Male sex | 0.024 | 0.012930548 | 0.060454018 | 0.131899676 |
| Aβ42/40 | Education | 0.005 | 0.001625555 | 0.003303298 | 0.016387176 |
| Aβ42/40 | Disease duration | 0.001 | 0.000764985 | 0.067148584 | 0.134297168 |

The effects of demographic factors (age, sex, education, and disease duration) on plasma markers were analyzed under the multiple linear regression model accounted for disease diagnosis. FDR-corrected p-value <0.05 was deemed statistically significant.

Abbreviations: Αβ, amyloid-β; FDR, false discovery rate; GFAP, glial fibrillary acidic protein; NfL, neurofilament light; p-tau181, phosphorylated tau at threonine 181.

**Supplementary Table 2: Results of receiver operating characteristic (ROC) analyses**

ROC analyses of single plasma biomarker

| MSA versus HC | NFL | GFAP | P-tau 181 | Aβ40 | Aβ42 | Aβ42/40 |
| --- | --- | --- | --- | --- | --- | --- |
| AUC (95% CI) | 0.930 (0.893, 0.967) | 0.636 (0.553, 0.719) | 0.723 (0.644, 0.801) | 0.753 (0.674, 0.831) | 0.605 (0.520, 0.691) | 0.719 (0.639, 0.800) |
| Cutoff | 22.680 | 51.798 | 1.422 | 67.582 | 5.505 | 0.071 |
| Sensitivity | 0.903 | 0.819 | 0.742 | 0.830 | 0.340 | 0.556 |
| Specificity | 0.860 | 0.410 | 0.639 | 0.722 | 0.833 | 0.860 |
| False positive rate | 0.140 | 0.590 | 0.361 | 0.278 | 0.167 | 0.140 |
| False negative rate | 0.097 | 0.181 | 0.258 | 0.170 | 0.660 | 0.444 |
| Positive predictive value | 0.823 | 0.500 | 0.735 | 0.806 | 0.739 | 0.741 |
| Negative predictive value | 0.925 | 0.759 | 0.648 | 0.754 | 0.476 | 0.729 |
|  |  |  |  |  |  |  |
|  |  |  |  |  |  |  |
| MSA-C versus HC | NFL | GFAP | P-tau 181 | Aβ40 | Aβ42 | Aβ42/40 |
| AUC (95% CI) | 0.940 (0.905, 0.975) | 0.664 (0.577, 0.751) | 0.742 (0.659, 0.824) | 0.725 (0.636, 0.814) | 0.582 (0.489, 0.675) | 0.728 (0.642, 0.813) |
| Cutoff | 22.680 | 51.798 | 1.422 | 67.582 | 5.505 | 0.071 |
| Sensitivity | 0.930 | 0.842 | 0.742 | 0.830 | 0.340 | 0.561 |
| Specificity | 0.860 | 0.410 | 0.684 | 0.667 | 0.807 | 0.860 |
| False positive rate | 0.140 | 0.590 | 0.316 | 0.333 | 0.193 | 0.140 |
| False negative rate | 0.070 | 0.158 | 0.258 | 0.170 | 0.660 | 0.439 |
| Positive predictive value | 0.791 | 0.449 | 0.800 | 0.814 | 0.756 | 0.696 |
| Negative predictive value | 0.956 | 0.820 | 0.609 | 0.691 | 0.411 | 0.775 |
|  |  |  |  |  |  |  |
|  |  |  |  |  |  |  |
| MSA-P versus HC | NFL | GFAP | P-tau 181 | Aβ40 | Aβ42 | Aβ42/40 |
| AUC (95% CI) | 0.893 (0.824, 0.961) | 0.531 (0.383, 0.678) | 0.651 (0.493, 0.809) | 0.858 (0.764, 0.952) | 0.695 (0.548, 0.841) | 0.688 (0.521, 0.855) |
| Cutoff | 20.248 | 53.151 | 1.044 | 67.144 | 3.418 | 0.066 |
| Sensitivity | 0.933 | 0.733 | 0.938 | 0.840 | 0.900 | 0.733 |
| Specificity | 0.810 | 0.430 | 0.333 | 0.933 | 0.467 | 0.680 |
| False positive rate | 0.190 | 0.570 | 0.667 | 0.067 | 0.533 | 0.320 |
| False negative rate | 0.067 | 0.267 | 0.062 | 0.160 | 0.100 | 0.267 |
| Positive predictive value | 0.424 | 0.162 | 0.901 | 0.988 | 0.918 | 0.256 |
| Negative predictive value | 0.988 | 0.915 | 0.455 | 0.467 | 0.412 | 0.944 |
|  |  |  |  |  |  |  |
|  |  |  |  |  |  |  |
| MSA-C versus MSA-P | NFL | GFAP | P-tau 181 | Aβ40 | Aβ42 | Aβ42/40 |
| AUC (95% CI) | 0.640 (0.462, 0.817) | 0.641 (0.486, 0.796) | 0.604 (0.440, 0.767) | 0.589 (0.438, 0.741) | 0.619 (0.450, 0.787) | 0.539 (0.370, 0.708) |
| Cutoff | 29.587 | 73.776 | 1.489 | 66.150 | 3.246 | 0.076 |
| Sensitivity | 0.667 | 0.526 | 0.533 | 0.368 | 0.860 | 0.456 |
| Specificity | 0.667 | 0.733 | 0.719 | 0.933 | 0.400 | 0.733 |
| False positive rate | 0.333 | 0.267 | 0.281 | 0.067 | 0.600 | 0.267 |
| False negative rate | 0.333 | 0.474 | 0.467 | 0.632 | 0.140 | 0.544 |
| Positive predictive value | 0.884 | 0.882 | 0.333 | 0.955 | 0.845 | 0.867 |
| Negative predictive value | 0.345 | 0.289 | 0.854 | 0.280 | 0.429 | 0.262 |
|  |  |  |  |  |  |  |
|  |  |  |  |  |  |  |
|  |  |  |  |  |  |  |
| MSA-C versus SCA | NFL | GFAP | P-tau 181 | Aβ40 | Aβ42 | Aβ42/40 |
| AUC (95% CI) | 0.598 (0.458, 0.738) | 0.717 (0.600, 0.835) | 0.553 (0.428, 0.678) | 0.570 (0.436, 0.704) | 0.567 (0.435, 0.698) | 0.586 (0.463, 0.709) |
| Cutoff | 19.362 | 81.298 | 0.937 | 72.996 | 5.532 | 0.071 |
| Sensitivity | 0.965 | 0.509 | 0.931 | 0.586 | 0.345 | 0.596 |
| Specificity | 0.310 | 0.862 | 0.246 | 0.684 | 0.807 | 0.655 |
| False positive rate | 0.690 | 0.138 | 0.754 | 0.316 | 0.193 | 0.345 |
| False negative rate | 0.035 | 0.491 | 0.069 | 0.414 | 0.655 | 0.404 |
| Positive predictive value | 0.733 | 0.879 | 0.386 | 0.486 | 0.476 | 0.773 |
| Negative predictive value | 0.818 | 0.472 | 0.875 | 0.765 | 0.708 | 0.452 |
|  |  |  |  |  |  |  |
|  |  |  |  |  |  |  |
| MSA-P versus PD | NFL | GFAP | P-tau 181 | Aβ40 | Aβ42 | Aβ42/40 |
| AUC (95% CI) | 0.662 (0.496, 0.828) | 0.757 (0.606, 0.908) | 0.540 (0.350, 0.731) | 0.807 (0.677, 0.937) | 0.769 (0.624, 0.914) | 0.552 (0.361, 0.743) |
| Cutoff | 20.248 | 77.827 | 1.047 | 65.769 | 5.681 | 0.072 |
| Sensitivity | 0.933 | 0.786 | 0.857 | 0.714 | 0.536 | 0.533 |
| Specificity | 0.571 | 0.733 | 0.333 | 0.933 | 0.933 | 0.679 |
| False positive rate | 0.429 | 0.267 | 0.667 | 0.067 | 0.067 | 0.321 |
| False negative rate | 0.067 | 0.214 | 0.143 | 0.286 | 0.464 | 0.467 |
| Positive predictive value | 0.538 | 0.846 | 0.706 | 0.952 | 0.938 | 0.471 |
| Negative predictive value | 0.941 | 0.647 | 0.556 | 0.636 | 0.519 | 0.731 |

ROC analyses of biomarker panels combining multiple variables

(Note: the tested biomarker panel in the reverse stepwise regression procedure: plasma NfL + GFAP + p-tau181 + Aβ42 + Aβ40 + Aβ42/40)

| Diagnostic groups | Derived marker panel with the lowest AIC | AUC | 95% CI | Specificity | Sensitivity | False positive rate | False negative rate |
| --- | --- | --- | --- | --- | --- | --- | --- |
| MSA versus HC | NfL + GFAP + p-tau181 + Aβ42 + Aβ40 + Aβ42/40 | 0.995 | 0.9897-1.0000 | 0.969 | 0.972 | 0.031 | 0.028 |
| MSA-C versus HC | NfL + GFAP + p-tau181 + Aβ42 + Aβ40 + Aβ42/40 | 0.997 | 0.9927-1.0000 | 0.979 | 0.982 | 0.021 | 0.018 |
| MSA-P versus HC | NfL + p-tau181 + Aβ42 + Aβ40 + Aβ42/40 | 0.990 | 0.9760-1.0000 | 0.928 | 1.000 | 0.072 | 0.000 |
| MSA-C versus MSA-P | GFAP + p-tau181 + Aβ42 + Aβ40 + Aβ42/40 | 0.759 | 0.6278-0.8903 | 0.867 | 0.596 | 0.133 | 0.404 |
| MSA-C versus SCA | GFAP + Aβ42 + Aβ42/40 | 0.764 | 0.6519-0.8762 | 0.621 | 0.789 | 0.379 | 0.211 |
| MSA-P versus PD | NfL + GFAP + Aβ40 | 0.910 | 0.8127-1.0000 | 0.929 | 0.800 | 0.071 | 0.200 |

Note: Optimal cutoff point was chosen at the highest Youden index. To examine if different multimodal combinations of variables were superior to unimodal metrics, all plasma biomarkers tested were entered into the binary logistic regression models to obtain probabilities for each individual. The desired biomarker panel was determined when the lowest AIC value was obtained during a reverse stepwise regression procedure. Only the best panels selected were shown in this study.

Abbreviations: Αβ, amyloid-β; AIC, Akaike information criterion; AUC, area under the curve; CI, confidence interval; GFAP, glial fibrillary acidic protein; HC, healthy control; MSA, multiple system atrophy; MSA-C, multiple system atrophy-cerebellar type; MSA-P, multiple system atrophy-parkinsonian type; NfL, neurofilament light; PD, Parkinson's disease; p-tau181, phosphorylated tau at threonine 181; SCA, spinocerebellar ataxia.

**Supplementary Table 3: Results of receiver operating characteristic (ROC) analyses after adjusting for age and sex**

| MSA versus HC | NFL | GFAP | P-tau 181 | Aβ40 | Aβ42 | Aβ42/40 |
| --- | --- | --- | --- | --- | --- | --- |
| AUC (95% CI) | 0.952 (0.922, 0.982) | 0.627 (0.541, 0.713) | 0.718 (0.639, 0.797) | 0.743 (0.665, 0.820) | 0.608 (0.524, 0.693) | 0.709 (0.628, 0.790) |
| Sensitivity | 0.972 | 0.681 | 0.814 | 0.700 | 0.480 | 0.597 |
| Specificity | 0.860 | 0.550 | 0.556 | 0.750 | 0.694 | 0.790 |
| False positive rate | 0.140 | 0.450 | 0.444 | 0.250 | 0.306 | 0.210 |
| False negative rate | 0.028 | 0.319 | 0.186 | 0.300 | 0.520 | 0.403 |
| Positive predictive value | 0.833 | 0.521 | 0.712 | 0.795 | 0.686 | 0.672 |
| Negative predictive value | 0.977 | 0.705 | 0.690 | 0.643 | 0.490 | 0.731 |
|  |  |  |  |  |  |  |
|  |  |  |  |  |  |  |
| MSA-C versus HC | NFL | GFAP | P-tau 181 | Aβ40 | Aβ42 | Aβ42/40 |
| AUC (95% CI) | 0.960 (0.933, 0.986) | 0.652 (0.559, 0.744) | 0.729 (0.646, 0.813) | 0.711 (0.624, 0.799) | 0.585 (0.493, 0.677) | 0.719 (0.632, 0.805) |
| Sensitivity | 0.982 | 0.737 | 0.711 | 0.750 | 0.720 | 0.614 |
| Specificity | 0.860 | 0.550 | 0.667 | 0.649 | 0.439 | 0.790 |
| False positive rate | 0.140 | 0.450 | 0.333 | 0.351 | 0.561 | 0.210 |
| False negative rate | 0.018 | 0.263 | 0.289 | 0.250 | 0.280 | 0.386 |
| Positive predictive value | 0.800 | 0.483 | 0.784 | 0.789 | 0.692 | 0.625 |
| Negative predictive value | 0.989 | 0.786 | 0.576 | 0.597 | 0.472 | 0.782 |
|  |  |  |  |  |  |  |
|  |  |  |  |  |  |  |
| MSA-P versus HC | NFL | GFAP | P-tau 181 | Aβ40 | Aβ42 | Aβ42/40 |
| AUC (95% CI) | 0.922 (0.849, 0.995) | 0.535 (0.375, 0.696) | 0.652 (0.501, 0.804) | 0.841 (0.767, 0.916) | 0.700 (0.563, 0.837) | 0.672 (0.498, 0.846) |
| Sensitivity | 0.933 | 0.933 | 0.330 | 0.720 | 0.910 | 0.733 |
| Specificity | 0.860 | 0.200 | 0.933 | 0.933 | 0.467 | 0.640 |
| False positive rate | 0.140 | 0.800 | 0.067 | 0.067 | 0.533 | 0.360 |
| False negative rate | 0.067 | 0.067 | 0.670 | 0.280 | 0.090 | 0.267 |
| Positive predictive value | 0.500 | 0.149 | 0.970 | 0.986 | 0.919 | 0.234 |
| Negative predictive value | 0.989 | 0.952 | 0.177 | 0.333 | 0.438 | 0.941 |
|  |  |  |  |  |  |  |
|  |  |  |  |  |  |  |
| MSA-C versus MSA-P | NFL | GFAP | P-tau 181 | Aβ40 | Aβ42 | Aβ42/40 |
| AUC (95% CI) | 0.600 (0.443, 0.757) | 0.675 (0.532, 0.818) | 0.594 (0.427, 0.762) | 0.611 (0.468, 0.753) | 0.627 (0.460, 0.794) | 0.535 (0.364, 0.705) |
| Sensitivity | 0.667 | 0.596 | 0.867 | 0.456 | 0.807 | 0.895 |
| Specificity | 0.533 | 0.800 | 0.368 | 0.800 | 0.467 | 0.267 |
| False positive rate | 0.467 | 0.200 | 0.632 | 0.200 | 0.533 | 0.733 |
| False negative rate | 0.333 | 0.404 | 0.133 | 0.544 | 0.193 | 0.105 |
| Positive predictive value | 0.844 | 0.919 | 0.265 | 0.897 | 0.852 | 0.823 |
| Negative predictive value | 0.296 | 0.343 | 0.913 | 0.279 | 0.389 | 0.400 |
|  |  |  |  |  |  |  |
|  |  |  |  |  |  |  |
| MSA-C versus SCA | NFL | GFAP | P-tau 181 | Aβ40 | Aβ42 | Aβ42/40 |
| AUC (95% CI) | 0.542 (0.398, 0.686) | 0.668 (0.548, 0.789) | 0.607 (0.486, 0.727) | 0.587 (0.456, 0.719) | 0.483 (0.346, 0.620) | 0.556 (0.432, 0.680) |
| Sensitivity | 0.982 | 0.702 | 0.966 | 0.483 | 0.345 | 0.561 |
| Specificity | 0.276 | 0.621 | 0.298 | 0.789 | 0.772 | 0.690 |
| False positive rate | 0.724 | 0.379 | 0.702 | 0.211 | 0.228 | 0.310 |
| False negative rate | 0.018 | 0.298 | 0.034 | 0.517 | 0.655 | 0.439 |
| Positive predictive value | 0.727 | 0.784 | 0.412 | 0.538 | 0.435 | 0.780 |
| Negative predictive value | 0.889 | 0.514 | 0.944 | 0.750 | 0.698 | 0.444 |
|  |  |  |  |  |  |  |
|  |  |  |  |  |  |  |
| MSA-P versus PD | NFL | GFAP | P-tau 181 | Aβ40 | Aβ42 | Aβ42/40 |
| AUC (95% CI) | 0.724 (0.568, 0.880) | 0.795 (0.660, 0.930) | 0.545 (0.351, 0.740) | 0.850 (0.739, 0.961) | 0.802 (0.669, 0.936) | 0.488 (0.292, 0.684) |
| Sensitivity | 0.867 | 0.750 | 0.964 | 0.679 | 0.500 | 0.467 |
| Specificity | 0.643 | 0.800 | 0.267 | 0.933 | 1.000 | 0.679 |
| False positive rate | 0.357 | 0.200 | 0.733 | 0.067 | 0.000 | 0.321 |
| False negative rate | 0.133 | 0.250 | 0.036 | 0.321 | 0.500 | 0.533 |
| Positive predictive value | 0.565 | 0.875 | 0.711 | 0.950 | 1.000 | 0.438 |
| Negative predictive value | 0.900 | 0.632 | 0.800 | 0.609 | 0.517 | 0.704 |

Abbreviations: Αβ, amyloid-β; AUC, area under the curve; CI, confidence interval; GFAP, glial fibrillary acidic protein; HC, healthy control; MSA, multiple system atrophy; MSA-C, multiple system atrophy-cerebellar type; MSA-P, multiple system atrophy-parkinsonian type; NfL, neurofilament light; PD, Parkinson's disease; p-tau181, phosphorylated tau at threonine 181; SCA, spinocerebellar ataxia.

**Supplementary Table 4: Relationship of plasma indicators with clinical assessment scales in MSA** **and its subtypes**

| Group | Plasma measures | Clinical scales | β | Standard error | t | P | P (FDR-corrected) |
| --- | --- | --- | --- | --- | --- | --- | --- |
| MSA | NfL | UMSARS-I | 0.329024695 | 0.126332767 | 2.604428788 | 0.011969852 | 0.139648273 |
| MSA | NfL | UMSARS-II | 0.249852078 | 0.123362606 | 2.025346952 | 0.04778645 | 0.209065719 |
| MSA | NfL | UMSARS-III | 0.340728481 | 0.124366943 | 2.739702963 | 0.008663676 | 0.139648273 |
| MSA | NfL | UMSARS-IV | 0.103596555 | 0.13824905 | 0.749347316 | 0.45723045 | - |
| MSA | NfL | Total UMSARS | 0.305193001 | 0.123553114 | 2.470136045 | 0.016823865 | 0.147208819 |
| MSA | NfL | ICARS | 0.124628575 | 0.165508047 | 0.753006134 | 0.455048976 | - |
| MSA | NfL | SARA | 0.089589924 | 0.169467954 | 0.528654072 | 0.599431977 | - |
| MSA | NfL | COMPASS-I | 0.084515906 | 0.127107121 | 0.664918735 | 0.508932329 | - |
| MSA | NfL | COMPASS-II | -0.012192454 | 0.130015183 | -0.093777153 | 0.92563327 | - |
| MSA | NfL | COMPASS-III | 0.195157542 | 0.135348034 | 1.441894179 | 0.155107035 | - |
| MSA | NfL | COMPASS-IV | 0.276987408 | 0.133339804 | 2.077304759 | 0.042543908 | 0.209065719 |
| MSA | NfL | COMPASS-V | 0.413268508 | 0.124898279 | 3.3088407 | 0.001672251 | 0.058528785 |
| MSA | NfL | COMPASS-VI | 0.351803166 | 0.169127964 | 2.080100527 | 0.04512432 | 0.209065719 |
| MSA | NfL | Total COMPASS | 0.209750703 | 0.127015304 | 1.651381339 | 0.104465149 | - |
| MSA | NfL | RBDSQ | 0.039570567 | 0.136841981 | 0.289169788 | 0.773671414 | - |
| MSA | NfL | HAMA | -0.055913158 | 0.156413487 | -0.357470185 | 0.722488465 | - |
| MSA | NfL | HAMD | -0.071842315 | 0.156810265 | -0.458148035 | 0.649153094 | - |
| MSA | NfL | MMSE | -0.198733631 | 0.116420295 | -1.707035978 | 0.092663126 | - |
| MSA | NfL | MoCA | -0.218970702 | 0.11250395 | -1.946337906 | 0.056006927 | - |
| MSA | NfL | AVLT-learning | 0.006386903 | 0.135551952 | 0.04711775 | 0.962590056 | - |
| MSA | NfL | AVLT-recall | -0.084142515 | 0.139542632 | -0.602987871 | 0.548995255 | - |
| MSA | NfL | AVLT-recognition | -0.140728321 | 0.147279709 | -0.955517374 | 0.344006653 | - |
| MSA | NfL | ROCF-immediate | -0.019594888 | 0.130205915 | -0.150491531 | 0.880948125 | - |
| MSA | NfL | ROCF-delay | -0.007241928 | 0.116639945 | -0.062087892 | 0.950774025 | - |
| MSA | NfL | DST | -0.043669797 | 0.130504248 | -0.334623567 | 0.739159175 | - |
| MSA | NfL | SCWT-time | 0.010699648 | 0.132275545 | 0.080889087 | 0.935846762 | - |
| MSA | NfL | SCWT-number | 0.063244879 | 0.146805675 | 0.430806771 | 0.668424229 | - |
| MSA | NfL | VFT | 0.02797286 | 0.134579722 | 0.20785345 | 0.836084273 | - |
| MSA | NfL | BNT | -0.096141352 | 0.132886 | -0.723487435 | 0.472391825 | - |
| MSA | NfL | TMT-A | 0.052742606 | 0.118635233 | 0.44457793 | 0.658854504 | - |
| MSA | NfL | TMT-B | 0.002160388 | 0.169110874 | 0.012774982 | 0.989862562 | - |
| MSA | NfL | ADL | 0.271624527 | 0.126428037 | 2.148451668 | 0.036017062 | 0.209065719 |
| MSA | NfL | MBI-C | 0.041701986 | 0.136425141 | 0.305676695 | 0.761026122 | - |
| MSA | NfL | NPI | 0.101716227 | 0.137356683 | 0.740526234 | 0.462311525 | - |
| MSA | NfL | ZBI | 0.060957446 | 0.136845753 | 0.445446385 | 0.657919944 | - |

| Group | Plasma measures | Clinical scales | β | Standard error | t | P | P (FDR-corrected) |
| --- | --- | --- | --- | --- | --- | --- | --- |
| MSA | GFAP | UMSARS-I | 0.111628191 | 0.132226909 | 0.844216899 | 0.402415449 | - |
| MSA | GFAP | UMSARS-II | 0.09421183 | 0.126702048 | 0.743569906 | 0.460359847 | - |
| MSA | GFAP | UMSARS-III | -0.086603978 | 0.134501029 | -0.643890824 | 0.522776088 | - |
| MSA | GFAP | UMSARS-IV | 0.356493919 | 0.128056426 | 2.783881523 | 0.007611026 | 0.26638591 |
| MSA | GFAP | Total UMSARS | 0.109875516 | 0.12854393 | 0.854770165 | 0.396599492 | - |
| MSA | GFAP | ICARS | -0.002544317 | 0.141367232 | -0.017997928 | 0.985713593 | - |
| MSA | GFAP | SARA | 0.039553524 | 0.144218914 | 0.274260307 | 0.785037533 | - |
| MSA | GFAP | COMPASS-I | -0.045420541 | 0.126464319 | -0.359156963 | 0.720878272 | - |
| MSA | GFAP | COMPASS-II | -0.11041529 | 0.128117549 | -0.861827991 | 0.392593231 | - |
| MSA | GFAP | COMPASS-III | 0.011175126 | 0.136827758 | 0.081672948 | 0.935208765 | - |
| MSA | GFAP | COMPASS-IV | 0.038765582 | 0.137366041 | 0.282206446 | 0.778864033 | - |
| MSA | GFAP | COMPASS-V | 0.069284244 | 0.135563149 | 0.511084645 | 0.611375165 | - |
| MSA | GFAP | COMPASS-VI | -0.102995001 | 0.170688115 | -0.6034105 | 0.550240277 | - |
| MSA | GFAP | Total COMPASS | -0.025003447 | 0.129106809 | -0.19366482 | 0.847164993 | - |
| MSA | GFAP | RBDSQ | -0.0246862 | 0.135704056 | -0.181912032 | 0.856402236 | - |
| MSA | GFAP | HAMA | 0.118179111 | 0.195894966 | 0.603277939 | 0.54949292 | - |
| MSA | GFAP | HAMD | 0.090940415 | 0.196921186 | 0.461811227 | 0.646544675 | - |
| MSA | GFAP | MMSE | -0.015074456 | 0.121723543 | -0.123841743 | 0.901828706 | - |
| MSA | GFAP | MoCA | -0.074221783 | 0.118044895 | -0.62875894 | 0.531744634 | - |
| MSA | GFAP | AVLT-learning | 0.018198511 | 0.128499505 | 0.141623197 | 0.887895127 | - |
| MSA | GFAP | AVLT-recall | -0.040546322 | 0.134104315 | -0.302349125 | 0.763526404 | - |
| MSA | GFAP | AVLT-recognition | 0.086962484 | 0.163518644 | 0.531819993 | 0.597253739 | - |
| MSA | GFAP | ROCF-immediate | 0.099436959 | 0.121726776 | 0.816886488 | 0.417650165 | - |
| MSA | GFAP | ROCF-delay | 0.189560375 | 0.110261902 | 1.719182891 | 0.092609896 | - |
| MSA | GFAP | DST | 0.08418088 | 0.124109584 | 0.678278645 | 0.500388336 | - |
| MSA | GFAP | SCWT-time | 0.008631758 | 0.13080234 | 0.065990851 | 0.947643122 | - |
| MSA | GFAP | SCWT-number | 0.012082993 | 0.145421586 | 0.083089408 | 0.934105721 | - |
| MSA | GFAP | VFT | -0.122848225 | 0.128687119 | -0.954627205 | 0.343797391 | - |
| MSA | GFAP | BNT | -0.000366551 | 0.126862639 | -0.002889357 | 0.997704898 | - |
| MSA | GFAP | TMT-A | -0.01795233 | 0.110922615 | -0.161845532 | 0.872185603 | - |
| MSA | GFAP | TMT-B | 0.143166704 | 0.129131879 | 1.108685989 | 0.273328723 | - |
| MSA | GFAP | ADL | 0.05408013 | 0.125570311 | 0.430676088 | 0.668357049 | - |
| MSA | GFAP | MBI-C | 0.006130781 | 0.135529347 | 0.045235818 | 0.964086216 | - |
| MSA | GFAP | NPI | -0.035422288 | 0.140461512 | -0.252185014 | 0.801892192 | - |
| MSA | GFAP | ZBI | -0.168826166 | 0.132642315 | -1.272792667 | 0.208978283 | - |

| Group | Plasma measures | Clinical scales | β | Standard error | t | P | P (FDR-corrected) |
| --- | --- | --- | --- | --- | --- | --- | --- |
| MSA | Aβ42 | UMSARS-I | 0.050042141 | 0.138457458 | 0.36142611 | 0.719245358 | - |
| MSA | Aβ42 | UMSARS-II | 0.062142701 | 0.13050531 | 0.476169903 | 0.635874332 | - |
| MSA | Aβ42 | UMSARS-III | 0.120632938 | 0.146206784 | 0.825084407 | 0.413491875 | - |
| MSA | Aβ42 | UMSARS-IV | 0.265986555 | 0.143418904 | 1.854612943 | 0.069674138 | - |
| MSA | Aβ42 | Total UMSARS | 0.0697481 | 0.134445338 | 0.518784075 | 0.606112187 | - |
| MSA | Aβ42 | ICARS | 0.010824626 | 0.15964164 | 0.067805778 | 0.946216177 | - |
| MSA | Aβ42 | SARA | 0.047425433 | 0.16285315 | 0.291215937 | 0.772115384 | - |
| MSA | Aβ42 | COMPASS-I | -0.074027517 | 0.130260946 | -0.568301698 | 0.572185908 | - |
| MSA | Aβ42 | COMPASS-II | -0.014659237 | 0.133091168 | -0.110144325 | 0.912703248 | - |
| MSA | Aβ42 | COMPASS-III | 0.182811865 | 0.13898765 | 1.315310135 | 0.193963492 | - |
| MSA | Aβ42 | COMPASS-IV | 0.267051532 | 0.137113461 | 1.947668221 | 0.056661476 | - |
| MSA | Aβ42 | COMPASS-V | 0.176685006 | 0.138144027 | 1.278991278 | 0.206369178 | - |
| MSA | Aβ42 | COMPASS-VI | 0.282685638 | 0.161468117 | 1.750721087 | 0.089013374 | - |
| MSA | Aβ42 | Total COMPASS | 0.041392195 | 0.133147992 | 0.310873593 | 0.75709358 | - |
| MSA | Aβ42 | RBDSQ | 0.050575712 | 0.145968723 | 0.346483216 | 0.730463102 | - |
| MSA | Aβ42 | HAMA | -0.149383274 | 0.160265092 | -0.932101135 | 0.356488537 | - |
| MSA | Aβ42 | HAMD | -0.116233713 | 0.161471133 | -0.719842057 | 0.475516899 | - |
| MSA | Aβ42 | MMSE | 0.032800285 | 0.117388917 | 0.279415521 | 0.78082697 | - |
| MSA | Aβ42 | MoCA | 0.005467274 | 0.114246231 | 0.047855181 | 0.961980684 | - |
| MSA | Aβ42 | AVLT-learning | 0.09180542 | 0.129166248 | 0.71075394 | 0.480240008 | - |
| MSA | Aβ42 | AVLT-recall | -0.118853348 | 0.135635156 | -0.876272429 | 0.384693173 | - |
| MSA | Aβ42 | AVLT-recognition | 0.020121572 | 0.143664979 | 0.14005899 | 0.889187525 | - |
| MSA | Aβ42 | ROCF-immediate | -0.000321533 | 0.127965575 | -0.002512655 | 0.998004628 | - |
| MSA | Aβ42 | ROCF-delay | 0.149411722 | 0.114474746 | 1.30519374 | 0.198611306 | - |
| MSA | Aβ42 | DST | 0.051127131 | 0.124693629 | 0.410021997 | 0.683354205 | - |
| MSA | Aβ42 | SCWT-time | -0.126136162 | 0.130323924 | -0.967866513 | 0.337678684 | - |
| MSA | Aβ42 | SCWT-number | 0.050204293 | 0.146048866 | 0.343749969 | 0.732447533 | - |
| MSA | Aβ42 | VFT | 0.077890539 | 0.130103756 | 0.598680176 | 0.55175804 | - |
| MSA | Aβ42 | BNT | 0.024627122 | 0.130750072 | 0.188352646 | 0.851281119 | - |
| MSA | Aβ42 | TMT-A | -0.0479261 | 0.112475624 | -0.426102101 | 0.672158918 | - |
| MSA | Aβ42 | TMT-B | -0.084751778 | 0.14345756 | -0.590779444 | 0.557559926 | - |
| MSA | Aβ42 | ADL | 0.035001962 | 0.124017841 | 0.282233277 | 0.778805132 | - |
| MSA | Aβ42 | MBI-C | 0.032641334 | 0.137487974 | 0.237412281 | 0.81323541 | - |
| MSA | Aβ42 | NPI | 0.048571056 | 0.138506885 | 0.350676113 | 0.727248034 | - |
| MSA | Aβ42 | ZBI | -0.002272538 | 0.144220685 | -0.015757367 | 0.98749067 | - |

| Group | Plasma measures | Clinical scales | β | Standard error | t | P | P (FDR-corrected) |
| --- | --- | --- | --- | --- | --- | --- | --- |
| MSA | Aβ40 | UMSARS-I | 0.104320603 | 0.137459806 | 0.758917143 | 0.451328587 | - |
| MSA | Aβ40 | UMSARS-II | 0.071493831 | 0.129006749 | 0.554186748 | 0.581738954 | - |
| MSA | Aβ40 | UMSARS-III | 0.127633121 | 0.141271229 | 0.903461532 | 0.37088844 | - |
| MSA | Aβ40 | UMSARS-IV | 0.237986676 | 0.139761798 | 1.702802052 | 0.094941404 | - |
| MSA | Aβ40 | Total UMSARS | 0.105992609 | 0.133585725 | 0.793442627 | 0.431125712 | - |
| MSA | Aβ40 | ICARS | 0.145997032 | 0.187905053 | 0.776972355 | 0.440909987 | - |
| MSA | Aβ40 | SARA | 0.148001859 | 0.1918586 | 0.771411129 | 0.444167545 | - |
| MSA | Aβ40 | COMPASS-I | -0.130276933 | 0.131003265 | -0.99445562 | 0.324436511 | - |
| MSA | Aβ40 | COMPASS-II | -0.088912511 | 0.134138122 | -0.662842971 | 0.510250664 | - |
| MSA | Aβ40 | COMPASS-III | 0.097938498 | 0.14224594 | 0.68851524 | 0.494075854 | - |
| MSA | Aβ40 | COMPASS-IV | 0.233307038 | 0.139972296 | 1.666808676 | 0.101343036 | - |
| MSA | Aβ40 | COMPASS-V | 0.273300509 | 0.136920426 | 1.996053606 | 0.050981423 | - |
| MSA | Aβ40 | COMPASS-VI | 0.167388581 | 0.179767449 | 0.931139544 | 0.358346305 | - |
| MSA | Aβ40 | Total COMPASS | -0.016635524 | 0.13482658 | -0.123384603 | 0.902260652 | - |
| MSA | Aβ40 | RBDSQ | 0.139649698 | 0.140273925 | 0.995549941 | 0.324359928 | - |
| MSA | Aβ40 | HAMA | -0.12763386 | 0.182285109 | -0.700188077 | 0.487581132 | - |
| MSA | Aβ40 | HAMD | -0.152735116 | 0.182480754 | -0.83699301 | 0.40722429 | - |
| MSA | Aβ40 | MMSE | -0.010773304 | 0.118520481 | -0.090898245 | 0.927857344 | - |
| MSA | Aβ40 | MoCA | -0.01703938 | 0.115267037 | -0.147825265 | 0.882945414 | - |
| MSA | Aβ40 | AVLT-learning | 0.096012668 | 0.128230263 | 0.748752014 | 0.457196936 | - |
| MSA | Aβ40 | AVLT-recall | -0.054355604 | 0.136995989 | -0.396767851 | 0.69307405 | - |
| MSA | Aβ40 | AVLT-recognition | 0.096757288 | 0.142213013 | 0.680368733 | 0.499473292 | - |
| MSA | Aβ40 | ROCF-immediate | 0.066664935 | 0.126395816 | 0.527429923 | 0.600096139 | - |
| MSA | Aβ40 | ROCF-delay | 0.139304131 | 0.112794422 | 1.235026773 | 0.223375026 | - |
| MSA | Aβ40 | DST | 0.05820866 | 0.125426671 | 0.464085185 | 0.644385794 | - |
| MSA | Aβ40 | SCWT-time | -0.068074127 | 0.130454892 | -0.521821188 | 0.60405388 | - |
| MSA | Aβ40 | SCWT-number | -0.027939181 | 0.145372973 | -0.192189653 | 0.848356874 | - |
| MSA | Aβ40 | VFT | 0.127431038 | 0.127415035 | 1.000125598 | 0.321476686 | - |
| MSA | Aβ40 | BNT | 0.03904137 | 0.131137533 | 0.297713163 | 0.767023803 | - |
| MSA | Aβ40 | TMT-A | -0.037917493 | 0.112343132 | -0.337515006 | 0.737370818 | - |
| MSA | Aβ40 | TMT-B | 0.165449661 | 0.136495274 | 1.212127397 | 0.231652883 | - |
| MSA | Aβ40 | ADL | 0.054741511 | 0.125767315 | 0.435260238 | 0.665046535 | - |
| MSA | Aβ40 | MBI-C | 0.02374014 | 0.137732889 | 0.172363627 | 0.863796049 | - |
| MSA | Aβ40 | NPI | -0.023910879 | 0.140345519 | -0.170371516 | 0.86537913 | - |
| MSA | Aβ40 | ZBI | 0.040275486 | 0.145890567 | 0.276066414 | 0.783634933 | - |

| Group | Plasma measures | Clinical scales | β | Standard error | t | P | P (FDR-corrected) |
| --- | --- | --- | --- | --- | --- | --- | --- |
| MSA | Aβ42/40 | UMSARS-I | -0.113748546 | 0.138382663 | -0.821985527 | 0.414838296 | - |
| MSA | Aβ42/40 | UMSARS-II | -0.027398297 | 0.131778163 | -0.207912267 | 0.836079635 | - |
| MSA | Aβ42/40 | UMSARS-III | 0.022516827 | 0.148141336 | 0.151995572 | 0.879841022 | - |
| MSA | Aβ42/40 | UMSARS-IV | 0.011275954 | 0.144387236 | 0.078095229 | 0.938070343 | - |
| MSA | Aβ42/40 | Total UMSARS | -0.079558762 | 0.134972255 | -0.5894453 | 0.558115031 | - |
| MSA | Aβ42/40 | ICARS | -0.221667704 | 0.170431388 | -1.300627235 | 0.201650613 | - |
| MSA | Aβ42/40 | SARA | -0.156935472 | 0.175188418 | -0.895809631 | 0.376305552 | - |
| MSA | Aβ42/40 | COMPASS-I | 0.116486596 | 0.126353119 | 0.9219131 | 0.360674657 | - |
| MSA | Aβ42/40 | COMPASS-II | 0.212197681 | 0.126483236 | 1.677674353 | 0.099190264 | - |
| MSA | Aβ42/40 | COMPASS-III | 0.179879561 | 0.135428876 | 1.328221617 | 0.189691493 | - |
| MSA | Aβ42/40 | COMPASS-IV | 0.078552409 | 0.137844178 | 0.569863813 | 0.571133389 | - |
| MSA | Aβ42/40 | COMPASS-V | -0.117215107 | 0.13573829 | -0.863537525 | 0.39166141 | - |
| MSA | Aβ42/40 | COMPASS-VI | 0.198087845 | 0.186588643 | 1.061628628 | 0.295887382 | - |
| MSA | Aβ42/40 | Total COMPASS | 0.133918895 | 0.128609784 | 1.041280771 | 0.302384955 | - |
| MSA | Aβ42/40 | RBDSQ | -0.160405765 | 0.140377805 | -1.142671842 | 0.258727512 | - |
| MSA | Aβ42/40 | HAMA | -0.037529901 | 0.148744223 | -0.252311653 | 0.802002357 | - |
| MSA | Aβ42/40 | HAMD | -0.008719466 | 0.149367973 | -0.058375742 | 0.953719781 | - |
| MSA | Aβ42/40 | MMSE | 0.135312331 | 0.12042075 | 1.123662911 | 0.265353289 | - |
| MSA | Aβ42/40 | MoCA | 0.086645043 | 0.117780379 | 0.735649206 | 0.464631786 | - |
| MSA | Aβ42/40 | AVLT-learning | -0.004307962 | 0.129932745 | -0.033155321 | 0.973670759 | - |
| MSA | Aβ42/40 | AVLT-recall | -0.107186252 | 0.135217095 | -0.792697489 | 0.431360256 | - |
| MSA | Aβ42/40 | AVLT-recognition | -0.182360833 | 0.144564743 | -1.261447498 | 0.213120073 | - |
| MSA | Aβ42/40 | ROCF-immediate | -0.032819588 | 0.123027856 | -0.266765501 | 0.790684937 | - |
| MSA | Aβ42/40 | ROCF-delay | -0.028860659 | 0.113152765 | -0.25505925 | 0.799865795 | - |
| MSA | Aβ42/40 | DST | 0.055607055 | 0.125123538 | 0.44441722 | 0.658453777 | - |
| MSA | Aβ42/40 | SCWT-time | -0.070219355 | 0.125650081 | -0.558848464 | 0.578710871 | - |
| MSA | Aβ42/40 | SCWT-number | 0.050876086 | 0.139942837 | 0.363549058 | 0.717697484 | - |
| MSA | Aβ42/40 | VFT | -0.055163283 | 0.128474985 | -0.429369836 | 0.66927277 | - |
| MSA | Aβ42/40 | BNT | 0.012510025 | 0.12555255 | 0.099639753 | 0.920986178 | - |
| MSA | Aβ42/40 | TMT-A | -0.009603709 | 0.119634392 | -0.080275483 | 0.936390616 | - |
| MSA | Aβ42/40 | TMT-B | -0.343716878 | 0.12495215 | -2.750788022 | 0.0084737 | 0.2965795 |
| MSA | Aβ42/40 | ADL | 0.010398398 | 0.131019298 | 0.079365392 | 0.93702493 | - |
| MSA | Aβ42/40 | MBI-C | -0.005978436 | 0.136996378 | -0.04363937 | 0.96535284 | - |
| MSA | Aβ42/40 | NPI | 0.14035913 | 0.144473884 | 0.971519046 | 0.335787437 | - |
| MSA | Aβ42/40 | ZBI | -0.00511828 | 0.137011552 | -0.037356557 | 0.970349463 | - |

| Group | Plasma measures | Clinical scales | β | Standard error | t | P | P (FDR-corrected) |
| --- | --- | --- | --- | --- | --- | --- | --- |
| MSA | P-tau181 | UMSARS-I | 0.034269687 | 0.128053801 | 0.267619444 | 0.790050923 | - |
| MSA | P-tau181 | UMSARS-II | 0.029578671 | 0.119978201 | 0.24653371 | 0.806203904 | - |
| MSA | P-tau181 | UMSARS-III | 0.020239477 | 0.141765072 | 0.142767725 | 0.887083931 | - |
| MSA | P-tau181 | UMSARS-IV | 0.100466644 | 0.135686734 | 0.740430849 | 0.462571939 | - |
| MSA | P-tau181 | Total UMSARS | 0.027351603 | 0.124536193 | 0.219627744 | 0.827020971 | - |
| MSA | P-tau181 | ICARS | 0.331534497 | 0.137298958 | 2.414690552 | 0.105952697 | - |
| MSA | P-tau181 | SARA | 0.393822419 | 0.137193702 | 2.870557568 | 0.206034485 | - |
| MSA | P-tau181 | COMPASS-I | -0.023990758 | 0.124189004 | -0.193179405 | 0.847543236 | - |
| MSA | P-tau181 | COMPASS-II | -0.144107965 | 0.125038985 | -1.152504276 | 0.254190476 | - |
| MSA | P-tau181 | COMPASS-III | -0.156233819 | 0.132566393 | -1.17853262 | 0.243751566 | - |
| MSA | P-tau181 | COMPASS-IV | 0.022125004 | 0.134846049 | 0.164076024 | 0.870283892 | - |
| MSA | P-tau181 | COMPASS-V | 0.238182477 | 0.129333006 | 1.841621752 | 0.071025353 | - |
| MSA | P-tau181 | COMPASS-VI | 0.089015855 | 0.16123462 | 0.552088966 | 0.584498523 | - |
| MSA | P-tau181 | Total COMPASS | -0.01362084 | 0.12670697 | -0.107498743 | 0.914791735 | - |
| MSA | P-tau181 | RBDSQ | 0.040045588 | 0.13428047 | 0.298223472 | 0.766793571 | - |
| MSA | P-tau181 | HAMA | 0.19366758 | 0.160348902 | 1.207788622 | 0.23372975 | - |
| MSA | P-tau181 | HAMD | 0.01099568 | 0.163606645 | 0.067208029 | 0.946727627 | - |
| MSA | P-tau181 | MMSE | -0.064860215 | 0.112822233 | -0.574888599 | 0.567382171 | - |
| MSA | P-tau181 | MoCA | -0.0493816 | 0.109846535 | -0.449550822 | 0.6545532 | - |
| MSA | P-tau181 | AVLT-learning | 0.187470711 | 0.124582364 | 1.50479334 | 0.138098718 | - |
| MSA | P-tau181 | AVLT-recall | 0.150216434 | 0.129310314 | 1.161674035 | 0.250384512 | - |
| MSA | P-tau181 | AVLT-recognition | 0.041107461 | 0.150596769 | 0.272963762 | 0.78602822 | - |
| MSA | P-tau181 | ROCF-immediate | -0.091024955 | 0.118498411 | -0.768153379 | 0.445805253 | - |
| MSA | P-tau181 | ROCF-delay | -0.085357 | 0.111577879 | -0.764999307 | 0.448355828 | - |
| MSA | P-tau181 | DST | 0.04437028 | 0.12179052 | 0.364316367 | 0.716993847 | - |
| MSA | P-tau181 | SCWT-time | 0.189219271 | 0.121755267 | 1.554095158 | 0.126345986 | - |
| MSA | P-tau181 | SCWT-number | 0.123372027 | 0.137453588 | 0.897554066 | 0.373641062 | - |
| MSA | P-tau181 | VFT | 0.045330466 | 0.125571698 | 0.360992694 | 0.719439439 | - |
| MSA | P-tau181 | BNT | -0.003124458 | 0.121362495 | -0.025744839 | 0.979552383 | - |
| MSA | P-tau181 | TMT-A | 0.106628693 | 0.111649775 | 0.955028284 | 0.344901843 | - |
| MSA | P-tau181 | TMT-B | 0.216218038 | 0.122016585 | 1.772038099 | 0.083011672 | - |
| MSA | P-tau181 | ADL | -0.042186623 | 0.115634934 | -0.364825939 | 0.716615522 | - |
| MSA | P-tau181 | MBI-C | -0.036070735 | 0.135567124 | -0.266072876 | 0.791196381 | - |
| MSA | P-tau181 | NPI | -0.138294144 | 0.140796975 | -0.982223832 | 0.330537659 | - |
| MSA | P-tau181 | ZBI | -0.008273825 | 0.142728145 | -0.057969118 | 0.954004283 | - |

| Group | Plasma measures | Clinical scales | β | Standard error | t | P | P (FDR-corrected) |
| --- | --- | --- | --- | --- | --- | --- | --- |
| MSA-C | NfL | UMSARS-I | 0.295443264 | 0.13983588 | 2.112785827 | 0.040752757 | 0.361210185 |
| MSA-C | NfL | UMSARS-II | 0.181061942 | 0.138397789 | 1.308271929 | 0.197895885 | - |
| MSA-C | NfL | UMSARS-III | 0.333805377 | 0.139939942 | 2.385347398 | 0.022297133 | 0.361210185 |
| MSA-C | NfL | UMSARS-IV | 0.071948011 | 0.154478976 | 0.465746294 | 0.643987779 | - |
| MSA-C | NfL | Total UMSARS | 0.255427099 | 0.137972552 | 1.851289222 | 0.071340081 | - |
| MSA-C | NfL | ICARS | 0.001657663 | 0.191041319 | 0.008676989 | 0.993122246 | - |
| MSA-C | NfL | SARA | -0.074038684 | 0.194568265 | -0.380528059 | 0.705672415 | - |
| MSA-C | NfL | COMPASS-I | 0.118412125 | 0.134440551 | 0.880776848 | 0.383335164 | - |
| MSA-C | NfL | COMPASS-II | -0.067663791 | 0.13509225 | -0.500871002 | 0.619016249 | - |
| MSA-C | NfL | COMPASS-III | 0.238074494 | 0.140596414 | 1.693318401 | 0.097628526 | - |
| MSA-C | NfL | COMPASS-IV | 0.296739333 | 0.141048767 | 2.103806652 | 0.041281164 | 0.361210185 |
| MSA-C | NfL | COMPASS-V | 0.457295753 | 0.133513571 | 3.42508817 | 0.00136271 | 0.04769485 |
| MSA-C | NfL | COMPASS-VI | 0.220480532 | 0.18133631 | 1.215865326 | 0.234960756 | - |
| MSA-C | NfL | Total COMPASS | 0.242132255 | 0.133114746 | 1.818973941 | 0.075883728 | - |
| MSA-C | NfL | RBDSQ | 0.020097868 | 0.146638657 | 0.137057094 | 0.891690788 | - |
| MSA-C | NfL | HAMA | -0.143445952 | 0.174143306 | -0.82372361 | 0.416389154 | - |
| MSA-C | NfL | HAMD | -0.154975474 | 0.172188281 | -0.900034966 | 0.375040511 | - |
| MSA-C | NfL | MMSE | -0.194314562 | 0.136141848 | -1.427294875 | 0.159836897 | - |
| MSA-C | NfL | MoCA | -0.217694131 | 0.131687684 | -1.653109264 | 0.104702505 | - |
| MSA-C | NfL | AVLT-learning | 0.038056386 | 0.146446488 | 0.259865477 | 0.796207799 | - |
| MSA-C | NfL | AVLT-recall | -0.017099632 | 0.154420632 | -0.110734112 | 0.912342438 | - |
| MSA-C | NfL | AVLT-recognition | -0.199329329 | 0.156581248 | -1.27300894 | 0.210748132 | - |
| MSA-C | NfL | ROCF-immediate | -0.049794554 | 0.145472657 | -0.342294937 | 0.733877505 | - |
| MSA-C | NfL | ROCF-delay | -0.035966559 | 0.12404687 | -0.289943304 | 0.773620594 | - |
| MSA-C | NfL | DST | -0.085384334 | 0.144937804 | -0.589110169 | 0.558800052 | - |
| MSA-C | NfL | SCWT-time | -0.053642038 | 0.141765191 | -0.378386523 | 0.707144328 | - |
| MSA-C | NfL | SCWT-number | 0.058427876 | 0.161129089 | 0.36261532 | 0.718801307 | - |
| MSA-C | NfL | VFT | 0.099002312 | 0.147570605 | 0.670880981 | 0.505651155 | - |
| MSA-C | NfL | BNT | -0.125828145 | 0.150469772 | -0.836235368 | 0.40754107 | - |
| MSA-C | NfL | TMT-A | 0.025985874 | 0.128917126 | 0.201570379 | 0.841419573 | - |
| MSA-C | NfL | TMT-B | 0.023769632 | 0.187318609 | 0.126894133 | 0.8997113 | - |
| MSA-C | NfL | ADL | 0.274731218 | 0.139176619 | 1.973975362 | 0.054683464 | - |
| MSA-C | NfL | MBI-C | 0.063545437 | 0.146409444 | 0.434025533 | 0.666439927 | - |
| MSA-C | NfL | NPI | 0.108191114 | 0.149260426 | 0.724847952 | 0.472662001 | - |
| MSA-C | NfL | ZBI | -0.01039168 | 0.155036044 | -0.06702751 | 0.946911233 | - |

| Group | Plasma measures | Clinical scales | β | Standard error | t | P | P (FDR-corrected) |
| --- | --- | --- | --- | --- | --- | --- | --- |
| MSA-C | GFAP | UMSARS-I | 0.157660771 | 0.148552633 | 1.061312531 | 0.294759839 | - |
| MSA-C | GFAP | UMSARS-II | 0.130196402 | 0.143097904 | 0.909841434 | 0.368098174 | - |
| MSA-C | GFAP | UMSARS-III | -0.085148056 | 0.153232464 | -0.555678959 | 0.581775078 | - |
| MSA-C | GFAP | UMSARS-IV | 0.392933456 | 0.143494233 | 2.738322286 | 0.009258181 | 0.324036335 |
| MSA-C | GFAP | Total UMSARS | 0.155109245 | 0.144857901 | 1.07076828 | 0.290536711 | - |
| MSA-C | GFAP | ICARS | -0.021670023 | 0.161896671 | -0.133850946 | 0.89422683 | - |
| MSA-C | GFAP | SARA | 0.017253689 | 0.165214481 | 0.10443206 | 0.91737575 | - |
| MSA-C | GFAP | COMPASS-I | -0.05305497 | 0.139350061 | -0.380731585 | 0.705276941 | - |
| MSA-C | GFAP | COMPASS-II | -0.186920042 | 0.136472795 | -1.369650571 | 0.177906564 | - |
| MSA-C | GFAP | COMPASS-III | 0.024672771 | 0.149375376 | 0.165172948 | 0.869581947 | - |
| MSA-C | GFAP | COMPASS-IV | 0.022196025 | 0.152391671 | 0.145651171 | 0.884877409 | - |
| MSA-C | GFAP | COMPASS-V | 0.066296607 | 0.154670673 | 0.428630755 | 0.670331625 | - |
| MSA-C | GFAP | COMPASS-VI | -0.152545545 | 0.184861664 | -0.82518756 | 0.416772366 | - |
| MSA-C | GFAP | Total COMPASS | -0.03708914 | 0.142037831 | -0.261121556 | 0.795245375 | - |
| MSA-C | GFAP | RBDSQ | -0.035570316 | 0.148245013 | -0.239942747 | 0.811631349 | - |
| MSA-C | GFAP | HAMA | 0.206872547 | 0.234712188 | 0.881388176 | 0.38489069 | - |
| MSA-C | GFAP | HAMD | 0.112867666 | 0.234580125 | 0.481147609 | 0.633790436 | - |
| MSA-C | GFAP | MMSE | -0.116420652 | 0.143244338 | -0.812741732 | 0.42029753 | - |
| MSA-C | GFAP | MoCA | -0.169087156 | 0.138342963 | -1.222231715 | 0.227466948 | - |
| MSA-C | GFAP | AVLT-learning | -0.048338665 | 0.144151137 | -0.335333222 | 0.73900429 | - |
| MSA-C | GFAP | AVLT-recall | -0.059486188 | 0.153358979 | -0.387888522 | 0.700012155 | - |
| MSA-C | GFAP | AVLT-recognition | 0.028235003 | 0.182834843 | 0.154429006 | 0.878088784 | - |
| MSA-C | GFAP | ROCF-immediate | 0.004908797 | 0.142669806 | 0.034406701 | 0.972719806 | - |
| MSA-C | GFAP | ROCF-delay | 0.150744803 | 0.122680047 | 1.228763817 | 0.227597632 | - |
| MSA-C | GFAP | DST | 0.066057084 | 0.143771704 | 0.45945817 | 0.648167267 | - |
| MSA-C | GFAP | SCWT-time | 0.020342747 | 0.144850061 | 0.140440032 | 0.88901717 | - |
| MSA-C | GFAP | SCWT-number | -0.014315654 | 0.164636379 | -0.086953165 | 0.931142577 | - |
| MSA-C | GFAP | VFT | -0.173215598 | 0.145529585 | -1.190243201 | 0.240057376 | - |
| MSA-C | GFAP | BNT | -0.080116473 | 0.149581641 | -0.535603647 | 0.59492965 | - |
| MSA-C | GFAP | TMT-A | -0.04431641 | 0.124064516 | -0.357204555 | 0.723083564 | - |
| MSA-C | GFAP | TMT-B | 0.162477886 | 0.143433736 | 1.132773157 | 0.264596965 | - |
| MSA-C | GFAP | ADL | 0.135091259 | 0.143309467 | 0.942654116 | 0.351005139 | - |
| MSA-C | GFAP | MBI-C | -0.013140094 | 0.151273956 | -0.086862898 | 0.931183733 | - |
| MSA-C | GFAP | NPI | -0.059578238 | 0.158184576 | -0.376637467 | 0.708385285 | - |
| MSA-C | GFAP | ZBI | -0.172240308 | 0.155585332 | -1.107047213 | 0.275232327 | - |

| Group | Plasma measures | Clinical scales | β | Standard error | t | P | P (FDR-corrected) |
| --- | --- | --- | --- | --- | --- | --- | --- |
| MSA-C | Aβ42 | UMSARS-I | 0.076564953 | 0.156878459 | 0.488052686 | 0.628112513 | - |
| MSA-C | Aβ42 | UMSARS-II | 0.091806512 | 0.1479841 | 0.620380918 | 0.538358946 | - |
| MSA-C | Aβ42 | UMSARS-III | 0.149647542 | 0.170554007 | 0.877420265 | 0.385920317 | - |
| MSA-C | Aβ42 | UMSARS-IV | 0.35265136 | 0.161691222 | 2.181017356 | 0.055276432 | - |
| MSA-C | Aβ42 | Total UMSARS | 0.100770934 | 0.152647959 | 0.660152514 | 0.512847831 | - |
| MSA-C | Aβ42 | ICARS | -0.075944767 | 0.191818873 | -0.395919156 | 0.694378972 | - |
| MSA-C | Aβ42 | SARA | -0.01962125 | 0.196109301 | -0.100052623 | 0.920828553 | - |
| MSA-C | Aβ42 | COMPASS-I | -0.068174745 | 0.146876323 | -0.464164298 | 0.644871509 | - |
| MSA-C | Aβ42 | COMPASS-II | -0.091017956 | 0.1464114 | -0.621658943 | 0.537448325 | - |
| MSA-C | Aβ42 | COMPASS-III | 0.173172444 | 0.155393604 | 1.114411656 | 0.271292854 | - |
| MSA-C | Aβ42 | COMPASS-IV | 0.262843544 | 0.155716918 | 1.687957527 | 0.098660745 | - |
| MSA-C | Aβ42 | COMPASS-V | 0.31397358 | 0.156338005 | 2.008299769 | 0.050918909 | - |
| MSA-C | Aβ42 | COMPASS-VI | 0.225866826 | 0.180496201 | 1.251366095 | 0.221943552 | - |
| MSA-C | Aβ42 | Total COMPASS | 0.053307768 | 0.149729732 | 0.356026605 | 0.723561514 | - |
| MSA-C | Aβ42 | RBDSQ | 0.069837466 | 0.161778649 | 0.431685306 | 0.66834734 | - |
| MSA-C | Aβ42 | HAMA | -0.279129452 | 0.189905792 | -1.469831165 | 0.151684494 | - |
| MSA-C | Aβ42 | HAMD | -0.276967372 | 0.188144323 | -1.472100602 | 0.151074217 | - |
| MSA-C | Aβ42 | MMSE | -0.003364409 | 0.142097495 | -0.023676766 | 0.981206598 | - |
| MSA-C | Aβ42 | MoCA | -0.069953345 | 0.138021456 | -0.506829494 | 0.614547745 | - |
| MSA-C | Aβ42 | AVLT-learning | 0.151688554 | 0.147924679 | 1.025444535 | 0.310887808 | - |
| MSA-C | Aβ42 | AVLT-recall | -0.047540465 | 0.16026067 | -0.296644618 | 0.768166016 | - |
| MSA-C | Aβ42 | AVLT-recognition | 0.066149422 | 0.163225824 | 0.405263212 | 0.687556697 | - |
| MSA-C | Aβ42 | ROCF-immediate | -0.202625968 | 0.152715615 | -1.326818923 | 0.191911196 | - |
| MSA-C | Aβ42 | ROCF-delay | 0.013137161 | 0.133859277 | 0.098141579 | 0.922396184 | - |
| MSA-C | Aβ42 | DST | 0.041005378 | 0.147062807 | 0.278829017 | 0.781682384 | - |
| MSA-C | Aβ42 | SCWT-time | -0.156916703 | 0.147406788 | -1.064514773 | 0.293479611 | - |
| MSA-C | Aβ42 | SCWT-number | -0.026718309 | 0.16982064 | -0.157332518 | 0.875774307 | - |
| MSA-C | Aβ42 | VFT | 0.209638 | 0.144988746 | 1.445891532 | 0.154986926 | - |
| MSA-C | Aβ42 | BNT | -0.062408443 | 0.159257523 | -0.391871239 | 0.69704498 | - |
| MSA-C | Aβ42 | TMT-A | -0.089640905 | 0.130312361 | -0.68789257 | 0.496054387 | - |
| MSA-C | Aβ42 | TMT-B | -0.08360594 | 0.166940612 | -0.500812467 | 0.619469457 | - |
| MSA-C | Aβ42 | ADL | 0.006685021 | 0.144686609 | 0.046203453 | 0.96335716 | - |
| MSA-C | Aβ42 | MBI-C | 0.087125695 | 0.156310706 | 0.55738789 | 0.580152595 | - |
| MSA-C | Aβ42 | NPI | 0.034228568 | 0.159924085 | 0.214030099 | 0.831584465 | - |
| MSA-C | Aβ42 | ZBI | -0.040073141 | 0.179529469 | -0.223212053 | 0.824566062 | - |

| Group | Plasma measures | Clinical scales | β | Standard error | t | P | P (FDR-corrected) |
| --- | --- | --- | --- | --- | --- | --- | --- |
| MSA-C | Aβ40 | UMSARS-I | 0.177077368 | 0.157391074 | 1.125078844 | 0.267099017 | - |
| MSA-C | Aβ40 | UMSARS-II | 0.152193739 | 0.149182426 | 1.020185439 | 0.313481256 | - |
| MSA-C | Aβ40 | UMSARS-III | 0.171962665 | 0.164559147 | 1.044990011 | 0.302806964 | - |
| MSA-C | Aβ40 | UMSARS-IV | 0.322157202 | 0.161291191 | 1.997363902 | 0.052796255 | - |
| MSA-C | Aβ40 | Total UMSARS | 0.188652087 | 0.153055032 | 1.232576832 | 0.224755023 | - |
| MSA-C | Aβ40 | ICARS | 0.087174422 | 0.220101895 | 0.396063931 | 0.694273071 | - |
| MSA-C | Aβ40 | SARA | 0.096919296 | 0.22450505 | 0.431702074 | 0.668397563 | - |
| MSA-C | Aβ40 | COMPASS-I | -0.103127689 | 0.146383375 | -0.70450411 | 0.484917243 | - |
| MSA-C | Aβ40 | COMPASS-II | -0.106777244 | 0.146145485 | -0.730622943 | 0.468971887 | - |
| MSA-C | Aβ40 | COMPASS-III | 0.157905731 | 0.155751807 | 1.013829203 | 0.31633517 | - |
| MSA-C | Aβ40 | COMPASS-IV | 0.278518219 | 0.155061526 | 1.796178755 | 0.079494055 | - |
| MSA-C | Aβ40 | COMPASS-V | 0.383221556 | 0.152683464 | 2.509908713 | 0.061592044 | - |
| MSA-C | Aβ40 | COMPASS-VI | 0.173887895 | 0.194431241 | 0.894341332 | 0.379343945 | - |
| MSA-C | Aβ40 | Total COMPASS | 0.031246705 | 0.149856127 | 0.20851136 | 0.835814276 | - |
| MSA-C | Aβ40 | RBDSQ | 0.159594714 | 0.158289088 | 1.008248362 | 0.319550105 | - |
| MSA-C | Aβ40 | HAMA | -0.156625928 | 0.215064756 | -0.728273341 | 0.471912096 | - |
| MSA-C | Aβ40 | HAMD | -0.215390241 | 0.211396016 | -1.018894515 | 0.316139382 | - |
| MSA-C | Aβ40 | MMSE | -0.110113603 | 0.14264204 | -0.771957572 | 0.443846834 | - |
| MSA-C | Aβ40 | MoCA | -0.127534066 | 0.13856176 | -0.920413148 | 0.361866224 | - |
| MSA-C | Aβ40 | AVLT-learning | 0.10667168 | 0.144076432 | 0.740382578 | 0.463091617 | - |
| MSA-C | Aβ40 | AVLT-recall | -0.029865157 | 0.156970792 | -0.190259326 | 0.850001304 | - |
| MSA-C | Aβ40 | AVLT-recognition | 0.080820909 | 0.158936121 | 0.508511902 | 0.614034115 | - |
| MSA-C | Aβ40 | ROCF-immediate | -0.049418751 | 0.148983078 | -0.331707142 | 0.741800109 | - |
| MSA-C | Aβ40 | ROCF-delay | 0.054030769 | 0.128094719 | 0.421803256 | 0.675823769 | - |
| MSA-C | Aβ40 | DST | 0.05311858 | 0.14503085 | 0.366257112 | 0.715928504 | - |
| MSA-C | Aβ40 | SCWT-time | -0.071005999 | 0.145333424 | -0.488573084 | 0.627811956 | - |
| MSA-C | Aβ40 | SCWT-number | -0.063007502 | 0.165352899 | -0.381048667 | 0.705183575 | - |
| MSA-C | Aβ40 | VFT | 0.147602466 | 0.146273011 | 1.009088862 | 0.318210722 | - |
| MSA-C | Aβ40 | BNT | -0.084737774 | 0.155559549 | -0.544728847 | 0.588690452 | - |
| MSA-C | Aβ40 | TMT-A | -0.059460551 | 0.126739881 | -0.469154225 | 0.641868861 | - |
| MSA-C | Aβ40 | TMT-B | 0.1880832 | 0.15391581 | 1.221987525 | 0.229440572 | - |
| MSA-C | Aβ40 | ADL | 0.154110428 | 0.143086264 | 1.077045577 | 0.287329823 | - |
| MSA-C | Aβ40 | MBI-C | 0.014885557 | 0.155508801 | 0.09572164 | 0.924186372 | - |
| MSA-C | Aβ40 | NPI | -0.040839493 | 0.15982722 | -0.255522766 | 0.799596936 | - |
| MSA-C | Aβ40 | ZBI | 0.130240608 | 0.173414978 | 0.751034367 | 0.457257981 | - |

| Group | Plasma measures | Clinical scales | β | Standard error | t | P | P (FDR-corrected) |
| --- | --- | --- | --- | --- | --- | --- | --- |
| MSA-C | Aβ42/40 | UMSARS-I | -0.178600911 | 0.154065891 | -1.15925018 | 0.253061311 | - |
| MSA-C | Aβ42/40 | UMSARS-II | -0.101464498 | 0.149309431 | -0.679558533 | 0.500512414 | - |
| MSA-C | Aβ42/40 | UMSARS-III | -0.013125922 | 0.169298674 | -0.077531155 | 0.938618768 | - |
| MSA-C | Aβ42/40 | UMSARS-IV | -0.001417267 | 0.162903492 | -0.008700042 | 0.99310281 | - |
| MSA-C | Aβ42/40 | Total UMSARS | -0.153051257 | 0.150830617 | -1.014722744 | 0.316188971 | - |
| MSA-C | Aβ42/40 | ICARS | -0.235651975 | 0.193162074 | -1.219970203 | 0.232652397 | - |
| MSA-C | Aβ42/40 | SARA | -0.138411154 | 0.199100555 | -0.695182163 | 0.492670585 | - |
| MSA-C | Aβ42/40 | COMPASS-I | 0.099569667 | 0.143569486 | 0.693529453 | 0.491706878 | - |
| MSA-C | Aβ42/40 | COMPASS-II | 0.175342246 | 0.141696771 | 1.237447008 | 0.222636087 | - |
| MSA-C | Aβ42/40 | COMPASS-III | 0.068867412 | 0.154188254 | 0.446644996 | 0.65737284 | - |
| MSA-C | Aβ42/40 | COMPASS-IV | -0.03626443 | 0.157558056 | -0.23016551 | 0.819054645 | - |
| MSA-C | Aβ42/40 | COMPASS-V | -0.155972363 | 0.158540407 | -0.98380196 | 0.330716291 | - |
| MSA-C | Aβ42/40 | COMPASS-VI | 0.03936113 | 0.249176312 | 0.157964976 | 0.875704862 | - |
| MSA-C | Aβ42/40 | Total COMPASS | 0.074741116 | 0.146581355 | 0.509895105 | 0.612732276 | - |
| MSA-C | Aβ42/40 | RBDSQ | -0.158242874 | 0.152149046 | -1.040051698 | 0.304721004 | - |
| MSA-C | Aβ42/40 | HAMA | -0.117173864 | 0.185148848 | -0.632863045 | 0.531464136 | - |
| MSA-C | Aβ42/40 | HAMD | -0.101090148 | 0.183736338 | -0.550191374 | 0.586131009 | - |
| MSA-C | Aβ42/40 | MMSE | 0.255415538 | 0.145743697 | 1.752498002 | 0.085943764 | - |
| MSA-C | Aβ42/40 | MoCA | 0.154880638 | 0.144630678 | 1.070869888 | 0.289474426 | - |
| MSA-C | Aβ42/40 | AVLT-learning | 0.002720442 | 0.153798604 | 0.01768834 | 0.985969304 | - |
| MSA-C | Aβ42/40 | AVLT-recall | -0.077287566 | 0.163590955 | -0.472444007 | 0.638999003 | - |
| MSA-C | Aβ42/40 | AVLT-recognition | -0.157744176 | 0.170562391 | -0.924847353 | 0.360881514 | - |
| MSA-C | Aβ42/40 | ROCF-immediate | -0.145510198 | 0.148389221 | -0.980598165 | 0.332542112 | - |
| MSA-C | Aβ42/40 | ROCF-delay | -0.123467963 | 0.13009046 | -0.94909314 | 0.349271048 | - |
| MSA-C | Aβ42/40 | DST | 0.036380552 | 0.151562691 | 0.240036326 | 0.8114169 | - |
| MSA-C | Aβ42/40 | SCWT-time | -0.080773048 | 0.145255742 | -0.556074733 | 0.581257273 | - |
| MSA-C | Aβ42/40 | SCWT-number | -0.027575032 | 0.165651833 | -0.166463788 | 0.868630599 | - |
| MSA-C | Aβ42/40 | VFT | 0.092761745 | 0.150243241 | 0.617410434 | 0.540008502 | - |
| MSA-C | Aβ42/40 | BNT | 0.074992015 | 0.154232638 | 0.486226628 | 0.629219478 | - |
| MSA-C | Aβ42/40 | TMT-A | -0.028093334 | 0.135127289 | -0.207902741 | 0.836509965 | - |
| MSA-C | Aβ42/40 | TMT-B | -0.369128795 | 0.138187124 | -2.671224235 | 0.011167314 | 0.39085599 |
| MSA-C | Aβ42/40 | ADL | -0.183749276 | 0.15795697 | -1.163286917 | 0.250983099 | - |
| MSA-C | Aβ42/40 | MBI-C | 0.107922976 | 0.156887092 | 0.687902202 | 0.495208647 | - |
| MSA-C | Aβ42/40 | NPI | 0.173831044 | 0.170139783 | 1.02169546 | 0.312915989 | - |
| MSA-C | Aβ42/40 | ZBI | -0.118478011 | 0.165603375 | -0.715432347 | 0.478715041 | - |

| Group | Plasma measures | Clinical scales | β | Standard error | t | P | P (FDR-corrected) |
| --- | --- | --- | --- | --- | --- | --- | --- |
| MSA-C | P-tau181 | UMSARS-I | -0.050315942 | 0.146767041 | -0.342828622 | 0.733478929 | - |
| MSA-C | P-tau181 | UMSARS-II | -0.065771254 | 0.139663296 | -0.470927263 | 0.640129502 | - |
| MSA-C | P-tau181 | UMSARS-III | 0.109934077 | 0.171406757 | 0.641363733 | 0.525239733 | - |
| MSA-C | P-tau181 | UMSARS-IV | 0.209279462 | 0.163585529 | 1.279327472 | 0.208341037 | - |
| MSA-C | P-tau181 | Total UMSARS | -0.068473873 | 0.142956578 | -0.478983712 | 0.634495029 | - |
| MSA-C | P-tau181 | ICARS | 0.375357558 | 0.178943651 | 2.097629925 | 0.054264315 | - |
| MSA-C | P-tau181 | SARA | 0.426188098 | 0.180058847 | 2.366937831 | 0.06231333 | - |
| MSA-C | P-tau181 | COMPASS-I | -0.099365261 | 0.142520059 | -0.697201939 | 0.489428976 | - |
| MSA-C | P-tau181 | COMPASS-II | -0.273210828 | 0.136954758 | -1.994898407 | 0.052416611 | - |
| MSA-C | P-tau181 | COMPASS-III | -0.076315293 | 0.152982826 | -0.498848761 | 0.620428431 | - |
| MSA-C | P-tau181 | COMPASS-IV | 0.034779685 | 0.156421993 | 0.222345238 | 0.825097863 | - |
| MSA-C | P-tau181 | COMPASS-V | 0.105770792 | 0.158332769 | 0.668028431 | 0.507685464 | - |
| MSA-C | P-tau181 | COMPASS-VI | 0.322548152 | 0.185694727 | 1.73698067 | 0.094225819 | - |
| MSA-C | P-tau181 | Total COMPASS | -0.082788481 | 0.145410671 | -0.569342544 | 0.572086032 | - |
| MSA-C | P-tau181 | RBDSQ | -0.007609118 | 0.158102235 | -0.048127832 | 0.96185999 | - |
| MSA-C | P-tau181 | HAMA | 0.369000214 | 0.21017133 | 1.755711463 | 0.08901556 | - |
| MSA-C | P-tau181 | HAMD | 0.099140216 | 0.217623728 | 0.455557934 | 0.651882923 | - |
| MSA-C | P-tau181 | MMSE | -0.114061177 | 0.13549955 | -0.8417827 | 0.403999315 | - |
| MSA-C | P-tau181 | MoCA | -0.088668645 | 0.132302248 | -0.670197568 | 0.505878303 | - |
| MSA-C | P-tau181 | AVLT-learning | -0.090055161 | 0.1492271 | -0.603477256 | 0.549361577 | - |
| MSA-C | P-tau181 | AVLT-recall | -0.083153454 | 0.156052768 | -0.532854717 | 0.596877401 | - |
| MSA-C | P-tau181 | AVLT-recognition | -0.096645598 | 0.181549821 | -0.532336508 | 0.597592085 | - |
| MSA-C | P-tau181 | ROCF-immediate | -0.148229266 | 0.142791307 | -1.038083264 | 0.30531493 | - |
| MSA-C | P-tau181 | ROCF-delay | -0.032056408 | 0.134440785 | -0.238442585 | 0.81296914 | - |
| MSA-C | P-tau181 | DST | 0.037388744 | 0.147693412 | 0.253151064 | 0.801330547 | - |
| MSA-C | P-tau181 | SCWT-time | 0.260570798 | 0.139940592 | 1.862010115 | 0.069959653 | - |
| MSA-C | P-tau181 | SCWT-number | 0.129157963 | 0.164518496 | 0.785066514 | 0.437039918 | - |
| MSA-C | P-tau181 | VFT | -0.063377842 | 0.147419583 | -0.429914675 | 0.669265041 | - |
| MSA-C | P-tau181 | BNT | -0.070078905 | 0.148260867 | -0.472672975 | 0.638782801 | - |
| MSA-C | P-tau181 | TMT-A | 0.137918181 | 0.132585298 | 1.040222276 | 0.305369459 | - |
| MSA-C | P-tau181 | TMT-B | 0.343298465 | 0.134780778 | 2.54708773 | 0.051515335 | - |
| MSA-C | P-tau181 | ADL | -0.013613912 | 0.134615345 | -0.10113195 | 0.919905163 | - |
| MSA-C | P-tau181 | MBI-C | -0.164342169 | 0.156232492 | -1.051907746 | 0.298717537 | - |
| MSA-C | P-tau181 | NPI | -0.160071364 | 0.167471504 | -0.955812541 | 0.34476902 | - |
| MSA-C | P-tau181 | ZBI | -0.012385805 | 0.174650368 | -0.070917717 | 0.943835168 | - |

| Group | Plasma measures | Clinical scales | β | Standard error | t | P | P (FDR-corrected) |
| --- | --- | --- | --- | --- | --- | --- | --- |
| MSA-P | NfL | UMSARS-I | 1.098110104 | 0.229643199 | 4.781809822 | 0.002008068 | 0.02342746 |
| MSA-P | NfL | UMSARS-II | 1.194714114 | 0.172121859 | 6.941094639 | 0.000119482 | 0.00418187 |
| MSA-P | NfL | UMSARS-III | 0.764363942 | 0.338741289 | 2.256482946 | 0.064860788 | - |
| MSA-P | NfL | UMSARS-IV | 1.233922638 | 0.355713563 | 3.468865867 | 0.013322232 | 0.093255624 |
| MSA-P | NfL | Total UMSARS | 1.145494092 | 0.181188271 | 6.32212055 | 0.000395662 | 0.006924085 |
| MSA-P | NfL | ICARS | 0.701771296 | 0.387303188 | 1.811942989 | 0.11289313 | - |
| MSA-P | NfL | SARA | 0.737555041 | 0.393591929 | 1.873907937 | 0.103084398 | - |
| MSA-P | NfL | COMPASS-I | 0.661422958 | 0.754643783 | 0.876470427 | 0.409842203 | - |
| MSA-P | NfL | COMPASS-II | 1.140108351 | 0.59668177 | 1.910747753 | 0.097650096 | - |
| MSA-P | NfL | COMPASS-III | -0.262678016 | 0.713680943 | -0.368060853 | 0.723705622 | - |
| MSA-P | NfL | COMPASS-IV | -0.020740518 | 0.740588769 | -0.028005444 | 0.978439507 | - |
| MSA-P | NfL | COMPASS-V | 1.319376045 | 0.350016109 | 3.769472348 | 0.006987751 | 0.061142821 |
| MSA-P | NfL | COMPASS-VI | 1.413069823 | 0.409230677 | 3.452990947 | 0.0259834 | 0.151569833 |
| MSA-P | NfL | Total COMPASS | 0.807973343 | 0.737095834 | 1.096157793 | 0.309282917 | - |
| MSA-P | NfL | RBDSQ | 0.335087677 | 0.81761982 | 0.409833114 | 0.696159094 | - |
| MSA-P | NfL | HAMA | 0.837984373 | 0.373288217 | 2.244872286 | 0.055005675 | - |
| MSA-P | NfL | HAMD | 0.646670284 | 0.451617919 | 1.431896868 | 0.190061241 | - |
| MSA-P | NfL | MMSE | -0.146070577 | 0.216563378 | -0.674493435 | 0.513923461 | - |
| MSA-P | NfL | MoCA | -0.224961342 | 0.166567058 | -1.350575229 | 0.203961243 | - |
| MSA-P | NfL | AVLT-learning | 0.332753255 | 0.349159218 | 0.953012946 | 0.368486804 | - |
| MSA-P | NfL | AVLT-recall | 0.096060391 | 0.362999675 | 0.264629413 | 0.797985151 | - |
| MSA-P | NfL | AVLT-recognition | 0.18662518 | 0.456955058 | 0.408410361 | 0.695176742 | - |
| MSA-P | NfL | ROCF-immediate | -0.230038524 | 0.281181504 | -0.818113995 | 0.436979661 | - |
| MSA-P | NfL | ROCF-delay | -0.321991361 | 0.351287655 | -0.916603121 | 0.394698995 | - |
| MSA-P | NfL | DST | 0.149598102 | 0.383467027 | 0.390119858 | 0.706629452 | - |
| MSA-P | NfL | SCWT-time | 0.807527194 | 0.449030138 | 1.798380833 | 0.115157012 | - |
| MSA-P | NfL | SCWT-number | 0.34900673 | 0.537556574 | 0.649246511 | 0.536891547 | - |
| MSA-P | NfL | VFT | -0.408221492 | 0.412220079 | -0.990299874 | 0.355016064 | - |
| MSA-P | NfL | BNT | -0.112691783 | 0.256955993 | -0.438564525 | 0.672579455 | - |
| MSA-P | NfL | TMT-A | 0.923877135 | 0.73862895 | 1.250800059 | 0.279176639 | - |
| MSA-P | NfL | TMT-B | 0.573770937 | 0.918164693 | 0.624910695 | 0.559437501 | - |
| MSA-P | NfL | ADL | 0.739649626 | 0.311787273 | 2.372289346 | 0.045085024 | 0.22542512 |
| MSA-P | NfL | MBI-C | 0.528062611 | 0.700556244 | 0.753776182 | 0.475568764 | - |
| MSA-P | NfL | NPI | -0.286697735 | 0.775555356 | -0.369667662 | 0.7225601 | - |
| MSA-P | NfL | ZBI | 0.657249912 | 0.394209095 | 1.667262175 | 0.134020239 | - |

| Group | Plasma measures | Clinical scales | β | Standard error | t | P | P (FDR-corrected) |
| --- | --- | --- | --- | --- | --- | --- | --- |
| MSA-P | GFAP | UMSARS-I | 0.19618313 | 0.420587385 | 0.466450343 | 0.655061431 | - |
| MSA-P | GFAP | UMSARS-II | 0.326081358 | 0.39670599 | 0.821972358 | 0.434904421 | - |
| MSA-P | GFAP | UMSARS-III | -0.110652814 | 0.426684286 | -0.259331823 | 0.804047616 | - |
| MSA-P | GFAP | UMSARS-IV | 0.132851054 | 0.390687581 | 0.34004422 | 0.74541167 | - |
| MSA-P | GFAP | Total UMSARS | 0.261851166 | 0.410817542 | 0.637390421 | 0.544146528 | - |
| MSA-P | GFAP | ICARS | 0.240850582 | 0.439410107 | 0.548122536 | 0.600642273 | - |
| MSA-P | GFAP | SARA | 0.360629869 | 0.440454821 | 0.818766991 | 0.439899404 | - |
| MSA-P | GFAP | COMPASS-I | 0.51430384 | 0.398003373 | 1.292209751 | 0.237305119 | - |
| MSA-P | GFAP | COMPASS-II | 0.828714468 | 0.264693568 | 3.130844752 | 0.016590107 | 0.580653745 |
| MSA-P | GFAP | COMPASS-III | -0.099161021 | 0.39971902 | -0.248076814 | 0.811195068 | - |
| MSA-P | GFAP | COMPASS-IV | 0.17392336 | 0.407388779 | 0.426922314 | 0.682259585 | - |
| MSA-P | GFAP | COMPASS-V | 0.311448578 | 0.318394964 | 0.978183118 | 0.360571312 | - |
| MSA-P | GFAP | COMPASS-VI | -0.100967254 | 0.528166823 | -0.19116546 | 0.857707097 | - |
| MSA-P | GFAP | Total COMPASS | 0.543588528 | 0.394213946 | 1.378917548 | 0.210368502 | - |
| MSA-P | GFAP | RBDSQ | 0.029986225 | 0.53008598 | 0.056568606 | 0.956725605 | - |
| MSA-P | GFAP | HAMA | 0.238433315 | 0.419371107 | 0.568549695 | 0.585263645 | - |
| MSA-P | GFAP | HAMD | 0.468520067 | 0.423074427 | 1.107417603 | 0.300296166 | - |
| MSA-P | GFAP | MMSE | 0.034187233 | 0.231348129 | 0.147773975 | 0.885195056 | - |
| MSA-P | GFAP | MoCA | -0.120707582 | 0.184911422 | -0.652785969 | 0.527301518 | - |
| MSA-P | GFAP | AVLT-learning | 0.443314123 | 0.246248295 | 1.800272861 | 0.10950733 | - |
| MSA-P | GFAP | AVLT-recall | 0.243218354 | 0.275741163 | 0.882053123 | 0.403471414 | - |
| MSA-P | GFAP | AVLT-recognition | 0.163391591 | 0.37008634 | 0.441495871 | 0.672169996 | - |
| MSA-P | GFAP | ROCF-immediate | 0.229910874 | 0.217181016 | 1.058614043 | 0.320689666 | - |
| MSA-P | GFAP | ROCF-delay | 0.278220719 | 0.287780137 | 0.966782217 | 0.370989225 | - |
| MSA-P | GFAP | DST | -0.133053973 | 0.303044136 | -0.439058068 | 0.672236493 | - |
| MSA-P | GFAP | SCWT-time | 0.066636311 | 0.439867624 | 0.151491738 | 0.88386136 | - |
| MSA-P | GFAP | SCWT-number | 0.45766596 | 0.414514807 | 1.104100391 | 0.306051421 | - |
| MSA-P | GFAP | VFT | -0.124056887 | 0.357629693 | -0.34688643 | 0.73887118 | - |
| MSA-P | GFAP | BNT | -0.091479317 | 0.203448324 | -0.449643993 | 0.664900123 | - |
| MSA-P | GFAP | TMT-A | 0.795578157 | 0.686248948 | 1.159314209 | 0.31081858 | - |
| MSA-P | GFAP | TMT-B | 0.47039278 | 0.844537803 | 0.556982503 | 0.601549738 | - |
| MSA-P | GFAP | ADL | 0.305674614 | 0.303736272 | 1.006381662 | 0.343697743 | - |
| MSA-P | GFAP | MBI-C | 0.603538104 | 0.335689533 | 1.797905634 | 0.115237121 | - |
| MSA-P | GFAP | NPI | 0.506844933 | 0.392010366 | 1.292937579 | 0.237067065 | - |
| MSA-P | GFAP | ZBI | -0.060150425 | 0.364097716 | -0.165204072 | 0.872881666 | - |

| Group | Plasma measures | Clinical scales | β | Standard error | t | P | P (FDR-corrected) |
| --- | --- | --- | --- | --- | --- | --- | --- |
| MSA-P | Aβ42 | UMSARS-I | 0.026633326 | 0.361383665 | 0.0736982 | 0.943312285 | - |
| MSA-P | Aβ42 | UMSARS-II | 0.093443769 | 0.344285785 | 0.271413381 | 0.792945324 | - |
| MSA-P | Aβ42 | UMSARS-III | 0.071516126 | 0.35946017 | 0.198954244 | 0.848869175 | - |
| MSA-P | Aβ42 | UMSARS-IV | -0.198007534 | 0.3333674 | -0.593961898 | 0.574233029 | - |
| MSA-P | Aβ42 | Total UMSARS | 0.055346415 | 0.357099935 | 0.154988588 | 0.881204676 | - |
| MSA-P | Aβ42 | ICARS | 0.216221389 | 0.328230635 | 0.658748347 | 0.531120433 | - |
| MSA-P | Aβ42 | SARA | 0.162373613 | 0.342078654 | 0.474667482 | 0.649475428 | - |
| MSA-P | Aβ42 | COMPASS-I | 0.118231779 | 0.386457147 | 0.305937618 | 0.768550334 | - |
| MSA-P | Aβ42 | COMPASS-II | 0.273052435 | 0.345078356 | 0.791276621 | 0.454753508 | - |
| MSA-P | Aβ42 | COMPASS-III | 0.147291765 | 0.348190908 | 0.423020135 | 0.68497313 | - |
| MSA-P | Aβ42 | COMPASS-IV | 0.198369546 | 0.354598212 | 0.559420603 | 0.593314034 | - |
| MSA-P | Aβ42 | COMPASS-V | -0.073910501 | 0.296835566 | -0.24899476 | 0.810512672 | - |
| MSA-P | Aβ42 | COMPASS-VI | 0.271375516 | 0.469155659 | 0.578433854 | 0.593977102 | - |
| MSA-P | Aβ42 | Total COMPASS | 0.172382017 | 0.384966555 | 0.447784397 | 0.667838425 | - |
| MSA-P | Aβ42 | RBDSQ | -0.194441939 | 0.454239629 | -0.428060272 | 0.683546267 | - |
| MSA-P | Aβ42 | HAMA | 0.399205788 | 0.320924586 | 1.243923979 | 0.248738912 | - |
| MSA-P | Aβ42 | HAMD | 0.473238313 | 0.332675181 | 1.422523649 | 0.192673371 | - |
| MSA-P | Aβ42 | MMSE | -0.130890039 | 0.172923477 | -0.756924631 | 0.464999266 | - |
| MSA-P | Aβ42 | MoCA | -0.086536243 | 0.141966572 | -0.609553655 | 0.554539309 | - |
| MSA-P | Aβ42 | AVLT-learning | 0.079992496 | 0.237677055 | 0.336559606 | 0.745107362 | - |
| MSA-P | Aβ42 | AVLT-recall | -0.131308012 | 0.232250873 | -0.565371446 | 0.587322385 | - |
| MSA-P | Aβ42 | AVLT-recognition | -0.43559811 | 0.258692506 | -1.683845108 | 0.13608549 | - |
| MSA-P | Aβ42 | ROCF-immediate | 0.182477148 | 0.17887257 | 1.020151654 | 0.337512454 | - |
| MSA-P | Aβ42 | ROCF-delay | 0.445124352 | 0.176884063 | 2.51647516 | 0.08550327 | - |
| MSA-P | Aβ42 | DST | -0.070346949 | 0.250237074 | -0.281121209 | 0.785751386 | - |
| MSA-P | Aβ42 | SCWT-time | 0.058248095 | 0.359403156 | 0.162068958 | 0.875830518 | - |
| MSA-P | Aβ42 | SCWT-number | 0.474186657 | 0.320355833 | 1.480187366 | 0.182366398 | - |
| MSA-P | Aβ42 | VFT | -0.392346935 | 0.266625133 | -1.4715302 | 0.184621823 | - |
| MSA-P | Aβ42 | BNT | 0.029946736 | 0.168588874 | 0.177631743 | 0.863427468 | - |
| MSA-P | Aβ42 | TMT-A | 0.082639787 | 0.460700051 | 0.179378724 | 0.866360251 | - |
| MSA-P | Aβ42 | TMT-B | 0.093584716 | 0.505468506 | 0.185144505 | 0.860391617 | - |
| MSA-P | Aβ42 | ADL | 0.345518914 | 0.23443964 | 1.473807558 | 0.178759676 | - |
| MSA-P | Aβ42 | MBI-C | -0.012601426 | 0.356437442 | -0.035353824 | 0.972784639 | - |
| MSA-P | Aβ42 | NPI | 0.204003633 | 0.375378574 | 0.543461048 | 0.603680419 | - |
| MSA-P | Aβ42 | ZBI | 0.177247725 | 0.297703194 | 0.595384023 | 0.568042905 | - |

| Group | Plasma measures | Clinical scales | β | Standard error | t | P | P (FDR-corrected) |
| --- | --- | --- | --- | --- | --- | --- | --- |
| MSA-P | Aβ40 | UMSARS-I | 0.090132866 | 0.367667764 | 0.245147589 | 0.81337381 | - |
| MSA-P | Aβ40 | UMSARS-II | 0.048527257 | 0.351110557 | 0.13821076 | 0.893489417 | - |
| MSA-P | Aβ40 | UMSARS-III | -0.122824989 | 0.35452891 | -0.346445623 | 0.740834028 | - |
| MSA-P | Aβ40 | UMSARS-IV | -0.17387321 | 0.317910194 | -0.546925559 | 0.604159486 | - |
| MSA-P | Aβ40 | Total UMSARS | 0.076228635 | 0.364211746 | 0.209297576 | 0.840176601 | - |
| MSA-P | Aβ40 | ICARS | 0.602349334 | 0.38076195 | 1.581957794 | 0.157673539 | - |
| MSA-P | Aβ40 | SARA | 0.521487974 | 0.410998123 | 1.268832982 | 0.245061592 | - |
| MSA-P | Aβ40 | COMPASS-I | -0.097173062 | 0.41808232 | -0.232425666 | 0.822856636 | - |
| MSA-P | Aβ40 | COMPASS-II | 0.248787226 | 0.37701758 | 0.659882294 | 0.530434292 | - |
| MSA-P | Aβ40 | COMPASS-III | -0.158279634 | 0.375669779 | -0.421326502 | 0.686152427 | - |
| MSA-P | Aβ40 | COMPASS-IV | 0.011678694 | 0.390977498 | 0.029870501 | 0.977004133 | - |
| MSA-P | Aβ40 | COMPASS-V | -0.260526853 | 0.306200823 | -0.850836554 | 0.423006572 | - |
| MSA-P | Aβ40 | COMPASS-VI | -0.273203904 | 0.81473344 | -0.33532919 | 0.754225901 | - |
| MSA-P | Aβ40 | Total COMPASS | -0.158046999 | 0.416955068 | -0.379050434 | 0.715886274 | - |
| MSA-P | Aβ40 | RBDSQ | 0.062298059 | 0.43714222 | 0.142512107 | 0.891340733 | - |
| MSA-P | Aβ40 | HAMA | 0.689902282 | 0.420730572 | 1.63977217 | 0.139683344 | - |
| MSA-P | Aβ40 | HAMD | 0.755125504 | 0.442206523 | 1.707630859 | 0.126087564 | - |
| MSA-P | Aβ40 | MMSE | -0.3460055 | 0.172616148 | -2.004479324 | 0.070262518 | - |
| MSA-P | Aβ40 | MoCA | -0.230961991 | 0.14863396 | -1.553897853 | 0.148490461 | - |
| MSA-P | Aβ40 | AVLT-learning | -0.072801619 | 0.323576356 | -0.22499054 | 0.827627251 | - |
| MSA-P | Aβ40 | AVLT-recall | -0.402066372 | 0.28802792 | -1.395928465 | 0.200257028 | - |
| MSA-P | Aβ40 | AVLT-recognition | -0.468519107 | 0.366511862 | -1.278319081 | 0.241888147 | - |
| MSA-P | Aβ40 | ROCF-immediate | 0.023526433 | 0.257739003 | 0.091280066 | 0.929514275 | - |
| MSA-P | Aβ40 | ROCF-delay | 0.460136197 | 0.280357802 | 1.641246272 | 0.151855152 | - |
| MSA-P | Aβ40 | DST | -0.398295647 | 0.31059853 | -1.282348781 | 0.235627984 | - |
| MSA-P | Aβ40 | SCWT-time | 0.047618732 | 0.477644986 | 0.099694822 | 0.923381707 | - |
| MSA-P | Aβ40 | SCWT-number | 0.524712726 | 0.44509965 | 1.178865735 | 0.276964865 | - |
| MSA-P | Aβ40 | VFT | -0.293546247 | 0.309576977 | -0.948217308 | 0.37459626 | - |
| MSA-P | Aβ40 | BNT | 0.235300824 | 0.213441997 | 1.102411088 | 0.302339679 | - |
| MSA-P | Aβ40 | TMT-A | 0.156074502 | 0.472982146 | 0.32997969 | 0.757973433 | - |
| MSA-P | Aβ40 | TMT-B | 0.2703005 | 0.444287103 | 0.608391508 | 0.569503357 | - |
| MSA-P | Aβ40 | ADL | 0.307881872 | 0.341582223 | 0.901340442 | 0.393732934 | - |
| MSA-P | Aβ40 | MBI-C | 0.213996199 | 0.375961115 | 0.569197692 | 0.587013077 | - |
| MSA-P | Aβ40 | NPI | 0.243896282 | 0.403009063 | 0.605188082 | 0.564150382 | - |
| MSA-P | Aβ40 | ZBI | 0.221979253 | 0.349434546 | 0.63525274 | 0.543002043 | - |

| Group | Plasma measures | Clinical scales | β | Standard error | t | P | P (FDR-corrected) |
| --- | --- | --- | --- | --- | --- | --- | --- |
| MSA-P | Aβ42/40 | UMSARS-I | -0.095857694 | 0.414906892 | -0.23103423 | 0.823895764 | - |
| MSA-P | Aβ42/40 | UMSARS-II | 0.077698831 | 0.351261267 | 0.22119954 | 0.830478503 | - |
| MSA-P | Aβ42/40 | UMSARS-III | 0.373053013 | 0.427029046 | 0.873601026 | 0.415926335 | - |
| MSA-P | Aβ42/40 | UMSARS-IV | -0.142675112 | 0.377381516 | -0.378065978 | 0.718395796 | - |
| MSA-P | Aβ42/40 | Total UMSARS | -0.020366151 | 0.412022676 | -0.049429685 | 0.961957691 | - |
| MSA-P | Aβ42/40 | ICARS | -0.417784117 | 0.472037978 | -0.885064629 | 0.426119145 | - |
| MSA-P | Aβ42/40 | SARA | -0.577392844 | 0.465637705 | -1.240004489 | 0.282745465 | - |
| MSA-P | Aβ42/40 | COMPASS-I | 0.261633115 | 0.36637103 | 0.714120643 | 0.498266742 | - |
| MSA-P | Aβ42/40 | COMPASS-II | 0.155448382 | 0.34639122 | 0.448765364 | 0.667163946 | - |
| MSA-P | Aβ42/40 | COMPASS-III | 0.364307993 | 0.315192727 | 1.15582614 | 0.285673869 | - |
| MSA-P | Aβ42/40 | COMPASS-IV | 0.304238218 | 0.334317278 | 0.910028404 | 0.393063435 | - |
| MSA-P | Aβ42/40 | COMPASS-V | 0.126223531 | 0.286888296 | 0.439974489 | 0.673219949 | - |
| MSA-P | Aβ42/40 | COMPASS-VI | 0.296661807 | 0.351926912 | 0.842964256 | 0.446704677 | - |
| MSA-P | Aβ42/40 | Total COMPASS | 0.400938041 | 0.349409613 | 1.147472841 | 0.288886971 | - |
| MSA-P | Aβ42/40 | RBDSQ | -0.417077911 | 0.484247546 | -0.861290707 | 0.422158038 | - |
| MSA-P | Aβ42/40 | HAMA | 0.109968113 | 0.289586519 | 0.379741823 | 0.714019557 | - |
| MSA-P | Aβ42/40 | HAMD | 0.156342399 | 0.305382986 | 0.511955171 | 0.622511996 | - |
| MSA-P | Aβ42/40 | MMSE | 0.108786745 | 0.168652116 | 0.64503635 | 0.532126267 | - |
| MSA-P | Aβ42/40 | MoCA | 0.080973671 | 0.137673526 | 0.588157165 | 0.568305752 | - |
| MSA-P | Aβ42/40 | AVLT-learning | 0.155097089 | 0.214295345 | 0.723753888 | 0.489836335 | - |
| MSA-P | Aβ42/40 | AVLT-recall | 0.049561925 | 0.218179111 | 0.227161641 | 0.825995564 | - |
| MSA-P | Aβ42/40 | AVLT-recognition | -0.347284956 | 0.267822435 | -1.296698524 | 0.235840244 | - |
| MSA-P | Aβ42/40 | ROCF-immediate | 0.235283352 | 0.154793614 | 1.519981006 | 0.1670035 | - |
| MSA-P | Aβ42/40 | ROCF-delay | 0.377785772 | 0.187697782 | 2.012734343 | 0.090809854 | - |
| MSA-P | Aβ42/40 | DST | 0.10489753 | 0.229418843 | 0.457231538 | 0.659665198 | - |
| MSA-P | Aβ42/40 | SCWT-time | 0.015697974 | 0.350184521 | 0.044827722 | 0.965496627 | - |
| MSA-P | Aβ42/40 | SCWT-number | 0.41804138 | 0.320185255 | 1.305623458 | 0.232950916 | - |
| MSA-P | Aβ42/40 | VFT | -0.25299458 | 0.27489325 | -0.920337551 | 0.38801249 | - |
| MSA-P | Aβ42/40 | BNT | -0.074933741 | 0.153844638 | -0.487074116 | 0.639270714 | - |
| MSA-P | Aβ42/40 | TMT-A | -0.089403095 | 0.529628968 | -0.168803257 | 0.874143536 | - |
| MSA-P | Aβ42/40 | TMT-B | -0.237938253 | 0.445616513 | -0.533952952 | 0.616250229 | - |
| MSA-P | Aβ42/40 | ADL | 0.27592954 | 0.223983997 | 1.231916316 | 0.252959264 | - |
| MSA-P | Aβ42/40 | MBI-C | -0.243142608 | 0.335354428 | -0.725031751 | 0.491951636 | - |
| MSA-P | Aβ42/40 | NPI | 0.035482082 | 0.373567769 | 0.094981646 | 0.926991187 | - |
| MSA-P | Aβ42/40 | ZBI | 0.069612273 | 0.301383516 | 0.230975716 | 0.823131285 | - |

| Group | Plasma measures | Clinical scales | β | Standard error | t | P | P (FDR-corrected) |
| --- | --- | --- | --- | --- | --- | --- | --- |
| MSA-P | P-tau181 | UMSARS-I | 0.535749213 | 0.255598359 | 2.096058891 | 0.074302328 | - |
| MSA-P | P-tau181 | UMSARS-II | 0.447768791 | 0.255790389 | 1.750530164 | 0.118137458 | - |
| MSA-P | P-tau181 | UMSARS-III | -0.284186217 | 0.308137325 | -0.922271319 | 0.391963651 | - |
| MSA-P | P-tau181 | UMSARS-IV | 0.00343821 | 0.290477665 | 0.011836401 | 0.990939889 | - |
| MSA-P | P-tau181 | Total UMSARS | 0.491714614 | 0.263748934 | 1.864328348 | 0.104544975 | - |
| MSA-P | P-tau181 | ICARS | 0.420075144 | 0.254401505 | 1.651229007 | 0.142675076 | - |
| MSA-P | P-tau181 | SARA | 0.540420354 | 0.230681592 | 2.342711218 | 0.051638472 | - |
| MSA-P | P-tau181 | COMPASS-I | 0.212551094 | 0.351751555 | 0.604264832 | 0.564730261 | - |
| MSA-P | P-tau181 | COMPASS-II | 0.426531885 | 0.292574584 | 1.45785693 | 0.188235527 | - |
| MSA-P | P-tau181 | COMPASS-III | -0.096067655 | 0.325010219 | -0.295583491 | 0.776123682 | - |
| MSA-P | P-tau181 | COMPASS-IV | 0.265043548 | 0.320869909 | 0.826015592 | 0.436040061 | - |
| MSA-P | P-tau181 | COMPASS-V | 0.337915635 | 0.245253875 | 1.377819759 | 0.210691985 | - |
| MSA-P | P-tau181 | COMPASS-VI | 0.270682628 | 0.640612267 | 0.422537378 | 0.694357083 | - |
| MSA-P | P-tau181 | Total COMPASS | 0.29703784 | 0.344272166 | 0.862799464 | 0.41682552 | - |
| MSA-P | P-tau181 | RBDSQ | 0.525131544 | 0.326372021 | 1.608996818 | 0.158742096 | - |
| MSA-P | P-tau181 | HAMA | 0.08291541 | 0.307028783 | 0.27005745 | 0.79395183 | - |
| MSA-P | P-tau181 | HAMD | 0.017067222 | 0.327539276 | 0.052107405 | 0.95972074 | - |
| MSA-P | P-tau181 | MMSE | -0.05599154 | 0.174198013 | -0.321424677 | 0.753913406 | - |
| MSA-P | P-tau181 | MoCA | -0.005210458 | 0.142418715 | -0.036585484 | 0.971471015 | - |
| MSA-P | P-tau181 | AVLT-learning | 0.423665105 | 0.17446876 | 2.428314986 | 0.061310694 | - |
| MSA-P | P-tau181 | AVLT-recall | 0.393685985 | 0.1799994 | 2.187151649 | 0.060187676 | - |
| MSA-P | P-tau181 | AVLT-recognition | 0.248793422 | 0.280093115 | 0.888252542 | 0.403892446 | - |
| MSA-P | P-tau181 | ROCF-immediate | -0.07979411 | 0.180487404 | -0.442103484 | 0.670122049 | - |
| MSA-P | P-tau181 | ROCF-delay | -0.253232264 | 0.240207249 | -1.05422407 | 0.332370736 | - |
| MSA-P | P-tau181 | DST | 0.006861416 | 0.241575448 | 0.028402789 | 0.978036661 | - |
| MSA-P | P-tau181 | SCWT-time | 0.024728172 | 0.346818451 | 0.071300048 | 0.945153285 | - |
| MSA-P | P-tau181 | SCWT-number | 0.061772619 | 0.352923283 | 0.175031294 | 0.866010211 | - |
| MSA-P | P-tau181 | VFT | 0.165544125 | 0.276676659 | 0.59833065 | 0.568465811 | - |
| MSA-P | P-tau181 | BNT | -0.025966511 | 0.162022777 | -0.160264573 | 0.876645468 | - |
| MSA-P | P-tau181 | TMT-A | -0.291193211 | 0.346415189 | -0.840590192 | 0.447889018 | - |
| MSA-P | P-tau181 | TMT-B | -0.66568917 | 0.273082036 | -2.437689345 | 0.058820823 | - |
| MSA-P | P-tau181 | ADL | 0.074256605 | 0.252607967 | 0.293959868 | 0.776271009 | - |
| MSA-P | P-tau181 | MBI-C | 0.342234559 | 0.30425292 | 1.124835742 | 0.297744314 | - |
| MSA-P | P-tau181 | NPI | -0.051666442 | 0.354877752 | -0.145589408 | 0.888349195 | - |
| MSA-P | P-tau181 | ZBI | 0.172219814 | 0.301755068 | 0.570727163 | 0.583855489 | - |

Note: Results of multiple linear regressions were shown after accounting for age and sex.

Abbreviations: Αβ, amyloid-β; ADL, Activity of Daily Living Scale; AVLT: Auditory Verbal Learning Test; BNT: Boston Naming Test; COMPASS, Composite Autonomic Symptom Score; DST: Digit Span Test; FDR, false discovery rate; GFAP, glial fibrillary acidic protein; HAMA, Hamilton Anxiety Scale; HAMD, Hamilton Depression Scale; ICARS, International Cooperative Ataxia Rating Scale; MBI-C, Mild Behavioral Impairment Checklist; MMSE, Mini-Mental State Examination; MoCA, Montreal Cognitive Assessment; MSA, multiple system atrophy; MSA-C, multiple system atrophy-cerebellar type; MSA-P, multiple system atrophy-parkinsonian type; NfL, neurofilament light; NPI, Neuropsychiatric Inventory; p-tau, phosphorylated tau; RBDSQ, Rapid eye movement sleep behavior disorder screening questionnaire; ROCF: Rey-Osterreich Complex Figure; SARA, Scale for the Assessment and Rating of Ataxia; SCWT: Stroops Color Word Test; TMT: Trails Making Test; UMSARS, Unified Multiple System Atrophy Rating Scale; VFT, Verbal Fluency Test; ZBI, Zarit Caregiver Burden Interview.

**Supplementary Table 5: Associations of plasma measures with MRI indices in MSA patients**

| Plasma measure | Volume | β | Standard error | t | P | P value (FDR corrected) |
| --- | --- | --- | --- | --- | --- | --- |
| NfL | Medulla | 0.136203006 | 0.134979474 | 1.009064583 | 0.317802104 | - |
| NfL | Pons | 0.069684819 | 0.127211811 | 0.547785767 | 0.586276258 | - |
| NfL | Midbrain | 0.128545176 | 0.119237561 | 1.078059423 | 0.286180918 | - |
| NfL | Whole brainstem | 0.106742005 | 0.120845152 | 0.883295718 | 0.381303903 | - |

| Plasma measure | Volume | β | Standard error | t | P | P value (FDR corrected) |
| --- | --- | --- | --- | --- | --- | --- |
| GFAP | Medulla | 0.082501998 | 0.133708916 | 0.617026903 | 0.540016722 | - |
| GFAP | Pons | -0.375489312 | 0.113822075 | -3.298914657 | 0.001793165 | 0.00717266 |
| GFAP | Midbrain | -0.162447323 | 0.116487431 | -1.394548077 | 0.169317662 | - |
| GFAP | Whole brainstem | -0.301095282 | 0.112064749 | -2.686797461 | 0.009768057 | 0.019536114 |

| Plasma measure | Volume | β | Standard error | t | P | P value (FDR corrected) |
| --- | --- | --- | --- | --- | --- | --- |
| NfL | Left lateral ventricle | 0.14140723 | 0.131457697 | 1.075686198 | 0.287230916 | - |
| NfL | Left cerebellum white matter | -0.257497148 | 0.130273266 | -1.976592403 | 0.053616532 | - |
| NfL | Left cerebellum cortex | 0.03261511 | 0.126214346 | 0.258410485 | 0.797150904 | - |
| NfL | Left thalamus | -0.274617488 | 0.122582543 | -2.24026587 | 0.029548223 | 0.098494077 |
| NfL | Left caudate | -0.061536692 | 0.138084066 | -0.445646582 | 0.65777628 | - |
| NfL | Left putamen | -0.297075025 | 0.13174545 | -2.254916766 | 0.028548834 | 0.098494077 |
| NfL | Left pallidum | -0.367868616 | 0.122333419 | -3.007098285 | 0.00411968 | 0.0411968 |
| NfL | Left hippocampus | -0.247313168 | 0.104752884 | -2.3609199 | 0.022169183 | 0.098494077 |
| NfL | Left amygdala | -0.110120578 | 0.126743383 | -0.868846768 | 0.389082511 | - |
| NfL | Left accumbens | 0.182647892 | 0.141759964 | 1.288430713 | 0.203526387 | - |
| NfL | Right lateral ventricle | 0.14853422 | 0.126422414 | 1.174904158 | 0.245598098 | - |
| NfL | Right cerebellum white matter | -0.204600042 | 0.132326768 | -1.546172739 | 0.128369434 | - |
| NfL | Right cerebellum cortex | 0.107838114 | 0.129211549 | 0.834585725 | 0.407920954 | - |
| NfL | Right thalamus | -0.356136607 | 0.107575157 | -3.310584113 | 0.001733055 | 0.0346611 |
| NfL | Right caudate | -0.007349576 | 0.139123586 | -0.052827678 | 0.958079712 | - |
| NfL | Right putamen | -0.206915754 | 0.138401635 | -1.495038362 | 0.141189607 | - |
| NfL | Right pallidum | -0.325316306 | 0.128925885 | -2.523281552 | 0.014851745 | 0.098494077 |
| NfL | Right hippocampus | -0.219596736 | 0.107057565 | -2.051202422 | 0.045504892 | 0.130013977 |
| NfL | Right amygdala | -0.010737388 | 0.132779402 | -0.080866369 | 0.935871053 | - |
| NfL | Right accumbens | -0.022886634 | 0.144111289 | -0.158812222 | 0.874456557 | - |

| Plasma measure | Volume | β | Standard error | t | P | P value (FDR corrected) |
| --- | --- | --- | --- | --- | --- | --- |
| GFAP | Left lateral ventricle | 0.173479851 | 0.128573149 | 1.349269677 | 0.183328815 | - |
| GFAP | Left cerebellum white matter | -0.292133954 | 0.12658273 | -2.307850002 | 0.0251835 | 0.083945 |
| GFAP | Left cerebellum cortex | -0.253012954 | 0.119065347 | -2.124992368 | 0.038552331 | 0.08893416 |
| GFAP | Left thalamus | -0.107000844 | 0.12567115 | -0.851435227 | 0.398587188 | - |
| GFAP | Left caudate | -0.136061093 | 0.134830593 | -1.009126267 | 0.317772817 | - |
| GFAP | Left putamen | -0.275045097 | 0.130446108 | -2.108496007 | 0.040020372 | 0.08893416 |
| GFAP | Left pallidum | -0.389864078 | 0.118677401 | -3.285074294 | 0.001867016 | 0.02487707 |
| GFAP | Left hippocampus | -0.244446113 | 0.103069359 | -2.371666178 | 0.021599683 | 0.083945 |
| GFAP | Left amygdala | 0.022043675 | 0.125663131 | 0.175418793 | 0.861459058 | - |
| GFAP | Left accumbens | 0.265358182 | 0.13678877 | 1.939912035 | 0.05804142 | - |
| GFAP | Right lateral ventricle | 0.131601821 | 0.124773307 | 1.054727359 | 0.296620131 | - |
| GFAP | Right cerebellum white matter | -0.387288847 | 0.121568892 | -3.185756157 | 0.002487707 | 0.02487707 |
| GFAP | Right cerebellum cortex | -0.269251446 | 0.122284624 | -2.201842206 | 0.032317905 | 0.08893416 |
| GFAP | Right thalamus | -0.257051984 | 0.111131476 | -2.31304392 | 0.024873138 | 0.083945 |
| GFAP | Right caudate | -0.011542156 | 0.136944392 | -0.084283523 | 0.933167575 | - |
| GFAP | Right putamen | -0.232901492 | 0.135300082 | -1.721369919 | 0.091366399 | - |
| GFAP | Right pallidum | -0.345680619 | 0.125569622 | -2.752900053 | 0.008210851 | 0.054739007 |
| GFAP | Right hippocampus | -0.157633362 | 0.107441288 | -1.467158163 | 0.148595299 | - |
| GFAP | Right amygdala | 0.198631121 | 0.127659712 | 1.555942108 | 0.1260295 | - |
| GFAP | Right accumbens | 0.115020033 | 0.140960431 | 0.815973905 | 0.418385489 | - |

| Plasma measure | Volume | β | Standard error | t | P | P value (FDR corrected) |
| --- | --- | --- | --- | --- | --- | --- |
| NfL | Left bankssts | 0.005431012 | 0.122440505 | 0.044356334 | 0.964797043 | - |
| NfL | Left caudal anterior cingulate | 0.094512777 | 0.143855494 | 0.656998039 | 0.514195082 | - |
| NfL | Left caudal middle frontal | -0.21392538 | 0.132634509 | -1.612893814 | 0.113062297 | - |
| NfL | Left cuneus | -0.142316718 | 0.133468096 | -1.066297656 | 0.291410981 | - |
| NfL | Left entorhinal | -0.051351874 | 0.140458608 | -0.365601477 | 0.716204681 | - |
| NfL | Left fusiform | -0.121934715 | 0.117096 | -1.041322636 | 0.302734975 | - |
| NfL | Left inferior parietal | 0.017158325 | 0.113065195 | 0.151756027 | 0.879990044 | - |
| NfL | Left inferior temporal | -0.082576555 | 0.117947541 | -0.700112557 | 0.487101959 | - |
| NfL | Left isthmus cingulate | 0.135558125 | 0.121981376 | 1.111301823 | 0.271753814 | - |
| NfL | Left lateral occipital | -0.221039271 | 0.117250401 | -1.885189894 | 0.065221891 | - |
| NfL | Left lateral orbitofrontal | -0.60042041 | 0.093357457 | -6.431413491 | 4.65E-08 | 1.58E-06 |
| NfL | Left lingual | 0.017221816 | 0.135887681 | 0.126735664 | 0.899657912 | - |
| NfL | Left medial orbitofrontal | 0.616369623 | 0.088111553 | 6.995332613 | 6.10E-09 | 4.15E-07 |
| NfL | Left middle temporal | 0.006895138 | 0.126291401 | 0.054597048 | 0.956677065 | - |
| NfL | Left parahippocampal | 0.223945257 | 0.136971231 | 1.634980245 | 0.108334229 | - |
| NfL | Left paracentral | -0.075707029 | 0.127617645 | -0.593233239 | 0.555699155 | - |
| NfL | Left pars opercularis | -0.147572102 | 0.133009418 | -1.109486113 | 0.27252834 | - |
| NfL | Left pars orbitalis | -0.057357443 | 0.11998575 | -0.478035464 | 0.634708436 | - |
| NfL | Left pars triangularis | -0.158203047 | 0.136150645 | -1.161970605 | 0.250764093 | - |
| NfL | Left pericalcarine | -0.025050346 | 0.141075469 | -0.177566987 | 0.859780467 | - |
| NfL | Left postcentral | -0.140613972 | 0.113224201 | -1.241907386 | 0.220064732 | - |
| NfL | Left posterior cingulate | 0.104269005 | 0.140205257 | 0.743688271 | 0.460545664 | - |
| NfL | Left precentral | -0.199033146 | 0.098100934 | -2.028860874 | 0.047814211 | 0.325136635 |
| NfL | Left precuneus | -0.136313645 | 0.094314984 | -1.445302107 | 0.154611803 | - |
| NfL | Left rostral anterior cingulate | 0.049110262 | 0.118138289 | 0.415701485 | 0.67940721 | - |
| NfL | Left rostral middle frontal | -0.133246895 | 0.130870369 | -1.018159395 | 0.313503693 | - |
| NfL | Left superior frontal | -0.226483649 | 0.111041986 | -2.039621746 | 0.046689547 | 0.325136635 |
| NfL | Left superior parietal | -0.105008218 | 0.12780733 | -0.821613426 | 0.415197584 | - |
| NfL | Left superior temporal | -0.234978021 | 0.101415462 | -2.316984168 | 0.024639969 | 0.325136635 |
| NfL | Left supramarginal | -0.156726857 | 0.105967991 | -1.479001868 | 0.145412783 | - |
| NfL | Left frontal pole | 0.103620738 | 0.137378101 | 0.754274066 | 0.454222518 | - |
| NfL | Left temporal pole | 0.039398337 | 0.136894762 | 0.287800179 | 0.774689274 | - |
| NfL | Left transverse temporal | -0.268071118 | 0.125007563 | -2.144439198 | 0.036882452 | 0.325136635 |
| NfL | Left insula | -0.082860737 | 0.114834017 | -0.721569605 | 0.473920041 | - |
| NfL | Right bankssts | 0.050521009 | 0.124247423 | 0.406616154 | 0.686025231 | - |
| NfL | Right caudal anterior cingulate | -0.051313508 | 0.144136677 | -0.356005904 | 0.723333904 | - |
| NfL | Right caudal middle frontal | -0.249781016 | 0.132366848 | -1.887036067 | 0.064967867 | - |
| NfL | Right cuneus | -0.215544423 | 0.123221282 | -1.749246717 | 0.086384678 | - |
| NfL | Right entorhinal | -0.131355388 | 0.135283157 | -0.970966315 | 0.336238902 | - |
| NfL | Right fusiform | -0.017850485 | 0.124920807 | -0.142894412 | 0.886947874 | - |
| NfL | Right inferior parietal | -0.115008227 | 0.114606488 | -1.003505378 | 0.320449014 | - |
| NfL | Right inferior temporal | -0.025790612 | 0.108043257 | -0.238706349 | 0.812308975 | - |
| NfL | Right isthmus cingulate | 0.013059261 | 0.130520578 | 0.100055188 | 0.920700623 | - |
| NfL | Right lateral occipital | -0.230522039 | 0.120948951 | -1.905944923 | 0.062414169 | - |
| NfL | Right lateral orbitofrontal | -0.287482288 | 0.095964523 | -2.995714242 | 0.004251963 | 0.096377828 |
| NfL | Right lingual | -0.181617846 | 0.127910915 | -1.41987762 | 0.161848619 | - |
| NfL | Right medial orbitofrontal | 0.03704309 | 0.121935848 | 0.30379163 | 0.762547282 | - |
| NfL | Right middle temporal | 0.034622901 | 0.104090856 | 0.332621925 | 0.740810277 | - |
| NfL | Right parahippocampal | -0.005879364 | 0.142605307 | -0.041228225 | 0.967278146 | - |
| NfL | Right paracentral | -0.080775722 | 0.132642588 | -0.608972753 | 0.545299592 | - |
| NfL | Right pars opercularis | -0.127378999 | 0.139624898 | -0.912294301 | 0.365991697 | - |
| NfL | Right pars orbitalis | -0.271221362 | 0.127762646 | -2.122853356 | 0.038739986 | 0.325136635 |
| NfL | Right pars triangularis | -0.157874759 | 0.133873938 | -1.179279258 | 0.243868093 | - |
| NfL | Right pericalcarine | -0.213086785 | 0.129845787 | -1.641075851 | 0.107058097 | - |
| NfL | Right postcentral | -0.192986904 | 0.118273669 | -1.631697957 | 0.109026506 | - |
| NfL | Right posterior cingulate | 0.027807824 | 0.143057116 | 0.194382668 | 0.846663898 | - |
| NfL | Right precentral | 0.011173377 | 0.105687265 | 0.10572113 | 0.916226624 | - |
| NfL | Right precuneus | -0.048459708 | 0.102199007 | -0.474170049 | 0.6374429 | - |
| NfL | Right rostral anterior cingulate | 0.01792916 | 0.140507683 | 0.127602703 | 0.898975218 | - |
| NfL | Right rostral middle frontal | 0.022948153 | 0.125809567 | 0.182403881 | 0.856003335 | - |
| NfL | Right superior frontal | 0.011183704 | 0.107905231 | 0.103643765 | 0.917866665 | - |
| NfL | Right superior parietal | -0.00526135 | 0.111652668 | -0.047122472 | 0.962603334 | - |
| NfL | Right superior temporal | -0.084368992 | 0.122069171 | -0.691157245 | 0.492663347 | - |
| NfL | Right supramarginal | -0.150075754 | 0.10080581 | -1.48876096 | 0.142831036 | - |
| NfL | Right frontal pole | 0.331349575 | 0.134898992 | 2.456279101 | 0.017554903 | 0.298433351 |
| NfL | Right temporal pole | -0.170357938 | 0.132552566 | -1.285210417 | 0.204640271 | - |
| NfL | Right transverse temporal | -0.21463872 | 0.120596499 | -1.779808879 | 0.081184905 | - |
| NfL | Right insula | -0.220334326 | 0.104003087 | -2.118536412 | 0.039121149 | 0.325136635 |

| Plasma measure | Volume | β | Standard error | t | P | P value (FDR corrected) |
| --- | --- | --- | --- | --- | --- | --- |
| GFAP | Left bankssts | -0.040863616 | 0.120391579 | -0.339422541 | 0.735713029 | - |
| GFAP | Left caudal anterior cingulate | 0.071913551 | 0.141854119 | 0.506954268 | 0.614415504 | - |
| GFAP | Left caudal middle frontal | -0.103167816 | 0.133118844 | -0.775005348 | 0.441986401 | - |
| GFAP | Left cuneus | 0.130130575 | 0.131587909 | 0.988925016 | 0.327461525 | - |
| GFAP | Left entorhinal | -0.093686907 | 0.137813725 | -0.67980825 | 0.499761342 | - |
| GFAP | Left fusiform | -0.306679977 | 0.108136623 | -2.836041752 | 0.006577404 | 0.179002044 |
| GFAP | Left inferior parietal | -0.018188776 | 0.111294879 | -0.163428691 | 0.870839712 | - |
| GFAP | Left inferior temporal | -0.153126658 | 0.114645442 | -1.33565413 | 0.187710707 | - |
| GFAP | Left isthmus cingulate | 0.10912377 | 0.120566091 | 0.905095029 | 0.369755793 | - |
| GFAP | Left lateral occipital | 0.008174168 | 0.119444713 | 0.068434745 | 0.945712513 | - |
| GFAP | Left lateral orbitofrontal | -0.34772028 | 0.114078248 | -3.048085722 | 0.003674594 | 0.179002044 |
| GFAP | Left lingual | 0.064686334 | 0.133473297 | 0.484638766 | 0.630049017 | - |
| GFAP | Left medial orbitofrontal | 0.200472419 | 0.118667168 | 1.689367186 | 0.097376362 | - |
| GFAP | Left middle temporal | 0.013422272 | 0.124307781 | 0.107976118 | 0.91444677 | - |
| GFAP | Left parahippocampal | -0.138083882 | 0.137004179 | -1.007880804 | 0.318364497 | - |
| GFAP | Left paracentral | -0.13454072 | 0.124621292 | -1.079596572 | 0.285502256 | - |
| GFAP | Left pars opercularis | -0.264075379 | 0.127162996 | -2.07666843 | 0.042991271 | 0.319968671 |
| GFAP | Left pars orbitalis | -0.04531646 | 0.118207417 | -0.383363926 | 0.703075158 | - |
| GFAP | Left pars triangularis | -0.325214401 | 0.127797251 | -2.544768351 | 0.014068463 | 0.239163871 |
| GFAP | Left pericalcarine | 0.031722674 | 0.138843009 | 0.228478727 | 0.820205981 | - |
| GFAP | Left postcentral | 0.030142327 | 0.113081137 | 0.266554862 | 0.790908154 | - |
| GFAP | Left posterior cingulate | 0.092786764 | 0.138154524 | 0.671615819 | 0.504919686 | - |
| GFAP | Left precentral | -0.07903399 | 0.099841207 | -0.791596908 | 0.432335151 | - |
| GFAP | Left precuneus | -0.141999836 | 0.092608874 | -1.533328603 | 0.131498619 | - |
| GFAP | Left rostral anterior cingulate | -0.073078305 | 0.11603427 | -0.629799326 | 0.531693486 | - |
| GFAP | Left rostral middle frontal | -0.058878992 | 0.129888 | -0.453305866 | 0.652289738 | - |
| GFAP | Left superior frontal | -0.142896313 | 0.111954496 | -1.276378516 | 0.207718613 | - |
| GFAP | Left superior parietal | -0.084522164 | 0.12609195 | -0.670321652 | 0.505737192 | - |
| GFAP | Left superior temporal | -0.066170341 | 0.104636345 | -0.632383913 | 0.530017405 | - |
| GFAP | Left supramarginal | -0.187795167 | 0.10320767 | -1.819585382 | 0.074811844 | - |
| GFAP | Left frontal pole | 0.261665941 | 0.130867957 | 1.999465315 | 0.051006742 | - |
| GFAP | Left temporal pole | -0.089080761 | 0.134278191 | -0.663404534 | 0.510118772 | - |
| GFAP | Left transverse temporal | -0.055879598 | 0.128346093 | -0.435382151 | 0.665158798 | - |
| GFAP | Left insula | -0.098920537 | 0.112762735 | -0.877244927 | 0.384549314 | - |
| GFAP | Right bankssts | -0.098770602 | 0.121709642 | -0.811526518 | 0.420909945 | - |
| GFAP | Right caudal anterior cingulate | -0.231492133 | 0.138241211 | -1.674552268 | 0.10026682 | - |
| GFAP | Right caudal middle frontal | -0.128822404 | 0.133622918 | -0.964074174 | 0.339648506 | - |
| GFAP | Right cuneus | -0.004506924 | 0.124951178 | -0.03606948 | 0.971370578 | - |
| GFAP | Right entorhinal | -0.084258741 | 0.133890287 | -0.629311825 | 0.532009935 | - |
| GFAP | Right fusiform | -0.210245056 | 0.119346492 | -1.761635825 | 0.084244339 | - |
| GFAP | Right inferior parietal | -0.175429388 | 0.111212951 | -1.577418691 | 0.121005802 | - |
| GFAP | Right inferior temporal | -0.089786135 | 0.105655803 | -0.849798426 | 0.399488041 | - |
| GFAP | Right isthmus cingulate | -0.112817764 | 0.127500158 | -0.884844115 | 0.380476194 | - |
| GFAP | Right lateral occipital | 0.033766019 | 0.12321624 | 0.274038706 | 0.785183857 | - |
| GFAP | Right lateral orbitofrontal | -0.168135228 | 0.099799862 | -1.684724059 | 0.098274784 | - |
| GFAP | Right lingual | -0.081836251 | 0.127903622 | -0.639827469 | 0.525205826 | - |
| GFAP | Right medial orbitofrontal | -0.041006401 | 0.120001736 | -0.341715066 | 0.733997404 | - |
| GFAP | Right middle temporal | 0.015123736 | 0.102555825 | 0.147468327 | 0.883355439 | - |
| GFAP | Right parahippocampal | -0.283912925 | 0.134515493 | -2.110633642 | 0.039827425 | 0.319968671 |
| GFAP | Right paracentral | -0.065409934 | 0.130726992 | -0.500355234 | 0.619020249 | - |
| GFAP | Right pars opercularis | -0.265809389 | 0.133387213 | -1.99276515 | 0.051759638 | - |
| GFAP | Right pars orbitalis | -0.286734402 | 0.124894268 | -2.295817151 | 0.025915826 | 0.293712695 |
| GFAP | Right pars triangularis | -0.209307543 | 0.130282431 | -1.606567679 | 0.114446988 | - |
| GFAP | Right pericalcarine | -0.032659181 | 0.131133261 | -0.249053371 | 0.804339744 | - |
| GFAP | Right postcentral | -0.079454344 | 0.118956133 | -0.66792978 | 0.507249987 | - |
| GFAP | Right posterior cingulate | 0.200409995 | 0.137995093 | 1.452297982 | 0.152665579 | - |
| GFAP | Right precentral | -0.032537482 | 0.103946122 | -0.313022563 | 0.755565507 | - |
| GFAP | Right precuneus | -0.21261117 | 0.096240893 | -2.209156252 | 0.031773668 | 0.308658489 |
| GFAP | Right rostral anterior cingulate | -0.143407981 | 0.13684055 | -1.047993308 | 0.299681292 | - |
| GFAP | Right rostral middle frontal | 0.018362261 | 0.123858239 | 0.14825224 | 0.882739986 | - |
| GFAP | Right superior frontal | 0.102243192 | 0.105242372 | 0.971502169 | 0.335974764 | - |
| GFAP | Right superior parietal | -0.035093095 | 0.109798846 | -0.319612602 | 0.750593673 | - |
| GFAP | Right superior temporal | 0.006492434 | 0.120731452 | 0.053775827 | 0.957328062 | - |
| GFAP | Right supramarginal | -0.261348022 | 0.094431168 | -2.76760341 | 0.007897149 | 0.179002044 |
| GFAP | Right frontal pole | 0.168968961 | 0.138529446 | 1.219733182 | 0.228288387 | - |
| GFAP | Right temporal pole | -0.11630925 | 0.131595668 | -0.883837987 | 0.381013899 | - |
| GFAP | Right transverse temporal | -0.074733957 | 0.121958265 | -0.612783045 | 0.542797058 | - |
| GFAP | Right insula | -0.236139532 | 0.101523154 | -2.325967254 | 0.024115663 | 0.293712695 |

Abbreviations: FDR, false discovery rate; GFAP, glial fibrillary acidic protein; MRI, magnetic resonance imaging; MSA, multiple system atrophy; NfL, neurofilament light.

Note: Associations of plasma measures with MRI indices were analyzed using multiple linear regressions after adjusting for intracranial volumes.

**Supplementary Figure 1: Plasma NfL and GFAP in DAT PET-negative versus DAT PET-positive groups**


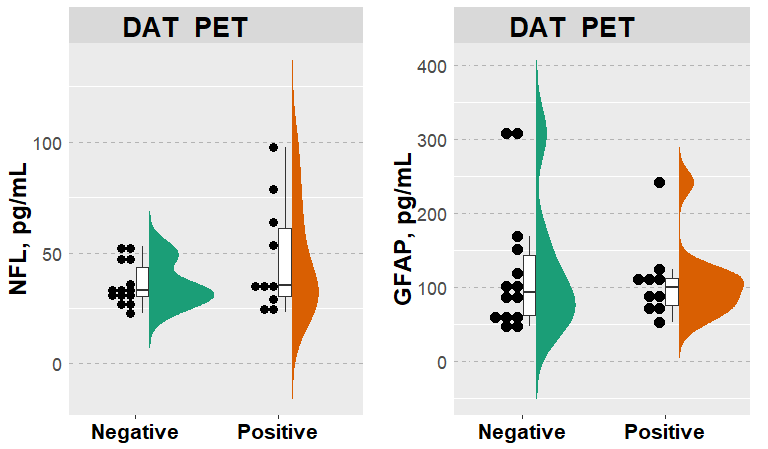


Note: Plasma levels (log-converted) were compared using analysis of covariance analyses after accounting for age and sex.

Abbreviations: DAT, dopamine transporter; GFAP, glial fibrillary acidic protein; NfL, neurofilament light; PET, positron emission tomography.

**Supplementary Table 6: Associations of plasma measures with PET imaging indices in MSA patients**

| Plasma measure | Brain regions (glucose metabolism) | β | Standard error | t | P | P value (FDR corrected) |
| --- | --- | --- | --- | --- | --- | --- |
| NfL | Left cerebral white matter | -0.294524176 | 0.219350215 | -1.342712047 | 0.193056074 | - |
| NfL | Left cerebellum white matter | -0.233623916 | 0.215911199 | -1.082037047 | 0.290954216 | - |
| NfL | Left cerebellum cortex | -0.020761295 | 0.218730291 | -0.094917329 | 0.925239731 | - |
| NfL | Left thalamus | -0.327345822 | 0.212047137 | -1.543740822 | 0.136914681 | - |
| NfL | Left caudate | -0.370437161 | 0.200451785 | -1.848011286 | 0.078089861 | - |
| NfL | Left putamen | -0.402601221 | 0.203391703 | -1.979437779 | 0.060414773 | - |
| NfL | Left pallidum | -0.198053268 | 0.224073402 | -0.88387674 | 0.386320298 | - |
| NfL | Brain stem | -0.39630212 | 0.211366544 | -1.874951979 | 0.074136832 | - |
| NfL | Left hippocampus | -0.378468373 | 0.21424244 | -1.766542485 | 0.091178173 | - |
| NfL | Left amygdala | -0.35517839 | 0.210957084 | -1.68365235 | 0.106386846 | - |
| NfL | Left accumbens | -0.388518865 | 0.205301775 | -1.892428185 | 0.071667008 | - |
| NfL | Right cerebral white matter | -0.272144986 | 0.220464979 | -1.234413679 | 0.230072295 | - |
| NfL | Right cerebellum white matter | -0.221680678 | 0.218047308 | -1.016663217 | 0.320364742 | - |
| NfL | Right cerebellum cortex | 0.020761295 | 0.218730291 | 0.094917329 | 0.925239731 | - |
| NfL | Right thalamus | -0.306612503 | 0.212687497 | -1.441610376 | 0.16349662 | - |
| NfL | Right caudate | -0.321298576 | 0.20572862 | -1.561759254 | 0.132615836 | - |
| NfL | Right putamen | -0.354802886 | 0.206599944 | -1.717342604 | 0.099963192 | - |
| NfL | Right pallidum | -0.267124873 | 0.221742636 | -1.204661757 | 0.241133868 | - |
| NfL | Right hippocampus | -0.351455914 | 0.21646601 | -1.623607853 | 0.118702658 | - |
| NfL | Right amygdala | -0.33407675 | 0.214542142 | -1.557161439 | 0.133702031 | - |
| NfL | Right accumbens | -0.469270383 | 0.204959989 | -2.289570692 | 0.032006075 | 0.272494367 |
| NfL | Pons | -0.359801897 | 0.187407157 | -1.919894108 | 0.067930968 | - |
| NfL | Left bankssts | -0.330011565 | 0.21538012 | -1.532228536 | 0.139721 | - |
| NfL | Left caudal anterior cingulate | -0.30899612 | 0.207986632 | -1.485653749 | 0.15156083 | - |
| NfL | Left caudal middle frontal | -0.281608156 | 0.212074034 | -1.327876643 | 0.197832484 | - |
| NfL | Left cuneus | -0.242308739 | 0.216907294 | -1.117107383 | 0.276000388 | - |
| NfL | Left entorhinal | -0.270561266 | 0.220497958 | -1.227046584 | 0.232774768 | - |
| NfL | Left fusiform | -0.316374632 | 0.216074091 | -1.464195132 | 0.157284591 | - |
| NfL | Left inferior parietal | -0.297696067 | 0.215408394 | -1.382007736 | 0.180842017 | - |
| NfL | Left inferior temporal | -0.310909773 | 0.214779195 | -1.447578628 | 0.161836098 | - |
| NfL | Left isthmus cingulate | -0.366980229 | 0.206291408 | -1.778940926 | 0.089071873 | - |
| NfL | Left lateral occipital | -0.345509598 | 0.214715633 | -1.60914971 | 0.121840697 | - |
| NfL | Left lateral orbitofrontal | -0.671202384 | 0.165572855 | -4.053818985 | 0.000528964 | 0.029476125 |
| NfL | Left lingual | -0.291822295 | 0.219332208 | -1.33050361 | 0.196980031 | - |
| NfL | Left medial orbitofrontal | -0.655469593 | 0.165266537 | -3.966136184 | 0.000655025 | 0.029476125 |
| NfL | Left middle temporal | -0.311337188 | 0.215052273 | -1.447727957 | 0.161794726 | - |
| NfL | Left parahippocampal | -0.277949376 | 0.213347336 | -1.302802186 | 0.206114942 | - |
| NfL | Left paracentral | -0.282476964 | 0.218347138 | -1.293705821 | 0.20918534 | - |
| NfL | Left pars opercularis | -0.355185496 | 0.206508461 | -1.719956147 | 0.099478982 | - |
| NfL | Left pars orbitalis | -0.548934037 | 0.190329613 | -2.884123116 | 0.008613378 | 0.19755054 |
| NfL | Left pars triangularis | -0.374120318 | 0.201762579 | -1.854260195 | 0.077156956 | - |
| NfL | Left pericalcarine | -0.170533717 | 0.21465965 | -0.794437694 | 0.435427322 | - |
| NfL | Left postcentral | -0.243846145 | 0.215501427 | -1.131529145 | 0.270016314 | - |
| NfL | Left posterior cingulate | -0.289364631 | 0.212490076 | -1.361779506 | 0.187050707 | - |
| NfL | Left precentral | -0.304947779 | 0.216274238 | -1.410005106 | 0.172520002 | - |
| NfL | Left precuneus | -0.309867466 | 0.214360275 | -1.445545193 | 0.162400317 | - |
| NfL | Left rostral anterior cingulate | -0.381101642 | 0.201163526 | -1.894486784 | 0.071380872 | - |
| NfL | Left rostral middle frontal | -0.386821357 | 0.203198867 | -1.903659026 | 0.070118106 | - |
| NfL | Left superior frontal | -0.369597547 | 0.207016658 | -1.785351722 | 0.087999306 | - |
| NfL | Left superior parietal | -0.265282579 | 0.217387397 | -1.2203218 | 0.235262581 | - |
| NfL | Left superior temporal | -0.343464378 | 0.212390831 | -1.61713373 | 0.12009935 | - |
| NfL | Left supramarginal | -0.241422148 | 0.216330375 | -1.115988208 | 0.276468786 | - |
| NfL | Left frontal pole | -0.524688653 | 0.182452369 | -2.875756867 | 0.008780024 | 0.19755054 |
| NfL | Left temporal pole | -0.334000027 | 0.215884235 | -1.547125604 | 0.136098479 | - |
| NfL | Left transverse temporal | -0.347034404 | 0.1990698 | -1.743280016 | 0.095245717 | - |
| NfL | Left insula | -0.363928953 | 0.206911521 | -1.758862679 | 0.092504209 | - |
| NfL | Right bankssts | -0.337279996 | 0.213119328 | -1.58258755 | 0.127786484 | - |
| NfL | Right caudal anterior cingulate | -0.33851946 | 0.21296731 | -1.589537195 | 0.126207988 | - |
| NfL | Right caudal middle frontal | -0.244923031 | 0.213389688 | -1.147773511 | 0.263390564 | - |
| NfL | Right cuneus | -0.22208522 | 0.220293983 | -1.008131119 | 0.324351674 | - |
| NfL | Right entorhinal | -0.335062694 | 0.216746165 | -1.545875996 | 0.136399339 | - |
| NfL | Right fusiform | -0.301929575 | 0.216804886 | -1.392632709 | 0.177647013 | - |
| NfL | Right inferior parietal | -0.282449667 | 0.218694899 | -1.291523799 | 0.209927106 | - |
| NfL | Right inferior temporal | -0.300587442 | 0.21793598 | -1.379246525 | 0.181679759 | - |
| NfL | Right isthmus cingulate | -0.27652676 | 0.215154377 | -1.285248127 | 0.212071831 | - |
| NfL | Right lateral occipital | -0.263190212 | 0.222042973 | -1.18531205 | 0.248540004 | - |
| NfL | Right lateral orbitofrontal | -0.401301641 | 0.205239784 | -1.955281925 | 0.063370464 | - |
| NfL | Right lingual | -0.24737437 | 0.216954431 | -1.140213493 | 0.266459081 | - |
| NfL | Right medial orbitofrontal | -0.393134305 | 0.203401028 | -1.932803929 | 0.066234829 | - |
| NfL | Right middle temporal | -0.2792559 | 0.216712996 | -1.288597851 | 0.210924958 | - |
| NfL | Right parahippocampal | -0.310220062 | 0.213374206 | -1.45387799 | 0.160098245 | - |
| NfL | Right paracentral | -0.266022977 | 0.22046694 | -1.206634322 | 0.240388292 | - |
| NfL | Right pars opercularis | -0.38559455 | 0.203834422 | -1.891704776 | 0.071767796 | - |
| NfL | Right pars orbitalis | -0.390914505 | 0.209832542 | -1.862983219 | 0.07587093 | - |
| NfL | Right pars triangularis | -0.370858137 | 0.206420766 | -1.796612542 | 0.086142244 | - |
| NfL | Right pericalcarine | -0.233063444 | 0.215473634 | -1.081633236 | 0.291129732 | - |
| NfL | Right postcentral | -0.253605624 | 0.215500889 | -1.176819385 | 0.251843812 | - |
| NfL | Right posterior cingulate | -0.286644395 | 0.211486904 | -1.355376572 | 0.18905068 | - |
| NfL | Right precentral | -0.33039133 | 0.215195163 | -1.535310205 | 0.138965189 | - |
| NfL | Right precuneus | -0.325034492 | 0.214891576 | -1.512551111 | 0.144627165 | - |
| NfL | Right rostral anterior cingulate | -0.340922444 | 0.199589571 | -1.708117525 | 0.101688411 | - |
| NfL | Right rostral middle frontal | -0.368216311 | 0.208887298 | -1.762751085 | 0.091830761 | - |
| NfL | Right superior frontal | -0.369418945 | 0.208425557 | -1.772426333 | 0.090173319 | - |
| NfL | Right superior parietal | -0.310389405 | 0.21720174 | -1.429037378 | 0.167039672 | - |
| NfL | Right superior temporal | -0.311536783 | 0.21243561 | -1.466499816 | 0.156661571 | - |
| NfL | Right supramarginal | -0.256664855 | 0.218298144 | -1.175753722 | 0.252260677 | - |
| NfL | Right frontal pole | -0.395968319 | 0.200606254 | -1.9738583 | 0.061086416 | - |
| NfL | Right temporal pole | -0.326403316 | 0.217872138 | -1.498141607 | 0.148308693 | - |
| NfL | Right transverse temporal | -0.364145106 | 0.200718783 | -1.814205425 | 0.083308694 | - |
| NfL | Right insula | -0.353180547 | 0.209129826 | -1.688810027 | 0.105381303 | - |

| Plasma measure | Brain regions (glucose metabolism) | β | Standard error | t | P | P value (FDR corrected) |
| --- | --- | --- | --- | --- | --- | --- |
| GFAP | Left cerebral white matter | 0.395969218 | 0.204185878 | 1.939258593 | 0.065400859 | - |
| GFAP | Left cerebellum white matter | 0.503354973 | 0.185815268 | 2.708899962 | 0.012819847 | 0.070509191 |
| GFAP | Left cerebellum cortex | -0.037737646 | 0.211707574 | -0.17825364 | 0.860155012 | - |
| GFAP | Left thalamus | 0.389405131 | 0.199603919 | 1.950889207 | 0.063921476 | - |
| GFAP | Left caudate | 0.443482832 | 0.185985587 | 2.384501069 | 0.026152208 | 0.070509191 |
| GFAP | Left putamen | 0.382406419 | 0.197627799 | 1.934982939 | 0.06595225 | - |
| GFAP | Left pallidum | 0.310649354 | 0.210643645 | 1.474762529 | 0.154444359 | - |
| GFAP | Brain stem | 0.324748817 | 0.209278277 | 1.551755975 | 0.134988426 | - |
| GFAP | Left hippocampus | 0.418367936 | 0.202963075 | 2.061300731 | 0.051286962 | - |
| GFAP | Left amygdala | 0.441181014 | 0.19561269 | 2.255380331 | 0.034393248 | 0.070509191 |
| GFAP | Left accumbens | 0.315400066 | 0.203566347 | 1.549372333 | 0.135558926 | - |
| GFAP | Right cerebral white matter | 0.419309008 | 0.201856776 | 2.077260011 | 0.04965828 | 0.079805853 |
| GFAP | Right cerebellum white matter | 0.446099444 | 0.193999505 | 2.299487535 | 0.031342625 | 0.070509191 |
| GFAP | Right cerebellum cortex | 0.037737646 | 0.211707574 | 0.17825364 | 0.860155012 | - |
| GFAP | Right thalamus | 0.384189909 | 0.19929848 | 1.927711184 | 0.066899429 | - |
| GFAP | Right caudate | 0.446319364 | 0.18718172 | 2.384417478 | 0.026156897 | 0.070509191 |
| GFAP | Right putamen | 0.394068779 | 0.195794415 | 2.012666087 | 0.056548756 | - |
| GFAP | Right pallidum | 0.295546212 | 0.212560756 | 1.390408166 | 0.178312204 | - |
| GFAP | Right hippocampus | 0.415705697 | 0.203353382 | 2.044252689 | 0.053079398 | - |
| GFAP | Right amygdala | 0.423080761 | 0.199464139 | 2.121086844 | 0.045421623 | 0.077131058 |
| GFAP | Right accumbens | 0.349700727 | 0.207902479 | 1.682042123 | 0.106702437 | - |
| GFAP | Pons | 0.398006742 | 0.176788913 | 2.25131053 | 0.034687982 | 0.070509191 |
| GFAP | Left bankssts | 0.418717761 | 0.200438674 | 2.08900684 | 0.048489269 | 0.079805853 |
| GFAP | Left caudal anterior cingulate | 0.399444279 | 0.193350909 | 2.065903295 | 0.050812422 | - |
| GFAP | Left caudal middle frontal | 0.458635967 | 0.189727477 | 2.417340777 | 0.024368946 | 0.070509191 |
| GFAP | Left cuneus | 0.385455811 | 0.199677231 | 1.930394411 | 0.066548541 | - |
| GFAP | Left entorhinal | 0.398282301 | 0.203726726 | 1.954983075 | 0.063407818 | - |
| GFAP | Left fusiform | 0.496013568 | 0.192007421 | 2.583304155 | 0.016960651 | 0.070509191 |
| GFAP | Left inferior parietal | 0.496596075 | 0.189953351 | 2.614305423 | 0.015835213 | 0.070509191 |
| GFAP | Left inferior temporal | 0.453576741 | 0.195010855 | 2.325905097 | 0.029636329 | 0.070509191 |
| GFAP | Left isthmus cingulate | 0.284905242 | 0.20484068 | 1.390862605 | 0.178176155 | - |
| GFAP | Left lateral occipital | 0.377768622 | 0.204539575 | 1.846921904 | 0.078253497 | - |
| GFAP | Left lateral orbitofrontal | 0.264109702 | 0.204308676 | 1.292699395 | 0.209527216 | - |
| GFAP | Left lingual | 0.296814741 | 0.211516729 | 1.403268399 | 0.174493953 | - |
| GFAP | Left medial orbitofrontal | 0.284686877 | 0.200609022 | 1.419113029 | 0.169879688 | - |
| GFAP | Left middle temporal | 0.461195932 | 0.194509215 | 2.371074975 | 0.026915401 | 0.070509191 |
| GFAP | Left parahippocampal | 0.260401045 | 0.207114092 | 1.257283088 | 0.221835098 | - |
| GFAP | Left paracentral | 0.512550053 | 0.190182738 | 2.695039828 | 0.013224947 | 0.070509191 |
| GFAP | Left pars opercularis | 0.401376902 | 0.19505731 | 2.05773833 | 0.051656976 | - |
| GFAP | Left pars orbitalis | 0.30378611 | 0.206449656 | 1.471477919 | 0.155322679 | - |
| GFAP | Left pars triangularis | 0.426557162 | 0.189397251 | 2.252182437 | 0.034624645 | 0.070509191 |
| GFAP | Left pericalcarine | 0.43640792 | 0.189195116 | 2.306655318 | 0.030870966 | 0.070509191 |
| GFAP | Left postcentral | 0.522809194 | 0.183472517 | 2.849523202 | 0.009322745 | 0.070509191 |
| GFAP | Left posterior cingulate | 0.496306173 | 0.186321805 | 2.663704199 | 0.014185812 | 0.070509191 |
| GFAP | Left precentral | 0.502172189 | 0.190698534 | 2.633330093 | 0.015179704 | 0.070509191 |
| GFAP | Left precuneus | 0.465806443 | 0.193188976 | 2.411144007 | 0.024696581 | 0.070509191 |
| GFAP | Left rostral anterior cingulate | 0.482439707 | 0.183195936 | 2.633462927 | 0.015175219 | 0.070509191 |
| GFAP | Left rostral middle frontal | 0.455042931 | 0.188910686 | 2.408772846 | 0.024823024 | 0.070509191 |
| GFAP | Left superior frontal | 0.417729017 | 0.195143204 | 2.140628055 | 0.043639762 | 0.075530357 |
| GFAP | Left superior parietal | 0.482207144 | 0.1916972 | 2.515462634 | 0.019690106 | 0.070509191 |
| GFAP | Left superior temporal | 0.407510642 | 0.199458222 | 2.043087711 | 0.053203902 | - |
| GFAP | Left supramarginal | 0.538105141 | 0.182236376 | 2.95278666 | 0.007355366 | 0.070509191 |
| GFAP | Left frontal pole | 0.343494516 | 0.19388082 | 1.77167868 | 0.090300474 | - |
| GFAP | Left temporal pole | 0.394455078 | 0.203443461 | 1.938892876 | 0.065447863 | - |
| GFAP | Left transverse temporal | 0.334907131 | 0.192869583 | 1.736443483 | 0.096470277 | - |
| GFAP | Left insula | 0.470927855 | 0.188981288 | 2.491928483 | 0.020729123 | 0.070509191 |
| GFAP | Right bankssts | 0.395356544 | 0.200844152 | 1.968474257 | 0.061740793 | - |
| GFAP | Right caudal anterior cingulate | 0.366806018 | 0.20323 | 1.804881258 | 0.084800257 | - |
| GFAP | Right caudal middle frontal | 0.510608422 | 0.182779527 | 2.793575574 | 0.01058886 | 0.070509191 |
| GFAP | Right cuneus | 0.53412598 | 0.186131454 | 2.869616978 | 0.008904276 | 0.070509191 |
| GFAP | Right entorhinal | 0.436663567 | 0.200436249 | 2.178565853 | 0.040359559 | 0.07485516 |
| GFAP | Right fusiform | 0.502493744 | 0.191020112 | 2.630580298 | 0.015272847 | 0.070509191 |
| GFAP | Right inferior parietal | 0.541907325 | 0.186826809 | 2.900586529 | 0.008294225 | 0.070509191 |
| GFAP | Right inferior temporal | 0.518519417 | 0.190188345 | 2.726346961 | 0.012326577 | 0.070509191 |
| GFAP | Right isthmus cingulate | 0.466931896 | 0.1917309 | 2.43535025 | 0.023439463 | 0.070509191 |
| GFAP | Right lateral occipital | 0.439488996 | 0.201019639 | 2.186298806 | 0.039718993 | 0.07485516 |
| GFAP | Right lateral orbitofrontal | 0.421906338 | 0.195643325 | 2.156507705 | 0.042238514 | 0.07485516 |
| GFAP | Right lingual | 0.392520226 | 0.199364233 | 1.968859808 | 0.061693727 | - |
| GFAP | Right medial orbitofrontal | 0.457628262 | 0.189385758 | 2.416381607 | 0.024419394 | 0.070509191 |
| GFAP | Right middle temporal | 0.539867051 | 0.184712911 | 2.922735867 | 0.007882639 | 0.070509191 |
| GFAP | Right parahippocampal | 0.34445167 | 0.203482802 | 1.692780257 | 0.104612767 | - |
| GFAP | Right paracentral | 0.541806842 | 0.187763028 | 2.885588533 | 0.008584501 | 0.070509191 |
| GFAP | Right pars opercularis | 0.41669946 | 0.193413543 | 2.154448199 | 0.042417924 | 0.07485516 |
| GFAP | Right pars orbitalis | 0.453065506 | 0.196147965 | 2.309814973 | 0.030665127 | 0.070509191 |
| GFAP | Right pars triangularis | 0.421849725 | 0.194248202 | 2.171704654 | 0.040935735 | 0.07485516 |
| GFAP | Right pericalcarine | 0.433163661 | 0.193202289 | 2.242021367 | 0.035369356 | 0.070509191 |
| GFAP | Right postcentral | 0.540016998 | 0.181762428 | 2.971004531 | 0.007052349 | 0.070509191 |
| GFAP | Right posterior cingulate | 0.441746685 | 0.191249869 | 2.309788174 | 0.030666868 | 0.070509191 |
| GFAP | Right precentral | 0.478452415 | 0.194102196 | 2.464951067 | 0.021982888 | 0.070509191 |
| GFAP | Right precuneus | 0.486460648 | 0.192489781 | 2.527202457 | 0.019190159 | 0.070509191 |
| GFAP | Right rostral anterior cingulate | 0.354543684 | 0.191307025 | 1.853270594 | 0.077304045 | - |
| GFAP | Right rostral middle frontal | 0.470539779 | 0.191402073 | 2.458383922 | 0.022298587 | 0.070509191 |
| GFAP | Right superior frontal | 0.435175239 | 0.194801908 | 2.233937248 | 0.035972247 | 0.070509191 |
| GFAP | Right superior parietal | 0.520057029 | 0.189880705 | 2.738861909 | 0.01198387 | 0.070509191 |
| GFAP | Right superior temporal | 0.45402738 | 0.192583368 | 2.357562778 | 0.027704187 | 0.070509191 |
| GFAP | Right supramarginal | 0.552201353 | 0.183404382 | 3.010840566 | 0.006430927 | 0.070509191 |
| GFAP | Right frontal pole | 0.438707558 | 0.188876433 | 2.32272259 | 0.029837267 | 0.070509191 |
| GFAP | Right temporal pole | 0.446567073 | 0.199979644 | 2.233062648 | 0.036038031 | 0.070509191 |
| GFAP | Right transverse temporal | 0.165671748 | 0.205393118 | 0.806608079 | 0.428526394 | - |
| GFAP | Right insula | 0.457209415 | 0.191910431 | 2.382410441 | 0.026269719 | 0.070509191 |

| Plasma measure | Brain regions (dopamine uptake) | β | Standard error | t | P | P value (FDR corrected) |
| --- | --- | --- | --- | --- | --- | --- |
| NfL | Left cerebral white matter | 0.030199944 | 0.225833187 | 0.133726774 | 0.894834393 | - |
| NfL | Left cerebellum white matter | 0.084020796 | 0.223074625 | 0.376648828 | 0.7100429 | - |
| NfL | Left cerebellum cortex | -0.004608959 | 0.207973046 | -0.022161328 | 0.982519043 | - |
| NfL | Left thalamus | 0.011373561 | 0.223293069 | 0.050935574 | 0.959836503 | - |
| NfL | Left caudate | -0.067454672 | 0.225811775 | -0.298720792 | 0.767956503 | - |
| NfL | Left putamen | -0.142550739 | 0.223962153 | -0.636494767 | 0.531021302 | - |
| NfL | Left pallidum | 0.035144478 | 0.226174236 | 0.155386744 | 0.877933326 | - |
| NfL | Brain stem | -0.10455954 | 0.222681945 | -0.469546555 | 0.643299538 | - |
| NfL | Left hippocampus | -0.163505152 | 0.212511213 | -0.769395413 | 0.449840949 | - |
| NfL | Left amygdala | 0.021944031 | 0.219474574 | 0.099984387 | 0.921262264 | - |
| NfL | Left accumbens | -0.030161988 | 0.224350555 | -0.134441334 | 0.894275967 | - |
| NfL | Right cerebral white matter | 0.046034545 | 0.226208433 | 0.20350499 | 0.840610936 | - |
| NfL | Right cerebellum white matter | 0.078143669 | 0.222964791 | 0.350475377 | 0.729315478 | - |
| NfL | Right cerebellum cortex | 0.004608959 | 0.207973046 | 0.022161328 | 0.982519043 | - |
| NfL | Right thalamus | -0.020616683 | 0.222835616 | -0.092519694 | 0.927122496 | - |
| NfL | Right caudate | -0.072160482 | 0.22436772 | -0.32161704 | 0.750778623 | - |
| NfL | Right putamen | -0.116405465 | 0.224334343 | -0.518892754 | 0.609016203 | - |
| NfL | Right pallidum | 0.023215393 | 0.225801296 | 0.102813372 | 0.919042517 | - |
| NfL | Right hippocampus | -0.081798014 | 0.213949936 | -0.382323154 | 0.705890166 | - |
| NfL | Right amygdala | -0.103818932 | 0.216765326 | -0.478946213 | 0.636702968 | - |
| NfL | Right accumbens | -0.069765997 | 0.224687069 | -0.310502945 | 0.759101094 | - |
| NfL | Pons | 0.034232753 | 0.222655228 | 0.153747809 | 0.879210198 | - |
| NfL | Left bankssts | 0.000943079 | 0.217861138 | 0.004328808 | 0.996585135 | - |
| NfL | Left caudal anterior cingulate | 0.006250959 | 0.208231649 | 0.030019256 | 0.976322365 | - |
| NfL | Left caudal middle frontal | 0.073097569 | 0.219286335 | 0.333343019 | 0.742031475 | - |
| NfL | Left cuneus | -0.042952703 | 0.213084265 | -0.201576137 | 0.842100302 | - |
| NfL | Left entorhinal | 0.045166643 | 0.224300213 | 0.201366921 | 0.842261885 | - |
| NfL | Left fusiform | 0.055530786 | 0.221992182 | 0.250147486 | 0.804794427 | - |
| NfL | Left inferior parietal | 0.089476175 | 0.21368464 | 0.418730025 | 0.679473184 | - |
| NfL | Left inferior temporal | 0.043080997 | 0.218748281 | 0.196943249 | 0.845680051 | - |
| NfL | Left isthmus cingulate | 0.010171904 | 0.219314382 | 0.046380468 | 0.963425453 | - |
| NfL | Left lateral occipital | 0.009128396 | 0.221329917 | 0.041243389 | 0.967473882 | - |
| NfL | Left lateral orbitofrontal | -0.261521518 | 0.213028213 | -1.227637944 | 0.232556954 | - |
| NfL | Left lingual | -0.127947337 | 0.211129268 | -0.606014213 | 0.550707429 | - |
| NfL | Left medial orbitofrontal | -0.391674682 | 0.205597948 | -1.905051509 | 0.069928122 | - |
| NfL | Left middle temporal | 0.072747532 | 0.212822195 | 0.341823051 | 0.735727736 | - |
| NfL | Left parahippocampal | 0.021064002 | 0.222170914 | 0.094809899 | 0.925324082 | - |
| NfL | Left paracentral | 0.082582827 | 0.215002013 | 0.384102576 | 0.704589822 | - |
| NfL | Left pars opercularis | 0.04711336 | 0.2125298 | 0.221678843 | 0.826608678 | - |
| NfL | Left pars orbitalis | -0.078450403 | 0.20984601 | -0.373847484 | 0.712096464 | - |
| NfL | Left pars triangularis | 0.05916666 | 0.210045068 | 0.281685548 | 0.780817291 | - |
| NfL | Left pericalcarine | -0.037091269 | 0.213501829 | -0.173728109 | 0.863667778 | - |
| NfL | Left postcentral | 0.052286564 | 0.211200119 | 0.247568818 | 0.806763956 | - |
| NfL | Left posterior cingulate | -0.012753165 | 0.213542805 | -0.059721819 | 0.952916385 | - |
| NfL | Left precentral | 0.020969876 | 0.215638499 | 0.097245509 | 0.923411935 | - |
| NfL | Left precuneus | 0.016136523 | 0.213619104 | 0.075538763 | 0.940468768 | - |
| NfL | Left rostral anterior cingulate | 0.09371064 | 0.211416808 | 0.443250664 | 0.661912665 | - |
| NfL | Left rostral middle frontal | 0.063477314 | 0.215313328 | 0.294813677 | 0.770900306 | - |
| NfL | Left superior frontal | 0.018216215 | 0.216718693 | 0.084054655 | 0.933773159 | - |
| NfL | Left superior parietal | 0.091294088 | 0.216713375 | 0.421266513 | 0.677647804 | - |
| NfL | Left superior temporal | 0.003376534 | 0.217004627 | 0.015559733 | 0.987725879 | - |
| NfL | Left supramarginal | 0.062493523 | 0.216103791 | 0.289182911 | 0.775148994 | - |
| NfL | Left frontal pole | 0.033823784 | 0.209450001 | 0.161488583 | 0.873182477 | - |
| NfL | Left temporal pole | 0.079544506 | 0.217537017 | 0.365659633 | 0.718111444 | - |
| NfL | Left transverse temporal | -0.110911154 | 0.21987173 | -0.504435717 | 0.618970273 | - |
| NfL | Left insula | -0.039952424 | 0.2143689 | -0.186372295 | 0.853860681 | - |
| NfL | Right bankssts | 0.039341154 | 0.218604553 | 0.179964936 | 0.858827452 | - |
| NfL | Right caudal anterior cingulate | -0.046252709 | 0.217966254 | -0.212201238 | 0.833903772 | - |
| NfL | Right caudal middle frontal | 0.103651681 | 0.219914052 | 0.471328138 | 0.64204691 | - |
| NfL | Right cuneus | -0.009275881 | 0.209122929 | -0.044356119 | 0.965020688 | - |
| NfL | Right entorhinal | -0.07698878 | 0.221143856 | -0.348138904 | 0.731045072 | - |
| NfL | Right fusiform | 0.022966663 | 0.220592256 | 0.104113641 | 0.918022494 | - |
| NfL | Right inferior parietal | 0.077950813 | 0.208280428 | 0.374258943 | 0.711794698 | - |
| NfL | Right inferior temporal | 0.050579325 | 0.219122453 | 0.230826758 | 0.819582493 | - |
| NfL | Right isthmus cingulate | -0.004378265 | 0.214593159 | -0.020402631 | 0.983906101 | - |
| NfL | Right lateral occipital | 0.058589433 | 0.212580582 | 0.275610463 | 0.78541946 | - |
| NfL | Right lateral orbitofrontal | 0.044292942 | 0.215092056 | 0.205925512 | 0.838742791 | - |
| NfL | Right lingual | -0.055616103 | 0.223669643 | -0.248652887 | 0.805935807 | - |
| NfL | Right medial orbitofrontal | 0.057464564 | 0.213025485 | 0.269754411 | 0.78986331 | - |
| NfL | Right middle temporal | 0.062662494 | 0.212655882 | 0.294666171 | 0.771011514 | - |
| NfL | Right parahippocampal | 0.014138001 | 0.226634504 | 0.062382383 | 0.950821626 | - |
| NfL | Right paracentral | 0.02471046 | 0.213212178 | 0.115896102 | 0.908786288 | - |
| NfL | Right pars opercularis | -0.022537057 | 0.216488452 | -0.104102813 | 0.918030987 | - |
| NfL | Right pars orbitalis | -0.10786229 | 0.213076341 | -0.506214298 | 0.617741578 | - |
| NfL | Right pars triangularis | -0.010745359 | 0.2080492 | -0.051648162 | 0.959275135 | - |
| NfL | Right pericalcarine | -0.101336411 | 0.2036263 | -0.497658754 | 0.623662418 | - |
| NfL | Right postcentral | 0.017712805 | 0.217518 | 0.081431444 | 0.935835145 | - |
| NfL | Right posterior cingulate | -0.038789083 | 0.21267578 | -0.182385991 | 0.856950017 | - |
| NfL | Right precentral | -0.029846335 | 0.218701432 | -0.136470689 | 0.892690341 | - |
| NfL | Right precuneus | -0.004987741 | 0.215667141 | -0.023127033 | 0.981757429 | - |
| NfL | Right rostral anterior cingulate | -0.109294575 | 0.210475451 | -0.519274693 | 0.608754262 | - |
| NfL | Right rostral middle frontal | -0.016034454 | 0.21939781 | -0.07308393 | 0.942399735 | - |
| NfL | Right superior frontal | 0.009749098 | 0.216858579 | 0.044956018 | 0.964547937 | - |
| NfL | Right superior parietal | 0.081908016 | 0.215761078 | 0.37962369 | 0.7078646 | - |
| NfL | Right superior temporal | -0.018248738 | 0.21963811 | -0.083085481 | 0.934534928 | - |
| NfL | Right supramarginal | 0.058614012 | 0.21675369 | 0.270417599 | 0.789359681 | - |
| NfL | Right frontal pole | 0.081571986 | 0.215706136 | 0.378162566 | 0.708934169 | - |
| NfL | Right temporal pole | 0.085388983 | 0.224203514 | 0.380854793 | 0.706963889 | - |
| NfL | Right transverse temporal | -0.163045513 | 0.21834021 | -0.746749823 | 0.463121023 | - |
| NfL | Right insula | 0.006488985 | 0.220691291 | 0.029402995 | 0.976808292 | - |

| Plasma measure | Brain regions (dopamine uptake) | β | Standard error | t | P | P value (FDR corrected) |
| --- | --- | --- | --- | --- | --- | --- |
| GFAP | Left cerebral white matter | 0.036629452 | 0.21864488 | 0.16752943 | 0.868483928 | - |
| GFAP | Left cerebellum white matter | 0.07357978 | 0.216150937 | 0.340409256 | 0.736777391 | - |
| GFAP | Left cerebellum cortex | -0.101319726 | 0.20024025 | -0.505990811 | 0.617895906 | - |
| GFAP | Left thalamus | 0.047090321 | 0.216015171 | 0.217995434 | 0.829442007 | - |
| GFAP | Left caudate | 0.033514813 | 0.219001198 | 0.153034839 | 0.879765769 | - |
| GFAP | Left putamen | 0.038647815 | 0.218716208 | 0.176703021 | 0.86135829 | - |
| GFAP | Left pallidum | -0.031885517 | 0.219040399 | -0.145569114 | 0.885587064 | - |
| GFAP | Brain stem | 0.036148811 | 0.216584562 | 0.166903913 | 0.868970226 | - |
| GFAP | Left hippocampus | 0.098153469 | 0.207492286 | 0.473046355 | 0.640839866 | - |
| GFAP | Left amygdala | 0.078917665 | 0.211919206 | 0.372395059 | 0.713162065 | - |
| GFAP | Left accumbens | -0.034782125 | 0.217222352 | -0.160122218 | 0.874245901 | - |
| GFAP | Right cerebral white matter | 0.047626565 | 0.219029671 | 0.217443438 | 0.829866815 | - |
| GFAP | Right cerebellum white matter | 0.024884972 | 0.216454617 | 0.114966233 | 0.909514745 | - |
| GFAP | Right cerebellum cortex | 0.101319726 | 0.20024025 | 0.505990811 | 0.617895906 | - |
| GFAP | Right thalamus | 0.049212301 | 0.215579413 | 0.228279222 | 0.821537636 | - |
| GFAP | Right caudate | 0.019718931 | 0.217745891 | 0.090559373 | 0.92866218 | - |
| GFAP | Right putamen | 0.00298564 | 0.218568378 | 0.013659982 | 0.989224372 | - |
| GFAP | Right pallidum | -0.022342713 | 0.2186652 | -0.102177727 | 0.919541214 | - |
| GFAP | Right hippocampus | 0.113121171 | 0.206471133 | 0.54787887 | 0.589291367 | - |
| GFAP | Right amygdala | -0.017454163 | 0.210972888 | -0.082731781 | 0.934812951 | - |
| GFAP | Right accumbens | -0.006555423 | 0.218057312 | -0.030062845 | 0.976287995 | - |
| GFAP | Pons | 0.025874066 | 0.215663187 | 0.119974422 | 0.905592316 | - |
| GFAP | Left bankssts | 0.092945923 | 0.210042754 | 0.442509543 | 0.662440581 | - |
| GFAP | Left caudal anterior cingulate | 0.172332965 | 0.198278937 | 0.869144085 | 0.394151931 | - |
| GFAP | Left caudal middle frontal | 0.228213697 | 0.207256568 | 1.101116839 | 0.282747818 | - |
| GFAP | Left cuneus | 0.168837726 | 0.203378975 | 0.830163127 | 0.415364747 | - |
| GFAP | Left entorhinal | 0.089236546 | 0.216576957 | 0.412031579 | 0.684303359 | - |
| GFAP | Left fusiform | 0.160305965 | 0.212551084 | 0.754199706 | 0.458726561 | - |
| GFAP | Left inferior parietal | 0.193360258 | 0.203622655 | 0.949600906 | 0.352631045 | - |
| GFAP | Left inferior temporal | 0.117157898 | 0.210544635 | 0.556451598 | 0.583518583 | - |
| GFAP | Left isthmus cingulate | 0.109373472 | 0.211109106 | 0.518089786 | 0.609567067 | - |
| GFAP | Left lateral occipital | 0.103537883 | 0.213203076 | 0.485630341 | 0.632030748 | - |
| GFAP | Left lateral orbitofrontal | 0.123133872 | 0.211622087 | 0.581857375 | 0.566578005 | - |
| GFAP | Left lingual | 0.028572436 | 0.206065697 | 0.138656924 | 0.89098265 | - |
| GFAP | Left medial orbitofrontal | 0.093897444 | 0.213960902 | 0.438853279 | 0.665047638 | - |
| GFAP | Left middle temporal | 0.170840904 | 0.203406861 | 0.83989745 | 0.410000918 | - |
| GFAP | Left parahippocampal | 0.116702583 | 0.21374962 | 0.545977966 | 0.590575224 | - |
| GFAP | Left paracentral | 0.176837183 | 0.205473282 | 0.860633464 | 0.398722542 | - |
| GFAP | Left pars opercularis | 0.190485417 | 0.202000262 | 0.942995891 | 0.355924251 | - |
| GFAP | Left pars orbitalis | 0.192702239 | 0.199675178 | 0.965078589 | 0.344995006 | - |
| GFAP | Left pars triangularis | 0.26461533 | 0.195807479 | 1.351405631 | 0.190299457 | - |
| GFAP | Left pericalcarine | 0.186742364 | 0.203028731 | 0.919782948 | 0.367662011 | - |
| GFAP | Left postcentral | 0.284351919 | 0.195631499 | 1.453507848 | 0.16019994 | - |
| GFAP | Left posterior cingulate | 0.225894637 | 0.201124378 | 1.123158908 | 0.273477725 | - |
| GFAP | Left precentral | 0.211393675 | 0.203947382 | 1.036510852 | 0.311223174 | - |
| GFAP | Left precuneus | 0.217397076 | 0.201635733 | 1.078167407 | 0.292639283 | - |
| GFAP | Left rostral anterior cingulate | 0.240777282 | 0.1991368 | 1.209104907 | 0.239456928 | - |
| GFAP | Left rostral middle frontal | 0.280655861 | 0.200167488 | 1.402105128 | 0.174836627 | - |
| GFAP | Left superior frontal | 0.180821297 | 0.20633214 | 0.876360306 | 0.390303144 | - |
| GFAP | Left superior parietal | 0.189359006 | 0.206804891 | 0.915640847 | 0.369783315 | - |
| GFAP | Left superior temporal | 0.132684854 | 0.208234366 | 0.637189992 | 0.530576774 | - |
| GFAP | Left supramarginal | 0.220657976 | 0.204325023 | 1.079936136 | 0.291868208 | - |
| GFAP | Left frontal pole | 0.12917491 | 0.201072969 | 0.642428022 | 0.527234075 | - |
| GFAP | Left temporal pole | 0.06744333 | 0.210810853 | 0.319923422 | 0.752044868 | - |
| GFAP | Left transverse temporal | 0.153647242 | 0.211629968 | 0.726018362 | 0.475481155 | - |
| GFAP | Left insula | 0.194563039 | 0.203574131 | 0.955735573 | 0.349590873 | - |
| GFAP | Right bankssts | 0.129783337 | 0.210036281 | 0.617909136 | 0.54297947 | - |
| GFAP | Right caudal anterior cingulate | 0.142669353 | 0.209092198 | 0.682327485 | 0.502153582 | - |
| GFAP | Right caudal middle frontal | 0.271799337 | 0.206042277 | 1.319143533 | 0.200687134 | - |
| GFAP | Right cuneus | 0.244304004 | 0.195709953 | 1.248296268 | 0.225044684 | - |
| GFAP | Right entorhinal | 0.028270718 | 0.214658805 | 0.131700715 | 0.896418052 | - |
| GFAP | Right fusiform | 0.132122555 | 0.211807889 | 0.623784862 | 0.539183513 | - |
| GFAP | Right inferior parietal | 0.269656354 | 0.193999149 | 1.389987301 | 0.178438276 | - |
| GFAP | Right inferior temporal | 0.156206243 | 0.209827131 | 0.744452076 | 0.464481443 | - |
| GFAP | Right isthmus cingulate | 0.156101178 | 0.205130377 | 0.760985183 | 0.454745843 | - |
| GFAP | Right lateral occipital | 0.185884254 | 0.202372861 | 0.918523627 | 0.368306091 | - |
| GFAP | Right lateral orbitofrontal | 0.175012808 | 0.205128453 | 0.853186409 | 0.402749849 | - |
| GFAP | Right lingual | 0.112130378 | 0.215582975 | 0.520126316 | 0.608170398 | - |
| GFAP | Right medial orbitofrontal | 0.206792675 | 0.201875183 | 1.024359069 | 0.316798071 | - |
| GFAP | Right middle temporal | 0.232025884 | 0.200323119 | 1.158258147 | 0.259178249 | - |
| GFAP | Right parahippocampal | 0.097531091 | 0.21850366 | 0.446359073 | 0.659700437 | - |
| GFAP | Right paracentral | 0.315888942 | 0.195247394 | 1.617890699 | 0.119935339 | - |
| GFAP | Right pars opercularis | 0.191769459 | 0.205673293 | 0.932398446 | 0.361251247 | - |
| GFAP | Right pars orbitalis | 0.190564784 | 0.203524406 | 0.936323991 | 0.359271796 | - |
| GFAP | Right pars triangularis | 0.203431408 | 0.196762295 | 1.033894267 | 0.31241771 | - |
| GFAP | Right pericalcarine | 0.196614024 | 0.193816048 | 1.014436247 | 0.321402057 | - |
| GFAP | Right postcentral | 0.248411672 | 0.203909165 | 1.218246728 | 0.236034297 | - |
| GFAP | Right posterior cingulate | 0.111923792 | 0.204723551 | 0.546706968 | 0.590082699 | - |
| GFAP | Right precentral | 0.187638251 | 0.208067828 | 0.901812898 | 0.376923824 | - |
| GFAP | Right precuneus | 0.198260511 | 0.20453113 | 0.96934149 | 0.342911814 | - |
| GFAP | Right rostral anterior cingulate | 0.168632212 | 0.201892203 | 0.835258663 | 0.412551483 | - |
| GFAP | Right rostral middle frontal | 0.229237759 | 0.206792277 | 1.1085412 | 0.279600272 | - |
| GFAP | Right superior frontal | 0.200977463 | 0.205596538 | 0.977533302 | 0.338932821 | - |
| GFAP | Right superior parietal | 0.237504201 | 0.203417277 | 1.167571433 | 0.255478564 | - |
| GFAP | Right superior temporal | 0.115582552 | 0.211297445 | 0.547013484 | 0.589875672 | - |
| GFAP | Right supramarginal | 0.250451654 | 0.203358048 | 1.231579749 | 0.23110903 | - |
| GFAP | Right frontal pole | 0.222926723 | 0.204105618 | 1.092212575 | 0.286556533 | - |
| GFAP | Right temporal pole | 0.064915609 | 0.217391731 | 0.298611216 | 0.768039014 | - |
| GFAP | Right transverse temporal | 0.046724056 | 0.213870328 | 0.218469091 | 0.82907753 | - |
| GFAP | Right insula | 0.165193542 | 0.210798345 | 0.783656728 | 0.441597341 | - |

Abbreviations: FDR, false discovery rate; GFAP, glial fibrillar acidic protein; MSA, multiple system atrophy; NfL, neurofilament light; PET, positron emission tomography.

Note: Associations of plasma measures with PET indices were analyzed using multiple linear regressions after adjusting for age and sex.

**Supplementary Table 7: Demographic characteristics and plasma patterns per diagnostic group after expanding the sample size**

| Characteristic | HC | MSA | MSA-C | MSA-P | SCA | PD | P value |
| --- | --- | --- | --- | --- | --- | --- | --- |
|  | N = 100 | N = 76 | N = 60 | N = 16 | N = 60 | N = 39 |  |
| Age, years | 59.50 (8.91) | 58.70 (7.76) | 58.98 (7.98) | 57.62 (7.02) | 46.32 (12.47) | 63.77 (10.12) | <0.001 |
| Male, n (%) | 44.00 (44.00%) | 42.00 (55.26%) | 34.00 (56.67%) | 8.00 (50.00%) | 30.00 (50.00%) | 21.00 (53.85%) | 0.474 |
| Disease duration, months | – | 20.76 (10.70) | 21.55 (10.06) | 17.81 (12.72) | 55.08 (47.12) | 33.92 (26.71) | <0.001 |
| Education, years | 9.69 (4.22) | 8.11 (4.27) | 8.51 (4.22) | 6.67 (4.27) | 9.95 (4.01) | 7.92 (3.96) | 0.032 |
| Plasma NfL, pg/mL | 13.74 (8.53) | 40.64 (44.02) | 42.90 (48.82) | 32.15 (14.63) | 33.19 (24.54) | 26.97 (19.62) | <0.001 |
| Plasma GFAP, pg/mL | 69.20 (35.69) | 92.38 (57.23) | 98.35 (61.48) | 69.98 (28.87) | 68.63 (40.77) | 114.78 (57.04) | <0.001 |
| Plasma p-tau181, pg/mL | 1.99 (0.84) | 1.44 (0.66) | 1.41 (0.66) | 1.56 (0.66) | 1.46 (0.52) | 1.81 (1.02) | <0.001 |
| Plasma Aβ40, pg/mL | 83.02 (25.78) | 64.43 (23.04) | 66.50 (24.79) | 56.67 (12.49) | 69.60 (23.76) | 87.58 (28.38) | 0.001 |
| Plasma Aβ42, pg/mL | 4.96 (1.63) | 4.46 (1.40) | 4.61 (1.43) | 3.93 (1.19) | 4.78 (1.36) | 5.65 (1.76) | 0.015 |
| Plasma Aβ42/40 | 0.06 (0.02) | 0.07 (0.01) | 0.07 (0.01) | 0.07 (0.01) | 0.07 (0.01) | 0.07 (0.01) | 0.002 |

Note: We expanded the sample size irrespective of the duration of symptoms. Continuous data are described as mean (standard deviations), and categorical variables are presented as numbers (percentages).

Abbreviations: Αβ, amyloid-β; GFAP, glial fibrillary acidic protein; HC, healthy control; MSA, multiple system atrophy; MSA-C, multiple system atrophy-cerebellar type; MSA-P, multiple system atrophy-parkinsonian type; NfL, neurofilament light; PD, Parkinson's disease; p-tau181, phosphorylated tau at threonine 181; SCA, spinocerebellar ataxia.

**Supplementary Figure 2: Distributions of the plasma levels across diagnostic groups after expanding the sample size**


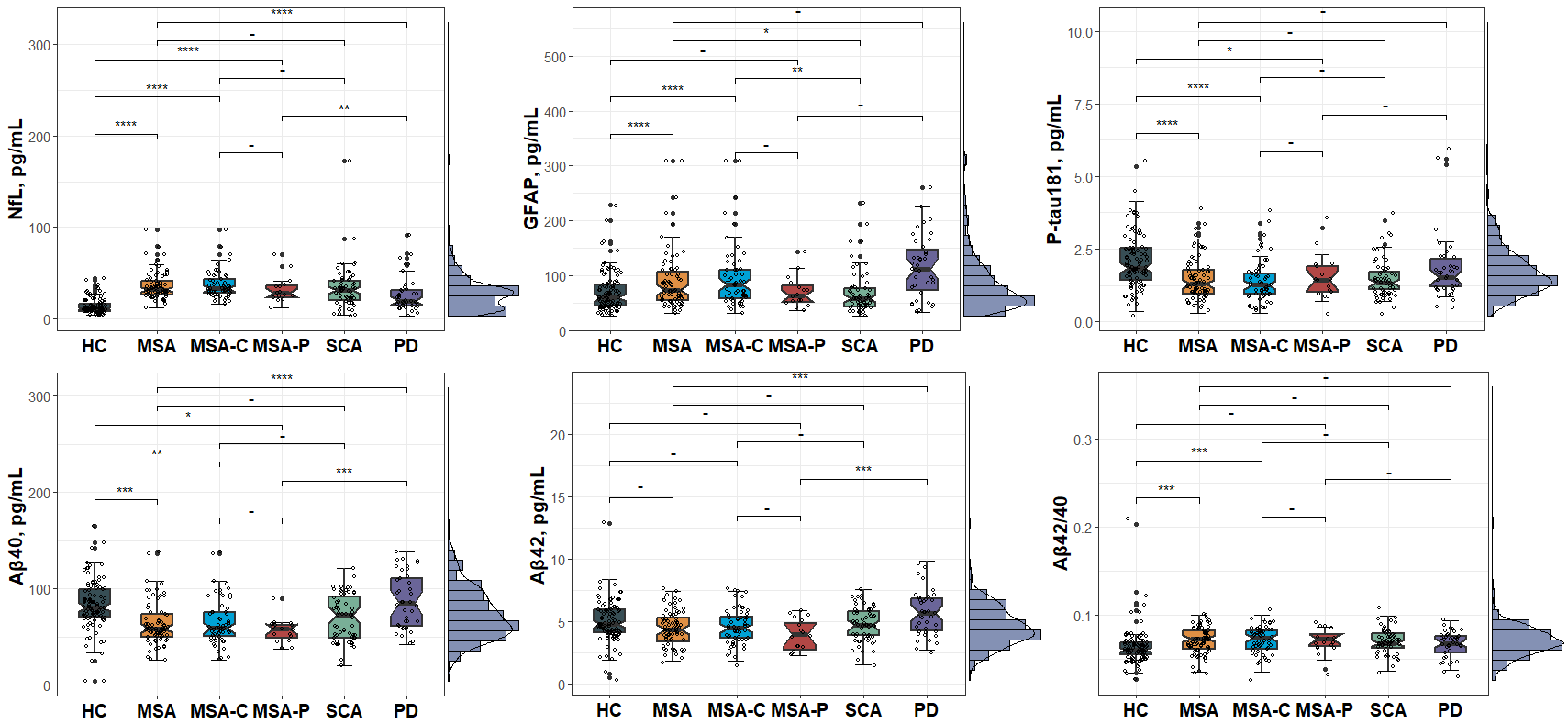


Plasma levels of NfL, GFAP, p-tau181, Αβ40, Aβ42, and Αβ42/40 per diagnostic group were compared using analysis of covariance after controlling for age and sex. Significance: ****p<0.0001, ***p<0.001, **p<0.01, *p<0.05, -: p≥0.05.

Abbreviations: Αβ, amyloid-β; GFAP, glial fibrillary acidic protein; HC, healthy control; MSA, multiple system atrophy; MSA-C, multiple system atrophy-cerebellar type; MSA-P, multiple system atrophy-parkinsonian type; NfL, neurofilament light; ns, non-significant; PD, Parkinson's disease; p-tau181, phosphorylated tau at threonine 181; SCA, spinocerebellar ataxia.

**Supplementary Table 8: Results of receiver operating characteristic (ROC) analyses after expanding the sample size**

ROC analyses of single plasma biomarker

| MSA versus HC | NFL | GFAP | P-tau 181 | Aβ40 | Aβ42 | Aβ42/40 |
| --- | --- | --- | --- | --- | --- | --- |
| AUC (95% CI) | 0.931 (0.894, 0.967) | 0.642 (0.56, 0.724) | 0.713 (0.634, 0.791) | 0.757 (0.68, 0.833) | 0.605 (0.52, 0.69) | 0.725 (0.647, 0.804) |
| Cutoff | 22.680 | 51.798 | 1.422 | 67.582 | 3.958 | 0.071 |
| Sensitivity | 0.907 | 0.827 | 0.742 | 0.830 | 0.800 | 0.560 |
| Specificity | 0.860 | 0.410 | 0.627 | 0.720 | 0.373 | 0.860 |
| False positive rate | 0.140 | 0.590 | 0.373 | 0.280 | 0.627 | 0.140 |
| False negative rate | 0.093 | 0.173 | 0.258 | 0.170 | 0.200 | 0.440 |
| Positive predictive value | 0.829 | 0.512 | 0.720 | 0.798 | 0.630 | 0.750 |
| Negative predictive value | 0.925 | 0.759 | 0.653 | 0.761 | 0.583 | 0.723 |
|  |  |  |  |  |  |  |
|  |  |  |  |  |  |  |
| MSA-C versus HC | NFL | GFAP | P-tau 181 | Aβ40 | Aβ42 | Aβ42/40 |
| AUC (95% CI) | 0.94 (0.905, 0.974) | 0.669 (0.584, 0.755) | 0.727 (0.643, 0.811) | 0.728 (0.641, 0.815) | 0.579 (0.487, 0.672) | 0.732 (0.648, 0.816) |
| Cutoff | 22.680 | 83.364 | 1.422 | 67.582 | 4.850 | 0.071 |
| Sensitivity | 0.932 | 0.508 | 0.742 | 0.830 | 0.480 | 0.559 |
| Specificity | 0.860 | 0.760 | 0.661 | 0.661 | 0.661 | 0.860 |
| False positive rate | 0.140 | 0.240 | 0.339 | 0.339 | 0.339 | 0.140 |
| False negative rate | 0.068 | 0.492 | 0.258 | 0.170 | 0.520 | 0.441 |
| Positive predictive value | 0.797 | 0.556 | 0.783 | 0.806 | 0.706 | 0.702 |
| Negative predictive value | 0.956 | 0.724 | 0.609 | 0.696 | 0.429 | 0.768 |
|  |  |  |  |  |  |  |
|  |  |  |  |  |  |  |
| MSA-P versus HC | NFL | GFAP | P-tau 181 | Aβ40 | Aβ42 | Aβ42/40 |
| AUC (95% CI) | 0.897 (0.832, 0.963) | 0.541 (0.4, 0.683) | 0.66 (0.51, 0.811) | 0.861 (0.771, 0.951) | 0.701 (0.563, 0.84) | 0.7 (0.541, 0.859) |
| Cutoff | 20.248 | 53.151 | 1.843 | 67.144 | 4.007 | 0.066 |
| Sensitivity | 0.938 | 0.750 | 0.515 | 0.840 | 0.780 | 0.750 |
| Specificity | 0.810 | 0.430 | 0.750 | 0.938 | 0.563 | 0.680 |
| False positive rate | 0.190 | 0.570 | 0.250 | 0.063 | 0.438 | 0.320 |
| False negative rate | 0.063 | 0.250 | 0.485 | 0.160 | 0.220 | 0.250 |
| Positive predictive value | 0.441 | 0.174 | 0.926 | 0.988 | 0.918 | 0.273 |
| Negative predictive value | 0.988 | 0.915 | 0.203 | 0.484 | 0.290 | 0.944 |
|  |  |  |  |  |  |  |
|  |  |  |  |  |  |  |
| MSA-C versus MSA-P | NFL | GFAP | P-tau 181 | Aβ40 | Aβ42 | Aβ42/40 |
| AUC (95% CI) | 0.625 (0.454, 0.796) | 0.639 (0.49, 0.788) | 0.587 (0.431, 0.743) | 0.592 (0.449, 0.736) | 0.619 (0.462, 0.776) | 0.542 (0.383, 0.702) |
| Cutoff | 29.587 | 81.298 | 1.489 | 66.150 | 4.015 | 0.076 |
| Sensitivity | 0.661 | 0.525 | 0.500 | 0.373 | 0.661 | 0.458 |
| Specificity | 0.625 | 0.750 | 0.695 | 0.938 | 0.563 | 0.750 |
| False positive rate | 0.375 | 0.250 | 0.305 | 0.063 | 0.438 | 0.250 |
| False negative rate | 0.339 | 0.475 | 0.500 | 0.627 | 0.339 | 0.542 |
| Positive predictive value | 0.867 | 0.886 | 0.308 | 0.957 | 0.848 | 0.871 |
| Negative predictive value | 0.333 | 0.300 | 0.837 | 0.288 | 0.310 | 0.273 |
|  |  |  |  |  |  |  |
|  |  |  |  |  |  |  |
|  |  |  |  |  |  |  |
| MSA-C versus SCA | NFL | GFAP | P-tau 181 | Aβ40 | Aβ42 | Aβ42/40 |
| AUC (95% CI) | 0.59 (0.486, 0.694) | 0.691 (0.595, 0.786) | 0.562 (0.457, 0.666) | 0.544 (0.438, 0.65) | 0.554 (0.449, 0.658) | 0.559 (0.454, 0.665) |
| Cutoff | 22.680 | 61.548 | 1.040 | 70.315 | 4.835 | 0.071 |
| Sensitivity | 0.932 | 0.712 | 0.850 | 0.533 | 0.475 | 0.593 |
| Specificity | 0.317 | 0.600 | 0.339 | 0.678 | 0.644 | 0.610 |
| False positive rate | 0.683 | 0.400 | 0.661 | 0.322 | 0.356 | 0.390 |
| False negative rate | 0.068 | 0.288 | 0.150 | 0.467 | 0.525 | 0.407 |
| Positive predictive value | 0.573 | 0.636 | 0.567 | 0.627 | 0.571 | 0.603 |
| Negative predictive value | 0.826 | 0.679 | 0.690 | 0.588 | 0.551 | 0.600 |
|  |  |  |  |  |  |  |
|  |  |  |  |  |  |  |
| MSA-P versus PD | NFL | GFAP | P-tau 181 | Aβ40 | Aβ42 | Aβ42/40 |
| AUC (95% CI) | 0.668 (0.524, 0.813) | 0.726 (0.589, 0.863) | 0.561 (0.388, 0.734) | 0.825 (0.715, 0.935) | 0.78 (0.653, 0.908) | 0.588 (0.414, 0.762) |
| Cutoff | 20.248 | 87.205 | 1.047 | 65.769 | 5.491 | 0.072 |
| Sensitivity | 0.938 | 0.667 | 0.868 | 0.718 | 0.538 | 0.563 |
| Specificity | 0.513 | 0.813 | 0.313 | 0.938 | 0.938 | 0.692 |
| False positive rate | 0.487 | 0.188 | 0.688 | 0.063 | 0.063 | 0.308 |
| False negative rate | 0.063 | 0.333 | 0.132 | 0.282 | 0.462 | 0.438 |
| Positive predictive value | 0.441 | 0.897 | 0.750 | 0.966 | 0.955 | 0.429 |
| Negative predictive value | 0.952 | 0.500 | 0.500 | 0.577 | 0.455 | 0.794 |

ROC analyses of biomarker panels combining multiple variables

(Note: the tested biomarker panel in the reverse stepwise regression procedure: plasma NfL + GFAP + p-tau181 + Aβ42 + Aβ40 + Aβ42/40)

| Diagnostic groups | Derived marker panel with the lowest AIC | AUC | 95% CI | Specificity | Sensitivity | False positive rate | False negative rate |
| --- | --- | --- | --- | --- | --- | --- | --- |
| MSA versus HC | NfL + GFAP + p-tau181 + Aβ42 + Aβ40 + Aβ42/40 | 0.996 | 0.9901-1.0000 | 0.969 | 0.973 | 0.031 | 0.027 |
| MSA-C versus HC | NfL + GFAP + p-tau181 + Aβ42 + Aβ40 + Aβ42/40 | 0.997 | 0.993-1.000 | 0.979 | 0.983 | 0.021 | 0.017 |
| MSA-P versus HC | NfL + p-tau181 + Aβ42 + Aβ40 + Aβ42/40 | 0.990 | 0.9775-1.0000 | 0.928 | 1.000 | 0.072 | 0.000 |
| MSA-C versus MSA-P | Aβ42 + Aβ40 + Aβ42/40 | 0.724 | 0.5865-0.8606 | 0.875 | 0.542 | 0.125 | 0.458 |
| MSA-C versus SCA | GFAP + Aβ42 + Aβ42/40 | 0.710 | 0.6169-0.8022 | 0.373 | 0.949 | 0.627 | 0.051 |
| MSA-P versus PD | NfL + GFAP + Aβ40 | 0.918 | 0.8338-1.0000 | 0.947 | 0.813 | 0.053 | 0.188 |

Note: Optimal cutoff point was chosen at the highest Youden index. To examine if different multimodal combinations of variables were superior to unimodal metrics, all plasma biomarkers tested were entered into the binary logistic regression models to obtain probabilities for each individual. The desired biomarker panel was determined when the lowest AIC value was obtained during a reverse stepwise regression procedure. Only the best panels selected were shown in this study.

Abbreviations: Αβ, amyloid-β; AIC, Akaike information criterion; AUC, area under the curve; CI, confidence interval; GFAP, glial fibrillary acidic protein; HC, healthy control; MSA, multiple system atrophy; MSA-C, multiple system atrophy-cerebellar type; MSA-P, multiple system atrophy-parkinsonian type; NfL, neurofilament light; PD, Parkinson's disease; p-tau181, phosphorylated tau at threonine 181; SCA, spinocerebellar ataxia.

**Supplementary Table 9: Relationship of plasma indicators with clinical assessment scales in MSA and its subtypes after expanding the sample size**

| Group | Plasma measures | Clinical scales | β | Standard error | t | P | P (FDR-corrected) |
| --- | --- | --- | --- | --- | --- | --- | --- |
| MSA | NfL | UMSARS-I | 0.339109418 | 0.105058528 | 3.227814298 | 0.002122752 | 0.03714816 |
| MSA | NfL | UMSARS-II | 0.21032581 | 0.107068804 | 1.964398602 | 0.054453808 | - |
| MSA | NfL | UMSARS-III | 0.161396235 | 0.1154235 | 1.398296141 | 0.168321275 | - |
| MSA | NfL | UMSARS-IV | 0.398323015 | 0.105496817 | 3.775687505 | 0.000417799 | 0.014622965 |
| MSA | NfL | Total UMSARS | 0.287807074 | 0.105081329 | 2.738898307 | 0.008334218 | 0.072924408 |
| MSA | NfL | COMPASS-I | 0.229930298 | 0.111016914 | 2.071128532 | 0.042966782 | 0.21483391 |
| MSA | NfL | COMPASS-II | 0.009409598 | 0.114601163 | 0.082107351 | 0.934854121 | - |
| MSA | NfL | COMPASS-III | 0.170615776 | 0.11946726 | 1.428138357 | 0.158805647 | - |
| MSA | NfL | COMPASS-IV | 0.146932245 | 0.120464657 | 1.219712479 | 0.227686039 | - |
| MSA | NfL | COMPASS-V | 0.263052866 | 0.115669661 | 2.2741734 | 0.0268077 | 0.15637825 |
| MSA | NfL | COMPASS-VI | 0.003737196 | 0.12100179 | 0.030885458 | 0.975531486 | - |
| MSA | NfL | Total COMPASS | 0.282636832 | 0.110643888 | 2.554473058 | 0.013381438 | 0.093670066 |
| MSA | NfL | RBDSQ | 0.067701495 | 0.117904039 | 0.574208442 | 0.568350847 | - |
| MSA | NfL | ICARS | 0.08685693 | 0.558015423 | 0.155653278 | 0.877130254 | - |
| MSA | NfL | SARA | -0.024884664 | 0.565776382 | -0.043983214 | 0.9651481 | - |
| MSA | NfL | HAMA | -0.112420818 | 0.123326092 | -0.911573666 | 0.366850821 | - |
| MSA | NfL | HAMD | -0.14487807 | 0.123422348 | -1.173839848 | 0.246634463 | - |
| MSA | NfL | MMSE | 0.034866101 | 0.110533865 | 0.315433655 | 0.753412836 | - |
| MSA | NfL | MoCA | 0.064296735 | 0.107526853 | 0.597959799 | 0.551882695 | - |
| MSA | NfL | AVLT-learning | 0.020174909 | 0.393285997 | 0.051298316 | 0.959270355 | - |
| MSA | NfL | AVLT-recall | -0.248104089 | 0.40461402 | -0.613187079 | 0.542235194 | - |
| MSA | NfL | AVLT-recognition | -0.419480602 | 0.426423048 | -0.983719346 | 0.329989904 | - |
| MSA | NfL | ROCF-immediate | -0.050141874 | 0.376782684 | -0.133079029 | 0.894625569 | - |
| MSA | NfL | ROCF-delay | -0.013743415 | 0.337322885 | -0.040742612 | 0.967681323 | - |
| MSA | NfL | DST | -0.137067732 | 0.37984612 | -0.360850683 | 0.719545026 | - |
| MSA | NfL | SCWT-time | 0.024117125 | 0.384269311 | 0.062760996 | 0.950197453 | - |
| MSA | NfL | SCWT-number | 0.189517519 | 0.425270873 | 0.445639545 | 0.65770756 | - |
| MSA | NfL | VFT | 0.008298649 | 0.113300642 | 0.073244503 | 0.941859306 | - |
| MSA | NfL | BNT | -0.273256614 | 0.384466607 | -0.710742129 | 0.480142256 | - |
| MSA | NfL | TMT-A | 0.15390794 | 0.343495885 | 0.448063416 | 0.656305983 | - |
| MSA | NfL | TMT-B | 0.009517555 | 0.489445908 | 0.019445571 | 0.984567975 | - |
| MSA | NfL | ADL | 0.300025345 | 0.102700582 | 2.921359735 | 0.004958783 | 0.057852468 |
| MSA | NfL | MBI-C | -0.05901094 | 0.119529169 | -0.493694889 | 0.623452487 | - |
| MSA | NfL | NPI | -0.03434649 | 0.120658826 | -0.284657918 | 0.776995063 | - |
| MSA | NfL | ZBI | 0.019575332 | 0.119284488 | 0.164106268 | 0.870283705 | - |
|  |  |  |  |  |  |  |  |
|  |  |  |  |  |  |  |  |
| Group | Plasma measures | Clinical scales | β | Standard error | t | P | P (FDR-corrected) |
| MSA | GFAP | UMSARS-I | 0.20395758 | 0.121725646 | 1.675551422 | 0.099607898 | - |
| MSA | GFAP | UMSARS-II | 0.138284298 | 0.119154041 | 1.160550635 | 0.250748348 | - |
| MSA | GFAP | UMSARS-III | -0.029898223 | 0.130928842 | -0.228354754 | 0.820320775 | - |
| MSA | GFAP | UMSARS-IV | 0.411893226 | 0.117997152 | 3.490704815 | 0.001003204 | 0.03511214 |
| MSA | GFAP | Total UMSARS | 0.181147288 | 0.119500559 | 1.515869801 | 0.135384652 | - |
| MSA | GFAP | COMPASS-I | 0.054426047 | 0.12421112 | 0.438173706 | 0.662946005 | - |
| MSA | GFAP | COMPASS-II | -0.087491472 | 0.123241534 | -0.709918721 | 0.480700345 | - |
| MSA | GFAP | COMPASS-III | 0.066336467 | 0.131073084 | 0.506102893 | 0.614771079 | - |
| MSA | GFAP | COMPASS-IV | 0.045921275 | 0.131695091 | 0.348693901 | 0.728626735 | - |
| MSA | GFAP | COMPASS-V | 0.108686692 | 0.129773432 | 0.837511114 | 0.405865548 | - |
| MSA | GFAP | COMPASS-VI | -0.101754237 | 0.154830314 | -0.657198412 | 0.515233348 | - |
| MSA | GFAP | Total COMPASS | 0.075378168 | 0.125881797 | 0.598801179 | 0.551720128 | - |
| MSA | GFAP | RBDSQ | -0.008115757 | 0.130179049 | -0.062343036 | 0.950533351 | - |
| MSA | GFAP | ICARS | -0.10926589 | 0.195306921 | -0.559457337 | 0.579132478 | - |
| MSA | GFAP | SARA | -0.210231889 | 0.19583139 | -1.073535193 | 0.289801155 | - |
| MSA | GFAP | HAMA | 0.028430572 | 0.176292385 | 0.161269427 | 0.872603073 | - |
| MSA | GFAP | HAMD | -0.022189427 | 0.177500052 | -0.125010822 | 0.901071786 | - |
| MSA | GFAP | MMSE | 0.020816526 | 0.11641371 | 0.17881507 | 0.858622535 | - |
| MSA | GFAP | MoCA | -0.01267453 | 0.113480807 | -0.111688755 | 0.911404127 | - |
| MSA | GFAP | AVLT-learning | 0.020446919 | 0.131038576 | 0.156037399 | 0.876564684 | - |
| MSA | GFAP | AVLT-recall | -0.043610566 | 0.136696651 | -0.319031709 | 0.750889464 | - |
| MSA | GFAP | AVLT-recognition | 0.07316324 | 0.166552023 | 0.439281604 | 0.662350191 | - |
| MSA | GFAP | ROCF-immediate | 0.107300527 | 0.123715777 | 0.867314823 | 0.389607402 | - |
| MSA | GFAP | ROCF-delay | 0.199840871 | 0.111830445 | 1.786998802 | 0.080674652 | - |
| MSA | GFAP | DST | 0.076837033 | 0.127142496 | 0.604337932 | 0.548017048 | - |
| MSA | GFAP | SCWT-time | 0.002238829 | 0.133528188 | 0.016766715 | 0.986686891 | - |
| MSA | GFAP | SCWT-number | 0.0167488 | 0.148034146 | 0.113141466 | 0.910354077 | - |
| MSA | GFAP | VFT | -0.110845611 | 0.122736774 | -0.903116543 | 0.370136606 | - |
| MSA | GFAP | BNT | 0.005292469 | 0.12898289 | 0.041032337 | 0.967413507 | - |
| MSA | GFAP | TMT-A | -0.018733186 | 0.112928641 | -0.165885165 | 0.86900722 | - |
| MSA | GFAP | TMT-B | 0.137001403 | 0.131601877 | 1.041029248 | 0.303187654 | - |
| MSA | GFAP | ADL | 0.134772097 | 0.117530048 | 1.146703326 | 0.256212915 | - |
| MSA | GFAP | MBI-C | -0.018466361 | 0.1296375 | -0.142446138 | 0.887238897 | - |
| MSA | GFAP | NPI | -0.05731148 | 0.133478316 | -0.429369219 | 0.66936289 | - |
| MSA | GFAP | ZBI | -0.163587788 | 0.126705284 | -1.291088925 | 0.202383162 | - |
|  |  |  |  |  |  |  |  |
|  |  |  |  |  |  |  |  |
| Group | Plasma measures | Clinical scales | β | Standard error | t | P | P (FDR-corrected) |
| MSA | Aβ42 | UMSARS-I | 0.171378801 | 0.132162482 | 1.296728073 | 0.200238576 | - |
| MSA | Aβ42 | UMSARS-II | 0.119111008 | 0.127387469 | 0.935029242 | 0.353787857 | - |
| MSA | Aβ42 | UMSARS-III | 0.168702704 | 0.144906283 | 1.164219386 | 0.249972438 | - |
| MSA | Aβ42 | UMSARS-IV | 0.37600034 | 0.135983634 | 2.765041126 | 0.007903458 | 0.27662103 |
| MSA | Aβ42 | Total UMSARS | 0.162642443 | 0.129277999 | 1.258082922 | 0.213774655 | - |
| MSA | Aβ42 | COMPASS-I | 0.041004853 | 0.132217398 | 0.310132054 | 0.757611789 | - |
| MSA | Aβ42 | COMPASS-II | -0.001118949 | 0.131661831 | -0.008498656 | 0.993249339 | - |
| MSA | Aβ42 | COMPASS-III | 0.229870879 | 0.136302624 | 1.686474348 | 0.097268566 | - |
| MSA | Aβ42 | COMPASS-IV | 0.25952849 | 0.135859507 | 1.910271104 | 0.061224107 | - |
| MSA | Aβ42 | COMPASS-V | 0.219337181 | 0.135754025 | 1.615695603 | 0.111780714 | - |
| MSA | Aβ42 | COMPASS-VI | 0.222007029 | 0.154906007 | 1.433172496 | 0.160436617 | - |
| MSA | Aβ42 | Total COMPASS | 0.150182003 | 0.132801562 | 1.130875272 | 0.262927611 | - |
| MSA | Aβ42 | RBDSQ | 0.065003727 | 0.144251378 | 0.450628115 | 0.654167614 | - |
| MSA | Aβ42 | ICARS | -0.275603298 | 0.184258723 | -1.495740846 | 0.142980841 | - |
| MSA | Aβ42 | SARA | -0.322148451 | 0.184944298 | -1.741867441 | 0.089621924 | - |
| MSA | Aβ42 | HAMA | -0.185807488 | 0.154536646 | -1.202352277 | 0.23551582 | - |
| MSA | Aβ42 | HAMD | -0.177046066 | 0.155837493 | -1.136094165 | 0.26193153 | - |
| MSA | Aβ42 | MMSE | 0.067119577 | 0.113626543 | 0.590703323 | 0.55670663 | - |
| MSA | Aβ42 | MoCA | 0.051518574 | 0.110857226 | 0.464729059 | 0.643632261 | - |
| MSA | Aβ42 | AVLT-learning | 0.100327987 | 0.137935127 | 0.727356323 | 0.470037757 | - |
| MSA | Aβ42 | AVLT-recall | -0.129611419 | 0.14478455 | -0.895202005 | 0.374510911 | - |
| MSA | Aβ42 | AVLT-recognition | 0.007929043 | 0.153103948 | 0.051788624 | 0.958903472 | - |
| MSA | Aβ42 | ROCF-immediate | 0.006291793 | 0.136206337 | 0.046193102 | 0.96332675 | - |
| MSA | Aβ42 | ROCF-delay | 0.166575253 | 0.12158006 | 1.37008694 | 0.177457025 | - |
| MSA | Aβ42 | DST | 0.04557693 | 0.133727238 | 0.340820096 | 0.734492071 | - |
| MSA | Aβ42 | SCWT-time | -0.142038007 | 0.139144603 | -1.020794224 | 0.312077747 | - |
| MSA | Aβ42 | SCWT-number | 0.058343627 | 0.155607199 | 0.374941693 | 0.709228749 | - |
| MSA | Aβ42 | VFT | 0.072164664 | 0.128406512 | 0.562001591 | 0.576243863 | - |
| MSA | Aβ42 | BNT | 0.032520606 | 0.139104741 | 0.233785029 | 0.815989501 | - |
| MSA | Aβ42 | TMT-A | -0.051797849 | 0.119907545 | -0.431981568 | 0.667864435 | - |
| MSA | Aβ42 | TMT-B | -0.09721162 | 0.153094359 | -0.634978461 | 0.528521734 | - |
| MSA | Aβ42 | ADL | 0.125415097 | 0.121225243 | 1.034562555 | 0.3051677 | - |
| MSA | Aβ42 | MBI-C | 0.002248049 | 0.136370192 | 0.016484903 | 0.986906139 | - |
| MSA | Aβ42 | NPI | 0.017953635 | 0.137352314 | 0.130712286 | 0.896488659 | - |
| MSA | Aβ42 | ZBI | -0.015460296 | 0.141893331 | -0.108957172 | 0.913655834 | - |
|  |  |  |  |  |  |  |  |
|  |  |  |  |  |  |  |  |
| Group | Plasma measures | Clinical scales | β | Standard error | t | P | P (FDR-corrected) |
| MSA | Aβ40 | UMSARS-I | 0.200348431 | 0.127949252 | 1.565842926 | 0.123227347 | - |
| MSA | Aβ40 | UMSARS-II | 0.114845988 | 0.123216892 | 0.932063667 | 0.355304798 | - |
| MSA | Aβ40 | UMSARS-III | 0.171024853 | 0.13714296 | 1.247055284 | 0.218305171 | - |
| MSA | Aβ40 | UMSARS-IV | 0.311393152 | 0.131512134 | 2.367790285 | 0.0217246 | 0.579769593 |
| MSA | Aβ40 | Total UMSARS | 0.17895766 | 0.12551227 | 1.425818048 | 0.159676325 | - |
| MSA | Aβ40 | COMPASS-I | -0.014137934 | 0.130486813 | -0.108347609 | 0.914107244 | - |
| MSA | Aβ40 | COMPASS-II | -0.06536397 | 0.129546634 | -0.504559386 | 0.615848034 | - |
| MSA | Aβ40 | COMPASS-III | 0.149569148 | 0.136331159 | 1.097101714 | 0.277292211 | - |
| MSA | Aβ40 | COMPASS-IV | 0.213423383 | 0.135303584 | 1.577366813 | 0.120343976 | - |
| MSA | Aβ40 | COMPASS-V | 0.287202024 | 0.131473929 | 2.184478897 | 0.033129691 | 0.579769593 |
| MSA | Aβ40 | COMPASS-VI | 0.125019342 | 0.165098898 | 0.757239108 | 0.453834653 | - |
| MSA | Aβ40 | Total COMPASS | 0.088611801 | 0.131921265 | 0.671702181 | 0.504535004 | - |
| MSA | Aβ40 | RBDSQ | 0.138568577 | 0.135956264 | 1.019214358 | 0.312911935 | - |
| MSA | Aβ40 | ICARS | -0.0924581 | 0.24114852 | -0.383407289 | 0.703554511 | - |
| MSA | Aβ40 | SARA | -0.196775038 | 0.242813849 | -0.810394625 | 0.422759767 | - |
| MSA | Aβ40 | HAMA | -0.16380922 | 0.170191177 | -0.962501252 | 0.340941676 | - |
| MSA | Aβ40 | HAMD | -0.210594914 | 0.170221226 | -1.237183632 | 0.222436258 | - |
| MSA | Aβ40 | MMSE | 0.018426162 | 0.115190707 | 0.159962231 | 0.873392107 | - |
| MSA | Aβ40 | MoCA | 0.035048642 | 0.112212052 | 0.312342937 | 0.755749956 | - |
| MSA | Aβ40 | AVLT-learning | 0.100552404 | 0.130984271 | 0.767667777 | 0.445909468 | - |
| MSA | Aβ40 | AVLT-recall | -0.058396104 | 0.13982036 | -0.417650931 | 0.677799395 | - |
| MSA | Aβ40 | AVLT-recognition | 0.080032816 | 0.145051512 | 0.551754443 | 0.583574347 | - |
| MSA | Aβ40 | ROCF-immediate | 0.07616941 | 0.128506225 | 0.592729346 | 0.555836488 | - |
| MSA | Aβ40 | ROCF-delay | 0.150546702 | 0.114443484 | 1.315467658 | 0.195016058 | - |
| MSA | Aβ40 | DST | 0.046982916 | 0.128666847 | 0.365151683 | 0.716349644 | - |
| MSA | Aβ40 | SCWT-time | -0.078337551 | 0.133136722 | -0.588399278 | 0.558811475 | - |
| MSA | Aβ40 | SCWT-number | -0.02242871 | 0.14807586 | -0.151467703 | 0.880192896 | - |
| MSA | Aβ40 | VFT | 0.117051407 | 0.122932674 | 0.95215863 | 0.344901115 | - |
| MSA | Aβ40 | BNT | 0.04778048 | 0.133317377 | 0.358396486 | 0.721370605 | - |
| MSA | Aβ40 | TMT-A | -0.039064603 | 0.114185064 | -0.342116575 | 0.733891967 | - |
| MSA | Aβ40 | TMT-B | 0.156837428 | 0.139555693 | 1.123833966 | 0.266790936 | - |
| MSA | Aβ40 | ADL | 0.128163535 | 0.119123516 | 1.075887782 | 0.28643256 | - |
| MSA | Aβ40 | MBI-C | 6.77493E-05 | 0.133084354 | 0.000509071 | 0.99959563 | - |
| MSA | Aβ40 | NPI | -0.045620531 | 0.135101488 | -0.337676008 | 0.736916535 | - |
| MSA | Aβ40 | ZBI | 0.01512772 | 0.140097079 | 0.10798027 | 0.914426914 | - |
|  |  |  |  |  |  |  |  |
|  |  |  |  |  |  |  |  |
| Group | Plasma measures | Clinical scales | β | Standard error | t | P | P (FDR-corrected) |
| MSA | P-tau181 | UMSARS-I | 0.043366811 | 0.12801164 | 0.338772401 | 0.736095008 | - |
| MSA | P-tau181 | UMSARS-II | 0.032327792 | 0.121152921 | 0.266834605 | 0.790576675 | - |
| MSA | P-tau181 | UMSARS-III | 0.030906858 | 0.146493336 | 0.21097791 | 0.833779484 | - |
| MSA | P-tau181 | UMSARS-IV | 0.092292433 | 0.137550036 | 0.670973527 | 0.505264999 | - |
| MSA | P-tau181 | Total UMSARS | 0.03388418 | 0.125154648 | 0.270738485 | 0.787624353 | - |
| MSA | P-tau181 | COMPASS-I | -0.007571129 | 0.129548667 | -0.058442352 | 0.953604329 | - |
| MSA | P-tau181 | COMPASS-II | -0.143635692 | 0.127460577 | -1.126902884 | 0.264589256 | - |
| MSA | P-tau181 | COMPASS-III | -0.148412817 | 0.135342307 | -1.096573722 | 0.277521055 | - |
| MSA | P-tau181 | COMPASS-IV | 0.01622328 | 0.137255223 | 0.118197903 | 0.906333804 | - |
| MSA | P-tau181 | COMPASS-V | 0.238925653 | 0.13216448 | 1.807790212 | 0.076010738 | - |
| MSA | P-tau181 | COMPASS-VI | 0.094997264 | 0.163188072 | 0.582133627 | 0.564104876 | - |
| MSA | P-tau181 | Total COMPASS | 0.001163514 | 0.131489499 | 0.00884872 | 0.992971283 | - |
| MSA | P-tau181 | RBDSQ | 0.039110074 | 0.136831392 | 0.28582676 | 0.776168574 | - |
| MSA | P-tau181 | ICARS | 0.308712187 | 0.154677676 | 1.995841899 | 0.053155045 | - |
| MSA | P-tau181 | SARA | 0.361428222 | 0.154012908 | 2.346739817 | 0.024252788 | 0.760170985 |
| MSA | P-tau181 | HAMA | 0.197320768 | 0.163138528 | 1.209528923 | 0.23277587 | - |
| MSA | P-tau181 | HAMD | 0.007252398 | 0.166881991 | 0.043458241 | 0.96552852 | - |
| MSA | P-tau181 | MMSE | -0.063077143 | 0.114720677 | -0.549832378 | 0.584262621 | - |
| MSA | P-tau181 | MoCA | -0.04470184 | 0.111932992 | -0.399362503 | 0.690896311 | - |
| MSA | P-tau181 | AVLT-learning | 0.192684008 | 0.126625347 | 1.521685931 | 0.133714838 | - |
| MSA | P-tau181 | AVLT-recall | 0.153142818 | 0.131380879 | 1.165640085 | 0.248700837 | - |
| MSA | P-tau181 | AVLT-recognition | 0.03828214 | 0.152852793 | 0.250451036 | 0.803264852 | - |
| MSA | P-tau181 | ROCF-immediate | -0.090855245 | 0.120177963 | -0.756005863 | 0.452931136 | - |
| MSA | P-tau181 | ROCF-delay | -0.084616351 | 0.113099215 | -0.74816038 | 0.458255853 | - |
| MSA | P-tau181 | DST | 0.04206341 | 0.124225923 | 0.338604124 | 0.73615214 | - |
| MSA | P-tau181 | SCWT-time | 0.191533002 | 0.124008428 | 1.544516003 | 0.12852798 | - |
| MSA | P-tau181 | SCWT-number | 0.127821194 | 0.139493556 | 0.916323288 | 0.36372755 | - |
| MSA | P-tau181 | VFT | 0.046471105 | 0.127227894 | 0.365258777 | 0.71622443 | - |
| MSA | P-tau181 | BNT | -0.00123153 | 0.123002447 | -0.010012239 | 0.992046483 | - |
| MSA | P-tau181 | TMT-A | 0.108985206 | 0.113190519 | 0.962847485 | 0.340886069 | - |
| MSA | P-tau181 | TMT-B | 0.216525911 | 0.123823028 | 1.748672401 | 0.086876684 | - |
| MSA | P-tau181 | ADL | -0.034510492 | 0.116556872 | -0.296082855 | 0.768224363 | - |
| MSA | P-tau181 | MBI-C | -0.038309762 | 0.137568378 | -0.278477963 | 0.781671204 | - |
| MSA | P-tau181 | NPI | -0.146303911 | 0.142751052 | -1.024888502 | 0.309985177 | - |
| MSA | P-tau181 | ZBI | -0.017181747 | 0.144895224 | -0.118580492 | 0.906064621 | - |
|  |  |  |  |  |  |  |  |
|  |  |  |  |  |  |  |  |
| Group | Plasma measures | Clinical scales | β | Standard error | t | P | P (FDR-corrected) |
| MSA | Aβ42/40 | UMSARS-I | -0.113972744 | 0.136588445 | -0.834424496 | 0.407718109 | - |
| MSA | Aβ42/40 | UMSARS-II | -0.026078417 | 0.131317841 | -0.198590054 | 0.84330266 | - |
| MSA | Aβ42/40 | UMSARS-III | 0.010116416 | 0.151170059 | 0.066920766 | 0.946917097 | - |
| MSA | Aβ42/40 | UMSARS-IV | 0.018431873 | 0.144159154 | 0.127857801 | 0.898764342 | - |
| MSA | Aβ42/40 | Total UMSARS | -0.080003065 | 0.133904839 | -0.597462091 | 0.552696169 | - |
| MSA | Aβ42/40 | COMPASS-I | 0.101823882 | 0.130292074 | 0.781504805 | 0.437797301 | - |
| MSA | Aβ42/40 | COMPASS-II | 0.208156379 | 0.127335632 | 1.634706448 | 0.107720263 | - |
| MSA | Aβ42/40 | COMPASS-III | 0.170527041 | 0.136426527 | 1.249955155 | 0.216514433 | - |
| MSA | Aβ42/40 | COMPASS-IV | 0.093022077 | 0.138248934 | 0.672859272 | 0.503804075 | - |
| MSA | Aβ42/40 | COMPASS-V | -0.105212505 | 0.136765236 | -0.769292754 | 0.444952267 | - |
| MSA | Aβ42/40 | COMPASS-VI | 0.193687093 | 0.186986689 | 1.03583359 | 0.307187927 | - |
| MSA | Aβ42/40 | Total COMPASS | 0.123463905 | 0.131931667 | 0.935817064 | 0.35338558 | - |
| MSA | Aβ42/40 | RBDSQ | -0.15687301 | 0.141216113 | -1.11087189 | 0.271833428 | - |
| MSA | Aβ42/40 | ICARS | -0.26812997 | 0.158502931 | -1.691640452 | 0.098900986 | - |
| MSA | Aβ42/40 | SARA | -0.223380752 | 0.162611166 | -1.37371103 | 0.177581628 | - |
| MSA | Aβ42/40 | HAMA | -0.040869268 | 0.149167675 | -0.273982067 | 0.785352163 | - |
| MSA | Aβ42/40 | HAMD | -0.008233244 | 0.150292405 | -0.054781503 | 0.956555034 | - |
| MSA | Aβ42/40 | MMSE | 0.137468474 | 0.116805118 | 1.176904545 | 0.243397835 | - |
| MSA | Aβ42/40 | MoCA | 0.068297756 | 0.114713494 | 0.595376826 | 0.553597387 | - |
| MSA | Aβ42/40 | AVLT-learning | -0.005553021 | 0.130162195 | -0.042662315 | 0.966122474 | - |
| MSA | Aβ42/40 | AVLT-recall | -0.106470258 | 0.135384243 | -0.786430204 | 0.434930882 | - |
| MSA | Aβ42/40 | AVLT-recognition | -0.171793546 | 0.144742746 | -1.186888814 | 0.240880157 | - |
| MSA | Aβ42/40 | ROCF-immediate | -0.03698078 | 0.122908484 | -0.300880612 | 0.764661006 | - |
| MSA | Aβ42/40 | ROCF-delay | -0.033537935 | 0.112946705 | -0.296935928 | 0.767881762 | - |
| MSA | Aβ42/40 | DST | 0.062437005 | 0.125715945 | 0.496651443 | 0.621344943 | - |
| MSA | Aβ42/40 | SCWT-time | -0.066659736 | 0.126096969 | -0.528638686 | 0.599305327 | - |
| MSA | Aβ42/40 | SCWT-number | 0.048465051 | 0.140026446 | 0.346113556 | 0.7306539 | - |
| MSA | Aβ42/40 | VFT | -0.055761976 | 0.128546057 | -0.43378986 | 0.666023139 | - |
| MSA | Aβ42/40 | BNT | 0.008906495 | 0.125434848 | 0.071004947 | 0.943642364 | - |
| MSA | Aβ42/40 | TMT-A | -0.0092276 | 0.119195238 | -0.077415841 | 0.938643713 | - |
| MSA | Aβ42/40 | TMT-B | -0.333802741 | 0.125254271 | -2.665000874 | 0.010517664 | 0.36811824 |
| MSA | Aβ42/40 | ADL | 0.007300724 | 0.130067807 | 0.056130137 | 0.955431053 | - |
| MSA | Aβ42/40 | MBI-C | -0.008565434 | 0.137110291 | -0.062471127 | 0.950410098 | - |
| MSA | Aβ42/40 | NPI | 0.143333494 | 0.144668794 | 0.990769957 | 0.326216824 | - |
| MSA | Aβ42/40 | ZBI | 0.00708537 | 0.137215695 | 0.051636735 | 0.959016001 | - |
|  |  |  |  |  |  |  |  |
|  |  |  |  |  |  |  |  |
| Group | Plasma measures | Clinical scales | β | Standard error | t | P | P (FDR-corrected) |
| MSA-C | NfL | UMSARS-I | 0.370138765 | 0.117272476 | 3.156228801 | 0.002954529 | 0.034469505 |
| MSA-C | NfL | UMSARS-II | 0.227715018 | 0.121622304 | 1.872312982 | 0.06797386 | - |
| MSA-C | NfL | UMSARS-III | 0.156552324 | 0.132238487 | 1.183863542 | 0.243819936 | - |
| MSA-C | NfL | UMSARS-IV | 0.426101523 | 0.119849736 | 3.555297957 | 0.00098759 | 0.022147773 |
| MSA-C | NfL | Total UMSARS | 0.320293846 | 0.117832125 | 2.718221747 | 0.009497667 | 0.0743127 |
| MSA-C | NfL | COMPASS-I | 0.269990324 | 0.120856356 | 2.233977041 | 0.03061495 | 0.15307475 |
| MSA-C | NfL | COMPASS-II | 0.0130418 | 0.124191536 | 0.105013599 | 0.916842286 | - |
| MSA-C | NfL | COMPASS-III | 0.204691393 | 0.130409757 | 1.569601829 | 0.123672123 | - |
| MSA-C | NfL | COMPASS-IV | 0.170025769 | 0.1335542 | 1.273084402 | 0.209673523 | - |
| MSA-C | NfL | COMPASS-V | 0.309983695 | 0.130411891 | 2.376958828 | 0.021870259 | 0.127576511 |
| MSA-C | NfL | COMPASS-VI | -0.030517539 | 0.132324399 | -0.230626698 | 0.819343418 | - |
| MSA-C | NfL | Total COMPASS | 0.3219818 | 0.120640835 | 2.668928813 | 0.0106161 | 0.0743127 |
| MSA-C | NfL | RBDSQ | 0.062119768 | 0.13080432 | 0.474906086 | 0.637436761 | - |
| MSA-C | NfL | ICARS | -0.362099637 | 0.691212324 | -0.523861662 | 0.604355693 | - |
| MSA-C | NfL | SARA | -0.582717914 | 0.696760901 | -0.836324073 | 0.409811185 | - |
| MSA-C | NfL | HAMA | -0.130535552 | 0.142213746 | -0.917882803 | 0.365547032 | - |
| MSA-C | NfL | HAMD | -0.167403451 | 0.140244953 | -1.193650447 | 0.241389061 | - |
| MSA-C | NfL | MMSE | 0.041837569 | 0.131082006 | 0.319170955 | 0.750900543 | - |
| MSA-C | NfL | MoCA | 0.073772411 | 0.127680838 | 0.577787643 | 0.565949921 | - |
| MSA-C | NfL | AVLT-learning | 0.122847747 | 0.472735929 | 0.259865477 | 0.796207799 | - |
| MSA-C | NfL | AVLT-recall | -0.055198389 | 0.49847683 | -0.110734112 | 0.912342438 | - |
| MSA-C | NfL | AVLT-recognition | -0.643444146 | 0.505451396 | -1.27300894 | 0.210748132 | - |
| MSA-C | NfL | ROCF-immediate | -0.160739086 | 0.469592357 | -0.342294937 | 0.733877505 | - |
| MSA-C | NfL | ROCF-delay | -0.11610169 | 0.400428942 | -0.289943304 | 0.773620594 | - |
| MSA-C | NfL | DST | -0.275624518 | 0.46786583 | -0.589110169 | 0.558800052 | - |
| MSA-C | NfL | SCWT-time | -0.17315894 | 0.45762449 | -0.378386523 | 0.707144328 | - |
| MSA-C | NfL | SCWT-number | 0.188607844 | 0.520132034 | 0.36261532 | 0.718801307 | - |
| MSA-C | NfL | VFT | 0.013574951 | 0.13035079 | 0.104141685 | 0.917500051 | - |
| MSA-C | NfL | BNT | -0.40617898 | 0.485723273 | -0.836235368 | 0.40754107 | - |
| MSA-C | NfL | TMT-A | 0.083883584 | 0.416150351 | 0.201570379 | 0.841419573 | - |
| MSA-C | NfL | TMT-B | 0.076729456 | 0.604672997 | 0.126894133 | 0.8997113 | - |
| MSA-C | NfL | ADL | 0.385176493 | 0.111970338 | 3.439986864 | 0.001265587 | 0.022147773 |
| MSA-C | NfL | MBI-C | -0.066430305 | 0.134531656 | -0.493789395 | 0.623910953 | - |
| MSA-C | NfL | NPI | -0.037858402 | 0.136593898 | -0.277160274 | 0.783016654 | - |
| MSA-C | NfL | ZBI | 0.045885441 | 0.139348619 | 0.32928522 | 0.743702056 | - |
|  |  |  |  |  |  |  |  |
|  |  |  |  |  |  |  |  |
| Group | Plasma measures | Clinical scales | β | Standard error | t | P | P (FDR-corrected) |
| MSA-C | GFAP | UMSARS-I | 0.250411777 | 0.136166636 | 1.839009792 | 0.072990723 | - |
| MSA-C | GFAP | UMSARS-II | 0.187333648 | 0.134482917 | 1.392992148 | 0.170782486 | - |
| MSA-C | GFAP | UMSARS-III | -0.047448314 | 0.147864222 | -0.32089111 | 0.750051096 | - |
| MSA-C | GFAP | UMSARS-IV | 0.471726758 | 0.129782285 | 3.634754609 | 0.000784583 | 0.027460405 |
| MSA-C | GFAP | Total UMSARS | 0.234492012 | 0.133847934 | 1.751928517 | 0.087085387 | - |
| MSA-C | GFAP | COMPASS-I | 0.048217889 | 0.137095013 | 0.351711476 | 0.726733489 | - |
| MSA-C | GFAP | COMPASS-II | -0.157123071 | 0.131599533 | -1.193948551 | 0.238895454 | - |
| MSA-C | GFAP | COMPASS-III | 0.078936982 | 0.143776995 | 0.549023725 | 0.585764757 | - |
| MSA-C | GFAP | COMPASS-IV | 0.056746577 | 0.146150791 | 0.388274167 | 0.699685494 | - |
| MSA-C | GFAP | COMPASS-V | 0.135340229 | 0.147733445 | 0.916110964 | 0.364602618 | - |
| MSA-C | GFAP | COMPASS-VI | -0.163966358 | 0.166480913 | -0.984895839 | 0.333419348 | - |
| MSA-C | GFAP | Total COMPASS | 0.070678233 | 0.139587606 | 0.506336023 | 0.615148609 | - |
| MSA-C | GFAP | RBDSQ | -0.010044307 | 0.142803373 | -0.070336624 | 0.944276214 | - |
| MSA-C | GFAP | ICARS | -0.08840509 | 0.22495255 | -0.392994389 | 0.697197748 | - |
| MSA-C | GFAP | SARA | -0.19947347 | 0.225990268 | -0.88266398 | 0.384676253 | - |
| MSA-C | GFAP | HAMA | 0.085264597 | 0.210995731 | 0.404105789 | 0.688823089 | - |
| MSA-C | GFAP | HAMD | -0.014213419 | 0.210431114 | -0.067544285 | 0.946568684 | - |
| MSA-C | GFAP | MMSE | -0.06631481 | 0.137899359 | -0.480892807 | 0.63264939 | - |
| MSA-C | GFAP | MoCA | -0.103366257 | 0.134152128 | -0.770515223 | 0.444549176 | - |
| MSA-C | GFAP | AVLT-learning | -0.049641149 | 0.148035285 | -0.335333222 | 0.73900429 | - |
| MSA-C | GFAP | AVLT-recall | -0.061089041 | 0.157491232 | -0.387888522 | 0.700012155 | - |
| MSA-C | GFAP | AVLT-recognition | 0.028995794 | 0.187761322 | 0.154429006 | 0.878088784 | - |
| MSA-C | GFAP | ROCF-immediate | 0.005041065 | 0.14651404 | 0.034406701 | 0.972719806 | - |
| MSA-C | GFAP | ROCF-delay | 0.154806617 | 0.125985658 | 1.228763817 | 0.227597632 | - |
| MSA-C | GFAP | DST | 0.06783699 | 0.147645628 | 0.45945817 | 0.648167267 | - |
| MSA-C | GFAP | SCWT-time | 0.020890882 | 0.148753041 | 0.140440032 | 0.88901717 | - |
| MSA-C | GFAP | SCWT-number | -0.014701389 | 0.169072501 | -0.086953165 | 0.931142577 | - |
| MSA-C | GFAP | VFT | -0.16055307 | 0.139368422 | -1.152004647 | 0.255145413 | - |
| MSA-C | GFAP | BNT | -0.082275209 | 0.153612114 | -0.535603647 | 0.59492965 | - |
| MSA-C | GFAP | TMT-A | -0.045510514 | 0.12740743 | -0.357204555 | 0.723083564 | - |
| MSA-C | GFAP | TMT-B | 0.166855848 | 0.147298554 | 1.132773157 | 0.264596965 | - |
| MSA-C | GFAP | ADL | 0.241736475 | 0.131720858 | 1.835217898 | 0.073087923 | - |
| MSA-C | GFAP | MBI-C | -0.045021661 | 0.145544054 | -0.309333564 | 0.758527959 | - |
| MSA-C | GFAP | NPI | -0.08141876 | 0.150673703 | -0.540364768 | 0.591799659 | - |
| MSA-C | GFAP | ZBI | -0.137591905 | 0.150169767 | -0.916242382 | 0.365170895 | - |
|  |  |  |  |  |  |  |  |
|  |  |  |  |  |  |  |  |
| Group | Plasma measures | Clinical scales | β | Standard error | t | P | P (FDR-corrected) |
| MSA-C | Aβ42 | UMSARS-I | 0.215158627 | 0.149899114 | 1.435356234 | 0.158591433 | - |
| MSA-C | Aβ42 | UMSARS-II | 0.170852365 | 0.14541648 | 1.174917483 | 0.246493046 | - |
| MSA-C | Aβ42 | UMSARS-III | 0.176719158 | 0.168614674 | 1.048065117 | 0.30122919 | - |
| MSA-C | Aβ42 | UMSARS-IV | 0.496005957 | 0.150314929 | 3.299778415 | 0.002040075 | 0.071402625 |
| MSA-C | Aβ42 | Total UMSARS | 0.216314706 | 0.146645759 | 1.475083275 | 0.147647727 | - |
| MSA-C | Aβ42 | COMPASS-I | 0.064179968 | 0.148840563 | 0.431199445 | 0.668428679 | - |
| MSA-C | Aβ42 | COMPASS-II | -0.066139102 | 0.144929991 | -0.456352076 | 0.650381545 | - |
| MSA-C | Aβ42 | COMPASS-III | 0.229424549 | 0.152875478 | 1.500728256 | 0.140568284 | - |
| MSA-C | Aβ42 | COMPASS-IV | 0.284468353 | 0.153165323 | 1.857263429 | 0.069974901 | - |
| MSA-C | Aβ42 | COMPASS-V | 0.377694082 | 0.151692583 | 2.489865182 | 0.016629957 | 0.291024248 |
| MSA-C | Aβ42 | COMPASS-VI | 0.145414462 | 0.173276462 | 0.83920493 | 0.408723474 | - |
| MSA-C | Aβ42 | Total COMPASS | 0.182104619 | 0.149596524 | 1.21730515 | 0.229976792 | - |
| MSA-C | Aβ42 | RBDSQ | 0.094128801 | 0.161069516 | 0.584398606 | 0.56223394 | - |
| MSA-C | Aβ42 | ICARS | -0.31564028 | 0.212231865 | -1.487242641 | 0.147743885 | - |
| MSA-C | Aβ42 | SARA | -0.338739559 | 0.214517361 | -1.579077601 | 0.125165368 | - |
| MSA-C | Aβ42 | HAMA | -0.303996717 | 0.181850773 | -1.671682295 | 0.104339316 | - |
| MSA-C | Aβ42 | HAMD | -0.323432494 | 0.179777419 | -1.799071856 | 0.081444968 | - |
| MSA-C | Aβ42 | MMSE | 0.041302411 | 0.137648086 | 0.30005801 | 0.765352566 | - |
| MSA-C | Aβ42 | MoCA | -0.019101557 | 0.134472481 | -0.142048074 | 0.887601704 | - |
| MSA-C | Aβ42 | AVLT-learning | 0.165838308 | 0.161723333 | 1.025444535 | 0.310887808 | - |
| MSA-C | Aβ42 | AVLT-recall | -0.051975117 | 0.175210045 | -0.296644618 | 0.768166016 | - |
| MSA-C | Aβ42 | AVLT-recognition | 0.072319947 | 0.178451794 | 0.405263212 | 0.687556697 | - |
| MSA-C | Aβ42 | ROCF-immediate | -0.221527247 | 0.166961175 | -1.326818923 | 0.191911196 | - |
| MSA-C | Aβ42 | ROCF-delay | 0.014362616 | 0.146345887 | 0.098141579 | 0.922396184 | - |
| MSA-C | Aβ42 | DST | 0.044830426 | 0.160781065 | 0.278829017 | 0.781682384 | - |
| MSA-C | Aβ42 | SCWT-time | -0.171554148 | 0.161157132 | -1.064514773 | 0.293479611 | - |
| MSA-C | Aβ42 | SCWT-number | -0.029210636 | 0.185661784 | -0.157332518 | 0.875774307 | - |
| MSA-C | Aβ42 | VFT | 0.187526458 | 0.145028132 | 1.293035046 | 0.20231885 | - |
| MSA-C | Aβ42 | BNT | -0.068230003 | 0.174113323 | -0.391871239 | 0.69704498 | - |
| MSA-C | Aβ42 | TMT-A | -0.098002754 | 0.142468109 | -0.68789257 | 0.496054387 | - |
| MSA-C | Aβ42 | TMT-B | -0.091404838 | 0.182513103 | -0.500812467 | 0.619469457 | - |
| MSA-C | Aβ42 | ADL | 0.156689905 | 0.141228832 | 1.10947533 | 0.273119195 | - |
| MSA-C | Aβ42 | MBI-C | 0.038579303 | 0.156682101 | 0.246226614 | 0.806651891 | - |
| MSA-C | Aβ42 | NPI | -0.001958272 | 0.159187105 | -0.012301697 | 0.990243163 | - |
| MSA-C | Aβ42 | ZBI | -0.008609731 | 0.177025697 | -0.048635488 | 0.96145801 | - |
|  |  |  |  |  |  |  |  |
|  |  |  |  |  |  |  |  |
| Group | Plasma measures | Clinical scales | β | Standard error | t | P | P (FDR-corrected) |
| MSA-C | Aβ40 | UMSARS-I | 0.272513788 | 0.146137737 | 1.864773566 | 0.069210782 | - |
| MSA-C | Aβ40 | UMSARS-II | 0.208824405 | 0.142645197 | 1.463942768 | 0.150482328 | - |
| MSA-C | Aβ40 | UMSARS-III | 0.190219047 | 0.159654919 | 1.191438683 | 0.240869961 | - |
| MSA-C | Aβ40 | UMSARS-IV | 0.425965135 | 0.149728748 | 2.844912151 | 0.006970377 | 0.121981598 |
| MSA-C | Aβ40 | Total UMSARS | 0.268666724 | 0.143076941 | 1.87777795 | 0.067366941 | - |
| MSA-C | Aβ40 | COMPASS-I | 0.00746028 | 0.14599595 | 0.051099223 | 0.959477733 | - |
| MSA-C | Aβ40 | COMPASS-II | -0.084868841 | 0.141623482 | -0.599256845 | 0.552074916 | - |
| MSA-C | Aβ40 | COMPASS-III | 0.203123606 | 0.150337375 | 1.351118488 | 0.183564443 | - |
| MSA-C | Aβ40 | COMPASS-IV | 0.292625604 | 0.14931132 | 1.959835359 | 0.056367889 | - |
| MSA-C | Aβ40 | COMPASS-V | 0.424036821 | 0.145146614 | 2.921437921 | 0.00548013 | 0.121981598 |
| MSA-C | Aβ40 | COMPASS-VI | 0.105816078 | 0.180494492 | 0.586256549 | 0.562572695 | - |
| MSA-C | Aβ40 | Total COMPASS | 0.136466946 | 0.147449777 | 0.925514763 | 0.359746778 | - |
| MSA-C | Aβ40 | RBDSQ | 0.171984558 | 0.154259056 | 1.114907364 | 0.27154406 | - |
| MSA-C | Aβ40 | ICARS | -0.087158591 | 0.278503475 | -0.312953336 | 0.756555734 | - |
| MSA-C | Aβ40 | SARA | -0.174440361 | 0.281388389 | -0.619927361 | 0.540146578 | - |
| MSA-C | Aβ40 | HAMA | -0.195969803 | 0.201688887 | -0.971644032 | 0.338515335 | - |
| MSA-C | Aβ40 | HAMD | -0.267089924 | 0.198039997 | -1.34866657 | 0.186912021 | - |
| MSA-C | Aβ40 | MMSE | -0.066458027 | 0.139772447 | -0.475473016 | 0.636480109 | - |
| MSA-C | Aβ40 | MoCA | -0.07291455 | 0.136374883 | -0.534662604 | 0.595206601 | - |
| MSA-C | Aβ40 | AVLT-learning | 0.110238793 | 0.148894363 | 0.740382578 | 0.463091617 | - |
| MSA-C | Aβ40 | AVLT-recall | -0.030863851 | 0.162219913 | -0.190259326 | 0.850001304 | - |
| MSA-C | Aβ40 | AVLT-recognition | 0.083523569 | 0.164250962 | 0.508511902 | 0.614034115 | - |
| MSA-C | Aβ40 | ROCF-immediate | -0.051071319 | 0.153965088 | -0.331707142 | 0.741800109 | - |
| MSA-C | Aβ40 | ROCF-delay | 0.055837564 | 0.13237822 | 0.421803256 | 0.675823769 | - |
| MSA-C | Aβ40 | DST | 0.054894871 | 0.149880697 | 0.366257112 | 0.715928504 | - |
| MSA-C | Aβ40 | SCWT-time | -0.073380448 | 0.15019339 | -0.488573084 | 0.627811956 | - |
| MSA-C | Aβ40 | SCWT-number | -0.06511448 | 0.170882318 | -0.381048667 | 0.705183575 | - |
| MSA-C | Aβ40 | VFT | 0.132644315 | 0.142394253 | 0.931528573 | 0.356340751 | - |
| MSA-C | Aβ40 | BNT | -0.087571414 | 0.160761478 | -0.544728847 | 0.588690452 | - |
| MSA-C | Aβ40 | TMT-A | -0.061448918 | 0.130978077 | -0.469154225 | 0.641868861 | - |
| MSA-C | Aβ40 | TMT-B | 0.194372723 | 0.159062772 | 1.221987525 | 0.229440572 | - |
| MSA-C | Aβ40 | ADL | 0.253247619 | 0.13383685 | 1.892211437 | 0.064907228 | - |
| MSA-C | Aβ40 | MBI-C | -0.018789715 | 0.151865666 | -0.123725889 | 0.902095793 | - |
| MSA-C | Aβ40 | NPI | -0.064361042 | 0.154997915 | -0.415238116 | 0.680081307 | - |
| MSA-C | Aβ40 | ZBI | 0.140947925 | 0.167847309 | 0.83973896 | 0.406173203 | - |
|  |  |  |  |  |  |  |  |
|  |  |  |  |  |  |  |  |
| Group | Plasma measures | Clinical scales | β | Standard error | t | P | P (FDR-corrected) |
| MSA-C | P-tau181 | UMSARS-I | -0.038717064 | 0.148574019 | -0.260591081 | 0.795681312 | - |
| MSA-C | P-tau181 | UMSARS-II | -0.059423234 | 0.143121164 | -0.415195294 | 0.68006343 | - |
| MSA-C | P-tau181 | UMSARS-III | 0.117932358 | 0.177735958 | 0.663525601 | 0.510999653 | - |
| MSA-C | P-tau181 | UMSARS-IV | 0.202797977 | 0.168231225 | 1.205471678 | 0.235104224 | - |
| MSA-C | P-tau181 | Total UMSARS | -0.058699286 | 0.145375736 | -0.403776361 | 0.688425739 | - |
| MSA-C | P-tau181 | COMPASS-I | -0.087230608 | 0.151143787 | -0.57713658 | 0.566788646 | - |
| MSA-C | P-tau181 | COMPASS-II | -0.282883347 | 0.141478245 | -1.999483012 | 0.051754535 | - |
| MSA-C | P-tau181 | COMPASS-III | -0.070488771 | 0.159074918 | -0.443116813 | 0.659852353 | - |
| MSA-C | P-tau181 | COMPASS-IV | 0.041624689 | 0.16166412 | 0.257476363 | 0.798011429 | - |
| MSA-C | P-tau181 | COMPASS-V | 0.119481014 | 0.16382075 | 0.729339932 | 0.46965862 | - |
| MSA-C | P-tau181 | COMPASS-VI | 0.321217446 | 0.189879414 | 1.691691787 | 0.102216775 | - |
| MSA-C | P-tau181 | Total COMPASS | -0.068649664 | 0.154358711 | -0.444741111 | 0.658686968 | - |
| MSA-C | P-tau181 | RBDSQ | -0.006058835 | 0.163389961 | -0.037082053 | 0.970604032 | - |
| MSA-C | P-tau181 | ICARS | 0.287800016 | 0.223838405 | 1.285749046 | 0.208703066 | - |
| MSA-C | P-tau181 | SARA | 0.298496112 | 0.226985274 | 1.315046153 | 0.198798615 | - |
| MSA-C | P-tau181 | HAMA | 0.378292166 | 0.217533315 | 1.739007959 | 0.09164679 | - |
| MSA-C | P-tau181 | HAMD | 0.095438154 | 0.225781003 | 0.422702321 | 0.675339503 | - |
| MSA-C | P-tau181 | MMSE | -0.110615532 | 0.139566497 | -0.792565082 | 0.431702758 | - |
| MSA-C | P-tau181 | MoCA | -0.088479462 | 0.136528915 | -0.648063904 | 0.519850755 | - |
| MSA-C | P-tau181 | AVLT-learning | -0.09316806 | 0.154385371 | -0.603477256 | 0.549361577 | - |
| MSA-C | P-tau181 | AVLT-recall | -0.086027785 | 0.16144698 | -0.532854717 | 0.596877401 | - |
| MSA-C | P-tau181 | AVLT-recognition | -0.099986306 | 0.187825379 | -0.532336508 | 0.597592085 | - |
| MSA-C | P-tau181 | ROCF-immediate | -0.153353046 | 0.147727115 | -1.038083264 | 0.30531493 | - |
| MSA-C | P-tau181 | ROCF-delay | -0.033164489 | 0.139087944 | -0.238442585 | 0.81296914 | - |
| MSA-C | P-tau181 | DST | 0.038681146 | 0.152798669 | 0.253151064 | 0.801330547 | - |
| MSA-C | P-tau181 | SCWT-time | 0.269577841 | 0.144777861 | 1.862010115 | 0.069959653 | - |
| MSA-C | P-tau181 | SCWT-number | 0.133622512 | 0.170205339 | 0.785066514 | 0.437039918 | - |
| MSA-C | P-tau181 | VFT | -0.066609758 | 0.151916176 | -0.438463893 | 0.663058913 | - |
| MSA-C | P-tau181 | BNT | -0.072501294 | 0.153385739 | -0.472672975 | 0.638782801 | - |
| MSA-C | P-tau181 | TMT-A | 0.142685541 | 0.137168319 | 1.040222276 | 0.305369459 | - |
| MSA-C | P-tau181 | TMT-B | 0.355165121 | 0.139439689 | 2.54708773 | 0.015153346 | 0.53036711 |
| MSA-C | P-tau181 | ADL | 0.004557959 | 0.136921745 | 0.033288787 | 0.973591514 | - |
| MSA-C | P-tau181 | MBI-C | -0.176190999 | 0.161764658 | -1.089181044 | 0.282003716 | - |
| MSA-C | P-tau181 | NPI | -0.172525298 | 0.172779564 | -0.998528384 | 0.323741587 | - |
| MSA-C | P-tau181 | ZBI | -0.01175424 | 0.180223914 | -0.065220202 | 0.948331854 | - |
|  |  |  |  |  |  |  |  |
|  |  |  |  |  |  |  |  |
| Group | Plasma measures | Clinical scales | β | Standard error | t | P | P (FDR-corrected) |
| MSA-C | Aβ42/40 | UMSARS-I | -0.168818601 | 0.152904595 | -1.104078008 | 0.275844797 | - |
| MSA-C | Aβ42/40 | UMSARS-II | -0.101259906 | 0.14978803 | -0.676021347 | 0.502647035 | - |
| MSA-C | Aβ42/40 | UMSARS-III | -0.014975835 | 0.171937315 | -0.087100554 | 0.931048955 | - |
| MSA-C | Aβ42/40 | UMSARS-IV | -0.000621018 | 0.163607632 | -0.003795775 | 0.996990285 | - |
| MSA-C | Aβ42/40 | Total UMSARS | -0.147870014 | 0.150217241 | -0.984374449 | 0.330568398 | - |
| MSA-C | Aβ42/40 | COMPASS-I | 0.099509045 | 0.149022639 | 0.667744481 | 0.50778421 | - |
| MSA-C | Aβ42/40 | COMPASS-II | 0.179256352 | 0.143352552 | 1.250458041 | 0.217741594 | - |
| MSA-C | Aβ42/40 | COMPASS-III | 0.0697551 | 0.157040178 | 0.44418633 | 0.65908491 | - |
| MSA-C | Aβ42/40 | COMPASS-IV | -0.037077944 | 0.159620319 | -0.232288375 | 0.817391107 | - |
| MSA-C | Aβ42/40 | COMPASS-V | -0.156540258 | 0.160981103 | -0.972413878 | 0.336160385 | - |
| MSA-C | Aβ42/40 | COMPASS-VI | 0.04614144 | 0.249185535 | 0.185169013 | 0.854480438 | - |
| MSA-C | Aβ42/40 | Total COMPASS | 0.074209632 | 0.15231776 | 0.487202756 | 0.628533182 | - |
| MSA-C | Aβ42/40 | RBDSQ | -0.161673513 | 0.153984731 | -1.049932108 | 0.300051619 | - |
| MSA-C | Aβ42/40 | ICARS | -0.285779141 | 0.178375098 | -1.602124648 | 0.119966193 | - |
| MSA-C | Aβ42/40 | SARA | -0.214011263 | 0.184726067 | -1.158533098 | 0.256098332 | - |
| MSA-C | Aβ42/40 | HAMA | -0.121402815 | 0.187239108 | -0.648383854 | 0.521362627 | - |
| MSA-C | Aβ42/40 | HAMD | -0.105409757 | 0.186568044 | -0.564993633 | 0.576017976 | - |
| MSA-C | Aβ42/40 | MMSE | 0.24152233 | 0.141116801 | 1.711506555 | 0.093063658 | - |
| MSA-C | Aβ42/40 | MoCA | 0.126095859 | 0.140563413 | 0.897074542 | 0.373894426 | - |
| MSA-C | Aβ42/40 | AVLT-learning | 0.00275516 | 0.155761386 | 0.01768834 | 0.985969304 | - |
| MSA-C | Aβ42/40 | AVLT-recall | -0.078273912 | 0.165678706 | -0.472444007 | 0.638999003 | - |
| MSA-C | Aβ42/40 | AVLT-recognition | -0.159757311 | 0.172739113 | -0.924847353 | 0.360881514 | - |
| MSA-C | Aβ42/40 | ROCF-immediate | -0.147367203 | 0.150282968 | -0.980598165 | 0.332542112 | - |
| MSA-C | Aβ42/40 | ROCF-delay | -0.125043664 | 0.131750678 | -0.94909314 | 0.349271048 | - |
| MSA-C | Aβ42/40 | DST | 0.036844841 | 0.153496938 | 0.240036326 | 0.8114169 | - |
| MSA-C | Aβ42/40 | SCWT-time | -0.081803875 | 0.147109499 | -0.556074733 | 0.581257273 | - |
| MSA-C | Aβ42/40 | SCWT-number | -0.027926945 | 0.167765886 | -0.166463788 | 0.868630599 | - |
| MSA-C | Aβ42/40 | VFT | 0.094789192 | 0.151793614 | 0.624461001 | 0.535344611 | - |
| MSA-C | Aβ42/40 | BNT | 0.075949065 | 0.156200958 | 0.486226628 | 0.629219478 | - |
| MSA-C | Aβ42/40 | TMT-A | -0.028451862 | 0.136851787 | -0.207902741 | 0.836509965 | - |
| MSA-C | Aβ42/40 | TMT-B | -0.373839625 | 0.139950671 | -2.671224235 | 0.011167314 | 0.39085599 |
| MSA-C | Aβ42/40 | ADL | -0.185504483 | 0.157316119 | -1.179182937 | 0.244522429 | - |
| MSA-C | Aβ42/40 | MBI-C | 0.110731287 | 0.159291054 | 0.695150692 | 0.490615942 | - |
| MSA-C | Aβ42/40 | NPI | 0.1783261 | 0.17237645 | 1.034515445 | 0.306815382 | - |
| MSA-C | Aβ42/40 | ZBI | -0.121308133 | 0.16730926 | -0.725053311 | 0.472747462 | - |
|  |  |  |  |  |  |  |  |
|  |  |  |  |  |  |  |  |
| Group | Plasma measures | Clinical scales | β | Standard error | t | P | P (FDR-corrected) |
| MSA-P | NfL | UMSARS-I | 1.063065877 | 0.261560071 | 4.06432783 | 0.003611701 | 0.025281907 |
| MSA-P | NfL | UMSARS-II | 1.197786429 | 0.168379133 | 7.113627497 | 5.58322E-05 | 0.001953 |
| MSA-P | NfL | UMSARS-III | 0.678788698 | 0.412614285 | 1.645092578 | 0.143947882 | - |
| MSA-P | NfL | UMSARS-IV | 1.186115978 | 0.31889945 | 3.719404276 | 0.00746192 | 0.038567675 |
| MSA-P | NfL | Total UMSARS | 1.142168159 | 0.195362284 | 5.84641077 | 0.000384338 | 0.006725915 |
| MSA-P | NfL | COMPASS-I | 0.30501233 | 0.728441168 | 0.418719236 | 0.686436729 | - |
| MSA-P | NfL | COMPASS-II | 0.96311945 | 0.581560534 | 1.656094927 | 0.136294754 | - |
| MSA-P | NfL | COMPASS-III | -0.3987253 | 0.655932069 | -0.607875904 | 0.560126132 | - |
| MSA-P | NfL | COMPASS-IV | 0.262533185 | 0.681095326 | 0.385457328 | 0.709945562 | - |
| MSA-P | NfL | COMPASS-V | 1.41680944 | 0.340678631 | 4.158785762 | 0.003170495 | 0.025281907 |
| MSA-P | NfL | COMPASS-VI | 1.47400641 | 0.398200615 | 3.701667841 | 0.013975187 | 0.061141443 |
| MSA-P | NfL | Total COMPASS | 0.565583099 | 0.70282649 | 0.804726497 | 0.444232981 | - |
| MSA-P | NfL | RBDSQ | 0.412170587 | 0.732844614 | 0.562425621 | 0.591373373 | - |
| MSA-P | NfL | ICARS | 1.361113003 | 0.180546008 | 7.538870648 | 0.000650426 | 0.007588303 |
| MSA-P | NfL | SARA | 1.287777394 | 0.299464908 | 4.300261428 | 0.007713535 | 0.038567675 |
| MSA-P | NfL | HAMA | 0.809519269 | 0.366951125 | 2.206068365 | 0.054795555 | - |
| MSA-P | NfL | HAMD | 0.653608445 | 0.427998618 | 1.527127466 | 0.161075778 | - |
| MSA-P | NfL | MMSE | -0.14666208 | 0.212788384 | -0.689239126 | 0.50378246 | - |
| MSA-P | NfL | MoCA | -0.224341646 | 0.160169765 | -1.400649156 | 0.18664334 | - |
| MSA-P | NfL | AVLT-learning | 0.338522233 | 0.33297705 | 1.016653349 | 0.33586828 | - |
| MSA-P | NfL | AVLT-recall | 0.099736664 | 0.346296789 | 0.288009209 | 0.779862062 | - |
| MSA-P | NfL | AVLT-recognition | 0.200574106 | 0.4853502 | 0.413256461 | 0.690273741 | - |
| MSA-P | NfL | ROCF-immediate | -0.232592818 | 0.267375026 | -0.869912278 | 0.406950725 | - |
| MSA-P | NfL | ROCF-delay | -0.330583441 | 0.333069637 | -0.992535506 | 0.353998343 | - |
| MSA-P | NfL | DST | 0.168940039 | 0.400747285 | 0.421562529 | 0.683234532 | - |
| MSA-P | NfL | SCWT-time | 0.820420101 | 0.425882101 | 1.926401931 | 0.090220174 | - |
| MSA-P | NfL | SCWT-number | 0.343436041 | 0.505332668 | 0.679623668 | 0.515921954 | - |
| MSA-P | NfL | VFT | -0.411139709 | 0.393209346 | -1.045600044 | 0.326307203 | - |
| MSA-P | NfL | BNT | -0.111693276 | 0.244258442 | -0.457274986 | 0.658311148 | - |
| MSA-P | NfL | TMT-A | 0.869630217 | 0.556083362 | 1.563848653 | 0.178620067 | - |
| MSA-P | NfL | TMT-B | 0.693241265 | 0.723344887 | 0.958382753 | 0.374878728 | - |
| MSA-P | NfL | ADL | 0.744051863 | 0.297934168 | 2.497370038 | 0.034008264 | 0.13225436 |
| MSA-P | NfL | MBI-C | 0.343844986 | 0.649215276 | 0.529631693 | 0.61074579 | - |
| MSA-P | NfL | NPI | -0.293504128 | 0.700565704 | -0.418953035 | 0.686272725 | - |
| MSA-P | NfL | ZBI | 0.674141659 | 0.378393782 | 1.781587571 | 0.108503827 | - |
|  |  |  |  |  |  |  |  |
|  |  |  |  |  |  |  |  |
| Group | Plasma measures | Clinical scales | β | Standard error | t | P | P (FDR-corrected) |
| MSA-P | GFAP | UMSARS-I | 0.258709946 | 0.391625745 | 0.660605051 | 0.527425926 | - |
| MSA-P | GFAP | UMSARS-II | 0.320364332 | 0.367670822 | 0.871334664 | 0.406213958 | - |
| MSA-P | GFAP | UMSARS-III | 0.051996011 | 0.441174466 | 0.117858159 | 0.909490624 | - |
| MSA-P | GFAP | UMSARS-IV | -0.014363768 | 0.356557216 | -0.040284608 | 0.968991126 | - |
| MSA-P | GFAP | Total UMSARS | 0.290357433 | 0.380376824 | 0.763341546 | 0.467171251 | - |
| MSA-P | GFAP | COMPASS-I | 0.53481012 | 0.380987286 | 1.403747944 | 0.198000665 | - |
| MSA-P | GFAP | COMPASS-II | 0.845246078 | 0.24941903 | 3.388859617 | 0.009516007 | 0.333060245 |
| MSA-P | GFAP | COMPASS-III | -0.057715901 | 0.386975195 | -0.149146256 | 0.885129487 | - |
| MSA-P | GFAP | COMPASS-IV | 0.084030446 | 0.395919266 | 0.212241367 | 0.837226591 | - |
| MSA-P | GFAP | COMPASS-V | 0.246266098 | 0.338893739 | 0.726676446 | 0.488139141 | - |
| MSA-P | GFAP | COMPASS-VI | -0.017081645 | 0.479914722 | -0.035593084 | 0.972984099 | - |
| MSA-P | GFAP | Total COMPASS | 0.566269082 | 0.371551878 | 1.524064646 | 0.165997843 | - |
| MSA-P | GFAP | RBDSQ | -0.029214918 | 0.485496184 | -0.060175382 | 0.953697918 | - |
| MSA-P | GFAP | ICARS | 0.044286228 | 0.524535674 | 0.084429392 | 0.935991305 | - |
| MSA-P | GFAP | SARA | 0.049379201 | 0.536192877 | 0.092092237 | 0.930200695 | - |
| MSA-P | GFAP | HAMA | 0.246180097 | 0.403088882 | 0.610734029 | 0.556482758 | - |
| MSA-P | GFAP | HAMD | 0.471666428 | 0.404233255 | 1.166817481 | 0.273269241 | - |
| MSA-P | GFAP | MMSE | 0.007179681 | 0.225972759 | 0.031772332 | 0.975175953 | - |
| MSA-P | GFAP | MoCA | -0.112815965 | 0.176980568 | -0.637448318 | 0.535806159 | - |
| MSA-P | GFAP | AVLT-learning | 0.424864172 | 0.235482286 | 1.804229858 | 0.104687305 | - |
| MSA-P | GFAP | AVLT-recall | 0.228411709 | 0.261027918 | 0.875047049 | 0.404295424 | - |
| MSA-P | GFAP | AVLT-recognition | 0.035129979 | 0.39278753 | 0.089437613 | 0.930932698 | - |
| MSA-P | GFAP | ROCF-immediate | 0.227919587 | 0.20389046 | 1.117853118 | 0.292579589 | - |
| MSA-P | GFAP | ROCF-delay | 0.296125748 | 0.266883272 | 1.109570286 | 0.303841936 | - |
| MSA-P | GFAP | DST | -0.203949737 | 0.308903471 | -0.660237763 | 0.525636552 | - |
| MSA-P | GFAP | SCWT-time | 0.053174017 | 0.412404847 | 0.128936451 | 0.900590575 | - |
| MSA-P | GFAP | SCWT-number | 0.464228921 | 0.382675873 | 1.213112592 | 0.259687843 | - |
| MSA-P | GFAP | VFT | -0.13959148 | 0.333458317 | -0.41861748 | 0.686508114 | - |
| MSA-P | GFAP | BNT | -0.095141278 | 0.190492171 | -0.499449805 | 0.62944368 | - |
| MSA-P | GFAP | TMT-A | 0.779272858 | 0.574119008 | 1.357336801 | 0.232725769 | - |
| MSA-P | GFAP | TMT-B | 0.526446583 | 0.752527448 | 0.699571271 | 0.510392756 | - |
| MSA-P | GFAP | ADL | 0.30674217 | 0.285212991 | 1.075484569 | 0.310142571 | - |
| MSA-P | GFAP | MBI-C | 0.615605128 | 0.313322418 | 1.96476566 | 0.085029209 | - |
| MSA-P | GFAP | NPI | 0.510548653 | 0.367076373 | 1.390851309 | 0.201734055 | - |
| MSA-P | GFAP | ZBI | -0.095632638 | 0.348009206 | -0.27479916 | 0.789674559 | - |
|  |  |  |  |  |  |  |  |
|  |  |  |  |  |  |  |  |
| Group | Plasma measures | Clinical scales | β | Standard error | t | P | P (FDR-corrected) |
| MSA-P | Aβ42 | UMSARS-I | 0.066988505 | 0.344350237 | 0.194535962 | 0.850605585 | - |
| MSA-P | Aβ42 | UMSARS-II | 0.096918536 | 0.32339472 | 0.299691151 | 0.771218771 | - |
| MSA-P | Aβ42 | UMSARS-III | 0.14785529 | 0.372310363 | 0.39712913 | 0.703102418 | - |
| MSA-P | Aβ42 | UMSARS-IV | -0.208793061 | 0.307334428 | -0.679367627 | 0.518730497 | - |
| MSA-P | Aβ42 | Total UMSARS | 0.076589332 | 0.337057635 | 0.227229186 | 0.825944815 | - |
| MSA-P | Aβ42 | COMPASS-I | 0.103736358 | 0.3733839 | 0.277827614 | 0.788189675 | - |
| MSA-P | Aβ42 | COMPASS-II | 0.272182509 | 0.329594838 | 0.825809382 | 0.432847419 | - |
| MSA-P | Aβ42 | COMPASS-III | 0.142251024 | 0.338103941 | 0.420731636 | 0.685025662 | - |
| MSA-P | Aβ42 | COMPASS-IV | 0.182792857 | 0.34420671 | 0.531055472 | 0.609803216 | - |
| MSA-P | Aβ42 | COMPASS-V | -0.067463142 | 0.307722673 | -0.219233574 | 0.831958183 | - |
| MSA-P | Aβ42 | COMPASS-VI | 0.319011844 | 0.426179989 | 0.74853783 | 0.48783096 | - |
| MSA-P | Aβ42 | Total COMPASS | 0.165136304 | 0.367690222 | 0.449118017 | 0.665263744 | - |
| MSA-P | Aβ42 | RBDSQ | -0.212366089 | 0.424590238 | -0.500167149 | 0.632295294 | - |
| MSA-P | Aβ42 | ICARS | -0.070548538 | 0.481395552 | -0.146550041 | 0.889212713 | - |
| MSA-P | Aβ42 | SARA | -0.405799102 | 0.458615081 | -0.884835931 | 0.416760662 | - |
| MSA-P | Aβ42 | HAMA | 0.389632825 | 0.309655074 | 1.258280124 | 0.239952256 | - |
| MSA-P | Aβ42 | HAMD | 0.478764339 | 0.316047165 | 1.514850921 | 0.164111247 | - |
| MSA-P | Aβ42 | MMSE | -0.132705513 | 0.169564657 | -0.78262484 | 0.449016547 | - |
| MSA-P | Aβ42 | MoCA | -0.085463153 | 0.136213022 | -0.627422783 | 0.542136865 | - |
| MSA-P | Aβ42 | AVLT-learning | 0.076119185 | 0.225952863 | 0.336880819 | 0.743929417 | - |
| MSA-P | Aβ42 | AVLT-recall | -0.135558704 | 0.220403271 | -0.615048515 | 0.553753538 | - |
| MSA-P | Aβ42 | AVLT-recognition | -0.440713404 | 0.284072891 | -1.551409578 | 0.159402924 | - |
| MSA-P | Aβ42 | ROCF-immediate | 0.183636028 | 0.169315185 | 1.084580968 | 0.30630433 | - |
| MSA-P | Aβ42 | ROCF-delay | 0.454511355 | 0.167020374 | 2.721292886 | 0.029710041 | 0.915366028 |
| MSA-P | Aβ42 | DST | -0.09985461 | 0.259593333 | -0.384657837 | 0.709422653 | - |
| MSA-P | Aβ42 | SCWT-time | 0.053810397 | 0.339832755 | 0.158343763 | 0.878109995 | - |
| MSA-P | Aβ42 | SCWT-number | 0.477806665 | 0.298855883 | 1.598786212 | 0.148535458 | - |
| MSA-P | Aβ42 | VFT | -0.400801354 | 0.250612267 | -1.599288652 | 0.148423906 | - |
| MSA-P | Aβ42 | BNT | 0.027552979 | 0.159527739 | 0.172715911 | 0.866696465 | - |
| MSA-P | Aβ42 | TMT-A | 0.118475461 | 0.383457738 | 0.308966149 | 0.769806882 | - |
| MSA-P | Aβ42 | TMT-B | 0.151749193 | 0.446842271 | 0.339603486 | 0.745727261 | - |
| MSA-P | Aβ42 | ADL | 0.348795227 | 0.222116837 | 1.570323225 | 0.150787291 | - |
| MSA-P | Aβ42 | MBI-C | -0.015243303 | 0.336476892 | -0.045302675 | 0.964976476 | - |
| MSA-P | Aβ42 | NPI | 0.204459779 | 0.353512947 | 0.578365745 | 0.578930636 | - |
| MSA-P | Aβ42 | ZBI | 0.162552988 | 0.287826093 | 0.564761124 | 0.586035111 | - |
|  |  |  |  |  |  |  |  |
|  |  |  |  |  |  |  |  |
| Group | Plasma measures | Clinical scales | β | Standard error | t | P | P (FDR-corrected) |
| MSA-P | Aβ40 | UMSARS-I | 0.154515066 | 0.340834202 | 0.453343781 | 0.662345037 | - |
| MSA-P | Aβ40 | UMSARS-II | 0.054105083 | 0.323368209 | 0.167317261 | 0.870819551 | - |
| MSA-P | Aβ40 | UMSARS-III | 0.031769122 | 0.365506056 | 0.086918182 | 0.933170637 | - |
| MSA-P | Aβ40 | UMSARS-IV | -0.237256319 | 0.281047096 | -0.844187053 | 0.426470597 | - |
| MSA-P | Aβ40 | Total UMSARS | 0.109794221 | 0.335928513 | 0.326838051 | 0.752177737 | - |
| MSA-P | Aβ40 | COMPASS-I | 0.027558711 | 0.394867784 | 0.069792249 | 0.94607206 | - |
| MSA-P | Aβ40 | COMPASS-II | 0.277221687 | 0.347941452 | 0.79674809 | 0.448594575 | - |
| MSA-P | Aβ40 | COMPASS-III | -0.079377392 | 0.3587742 | -0.221246099 | 0.830443469 | - |
| MSA-P | Aβ40 | COMPASS-IV | -0.111990291 | 0.366578504 | -0.305501522 | 0.767782247 | - |
| MSA-P | Aβ40 | COMPASS-V | -0.340749585 | 0.301781903 | -1.129125309 | 0.291562913 | - |
| MSA-P | Aβ40 | COMPASS-VI | 0.037785221 | 0.608384768 | 0.06210744 | 0.952883544 | - |
| MSA-P | Aβ40 | Total COMPASS | -0.075857405 | 0.391032902 | -0.193992383 | 0.851017177 | - |
| MSA-P | Aβ40 | RBDSQ | 0.012943157 | 0.40171523 | 0.032219731 | 0.975196262 | - |
| MSA-P | Aβ40 | ICARS | 0.172113621 | 0.688181955 | 0.250099003 | 0.812461717 | - |
| MSA-P | Aβ40 | SARA | -0.297501193 | 0.695344049 | -0.42784747 | 0.686564017 | - |
| MSA-P | Aβ40 | HAMA | 0.712837758 | 0.398027913 | 1.790924042 | 0.106914717 | - |
| MSA-P | Aβ40 | HAMD | 0.740786063 | 0.421811049 | 1.756203554 | 0.112935527 | - |
| MSA-P | Aβ40 | MMSE | -0.364004973 | 0.164196534 | -2.216885852 | 0.046699137 | 0.658790574 |
| MSA-P | Aβ40 | MoCA | -0.215536366 | 0.142218932 | -1.515525133 | 0.155529214 | - |
| MSA-P | Aβ40 | AVLT-learning | -0.085187848 | 0.285906871 | -0.29795663 | 0.772500044 | - |
| MSA-P | Aβ40 | AVLT-recall | -0.371094737 | 0.255978094 | -1.449712868 | 0.181074207 | - |
| MSA-P | Aβ40 | AVLT-recognition | -0.621114363 | 0.334361116 | -1.857615413 | 0.10029723 | - |
| MSA-P | Aβ40 | ROCF-immediate | 0.026566742 | 0.227330574 | 0.116863922 | 0.909534189 | - |
| MSA-P | Aβ40 | ROCF-delay | 0.448581912 | 0.242479899 | 1.849975664 | 0.106770907 | - |
| MSA-P | Aβ40 | DST | -0.50885615 | 0.283902719 | -1.792360961 | 0.106672073 | - |
| MSA-P | Aβ40 | SCWT-time | 0.013123926 | 0.420204612 | 0.031232227 | 0.975849473 | - |
| MSA-P | Aβ40 | SCWT-number | 0.493031889 | 0.386345934 | 1.276141006 | 0.237705834 | - |
| MSA-P | Aβ40 | VFT | -0.295678952 | 0.280142159 | -1.055460389 | 0.322043976 | - |
| MSA-P | Aβ40 | BNT | 0.190420302 | 0.191680512 | 0.993425466 | 0.34645916 | - |
| MSA-P | Aβ40 | TMT-A | 0.060847454 | 0.389694521 | 0.156141415 | 0.882030013 | - |
| MSA-P | Aβ40 | TMT-B | 0.202222046 | 0.405511748 | 0.49868357 | 0.635753842 | - |
| MSA-P | Aβ40 | ADL | 0.282810143 | 0.302446386 | 0.93507529 | 0.374158237 | - |
| MSA-P | Aβ40 | MBI-C | 0.256036855 | 0.342526333 | 0.74749539 | 0.476159335 | - |
| MSA-P | Aβ40 | NPI | 0.245066072 | 0.3698644 | 0.662583564 | 0.526221898 | - |
| MSA-P | Aβ40 | ZBI | 0.137268627 | 0.33170318 | 0.413829698 | 0.688686408 | - |
|  |  |  |  |  |  |  |  |
|  |  |  |  |  |  |  |  |
| Group | Plasma measures | Clinical scales | β | Standard error | t | P | P (FDR-corrected) |
| MSA-P | P-tau181 | UMSARS-I | 0.528225595 | 0.255876768 | 2.064374971 | 0.072863116 | - |
| MSA-P | P-tau181 | UMSARS-II | 0.45313074 | 0.244763259 | 1.85130212 | 0.097149456 | - |
| MSA-P | P-tau181 | UMSARS-III | -0.278969864 | 0.33328627 | -0.837027772 | 0.430222802 | - |
| MSA-P | P-tau181 | UMSARS-IV | 0.022576813 | 0.27101195 | 0.083305601 | 0.935940776 | - |
| MSA-P | P-tau181 | Total UMSARS | 0.496395747 | 0.255947514 | 1.939443519 | 0.088422246 | - |
| MSA-P | P-tau181 | COMPASS-I | 0.199778639 | 0.342877147 | 0.582653704 | 0.576176303 | - |
| MSA-P | P-tau181 | COMPASS-II | 0.431592405 | 0.281709084 | 1.532050009 | 0.164047034 | - |
| MSA-P | P-tau181 | COMPASS-III | -0.094618749 | 0.31719267 | -0.298300554 | 0.773074654 | - |
| MSA-P | P-tau181 | COMPASS-IV | 0.23552867 | 0.315999387 | 0.745345341 | 0.477387557 | - |
| MSA-P | P-tau181 | COMPASS-V | 0.331865815 | 0.263008339 | 1.261807198 | 0.242562712 | - |
| MSA-P | P-tau181 | COMPASS-VI | 0.318634248 | 0.600061421 | 0.531002723 | 0.618148492 | - |
| MSA-P | P-tau181 | Total COMPASS | 0.293664714 | 0.331506887 | 0.885848002 | 0.401541775 | - |
| MSA-P | P-tau181 | RBDSQ | 0.535769114 | 0.308771232 | 1.735165258 | 0.12629583 | - |
| MSA-P | P-tau181 | ICARS | 0.521777621 | 0.23164135 | 2.252523661 | 0.074042259 | - |
| MSA-P | P-tau181 | SARA | 0.654016545 | 0.165679306 | 3.947484813 | 0.010878078 | 0.38073273 |
| MSA-P | P-tau181 | HAMA | 0.088784063 | 0.295980355 | 0.299966067 | 0.77101576 | - |
| MSA-P | P-tau181 | HAMD | 0.015751897 | 0.313591847 | 0.05023057 | 0.961035776 | - |
| MSA-P | P-tau181 | MMSE | -0.064364228 | 0.171333401 | -0.375666551 | 0.713721402 | - |
| MSA-P | P-tau181 | MoCA | -0.003055812 | 0.137231675 | -0.022267538 | 0.982600532 | - |
| MSA-P | P-tau181 | AVLT-learning | 0.430134015 | 0.16762018 | 2.56612309 | 0.030380407 | 0.42502887 |
| MSA-P | P-tau181 | AVLT-recall | 0.399640167 | 0.172894276 | 2.311471361 | 0.046125287 | 0.42502887 |
| MSA-P | P-tau181 | AVLT-recognition | 0.226032437 | 0.30473612 | 0.741731691 | 0.479456532 | - |
| MSA-P | P-tau181 | ROCF-immediate | -0.080915312 | 0.172563194 | -0.468902497 | 0.650289736 | - |
| MSA-P | P-tau181 | ROCF-delay | -0.254816457 | 0.229921174 | -1.108277468 | 0.304362977 | - |
| MSA-P | P-tau181 | DST | 0.007361254 | 0.253882942 | 0.028994678 | 0.977501608 | - |
| MSA-P | P-tau181 | SCWT-time | 0.024690722 | 0.330837641 | 0.074630933 | 0.942340791 | - |
| MSA-P | P-tau181 | SCWT-number | 0.062776039 | 0.333064316 | 0.18848023 | 0.855193654 | - |
| MSA-P | P-tau181 | VFT | 0.161282038 | 0.264462373 | 0.609848714 | 0.558881727 | - |
| MSA-P | P-tau181 | BNT | -0.026224478 | 0.154768473 | -0.169443285 | 0.869195353 | - |
| MSA-P | P-tau181 | TMT-A | -0.253368622 | 0.301406152 | -0.840621933 | 0.438897897 | - |
| MSA-P | P-tau181 | TMT-B | -0.626622305 | 0.26550019 | -2.360157651 | 0.056271959 | - |
| MSA-P | P-tau181 | ADL | 0.075298705 | 0.241912796 | 0.311263837 | 0.762689073 | - |
| MSA-P | P-tau181 | MBI-C | 0.337447334 | 0.2904546 | 1.161790291 | 0.278807578 | - |
| MSA-P | P-tau181 | NPI | -0.052073421 | 0.336180472 | -0.15489722 | 0.880739072 | - |
| MSA-P | P-tau181 | ZBI | 0.166730173 | 0.293246622 | 0.56856639 | 0.583556773 | - |
|  |  |  |  |  |  |  |  |
|  |  |  |  |  |  |  |  |
| Group | Plasma measures | Clinical scales | β | Standard error | t | P | P (FDR-corrected) |
| MSA-P | Aβ42/40 | UMSARS-I | -0.120766492 | 0.400660979 | -0.301418152 | 0.770781781 | - |
| MSA-P | Aβ42/40 | UMSARS-II | 0.076050093 | 0.33484864 | 0.22711782 | 0.825407194 | - |
| MSA-P | Aβ42/40 | UMSARS-III | 0.274646479 | 0.466566746 | 0.588654209 | 0.574588058 | - |
| MSA-P | Aβ42/40 | UMSARS-IV | -0.040078327 | 0.351604072 | -0.113987095 | 0.912448497 | - |
| MSA-P | Aβ42/40 | Total UMSARS | -0.034318711 | 0.394548064 | -0.086982333 | 0.932823318 | - |
| MSA-P | Aβ42/40 | COMPASS-I | 0.118225038 | 0.35374663 | 0.334208238 | 0.74681516 | - |
| MSA-P | Aβ42/40 | COMPASS-II | 0.108232414 | 0.323738185 | 0.334320816 | 0.746733361 | - |
| MSA-P | Aβ42/40 | COMPASS-III | 0.272264492 | 0.309933356 | 0.878461405 | 0.405303859 | - |
| MSA-P | Aβ42/40 | COMPASS-IV | 0.374128213 | 0.305068042 | 1.226376287 | 0.254926442 | - |
| MSA-P | Aβ42/40 | COMPASS-V | 0.212374226 | 0.283254125 | 0.749765695 | 0.474864668 | - |
| MSA-P | Aβ42/40 | COMPASS-VI | 0.273628228 | 0.337780547 | 0.81007693 | 0.454706471 | - |
| MSA-P | Aβ42/40 | Total COMPASS | 0.300721653 | 0.337099876 | 0.892084734 | 0.398384903 | - |
| MSA-P | Aβ42/40 | RBDSQ | -0.370633648 | 0.458334094 | -0.80865389 | 0.445323913 | - |
| MSA-P | Aβ42/40 | ICARS | -0.234172312 | 0.451090948 | -0.519124389 | 0.625825455 | - |
| MSA-P | Aβ42/40 | SARA | -0.449488754 | 0.428649776 | -1.048615397 | 0.342374049 | - |
| MSA-P | Aβ42/40 | HAMA | 0.084505485 | 0.276363162 | 0.30577695 | 0.766729071 | - |
| MSA-P | Aβ42/40 | HAMD | 0.161070976 | 0.287942284 | 0.559386324 | 0.589545417 | - |
| MSA-P | Aβ42/40 | MMSE | 0.127724749 | 0.163481261 | 0.781280669 | 0.449776983 | - |
| MSA-P | Aβ42/40 | MoCA | 0.074357296 | 0.131714143 | 0.5645354 | 0.582788676 | - |
| MSA-P | Aβ42/40 | AVLT-learning | 0.159605073 | 0.201149769 | 0.793463866 | 0.447920651 | - |
| MSA-P | Aβ42/40 | AVLT-recall | 0.056642215 | 0.205015976 | 0.276281959 | 0.788571137 | - |
| MSA-P | Aβ42/40 | AVLT-recognition | -0.192753749 | 0.302289982 | -0.637645178 | 0.541520504 | - |
| MSA-P | Aβ42/40 | ROCF-immediate | 0.227349284 | 0.146288904 | 1.554111615 | 0.154577965 | - |
| MSA-P | Aβ42/40 | ROCF-delay | 0.340282673 | 0.185238607 | 1.836996499 | 0.108823261 | - |
| MSA-P | Aβ42/40 | DST | 0.162734024 | 0.233271669 | 0.697615896 | 0.503039695 | - |
| MSA-P | Aβ42/40 | SCWT-time | 0.027815762 | 0.325379577 | 0.085487118 | 0.933974889 | - |
| MSA-P | Aβ42/40 | SCWT-number | 0.37925801 | 0.299703345 | 1.265444702 | 0.241322338 | - |
| MSA-P | Aβ42/40 | VFT | -0.244037377 | 0.262284436 | -0.930430264 | 0.379369122 | - |
| MSA-P | Aβ42/40 | BNT | -0.068257755 | 0.144442736 | -0.472559276 | 0.647776789 | - |
| MSA-P | Aβ42/40 | TMT-A | 0.075357599 | 0.35978262 | 0.209453139 | 0.842361094 | - |
| MSA-P | Aβ42/40 | TMT-B | -0.070845705 | 0.379312604 | -0.186773927 | 0.857993212 | - |
| MSA-P | Aβ42/40 | ADL | 0.263704526 | 0.211910362 | 1.244415439 | 0.244777279 | - |
| MSA-P | Aβ42/40 | MBI-C | -0.280000594 | 0.303778522 | -0.921726105 | 0.383626165 | - |
| MSA-P | Aβ42/40 | NPI | 0.021721703 | 0.342494473 | 0.063422055 | 0.95098647 | - |
| MSA-P | Aβ42/40 | ZBI | 0.106634458 | 0.28632868 | 0.372419758 | 0.71819895 | - |

Note: Results of multiple linear regressions were shown after accounting for age and sex.

Abbreviations: Αβ, amyloid-β; ADL, Activity of Daily Living Scale; AVLT: Auditory Verbal Learning Test; BNT: Boston Naming Test; COMPASS, Composite Autonomic Symptom Score; DST: Digit Span Test; FDR, false discovery rate; GFAP, glial fibrillary acidic protein; HAMA, Hamilton Anxiety Scale; HAMD, Hamilton Depression Scale; ICARS, International Cooperative Ataxia Rating Scale; MBI-C, Mild Behavioral Impairment Checklist; MMSE, Mini-Mental State Examination; MoCA, Montreal Cognitive Assessment; MSA, multiple system atrophy; MSA-C, multiple system atrophy-cerebellar type; MSA-P, multiple system atrophy-parkinsonian type; NfL, neurofilament light; NPI, Neuropsychiatric Inventory; p-tau, phosphorylated tau; RBDSQ, Rapid eye movement sleep behavior disorder screening questionnaire; ROCF: Rey-Osterreich Complex Figure; SARA, Scale for the Assessment and Rating of Ataxia; SCWT: Stroops Color Word Test; TMT: Trails Making Test; UMSARS, Unified Multiple System Atrophy Rating Scale; VFT, Verbal Fluency Test; ZBI, Zarit Caregiver Burden Interview.

**Supplementary Table 10: Associations of plasma measures with MRI indices in MSA patients after expanding the sample size**

| Plasma measure | Volume | β | Standard error | t | P | P value (FDR corrected) |
| --- | --- | --- | --- | --- | --- | --- |
| NfL | Medulla | 0.124129756 | 0.129835011 | 0.956057661 | 0.343383816 | - |
| NfL | Pons | 0.090573109 | 0.126249885 | 0.71741142 | 0.476270434 | - |
| NfL | Midbrain | 0.140481375 | 0.117451334 | 1.196081557 | 0.236988425 | - |
| NfL | Whole brainstem | 0.123124755 | 0.11969992 | 1.028611833 | 0.308333681 | - |
|  |  |  |  |  |  |  |
|  |  |  |  |  |  |  |
| Plasma measure | Volume | β | Standard error | t | P | P value (FDR corrected) |
| GFAP | Medulla | 0.085764165 | 0.128465692 | 0.667603648 | 0.5072833 | - |
| GFAP | Pons | -0.376487082 | 0.113775547 | -3.309033358 | 0.001687573 | 0.006750292 |
| GFAP | Midbrain | -0.160308136 | 0.115171829 | -1.391904061 | 0.169765321 | - |
| GFAP | Whole brainstem | -0.301830224 | 0.111643564 | -2.703516569 | 0.009200692 | 0.018401384 |
|  |  |  |  |  |  |  |
|  |  |  |  |  |  |  |
| Plasma measure | Volume | β | Standard error | t | P | P value (FDR corrected) |
| NfL | Left lateral ventricle | 0.113463453 | 0.130664117 | 0.868359695 | 0.389112613 | - |
| NfL | Left cerebellum white matter | -0.193131244 | 0.13489494 | -1.431716007 | 0.158096201 | - |
| NfL | Left cerebellum cortex | 0.046748854 | 0.124649045 | 0.375043823 | 0.709124551 | - |
| NfL | Left thalamus | -0.277575699 | 0.116691049 | -2.378723139 | 0.02100783 | 0.087696656 |
| NfL | Left caudate | -0.070417889 | 0.132643779 | -0.530879692 | 0.597719873 | - |
| NfL | Left putamen | -0.290055635 | 0.128442802 | -2.258247494 | 0.028071767 | 0.093572557 |
| NfL | Left pallidum | -0.348246464 | 0.122209999 | -2.849574237 | 0.006221582 | 0.06221582 |
| NfL | Left hippocampus | -0.242103608 | 0.102533554 | -2.361213456 | 0.021924164 | 0.087696656 |
| NfL | Left amygdala | -0.096794425 | 0.124305566 | -0.778681345 | 0.439630297 | - |
| NfL | Left accumbens | 0.188746853 | 0.137041644 | 1.377295602 | 0.174209991 | - |
| NfL | Right lateral ventricle | 0.116259977 | 0.129490084 | 0.89782919 | 0.373337236 | - |
| NfL | Right cerebellum white matter | -0.177483496 | 0.132547044 | -1.339022661 | 0.186278155 | - |
| NfL | Right cerebellum cortex | 0.121402108 | 0.127200358 | 0.954416399 | 0.344205882 | - |
| NfL | Right thalamus | -0.342711802 | 0.105132789 | -3.259799392 | 0.001950648 | 0.03901296 |
| NfL | Right caudate | -0.00390461 | 0.135592949 | -0.02879656 | 0.977135007 | - |
| NfL | Right putamen | -0.182204833 | 0.135619054 | -1.343504674 | 0.184832866 | - |
| NfL | Right pallidum | -0.316779505 | 0.126256916 | -2.509007146 | 0.015200047 | 0.087696656 |
| NfL | Right hippocampus | -0.199016622 | 0.107273774 | -1.855221591 | 0.069129949 | - |
| NfL | Right amygdala | 0.005264482 | 0.129592575 | 0.040623333 | 0.967748829 | - |
| NfL | Right accumbens | -0.006892816 | 0.139718065 | -0.049333746 | 0.960838776 | - |
|  |  |  |  |  |  |  |
|  |  |  |  |  |  |  |
| Plasma measure | Volume | β | Standard error | t | P | P value (FDR corrected) |
| GFAP | Left lateral ventricle | 0.13580716 | 0.128286522 | 1.058623762 | 0.294572223 | - |
| GFAP | Left cerebellum white matter | -0.277727098 | 0.129952968 | -2.137135466 | 0.037219897 | 0.09622223 |
| GFAP | Left cerebellum cortex | -0.220647639 | 0.119166543 | -1.851590501 | 0.069659057 | - |
| GFAP | Left thalamus | -0.096253873 | 0.120213782 | -0.80068917 | 0.426886395 | - |
| GFAP | Left caudate | -0.125678099 | 0.12987823 | -0.967661007 | 0.337608824 | - |
| GFAP | Left putamen | -0.280247436 | 0.126766825 | -2.210731682 | 0.031392198 | 0.09622223 |
| GFAP | Left pallidum | -0.396709413 | 0.11724434 | -3.383612501 | 0.001352113 | 0.02031837 |
| GFAP | Left hippocampus | -0.230455201 | 0.101362624 | -2.273571784 | 0.027069345 | 0.09622223 |
| GFAP | Left amygdala | 0.015977624 | 0.123138118 | 0.129753681 | 0.897252509 | - |
| GFAP | Left accumbens | 0.25672979 | 0.132798372 | 1.933229941 | 0.058557566 | - |
| GFAP | Right lateral ventricle | 0.083871201 | 0.128015781 | 0.655162984 | 0.515196504 | - |
| GFAP | Right cerebellum white matter | -0.393600345 | 0.121261926 | -3.245869156 | 0.002031837 | 0.02031837 |
| GFAP | Right cerebellum cortex | -0.257522632 | 0.121331947 | -2.122463526 | 0.038488892 | 0.09622223 |
| GFAP | Right thalamus | -0.254829988 | 0.107946549 | -2.360705268 | 0.021951285 | 0.09622223 |
| GFAP | Right caudate | -0.020695727 | 0.133549688 | -0.154966498 | 0.877436392 | - |
| GFAP | Right putamen | -0.233478863 | 0.132021119 | -1.768496322 | 0.082732831 | - |
| GFAP | Right pallidum | -0.352524033 | 0.122325558 | -2.881851018 | 0.005697147 | 0.03798098 |
| GFAP | Right hippocampus | -0.157974206 | 0.106877276 | -1.478089752 | 0.145302372 | - |
| GFAP | Right amygdala | 0.191872306 | 0.12491963 | 1.535966015 | 0.130495556 | - |
| GFAP | Right accumbens | 0.116718799 | 0.136709 | 0.853775534 | 0.397071826 | - |
|  |  |  |  |  |  |  |
|  |  |  |  |  |  |  |
| Plasma measure | Volume | β | Standard error | t | P | P value (FDR corrected) |
| NfL | Left bankssts | 0.010200968 | 0.118996538 | 0.085724918 | 0.932008122 | - |
| NfL | Left caudal anterior cingulate | 0.062931936 | 0.139348393 | 0.451615796 | 0.653388504 | - |
| NfL | Left caudal middle frontal | -0.214801893 | 0.128094355 | -1.676903668 | 0.099450611 | - |
| NfL | Left cuneus | -0.151121784 | 0.127427689 | -1.185941497 | 0.240935018 | - |
| NfL | Left entorhinal | -0.050003366 | 0.136780615 | -0.36557348 | 0.716138396 | - |
| NfL | Left fusiform | -0.119415605 | 0.11248432 | -1.061620014 | 0.293221948 | - |
| NfL | Left inferior parietal | 0.015299764 | 0.10934744 | 0.139918818 | 0.889254512 | - |
| NfL | Left inferior temporal | -0.081859386 | 0.113459712 | -0.721484165 | 0.473782624 | - |
| NfL | Left isthmus cingulate | 0.138684997 | 0.120125407 | 1.15450179 | 0.253473624 | - |
| NfL | Left lateral occipital | -0.210981858 | 0.114341146 | -1.845196294 | 0.070599106 | - |
| NfL | Left lateral orbitofrontal | -0.586226497 | 0.089308173 | -6.56408565 | 2.27E-08 | 7.72E-07 |
| NfL | Left lingual | -0.017846501 | 0.129841329 | -0.13744854 | 0.89119711 | - |
| NfL | Left medial orbitofrontal | 0.606974724 | 0.08649571 | 7.017396869 | 4.23E-09 | 2.88E-07 |
| NfL | Left middle temporal | 0.015670608 | 0.122936846 | 0.127468761 | 0.899051809 | - |
| NfL | Left parahippocampal | 0.227322176 | 0.133118732 | 1.707664825 | 0.0935507 | - |
| NfL | Left paracentral | -0.070677204 | 0.122717486 | -0.575934256 | 0.567097273 | - |
| NfL | Left pars opercularis | -0.143160138 | 0.128380226 | -1.115126084 | 0.269827342 | - |
| NfL | Left pars orbitalis | -0.069092616 | 0.115894188 | -0.596169808 | 0.553599593 | - |
| NfL | Left pars triangularis | -0.165390294 | 0.130091502 | -1.271338189 | 0.209159566 | - |
| NfL | Left pericalcarine | -0.058146472 | 0.134743808 | -0.431533534 | 0.667830841 | - |
| NfL | Left postcentral | -0.127253527 | 0.110104721 | -1.155749968 | 0.252967084 | - |
| NfL | Left posterior cingulate | 0.116080668 | 0.136357 | 0.851299663 | 0.39843298 | - |
| NfL | Left precentral | -0.191516231 | 0.095896403 | -1.997115904 | 0.05095843 | - |
| NfL | Left precuneus | -0.135588429 | 0.089849438 | -1.50906263 | 0.137221589 | - |
| NfL | Left rostral anterior cingulate | 0.042731834 | 0.112672238 | 0.379257877 | 0.706011665 | - |
| NfL | Left rostral middle frontal | -0.137330523 | 0.125093621 | -1.097821951 | 0.277244848 | - |
| NfL | Left superior frontal | -0.214321421 | 0.109215943 | -1.962363866 | 0.054979645 | - |
| NfL | Left superior parietal | -0.108699434 | 0.122560028 | -0.886907717 | 0.379135544 | - |
| NfL | Left superior temporal | -0.205417019 | 0.105877415 | -1.940140102 | 0.057691408 | - |
| NfL | Left supramarginal | -0.144005359 | 0.102836605 | -1.400331707 | 0.167241247 | - |
| NfL | Left frontal pole | 0.100462893 | 0.133074837 | 0.754935306 | 0.453629541 | - |
| NfL | Left temporal pole | 0.039596996 | 0.133070351 | 0.297564379 | 0.767198945 | - |
| NfL | Left transverse temporal | -0.268956067 | 0.120629703 | -2.229600681 | 0.030034226 | 0.323198315 |
| NfL | Left insula | -0.080216343 | 0.110032085 | -0.729026838 | 0.469194697 | - |
| NfL | Right bankssts | 0.059240128 | 0.120964656 | 0.489730886 | 0.626345355 | - |
| NfL | Right caudal anterior cingulate | -0.048839174 | 0.139653969 | -0.349715616 | 0.727937992 | - |
| NfL | Right caudal middle frontal | -0.254740116 | 0.126967714 | -2.006337732 | 0.049934857 | 0.323198315 |
| NfL | Right cuneus | -0.214939559 | 0.116782142 | -1.840517349 | 0.071293746 | - |
| NfL | Right entorhinal | -0.12252667 | 0.131895992 | -0.92896432 | 0.357119324 | - |
| NfL | Right fusiform | -0.022820684 | 0.119498929 | -0.19096978 | 0.849278946 | - |
| NfL | Right inferior parietal | -0.103133851 | 0.111704556 | -0.923273454 | 0.36004906 | - |
| NfL | Right inferior temporal | -0.019496195 | 0.104256335 | -0.187002499 | 0.852372695 | - |
| NfL | Right isthmus cingulate | 0.027198511 | 0.128311245 | 0.211972936 | 0.832941228 | - |
| NfL | Right lateral occipital | -0.229794471 | 0.116570457 | -1.971292528 | 0.053921341 | - |
| NfL | Right lateral orbitofrontal | -0.28629202 | 0.091055993 | -3.144131566 | 0.002728876 | 0.061854523 |
| NfL | Right lingual | -0.182564294 | 0.123798855 | -1.474684833 | 0.146213126 | - |
| NfL | Right medial orbitofrontal | 0.03349187 | 0.116828731 | 0.286674945 | 0.775478947 | - |
| NfL | Right middle temporal | 0.048862979 | 0.103143637 | 0.473737208 | 0.637633039 | - |
| NfL | Right parahippocampal | -0.000978926 | 0.138481654 | -0.007068991 | 0.99438635 | - |
| NfL | Right paracentral | -0.07610407 | 0.128408043 | -0.592673703 | 0.555919986 | - |
| NfL | Right pars opercularis | -0.128247992 | 0.134926826 | -0.950500331 | 0.346172557 | - |
| NfL | Right pars orbitalis | -0.266667925 | 0.123294652 | -2.162850714 | 0.035083955 | 0.323198315 |
| NfL | Right pars triangularis | -0.150230627 | 0.129870956 | -1.156768466 | 0.252554292 | - |
| NfL | Right pericalcarine | -0.221484115 | 0.12392189 | -1.787288067 | 0.079609624 | - |
| NfL | Right postcentral | -0.185083529 | 0.113997979 | -1.623568511 | 0.110401524 | - |
| NfL | Right posterior cingulate | 0.041888937 | 0.138978853 | 0.301405117 | 0.764285013 | - |
| NfL | Right precentral | 0.019980007 | 0.103100094 | 0.193792328 | 0.847079327 | - |
| NfL | Right precuneus | -0.03994327 | 0.099232919 | -0.40252036 | 0.68892005 | - |
| NfL | Right rostral anterior cingulate | 0.044989292 | 0.138114584 | 0.325738891 | 0.745904523 | - |
| NfL | Right rostral middle frontal | 0.027913729 | 0.121625427 | 0.229505702 | 0.819359303 | - |
| NfL | Right superior frontal | 0.007177023 | 0.104180335 | 0.068890379 | 0.945336137 | - |
| NfL | Right superior parietal | -0.008350023 | 0.106939358 | -0.07808185 | 0.938056992 | - |
| NfL | Right superior temporal | -0.071847068 | 0.119117592 | -0.603160854 | 0.548974263 | - |
| NfL | Right supramarginal | -0.14059364 | 0.097309036 | -1.444815879 | 0.154396219 | - |
| NfL | Right frontal pole | 0.326470408 | 0.131075611 | 2.490702938 | 0.015916976 | 0.270588592 |
| NfL | Right temporal pole | -0.160585212 | 0.129944148 | -1.235801798 | 0.221981738 | - |
| NfL | Right transverse temporal | -0.214677662 | 0.115868379 | -1.852771773 | 0.069486553 | - |
| NfL | Right insula | -0.207709094 | 0.102430371 | -2.027807688 | 0.047620269 | 0.323198315 |
|  |  |  |  |  |  |  |
|  |  |  |  |  |  |  |
| Plasma measure | Volume | β | Standard error | t | P | P value (FDR corrected) |
| GFAP | Left bankssts | -0.041242573 | 0.117100175 | -0.352199073 | 0.726085617 | - |
| GFAP | Left caudal anterior cingulate | 0.091559782 | 0.136966239 | 0.668484309 | 0.506725631 | - |
| GFAP | Left caudal middle frontal | -0.103248034 | 0.128717128 | -0.802131275 | 0.426059126 | - |
| GFAP | Left cuneus | 0.134608785 | 0.125838524 | 1.069694564 | 0.289604408 | - |
| GFAP | Left entorhinal | -0.100475276 | 0.134210952 | -0.748636938 | 0.457385648 | - |
| GFAP | Left fusiform | -0.300057406 | 0.104124908 | -2.881706331 | 0.005699403 | 0.193779702 |
| GFAP | Left inferior parietal | -0.02030868 | 0.107707003 | -0.18855487 | 0.85116185 | - |
| GFAP | Left inferior temporal | -0.149390828 | 0.110431601 | -1.352790559 | 0.181865677 | - |
| GFAP | Left isthmus cingulate | 0.097944789 | 0.119062208 | 0.822635412 | 0.414401276 | - |
| GFAP | Left lateral occipital | 0.007711589 | 0.116199736 | 0.066364949 | 0.947336989 | - |
| GFAP | Left lateral orbitofrontal | -0.3258576 | 0.109682684 | -2.970911974 | 0.004455172 | 0.193779702 |
| GFAP | Left lingual | 0.07413875 | 0.127529544 | 0.581345685 | 0.563471853 | - |
| GFAP | Left medial orbitofrontal | 0.199000575 | 0.115152195 | 1.728152682 | 0.089783707 | - |
| GFAP | Left middle temporal | 0.00842032 | 0.121123824 | 0.069518283 | 0.944838714 | - |
| GFAP | Left parahippocampal | -0.14515517 | 0.133217057 | -1.089613997 | 0.280812747 | - |
| GFAP | Left paracentral | -0.120426138 | 0.120138935 | -1.002390586 | 0.320709685 | - |
| GFAP | Left pars opercularis | -0.256830453 | 0.122988724 | -2.088243903 | 0.041596456 | 0.298448996 |
| GFAP | Left pars orbitalis | -0.033933046 | 0.114460051 | -0.296461913 | 0.768036002 | - |
| GFAP | Left pars triangularis | -0.298896123 | 0.1234505 | -2.421181955 | 0.018926793 | 0.257404385 |
| GFAP | Left pericalcarine | 0.044647033 | 0.132833871 | 0.336111815 | 0.73811363 | - |
| GFAP | Left postcentral | 0.033859893 | 0.109729104 | 0.308577134 | 0.758852885 | - |
| GFAP | Left posterior cingulate | 0.084312054 | 0.134750133 | 0.625691801 | 0.534202327 | - |
| GFAP | Left precentral | -0.085021999 | 0.097263589 | -0.874140057 | 0.385985839 | - |
| GFAP | Left precuneus | -0.129531972 | 0.08862813 | -1.46152211 | 0.149776225 | - |
| GFAP | Left rostral anterior cingulate | -0.054980737 | 0.11089229 | -0.495803065 | 0.622083134 | - |
| GFAP | Left rostral middle frontal | -0.04918512 | 0.124445567 | -0.395234008 | 0.694256508 | - |
| GFAP | Left superior frontal | -0.146587571 | 0.109599664 | -1.337481934 | 0.186776964 | - |
| GFAP | Left superior parietal | -0.073921882 | 0.121207675 | -0.6098779 | 0.544548763 | - |
| GFAP | Left superior temporal | -0.080336913 | 0.107379626 | -0.748157883 | 0.457672072 | - |
| GFAP | Left supramarginal | -0.171373493 | 0.10044472 | -1.706147352 | 0.093834822 | - |
| GFAP | Left frontal pole | 0.256768825 | 0.126994421 | 2.02189059 | 0.048248722 | 0.298448996 |
| GFAP | Left temporal pole | -0.08909555 | 0.13063121 | -0.68203877 | 0.498184389 | - |
| GFAP | Left transverse temporal | -0.057365198 | 0.124036162 | -0.462487687 | 0.645624804 | - |
| GFAP | Left insula | -0.09332361 | 0.108183015 | -0.862645675 | 0.392219035 | - |
| GFAP | Right bankssts | -0.093741696 | 0.118741087 | -0.789463016 | 0.433359171 | - |
| GFAP | Right caudal anterior cingulate | -0.224245719 | 0.13424987 | -1.670360792 | 0.100744113 | - |
| GFAP | Right caudal middle frontal | -0.120848968 | 0.128678588 | -0.939153666 | 0.351912402 | - |
| GFAP | Right cuneus | 0.013005885 | 0.118653748 | 0.109612089 | 0.913131006 | - |
| GFAP | Right entorhinal | -0.088688714 | 0.130422712 | -0.680009733 | 0.499457939 | - |
| GFAP | Right fusiform | -0.194458947 | 0.114695175 | -1.695441384 | 0.095859622 | - |
| GFAP | Right inferior parietal | -0.174216831 | 0.108314718 | -1.608431748 | 0.113682057 | - |
| GFAP | Right inferior temporal | -0.086816389 | 0.102047205 | -0.850747344 | 0.398737021 | - |
| GFAP | Right isthmus cingulate | -0.118864057 | 0.125400481 | -0.947875604 | 0.347494824 | - |
| GFAP | Right lateral occipital | 0.033102167 | 0.118887608 | 0.278432446 | 0.78176386 | - |
| GFAP | Right lateral orbitofrontal | -0.157007819 | 0.09530197 | -1.647477163 | 0.105376944 | - |
| GFAP | Right lingual | -0.084203139 | 0.12389827 | -0.67961513 | 0.499705823 | - |
| GFAP | Right medial orbitofrontal | -0.024160422 | 0.115134774 | -0.209844699 | 0.834593472 | - |
| GFAP | Right middle temporal | 0.016054615 | 0.101802601 | 0.15770339 | 0.875289832 | - |
| GFAP | Right parahippocampal | -0.288282161 | 0.130551389 | -2.20818915 | 0.031579261 | 0.298448996 |
| GFAP | Right paracentral | -0.062696857 | 0.126626695 | -0.495131437 | 0.62255393 | - |
| GFAP | Right pars opercularis | -0.261117993 | 0.129163275 | -2.021611745 | 0.048278514 | 0.298448996 |
| GFAP | Right pars orbitalis | -0.280891196 | 0.120693076 | -2.32731824 | 0.023800184 | 0.269735419 |
| GFAP | Right pars triangularis | -0.207855719 | 0.126361533 | -1.644928751 | 0.105903468 | - |
| GFAP | Right pericalcarine | -0.021482427 | 0.12567175 | -0.170940782 | 0.864921272 | - |
| GFAP | Right postcentral | -0.07211648 | 0.11463636 | -0.62908906 | 0.53199302 | - |
| GFAP | Right posterior cingulate | 0.189351459 | 0.134540785 | 1.407390769 | 0.165149463 | - |
| GFAP | Right precentral | -0.033847719 | 0.101498227 | -0.333480883 | 0.74008709 | - |
| GFAP | Right precuneus | -0.198775762 | 0.094024046 | -2.114094971 | 0.039229507 | 0.298448996 |
| GFAP | Right rostral anterior cingulate | -0.156559875 | 0.134490714 | -1.164094312 | 0.249599385 | - |
| GFAP | Right rostral middle frontal | 0.017798145 | 0.119853436 | 0.148499245 | 0.882512351 | - |
| GFAP | Right superior frontal | 0.096403408 | 0.101779659 | 0.947177552 | 0.347847039 | - |
| GFAP | Right superior parietal | -0.023307585 | 0.10530832 | -0.221327099 | 0.825688273 | - |
| GFAP | Right superior temporal | 0.004191348 | 0.117748913 | 0.035595637 | 0.971738516 | - |
| GFAP | Right supramarginal | -0.242118193 | 0.091900672 | -2.634563891 | 0.011020719 | 0.249802964 |
| GFAP | Right frontal pole | 0.162252617 | 0.134644701 | 1.205042724 | 0.233539961 | - |
| GFAP | Right temporal pole | -0.123514696 | 0.128732082 | -0.959470973 | 0.341678308 | - |
| GFAP | Right transverse temporal | -0.071307983 | 0.117377954 | -0.607507461 | 0.546108434 | - |
| GFAP | Right insula | -0.24073401 | 0.099394034 | -2.4220167 | 0.018887806 | 0.257404385 |

Abbreviations: FDR, false discovery rate; GFAP, glial fibrillary acidic protein; MRI, magnetic resonance imaging; MSA, multiple system atrophy; NfL, neurofilament light.

Note: Associations of plasma measures with MRI indices were analyzed using multiple linear regressions after adjusting for intracranial volumes.

**Supplementary Table 11: Associations of plasma measures with PET imaging indices in MSA patients after expanding the sample size**

| Plasma measure | Brain regions (glucose metabolism) | β | Standard error | t | P | P value (FDR corrected) |
| --- | --- | --- | --- | --- | --- | --- |
| NfL | Left cerebral white matter | -0.277362793 | 0.211292354 | -1.312696782 | 0.201700075 | - |
| NfL | Left cerebellum white matter | -0.222218185 | 0.209097491 | -1.06274917 | 0.298472833 | - |
| NfL | Left cerebellum cortex | -0.021576102 | 0.208594312 | -0.103435714 | 0.918476652 | - |
| NfL | Left thalamus | -0.31480713 | 0.202723828 | -1.552886673 | 0.133538806 | - |
| NfL | Left caudate | -0.362078831 | 0.19096768 | -1.896021516 | 0.070060035 | - |
| NfL | Left putamen | -0.393843656 | 0.194229427 | -2.027723923 | 0.053830308 | - |
| NfL | Left pallidum | -0.189094799 | 0.214214503 | -0.882735745 | 0.386135646 | - |
| NfL | Brain stem | -0.364416095 | 0.205016128 | -1.777499652 | 0.088162162 | - |
| NfL | Left hippocampus | -0.357694841 | 0.206298358 | -1.73387149 | 0.095770592 | - |
| NfL | Left amygdala | -0.337361338 | 0.203458453 | -1.6581338 | 0.11030538 | - |
| NfL | Left accumbens | -0.368911193 | 0.196157797 | -1.880685844 | 0.072203529 | - |
| NfL | Right cerebral white matter | -0.252529302 | 0.212727566 | -1.187101921 | 0.246804893 | - |
| NfL | Right cerebellum white matter | -0.213185275 | 0.210687519 | -1.011855262 | 0.321700406 | - |
| NfL | Right cerebellum cortex | 0.021576102 | 0.208594312 | 0.103435714 | 0.918476652 | - |
| NfL | Right thalamus | -0.294884267 | 0.202978014 | -1.452789199 | 0.159232748 | - |
| NfL | Right caudate | -0.302328771 | 0.196812483 | -1.53612599 | 0.137587268 | - |
| NfL | Right putamen | -0.340380443 | 0.19654278 | -1.731838959 | 0.096138288 | - |
| NfL | Right pallidum | -0.249136229 | 0.212518671 | -1.172302778 | 0.252581192 | - |
| NfL | Right hippocampus | -0.331147025 | 0.208520147 | -1.588081678 | 0.125356401 | - |
| NfL | Right amygdala | -0.319986315 | 0.206453828 | -1.549917087 | 0.13424887 | - |
| NfL | Right accumbens | -0.433667707 | 0.198147844 | -2.188606744 | 0.038591954 | 0.289867746 |
| NfL | Pons | -0.347756639 | 0.183739138 | -1.892665022 | 0.070524375 | - |
| NfL | Left bankssts | -0.29624091 | 0.207306987 | -1.428996262 | 0.165892674 | - |
| NfL | Left caudal anterior cingulate | -0.303238485 | 0.200548799 | -1.512043385 | 0.143579894 | - |
| NfL | Left caudal middle frontal | -0.256108296 | 0.202410537 | -1.265291326 | 0.217914042 | - |
| NfL | Left cuneus | -0.20080575 | 0.206258931 | -0.973561481 | 0.339985362 | - |
| NfL | Left entorhinal | -0.258352437 | 0.21097008 | -1.224592778 | 0.232613274 | - |
| NfL | Left fusiform | -0.292259054 | 0.206939401 | -1.41229294 | 0.170699895 | - |
| NfL | Left inferior parietal | -0.271438184 | 0.20563936 | -1.319971936 | 0.199296674 | - |
| NfL | Left inferior temporal | -0.290812244 | 0.205157295 | -1.417508669 | 0.169187028 | - |
| NfL | Left isthmus cingulate | -0.347630009 | 0.196949861 | -1.765068573 | 0.090275456 | - |
| NfL | Left lateral occipital | -0.307508806 | 0.206459188 | -1.489441131 | 0.149396161 | - |
| NfL | Left lateral orbitofrontal | -0.654997956 | 0.16005423 | -4.092350172 | 0.000417133 | 0.024372675 |
| NfL | Left lingual | -0.255756562 | 0.211753922 | -1.207800824 | 0.238891741 | - |
| NfL | Left medial orbitofrontal | -0.636415696 | 0.159538534 | -3.989103329 | 0.000541615 | 0.024372675 |
| NfL | Left middle temporal | -0.294623097 | 0.205395773 | -1.434416556 | 0.164356183 | - |
| NfL | Left parahippocampal | -0.265512553 | 0.203664693 | -1.303674922 | 0.204711654 | - |
| NfL | Left paracentral | -0.259142895 | 0.210817861 | -1.229226468 | 0.230902852 | - |
| NfL | Left pars opercularis | -0.33698592 | 0.1984267 | -1.698289193 | 0.102383156 | - |
| NfL | Left pars orbitalis | -0.542752999 | 0.182258525 | -2.977929283 | 0.006539452 | 0.19618356 |
| NfL | Left pars triangularis | -0.357754988 | 0.192795467 | -1.855619295 | 0.075830086 | - |
| NfL | Left pericalcarine | -0.157959333 | 0.204115972 | -0.773870521 | 0.446563882 | - |
| NfL | Left postcentral | -0.205246018 | 0.209053919 | -0.981785079 | 0.336000013 | - |
| NfL | Left posterior cingulate | -0.266185208 | 0.205550909 | -1.294984338 | 0.207645416 | - |
| NfL | Left precentral | -0.288586784 | 0.208354328 | -1.385076981 | 0.178769571 | - |
| NfL | Left precuneus | -0.28619703 | 0.205092833 | -1.395451153 | 0.175658653 | - |
| NfL | Left rostral anterior cingulate | -0.354050973 | 0.195269884 | -1.813136598 | 0.082337487 | - |
| NfL | Left rostral middle frontal | -0.35733406 | 0.194571088 | -1.836521875 | 0.078698123 | - |
| NfL | Left superior frontal | -0.357616662 | 0.198126808 | -1.804988757 | 0.083639154 | - |
| NfL | Left superior parietal | -0.225575215 | 0.207612979 | -1.086517886 | 0.288042522 | - |
| NfL | Left superior temporal | -0.317857294 | 0.20341904 | -1.562573952 | 0.131243917 | - |
| NfL | Left supramarginal | -0.225208282 | 0.206483833 | -1.090682397 | 0.286242256 | - |
| NfL | Left frontal pole | -0.488296374 | 0.176883954 | -2.760546461 | 0.01087958 | 0.24479055 |
| NfL | Left temporal pole | -0.298647226 | 0.209902502 | -1.422790215 | 0.167665987 | - |
| NfL | Left transverse temporal | -0.317983036 | 0.189230692 | -1.680398842 | 0.105851309 | - |
| NfL | Left insula | -0.353810339 | 0.197967054 | -1.787218286 | 0.086539654 | - |
| NfL | Right bankssts | -0.311712422 | 0.205430675 | -1.517360648 | 0.14223876 | - |
| NfL | Right caudal anterior cingulate | -0.334676637 | 0.203534782 | -1.644321593 | 0.113146829 | - |
| NfL | Right caudal middle frontal | -0.226799324 | 0.203117728 | -1.116590493 | 0.275224041 | - |
| NfL | Right cuneus | -0.186838624 | 0.209937622 | -0.889972092 | 0.382315507 | - |
| NfL | Right entorhinal | -0.312961853 | 0.207913099 | -1.505253178 | 0.14530751 | - |
| NfL | Right fusiform | -0.277068357 | 0.207786604 | -1.333427428 | 0.194910264 | - |
| NfL | Right inferior parietal | -0.263800902 | 0.209906442 | -1.256754675 | 0.220936984 | - |
| NfL | Right inferior temporal | -0.268844536 | 0.208813176 | -1.287488368 | 0.210201859 | - |
| NfL | Right isthmus cingulate | -0.265674434 | 0.205233998 | -1.294495237 | 0.207811485 | - |
| NfL | Right lateral occipital | -0.228523193 | 0.214031759 | -1.067706934 | 0.296275377 | - |
| NfL | Right lateral orbitofrontal | -0.390370964 | 0.196825219 | -1.983338143 | 0.05888418 | - |
| NfL | Right lingual | -0.221114264 | 0.206697023 | -1.069750599 | 0.295372911 | - |
| NfL | Right medial orbitofrontal | -0.372222405 | 0.195068129 | -1.908166172 | 0.068402151 | - |
| NfL | Right middle temporal | -0.26370025 | 0.207025197 | -1.273759201 | 0.21494679 | - |
| NfL | Right parahippocampal | -0.289234222 | 0.204110676 | -1.417046027 | 0.169320788 | - |
| NfL | Right paracentral | -0.250379792 | 0.212345952 | -1.179112623 | 0.249910887 | - |
| NfL | Right pars opercularis | -0.368168122 | 0.194657273 | -1.891365864 | 0.070704821 | - |
| NfL | Right pars orbitalis | -0.38097532 | 0.201036171 | -1.895058582 | 0.070192975 | - |
| NfL | Right pars triangularis | -0.365631185 | 0.197250874 | -1.853635306 | 0.076123761 | - |
| NfL | Right pericalcarine | -0.208980941 | 0.204931765 | -1.019758654 | 0.31801312 | - |
| NfL | Right postcentral | -0.232403917 | 0.206705591 | -1.12432332 | 0.271995818 | - |
| NfL | Right posterior cingulate | -0.271177515 | 0.202841304 | -1.336894952 | 0.19379216 | - |
| NfL | Right precentral | -0.314605621 | 0.206324175 | -1.524812211 | 0.140376537 | - |
| NfL | Right precuneus | -0.299968848 | 0.20600402 | -1.45613104 | 0.158314787 | - |
| NfL | Right rostral anterior cingulate | -0.326031931 | 0.191879061 | -1.699153251 | 0.102218114 | - |
| NfL | Right rostral middle frontal | -0.346414666 | 0.199763695 | -1.734122239 | 0.095725314 | - |
| NfL | Right superior frontal | -0.360701386 | 0.199143992 | -1.811259191 | 0.082635856 | - |
| NfL | Right superior parietal | -0.273533221 | 0.208657843 | -1.310917513 | 0.202291277 | - |
| NfL | Right superior temporal | -0.295825849 | 0.202798185 | -1.458720397 | 0.157606458 | - |
| NfL | Right supramarginal | -0.238620148 | 0.209021557 | -1.141605446 | 0.264880916 | - |
| NfL | Right frontal pole | -0.384658884 | 0.192165786 | -2.001703279 | 0.056744322 | - |
| NfL | Right temporal pole | -0.304336601 | 0.209201414 | -1.454754033 | 0.158692516 | - |
| NfL | Right transverse temporal | -0.327644506 | 0.192315168 | -1.703685201 | 0.10135616 | - |
| NfL | Right insula | -0.340518484 | 0.199845048 | -1.703912548 | 0.101313082 | - |
|  |  |  |  |  |  |  |
|  |  |  |  |  |  |  |
| Plasma measure | Brain regions (glucose metabolism) | β | Standard error | t | P | P value (FDR corrected) |
| GFAP | Left cerebral white matter | 0.360733758 | 0.197652247 | 1.825093129 | 0.08045899 | - |
| GFAP | Left cerebellum white matter | 0.479600773 | 0.181601995 | 2.640944413 | 0.014312172 | 0.073802461 |
| GFAP | Left cerebellum cortex | -0.03463421 | 0.201054644 | -0.172262673 | 0.864675424 | - |
| GFAP | Left thalamus | 0.374738625 | 0.19025753 | 1.969638864 | 0.060526454 | - |
| GFAP | Left caudate | 0.440394256 | 0.175796533 | 2.505136182 | 0.019430649 | 0.073802461 |
| GFAP | Left putamen | 0.375110428 | 0.187672806 | 1.998746836 | 0.057084087 | - |
| GFAP | Left pallidum | 0.318510825 | 0.199555527 | 1.596101244 | 0.123551214 | - |
| GFAP | Brain stem | 0.287519137 | 0.201938168 | 1.423797886 | 0.16737703 | - |
| GFAP | Left hippocampus | 0.411735683 | 0.19355161 | 2.127265602 | 0.043872587 | 0.076401388 |
| GFAP | Left amygdala | 0.436443772 | 0.186974852 | 2.334237831 | 0.028281814 | 0.073802461 |
| GFAP | Left accumbens | 0.314651662 | 0.192150654 | 1.637525844 | 0.114567208 | - |
| GFAP | Right cerebral white matter | 0.380847528 | 0.196216567 | 1.940955004 | 0.064096272 | - |
| GFAP | Right cerebellum white matter | 0.419897194 | 0.188905106 | 2.222794309 | 0.035904599 | 0.07443306 |
| GFAP | Right cerebellum cortex | 0.03463421 | 0.201054644 | 0.172262673 | 0.864675424 | - |
| GFAP | Right thalamus | 0.374194648 | 0.189315135 | 1.97657017 | 0.059690548 | - |
| GFAP | Right caudate | 0.456440457 | 0.175710509 | 2.597684451 | 0.015786826 | 0.073802461 |
| GFAP | Right putamen | 0.398622609 | 0.1838012 | 2.168770439 | 0.040233274 | 0.07443306 |
| GFAP | Right pallidum | 0.310214893 | 0.200963238 | 1.543640002 | 0.135760012 | - |
| GFAP | Right hippocampus | 0.418603249 | 0.193321182 | 2.165325315 | 0.040524666 | 0.07443306 |
| GFAP | Right amygdala | 0.42749846 | 0.189685869 | 2.253718019 | 0.033621121 | 0.073802461 |
| GFAP | Right accumbens | 0.366779425 | 0.195409206 | 1.876981303 | 0.072729803 | - |
| GFAP | Pons | 0.386882828 | 0.172733332 | 2.239769375 | 0.034634274 | 0.074216301 |
| GFAP | Left bankssts | 0.362892553 | 0.194601486 | 1.86479847 | 0.074484121 | - |
| GFAP | Left caudal anterior cingulate | 0.352921178 | 0.189121433 | 1.866108837 | 0.074293681 | - |
| GFAP | Left caudal middle frontal | 0.41547703 | 0.182866991 | 2.272017645 | 0.032332809 | 0.073802461 |
| GFAP | Left cuneus | 0.378839817 | 0.187446652 | 2.021054052 | 0.054564325 | - |
| GFAP | Left entorhinal | 0.387882618 | 0.194161069 | 1.997736315 | 0.057200631 | - |
| GFAP | Left fusiform | 0.465156672 | 0.18468639 | 2.518629942 | 0.018854601 | 0.073802461 |
| GFAP | Left inferior parietal | 0.473345322 | 0.181205652 | 2.61219955 | 0.015276877 | 0.073802461 |
| GFAP | Left inferior temporal | 0.431658361 | 0.186132519 | 2.319091592 | 0.029222766 | 0.073802461 |
| GFAP | Left isthmus cingulate | 0.271112822 | 0.194121814 | 1.39661183 | 0.175313285 | - |
| GFAP | Left lateral occipital | 0.35287694 | 0.195207168 | 1.807704826 | 0.08320329 | - |
| GFAP | Left lateral orbitofrontal | 0.237711813 | 0.195149806 | 1.218099153 | 0.235026309 | - |
| GFAP | Left lingual | 0.309921471 | 0.200553298 | 1.545332207 | 0.135351263 | - |
| GFAP | Left medial orbitofrontal | 0.258701504 | 0.191223467 | 1.352875295 | 0.188703914 | - |
| GFAP | Left middle temporal | 0.444821963 | 0.185316049 | 2.400342359 | 0.024491526 | 0.073802461 |
| GFAP | Left parahippocampal | 0.261748191 | 0.196065687 | 1.335002545 | 0.194401745 | - |
| GFAP | Left paracentral | 0.455922258 | 0.187783117 | 2.427919318 | 0.023053234 | 0.073802461 |
| GFAP | Left pars opercularis | 0.349408822 | 0.189524454 | 1.843608116 | 0.077623144 | - |
| GFAP | Left pars orbitalis | 0.289933226 | 0.196961918 | 1.47202682 | 0.154006617 | - |
| GFAP | Left pars triangularis | 0.400074436 | 0.181240141 | 2.207427304 | 0.037090848 | 0.07443306 |
| GFAP | Left pericalcarine | 0.419657793 | 0.179902941 | 2.332690012 | 0.028376683 | 0.073802461 |
| GFAP | Left postcentral | 0.438688265 | 0.1850586 | 2.370537032 | 0.026138633 | 0.073802461 |
| GFAP | Left posterior cingulate | 0.410356546 | 0.187113794 | 2.193085487 | 0.03822984 | 0.07443306 |
| GFAP | Left precentral | 0.451601644 | 0.187324691 | 2.410796154 | 0.02393684 | 0.073802461 |
| GFAP | Left precuneus | 0.428006972 | 0.186140681 | 2.299373619 | 0.030490622 | 0.073802461 |
| GFAP | Left rostral anterior cingulate | 0.413684908 | 0.182145942 | 2.271172796 | 0.032391283 | 0.073802461 |
| GFAP | Left rostral middle frontal | 0.420963905 | 0.181000548 | 2.325760398 | 0.028804996 | 0.073802461 |
| GFAP | Left superior frontal | 0.381084389 | 0.188148821 | 2.025441281 | 0.05408052 | - |
| GFAP | Left superior parietal | 0.442092035 | 0.184127256 | 2.40101354 | 0.02445556 | 0.073802461 |
| GFAP | Left superior temporal | 0.388788924 | 0.189968981 | 2.046591623 | 0.051801169 | - |
| GFAP | Left supramarginal | 0.49956296 | 0.176654471 | 2.827910085 | 0.009304904 | 0.073802461 |
| GFAP | Left frontal pole | 0.30910258 | 0.185326103 | 1.66788474 | 0.108335702 | - |
| GFAP | Left temporal pole | 0.378150465 | 0.196114457 | 1.928213104 | 0.06574048 | - |
| GFAP | Left transverse temporal | 0.327104017 | 0.180974073 | 1.807463422 | 0.08324195 | - |
| GFAP | Left insula | 0.437032264 | 0.182562539 | 2.393877008 | 0.024840482 | 0.073802461 |
| GFAP | Right bankssts | 0.328913203 | 0.196197764 | 1.676437063 | 0.106632583 | - |
| GFAP | Right caudal anterior cingulate | 0.36066666 | 0.193482205 | 1.864081817 | 0.074588454 | - |
| GFAP | Right caudal middle frontal | 0.481689405 | 0.175167141 | 2.749884504 | 0.011150802 | 0.073802461 |
| GFAP | Right cuneus | 0.49490052 | 0.179233687 | 2.761202592 | 0.010863094 | 0.073802461 |
| GFAP | Right entorhinal | 0.436175348 | 0.189890583 | 2.296982512 | 0.030647737 | 0.073802461 |
| GFAP | Right fusiform | 0.462365207 | 0.184955615 | 2.499871149 | 0.019659813 | 0.073802461 |
| GFAP | Right inferior parietal | 0.500793997 | 0.182240543 | 2.747983458 | 0.011199827 | 0.073802461 |
| GFAP | Right inferior temporal | 0.483401007 | 0.183312107 | 2.637038081 | 0.014439835 | 0.073802461 |
| GFAP | Right isthmus cingulate | 0.452772512 | 0.182632351 | 2.479147365 | 0.02058641 | 0.073802461 |
| GFAP | Right lateral occipital | 0.40178998 | 0.194648696 | 2.064180178 | 0.049971159 | 0.080310791 |
| GFAP | Right lateral orbitofrontal | 0.39132385 | 0.188524788 | 2.075715631 | 0.048802447 | 0.07985855 |
| GFAP | Right lingual | 0.391648575 | 0.187683739 | 2.086747514 | 0.047707635 | 0.079512725 |
| GFAP | Right medial orbitofrontal | 0.415910048 | 0.183134573 | 2.271062425 | 0.032398929 | 0.073802461 |
| GFAP | Right middle temporal | 0.513863992 | 0.177595328 | 2.893454455 | 0.007981991 | 0.073802461 |
| GFAP | Right parahippocampal | 0.345269087 | 0.19237513 | 1.794769873 | 0.08529669 | - |
| GFAP | Right paracentral | 0.495084641 | 0.184767186 | 2.679505224 | 0.013107517 | 0.073802461 |
| GFAP | Right pars opercularis | 0.396979669 | 0.184158334 | 2.155643245 | 0.041353795 | 0.074436831 |
| GFAP | Right pars orbitalis | 0.432609138 | 0.188151994 | 2.299253539 | 0.030498495 | 0.073802461 |
| GFAP | Right pars triangularis | 0.404581861 | 0.185831432 | 2.177144393 | 0.039532856 | 0.07443306 |
| GFAP | Right pericalcarine | 0.422650344 | 0.182470752 | 2.316263508 | 0.0294016 | 0.073802461 |
| GFAP | Right postcentral | 0.489430399 | 0.178429534 | 2.742989841 | 0.011329575 | 0.073802461 |
| GFAP | Right posterior cingulate | 0.393479639 | 0.186150247 | 2.113774467 | 0.04511768 | 0.076614928 |
| GFAP | Right precentral | 0.451031493 | 0.186915103 | 2.413028616 | 0.023819894 | 0.073802461 |
| GFAP | Right precuneus | 0.44785674 | 0.185970291 | 2.408216585 | 0.024072627 | 0.073802461 |
| GFAP | Right rostral anterior cingulate | 0.307025175 | 0.185529958 | 1.654854983 | 0.110974418 | - |
| GFAP | Right rostral middle frontal | 0.447081484 | 0.182818622 | 2.445492027 | 0.022177814 | 0.073802461 |
| GFAP | Right superior frontal | 0.411664855 | 0.186685345 | 2.205126782 | 0.037271457 | 0.07443306 |
| GFAP | Right superior parietal | 0.469093897 | 0.184957735 | 2.536222113 | 0.018127418 | 0.073802461 |
| GFAP | Right superior temporal | 0.440381813 | 0.183159726 | 2.404359422 | 0.024276992 | 0.073802461 |
| GFAP | Right supramarginal | 0.510666697 | 0.178775751 | 2.85646512 | 0.008704821 | 0.073802461 |
| GFAP | Right frontal pole | 0.410125646 | 0.181816062 | 2.255717347 | 0.033478129 | 0.073802461 |
| GFAP | Right temporal pole | 0.410111413 | 0.193056623 | 2.124306361 | 0.044143024 | 0.076401388 |
| GFAP | Right transverse temporal | 0.155857877 | 0.193735678 | 0.804487222 | 0.429013272 | - |
| GFAP | Right insula | 0.433924922 | 0.183790443 | 2.360976527 | 0.026688154 | 0.073802461 |
|  |  |  |  |  |  |  |
|  |  |  |  |  |  |  |
| Plasma measure | Brain regions (dopamine uptake) | β | Standard error | t | P | P value (FDR corrected) |
| NfL | Left cerebral white matter | 0.039274858 | 0.217404706 | 0.180653209 | 0.858156628 | - |
| NfL | Left cerebellum white matter | 0.09348075 | 0.215443141 | 0.433899867 | 0.668236741 | - |
| NfL | Left cerebellum cortex | -0.007269201 | 0.197845741 | -0.036741763 | 0.970994812 | - |
| NfL | Left thalamus | 0.022436321 | 0.216444051 | 0.103658758 | 0.918301513 | - |
| NfL | Left caudate | -0.053444326 | 0.216829461 | -0.246480925 | 0.807405963 | - |
| NfL | Left putamen | -0.122853113 | 0.216358711 | -0.567821434 | 0.575430366 | - |
| NfL | Left pallidum | 0.047164386 | 0.217196187 | 0.217151079 | 0.829926058 | - |
| NfL | Brain stem | -0.086409842 | 0.216329741 | -0.399435795 | 0.693104538 | - |
| NfL | Left hippocampus | -0.141833069 | 0.208285685 | -0.680954474 | 0.502418057 | - |
| NfL | Left amygdala | 0.037439931 | 0.214041198 | 0.174919276 | 0.862610366 | - |
| NfL | Left accumbens | -0.014317104 | 0.215027051 | -0.066582804 | 0.947465403 | - |
| NfL | Right cerebral white matter | 0.054286237 | 0.217628306 | 0.249444742 | 0.805139342 | - |
| NfL | Right cerebellum white matter | 0.082629656 | 0.216167877 | 0.38224762 | 0.705641699 | - |
| NfL | Right cerebellum cortex | 0.007269201 | 0.197845741 | 0.036741763 | 0.970994812 | - |
| NfL | Right thalamus | -0.007352299 | 0.216308997 | -0.033989799 | 0.973166399 | - |
| NfL | Right caudate | -0.05568302 | 0.216534964 | -0.257154868 | 0.799251117 | - |
| NfL | Right putamen | -0.094776532 | 0.21702435 | -0.436709207 | 0.666226129 | - |
| NfL | Right pallidum | 0.036511125 | 0.216824481 | 0.168390236 | 0.867687343 | - |
| NfL | Right hippocampus | -0.060355277 | 0.209912986 | -0.287525217 | 0.776178304 | - |
| NfL | Right amygdala | -0.080305551 | 0.212356152 | -0.378164464 | 0.708632677 | - |
| NfL | Right accumbens | -0.053191578 | 0.2161027 | -0.246140276 | 0.807666589 | - |
| NfL | Pons | 0.041281645 | 0.216796448 | 0.19041661 | 0.850584184 | - |
| NfL | Left bankssts | 0.012777107 | 0.212074635 | 0.060248163 | 0.95245688 | - |
| NfL | Left caudal anterior cingulate | 0.009724443 | 0.204699737 | 0.047505888 | 0.96250315 | - |
| NfL | Left caudal middle frontal | 0.082245753 | 0.213423849 | 0.385363462 | 0.703362541 | - |
| NfL | Left cuneus | -0.031452351 | 0.203858325 | -0.154285342 | 0.878674846 | - |
| NfL | Left entorhinal | 0.057348997 | 0.216844667 | 0.264470407 | 0.793675434 | - |
| NfL | Left fusiform | 0.063140351 | 0.214839119 | 0.293895969 | 0.771364098 | - |
| NfL | Left inferior parietal | 0.099241726 | 0.207899966 | 0.477353259 | 0.63742907 | - |
| NfL | Left inferior temporal | 0.056014296 | 0.212549026 | 0.263535884 | 0.794387082 | - |
| NfL | Left isthmus cingulate | 0.022433988 | 0.21185073 | 0.105895258 | 0.916545597 | - |
| NfL | Left lateral occipital | 0.017807541 | 0.214322114 | 0.083087746 | 0.934470898 | - |
| NfL | Left lateral orbitofrontal | -0.233726614 | 0.208858038 | -1.119069277 | 0.274186207 | - |
| NfL | Left lingual | -0.121033288 | 0.203901253 | -0.593587763 | 0.558341384 | - |
| NfL | Left medial orbitofrontal | -0.357536779 | 0.202502352 | -1.765593216 | 0.090185398 | - |
| NfL | Left middle temporal | 0.083483716 | 0.208145411 | 0.401083626 | 0.691907212 | - |
| NfL | Left parahippocampal | 0.028890592 | 0.213764261 | 0.135151648 | 0.893619103 | - |
| NfL | Left paracentral | 0.087805565 | 0.209444649 | 0.419230405 | 0.67877638 | - |
| NfL | Left pars opercularis | 0.054575689 | 0.207870513 | 0.262546566 | 0.795140654 | - |
| NfL | Left pars orbitalis | -0.064918626 | 0.205662505 | -0.315656109 | 0.754992132 | - |
| NfL | Left pars triangularis | 0.0686181 | 0.204421497 | 0.335669688 | 0.740036697 | - |
| NfL | Left pericalcarine | -0.042952912 | 0.206511245 | -0.207993092 | 0.836989307 | - |
| NfL | Left postcentral | 0.058724145 | 0.206788634 | 0.283981494 | 0.778860179 | - |
| NfL | Left posterior cingulate | -0.007062663 | 0.209310931 | -0.033742448 | 0.973361595 | - |
| NfL | Left precentral | 0.029418729 | 0.21066839 | 0.139644723 | 0.890105989 | - |
| NfL | Left precuneus | 0.02349216 | 0.206759665 | 0.113620612 | 0.910483673 | - |
| NfL | Left rostral anterior cingulate | 0.100256217 | 0.204873464 | 0.48935677 | 0.629031901 | - |
| NfL | Left rostral middle frontal | 0.073971313 | 0.209935216 | 0.352353046 | 0.727648919 | - |
| NfL | Left superior frontal | 0.027051747 | 0.211055885 | 0.128173386 | 0.899079751 | - |
| NfL | Left superior parietal | 0.102119447 | 0.210109709 | 0.486029169 | 0.631354713 | - |
| NfL | Left superior temporal | 0.01618998 | 0.210684945 | 0.076844503 | 0.939384282 | - |
| NfL | Left supramarginal | 0.072081021 | 0.210278209 | 0.342788829 | 0.734741566 | - |
| NfL | Left frontal pole | 0.044409736 | 0.205661021 | 0.215936573 | 0.830861939 | - |
| NfL | Left temporal pole | 0.090474703 | 0.212932923 | 0.424897671 | 0.674696518 | - |
| NfL | Left transverse temporal | -0.101460196 | 0.211154338 | -0.480502539 | 0.635221105 | - |
| NfL | Left insula | -0.025243529 | 0.209050173 | -0.120753445 | 0.904891595 | - |
| NfL | Right bankssts | 0.043286613 | 0.212724572 | 0.203486661 | 0.840470144 | - |
| NfL | Right caudal anterior cingulate | -0.039361704 | 0.211044281 | -0.186509221 | 0.85361302 | - |
| NfL | Right caudal middle frontal | 0.109200973 | 0.213387198 | 0.511750349 | 0.613502526 | - |
| NfL | Right cuneus | -0.004948454 | 0.20187417 | -0.024512566 | 0.980646437 | - |
| NfL | Right entorhinal | -0.051693107 | 0.215980957 | -0.23934104 | 0.812873319 | - |
| NfL | Right fusiform | 0.030543209 | 0.214574995 | 0.142342816 | 0.88799746 | - |
| NfL | Right inferior parietal | 0.084776357 | 0.204500008 | 0.414554297 | 0.682150269 | - |
| NfL | Right inferior temporal | 0.062128191 | 0.214538106 | 0.289590471 | 0.774616635 | - |
| NfL | Right isthmus cingulate | 0.0099409 | 0.207802564 | 0.047838199 | 0.962241062 | - |
| NfL | Right lateral occipital | 0.064265699 | 0.209462581 | 0.306812311 | 0.761632441 | - |
| NfL | Right lateral orbitofrontal | 0.057398705 | 0.211044082 | 0.271974956 | 0.787967326 | - |
| NfL | Right lingual | -0.052131141 | 0.214473695 | -0.24306543 | 0.810020143 | - |
| NfL | Right medial orbitofrontal | 0.068406475 | 0.204863614 | 0.333912274 | 0.741345871 | - |
| NfL | Right middle temporal | 0.072383646 | 0.208477866 | 0.347200629 | 0.731466821 | - |
| NfL | Right parahippocampal | 0.024876185 | 0.217512511 | 0.114366685 | 0.909898532 | - |
| NfL | Right paracentral | 0.028927748 | 0.207844515 | 0.139179753 | 0.890469442 | - |
| NfL | Right pars opercularis | -0.008425248 | 0.209832546 | -0.040152244 | 0.968303906 | - |
| NfL | Right pars orbitalis | -0.091370523 | 0.208873744 | -0.437443792 | 0.665700815 | - |
| NfL | Right pars triangularis | -0.002135332 | 0.202182799 | -0.010561394 | 0.991660687 | - |
| NfL | Right pericalcarine | -0.10647832 | 0.194952509 | -0.546175681 | 0.589986892 | - |
| NfL | Right postcentral | 0.022566508 | 0.211549982 | 0.106672228 | 0.915935686 | - |
| NfL | Right posterior cingulate | -0.030688934 | 0.209012664 | -0.146828106 | 0.88449413 | - |
| NfL | Right precentral | -0.021053929 | 0.21252154 | -0.09906727 | 0.921907689 | - |
| NfL | Right precuneus | 0.004011233 | 0.209535039 | 0.019143496 | 0.984884898 | - |
| NfL | Right rostral anterior cingulate | -0.097510475 | 0.204716123 | -0.476320445 | 0.638153925 | - |
| NfL | Right rostral middle frontal | -0.00284928 | 0.212416914 | -0.013413622 | 0.989408683 | - |
| NfL | Right superior frontal | 0.018886157 | 0.211203195 | 0.089421739 | 0.929488806 | - |
| NfL | Right superior parietal | 0.090302764 | 0.209133147 | 0.431795558 | 0.669744432 | - |
| NfL | Right superior temporal | -0.005881859 | 0.212410297 | -0.027691026 | 0.978137556 | - |
| NfL | Right supramarginal | 0.065412131 | 0.211178315 | 0.309748335 | 0.759425846 | - |
| NfL | Right frontal pole | 0.091904889 | 0.209039779 | 0.439652633 | 0.664122287 | - |
| NfL | Right temporal pole | 0.093662693 | 0.216797495 | 0.432028485 | 0.669577475 | - |
| NfL | Right transverse temporal | -0.151180452 | 0.208769279 | -0.724150858 | 0.4759735 | - |
| NfL | Right insula | 0.01647891 | 0.212525633 | 0.077538458 | 0.93883802 | - |
|  |  |  |  |  |  |  |
|  |  |  |  |  |  |  |
| Plasma measure | Brain regions (dopamine uptake) | β | Standard error | t | P | P value (FDR corrected) |
| GFAP | Left cerebral white matter | 0.025965038 | 0.209704884 | 0.123817038 | 0.902491288 | - |
| GFAP | Left cerebellum white matter | 0.074427281 | 0.207997073 | 0.3578285 | 0.723599516 | - |
| GFAP | Left cerebellum cortex | -0.063710165 | 0.190331512 | -0.334732616 | 0.740734663 | - |
| GFAP | Left thalamus | 0.039918403 | 0.208590696 | 0.191371924 | 0.849844021 | - |
| GFAP | Left caudate | 0.051509916 | 0.209074935 | 0.246370594 | 0.807490373 | - |
| GFAP | Left putamen | 0.07287727 | 0.209489924 | 0.347879596 | 0.730963305 | - |
| GFAP | Left pallidum | -0.016180523 | 0.209607928 | -0.077194231 | 0.939108982 | - |
| GFAP | Brain stem | 0.021682163 | 0.209238249 | 0.103624279 | 0.918328586 | - |
| GFAP | Left hippocampus | 0.103601045 | 0.201661567 | 0.51373718 | 0.61213343 | - |
| GFAP | Left amygdala | 0.07850439 | 0.20589502 | 0.381283576 | 0.706347443 | - |
| GFAP | Left accumbens | -0.024654514 | 0.20729482 | -0.118934539 | 0.906317137 | - |
| GFAP | Right cerebral white matter | 0.037541872 | 0.209977031 | 0.178790375 | 0.859603035 | - |
| GFAP | Right cerebellum white matter | 0.012440806 | 0.209054876 | 0.059509764 | 0.953038847 | - |
| GFAP | Right cerebellum cortex | 0.063710165 | 0.190331512 | 0.334732616 | 0.740734663 | - |
| GFAP | Right thalamus | 0.04132005 | 0.208407264 | 0.198265882 | 0.844506902 | - |
| GFAP | Right caudate | 0.053812651 | 0.208789435 | 0.257736466 | 0.798807438 | - |
| GFAP | Right putamen | 0.046500518 | 0.209877891 | 0.221559866 | 0.826530873 | - |
| GFAP | Right pallidum | -0.00729713 | 0.209188062 | -0.034883108 | 0.972461461 | - |
| GFAP | Right hippocampus | 0.117627118 | 0.201327184 | 0.584258497 | 0.564498454 | - |
| GFAP | Right amygdala | -0.011143632 | 0.205357925 | -0.054264438 | 0.957173675 | - |
| GFAP | Right accumbens | 0.009002283 | 0.208628678 | 0.043149787 | 0.965939128 | - |
| GFAP | Pons | 0.006862229 | 0.209196025 | 0.032802866 | 0.974103079 | - |
| GFAP | Left bankssts | 0.088507008 | 0.203705814 | 0.434484446 | 0.667818155 | - |
| GFAP | Left caudal anterior cingulate | 0.144820503 | 0.195161918 | 0.742053084 | 0.465255906 | - |
| GFAP | Left caudal middle frontal | 0.212262 | 0.201828242 | 1.051696224 | 0.30341348 | - |
| GFAP | Left cuneus | 0.182309582 | 0.193111952 | 0.944061614 | 0.354545844 | - |
| GFAP | Left entorhinal | 0.08889504 | 0.208606107 | 0.426138244 | 0.67380478 | - |
| GFAP | Left fusiform | 0.148515625 | 0.205301787 | 0.723401521 | 0.476425228 | - |
| GFAP | Left inferior parietal | 0.189729912 | 0.197655483 | 0.95990209 | 0.346675973 | - |
| GFAP | Left inferior temporal | 0.119130283 | 0.203798017 | 0.584550745 | 0.564305051 | - |
| GFAP | Left isthmus cingulate | 0.114308321 | 0.20298508 | 0.563136565 | 0.578565547 | - |
| GFAP | Left lateral occipital | 0.09458442 | 0.205783021 | 0.459631796 | 0.649917038 | - |
| GFAP | Left lateral orbitofrontal | 0.115042442 | 0.205236689 | 0.560535463 | 0.580309921 | - |
| GFAP | Left lingual | 0.028816531 | 0.197959446 | 0.14556785 | 0.88547824 | - |
| GFAP | Left medial orbitofrontal | 0.082156997 | 0.206875337 | 0.397132872 | 0.694779224 | - |
| GFAP | Left middle temporal | 0.165129306 | 0.198531675 | 0.831752949 | 0.413747928 | - |
| GFAP | Left parahippocampal | 0.116850427 | 0.204813354 | 0.570521525 | 0.573627313 | - |
| GFAP | Left paracentral | 0.161044366 | 0.20000864 | 0.805187046 | 0.42861715 | - |
| GFAP | Left pars opercularis | 0.171741825 | 0.197638741 | 0.868968423 | 0.393471721 | - |
| GFAP | Left pars orbitalis | 0.179283187 | 0.195319509 | 0.917896977 | 0.36780574 | - |
| GFAP | Left pars triangularis | 0.251621228 | 0.190779715 | 1.318909758 | 0.199646179 | - |
| GFAP | Left pericalcarine | 0.157805662 | 0.196684597 | 0.802328524 | 0.430236584 | - |
| GFAP | Left postcentral | 0.260207783 | 0.192535666 | 1.351478345 | 0.189144493 | - |
| GFAP | Left posterior cingulate | 0.19432127 | 0.197893719 | 0.981947641 | 0.335921555 | - |
| GFAP | Left precentral | 0.191435316 | 0.199424073 | 0.959940856 | 0.346656859 | - |
| GFAP | Left precuneus | 0.210576209 | 0.194731102 | 1.081369166 | 0.290279457 | - |
| GFAP | Left rostral anterior cingulate | 0.226693875 | 0.193061311 | 1.174206648 | 0.251832518 | - |
| GFAP | Left rostral middle frontal | 0.264307895 | 0.195647374 | 1.350940163 | 0.189314444 | - |
| GFAP | Left superior frontal | 0.166834887 | 0.200708625 | 0.831229285 | 0.414037859 | - |
| GFAP | Left superior parietal | 0.189436276 | 0.199883922 | 0.947731435 | 0.352712 | - |
| GFAP | Left superior temporal | 0.131470841 | 0.201394787 | 0.65280161 | 0.520088944 | - |
| GFAP | Left supramarginal | 0.209907175 | 0.198685949 | 1.056477196 | 0.301269342 | - |
| GFAP | Left frontal pole | 0.118501876 | 0.197018877 | 0.601474733 | 0.553163405 | - |
| GFAP | Left temporal pole | 0.066105077 | 0.20564609 | 0.321450688 | 0.750651692 | - |
| GFAP | Left transverse temporal | 0.14290552 | 0.202489188 | 0.705743952 | 0.487141876 | - |
| GFAP | Left insula | 0.18569989 | 0.198039802 | 0.937689737 | 0.357745114 | - |
| GFAP | Right bankssts | 0.107460483 | 0.204118246 | 0.526461917 | 0.603399296 | - |
| GFAP | Right caudal anterior cingulate | 0.127779137 | 0.201966586 | 0.632674641 | 0.532928291 | - |
| GFAP | Right caudal middle frontal | 0.250692128 | 0.200446259 | 1.250670021 | 0.223111082 | - |
| GFAP | Right cuneus | 0.233660736 | 0.188722934 | 1.238115218 | 0.227648401 | - |
| GFAP | Right entorhinal | 0.032900758 | 0.208396745 | 0.157875585 | 0.875875649 | - |
| GFAP | Right fusiform | 0.111809288 | 0.205726076 | 0.543486226 | 0.591808058 | - |
| GFAP | Right inferior parietal | 0.249407922 | 0.19123016 | 1.304229007 | 0.204525698 | - |
| GFAP | Right inferior temporal | 0.139814746 | 0.205251733 | 0.681186676 | 0.502273724 | - |
| GFAP | Right isthmus cingulate | 0.165258017 | 0.197520372 | 0.836663152 | 0.411035576 | - |
| GFAP | Right lateral occipital | 0.158709997 | 0.199757012 | 0.794515273 | 0.434682245 | - |
| GFAP | Right lateral orbitofrontal | 0.166126375 | 0.200968734 | 0.826627962 | 0.416590911 | - |
| GFAP | Right lingual | 0.108335055 | 0.205873306 | 0.526221963 | 0.603563446 | - |
| GFAP | Right medial orbitofrontal | 0.212339069 | 0.193192666 | 1.099105225 | 0.282625912 | - |
| GFAP | Right middle temporal | 0.216134136 | 0.19663753 | 1.099149975 | 0.282606787 | - |
| GFAP | Right parahippocampal | 0.102497558 | 0.208744578 | 0.491019019 | 0.627873036 | - |
| GFAP | Right paracentral | 0.289184226 | 0.191605178 | 1.509271454 | 0.144283116 | - |
| GFAP | Right pars opercularis | 0.190774124 | 0.198552085 | 0.960826594 | 0.34622034 | - |
| GFAP | Right pars orbitalis | 0.175638713 | 0.199001061 | 0.882601892 | 0.386206541 | - |
| GFAP | Right pars triangularis | 0.19295182 | 0.19093229 | 1.010577208 | 0.322299452 | - |
| GFAP | Right pericalcarine | 0.179125306 | 0.18557706 | 0.965234099 | 0.34405372 | - |
| GFAP | Right postcentral | 0.22234127 | 0.19892058 | 1.1177389 | 0.274742864 | - |
| GFAP | Right posterior cingulate | 0.087594011 | 0.200833609 | 0.436152155 | 0.666624603 | - |
| GFAP | Right precentral | 0.170028475 | 0.202002861 | 0.841713202 | 0.408257716 | - |
| GFAP | Right precuneus | 0.188597292 | 0.198341153 | 0.950873227 | 0.351147098 | - |
| GFAP | Right rostral anterior cingulate | 0.155707232 | 0.195762031 | 0.79539036 | 0.434182931 | - |
| GFAP | Right rostral middle frontal | 0.224450413 | 0.199630782 | 1.124327675 | 0.271994008 | - |
| GFAP | Right superior frontal | 0.185742566 | 0.200123678 | 0.928138879 | 0.362576604 | - |
| GFAP | Right superior parietal | 0.230688797 | 0.19688247 | 1.171708164 | 0.252815355 | - |
| GFAP | Right superior temporal | 0.116286558 | 0.203436732 | 0.571610433 | 0.572900973 | - |
| GFAP | Right supramarginal | 0.227161037 | 0.198693427 | 1.143274041 | 0.264201253 | - |
| GFAP | Right frontal pole | 0.217438851 | 0.197446492 | 1.101254563 | 0.281708389 | - |
| GFAP | Right temporal pole | 0.059826547 | 0.209499544 | 0.285568867 | 0.777658513 | - |
| GFAP | Right transverse temporal | 0.051218872 | 0.203221351 | 0.252034896 | 0.803159911 | - |
| GFAP | Right insula | 0.160073119 | 0.202329062 | 0.791152381 | 0.436604352 | - |

Abbreviations: FDR, false discovery rate; GFAP, glial fibrillar acidic protein; MSA, multiple system atrophy; NfL, neurofilament light; PET, positron emission tomography.

Note: Associations of plasma measures with PET indices were analyzed using multiple linear regressions after adjusting for age and sex.

**Supplementary Table 12: Demographic characteristics and plasma patterns per diagnostic group in an independent advanced disease cohort**

| Characteristic | HC | MSA | MSA-C | MSA-P | SCA | PD | P value |
| --- | --- | --- | --- | --- | --- | --- | --- |
|  | N = 100 | N = 3 | N = 2 | N = 1 | N = 31 | N = 11 |  |
| Age, years | 59.50 (8.91) | 60.67 (5.86) | 64.00 (1.41) | 54.00 (NA) | 46.10 (13.17) | 63.09 (12.38) | <0.001 |
| Male, n (%) | 44 (44.00%) | 1 (33.33%) | 1 (50.00%) | 0 (0.00%) | 14 (45.16%) | 5 (45.45%) | >0.999 |
| Disease duration, months | – | 52.00 (6.93) | 48.00 (0.00) | 60.00 (NA) | 86.35 (46.51) | 69.91 (20.14) | 0.147 |
| Education, years | 9.69 (4.22) | 3.00 (0.00) | 3.00 (NA) | 3.00 (NA) | 9.47 (3.84) | 8.27 (4.50) | 0.147 |
| Plasma NfL, pg/mL | 13.74 (8.53) | 33.87 (7.25) | 33.23 (10.13) | 35.14 (NA) | 32.76 (16.42) | 26.23 (20.12) | <0.001 |
| Plasma GFAP, pg/mL | 69.20 (35.69) | 90.74 (17.30) | 96.94 (19.19) | 78.36 (NA) | 71.32 (39.13) | 113.36 (65.46) | 0.005 |
| Plasma p-tau181, pg/mL | 1.99 (0.84) | 2.05 (0.89) | 2.43 (0.84) | 1.29 (NA) | 1.48 (0.46) | 1.99 (1.33) | 0.442 |
| Plasma Aβ40, pg/mL | 83.02 (25.78) | 53.67 (19.48) | 53.91 (27.54) | 53.18 (NA) | 67.99 (26.07) | 92.32 (27.04) | 0.331 |
| Plasma Aβ42, pg/mL | 4.96 (1.63) | 4.54 (2.49) | 4.84 (3.44) | 3.95 (NA) | 4.74 (1.38) | 5.62 (1.58) | 0.666 |
| Plasma Aβ42/40 | 0.06 (0.02) | 0.08 (0.02) | 0.08 (0.02) | 0.07 (NA) | 0.07 (0.01) | 0.06 (0.01) | 0.196 |

Continuous data are described as mean (standard deviations), and categorical variables are presented as numbers (percentages).

Abbreviations: Αβ, amyloid-β; GFAP, glial fibrillary acidic protein; HC, healthy control; MSA, multiple system atrophy; MSA-C, multiple system atrophy-cerebellar type; MSA-P, multiple system atrophy-parkinsonian type; NfL, neurofilament light; PD, Parkinson's disease; p-tau181, phosphorylated tau at threonine 181; SCA, spinocerebellar ataxia.

**Supplementary Figure 3: Distributions of the plasma levels across diagnostic groups in an independent advanced disease cohort**


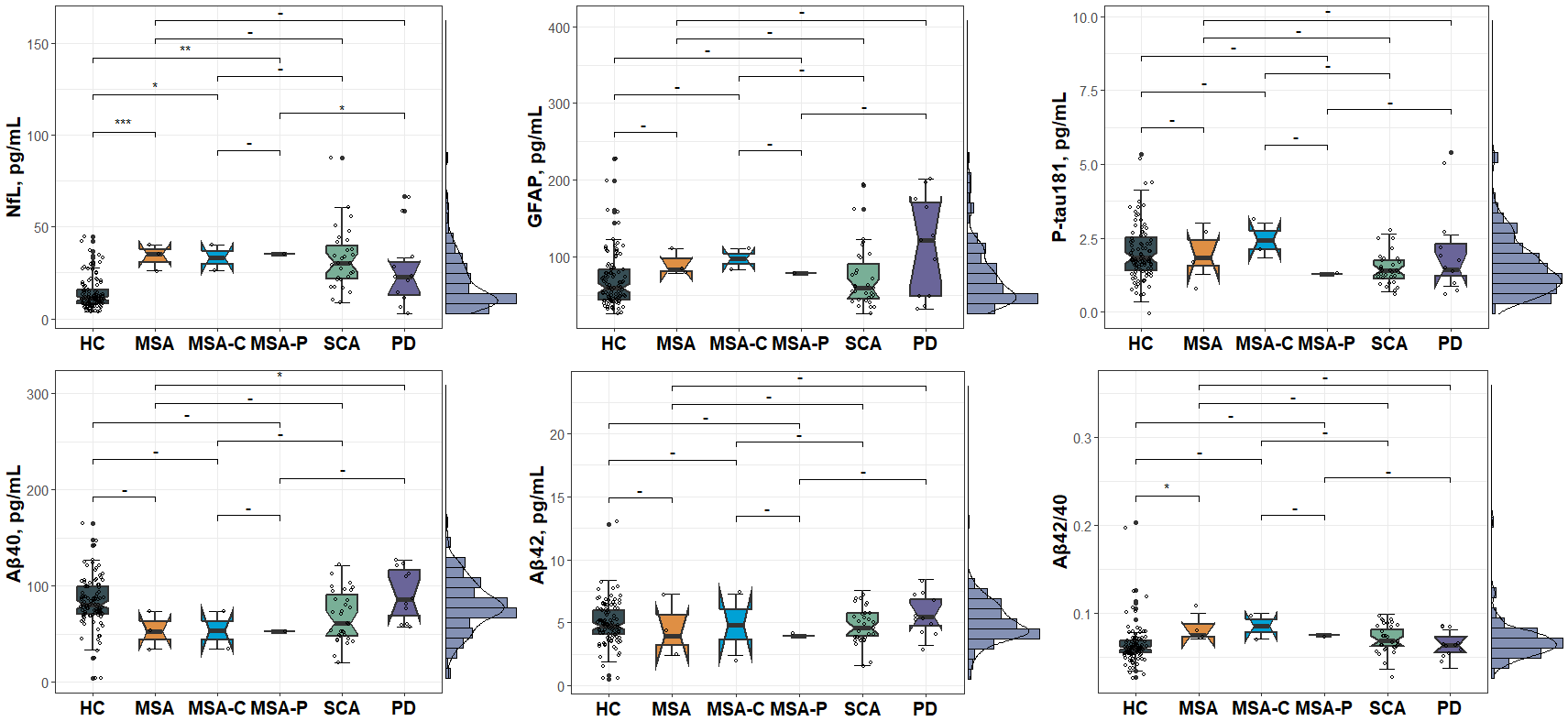


Plasma levels of NfL, GFAP, p-tau181, Αβ40, Aβ42, and Αβ42/40 per diagnostic group were compared using analysis of covariance after controlling for age and sex. Significance: ****p<0.0001, ***p<0.001, **p<0.01, *p<0.05, -: p≥0.05.

Abbreviations: Αβ, amyloid-β; GFAP, glial fibrillary acidic protein; HC, healthy control; MSA, multiple system atrophy; MSA-C, multiple system atrophy-cerebellar type; MSA-P, multiple system atrophy-parkinsonian type; NfL, neurofilament light; ns, non-significant; PD, Parkinson's disease; p-tau181, phosphorylated tau at threonine 181; SCA, spinocerebellar ataxia.

**Supplementary Table 13: Results of receiver operating characteristic (ROC) analyses in an independent advanced disease cohort**

| MSA versus HC | NFL | GFAP | P-tau 181 | Aβ40 | Aβ42 | Aβ42/40 |
| --- | --- | --- | --- | --- | --- | --- |
| AUC (95% CI) | 0.943 (0.872, 1.015) | 0.783 (0.653, 0.914) | 0.526 (0.122, 0.929) | 0.847 (0.664, 1.029) | 0.603 (0.052, 1.154) | 0.873 (0.768, 0.979) |
| Cutoff | 26.062 | 78.360 | 3.031 | 74.636 | 3.958 | 0.070 |
| Sensitivity | 1.000 | 1.000 | 0.333 | 0.670 | 0.800 | 1.000 |
| Specificity | 0.880 | 0.700 | 0.897 | 1.000 | 0.667 | 0.790 |
| False positive rate | 0.120 | 0.300 | 0.103 | 0.000 | 0.333 | 0.210 |
| False negative rate | 0.000 | 0.000 | 0.667 | 0.330 | 0.200 | 0.000 |
| Positive predictive value | 0.200 | 0.091 | 0.091 | 1.000 | 0.988 | 0.125 |
| Negative predictive value | 1.000 | 1.000 | 0.978 | 0.083 | 0.091 | 1.000 |
|  |  |  |  |  |  |  |
|  |  |  |  |  |  |  |
| MSA versus SCA | NFL | GFAP | P-tau 181 | Aβ40 | Aβ42 | Aβ42/40 |
| AUC (95% CI) | 0.591 (0.339, 0.844) | 0.763 (0.605, 0.922) | 0.763 (0.409, 1.118) | 0.634 (0.298, 0.971) | 0.556 (0.021, 1.09) | 0.711 (0.404, 1.019) |
| Cutoff | 26.062 | 78.360 | 1.836 | 77.841 | 4.007 | 0.070 |
| Sensitivity | 1.000 | 1.000 | 0.667 | 0.387 | 0.700 | 1.000 |
| Specificity | 0.387 | 0.710 | 0.871 | 1.000 | 0.667 | 0.567 |
| False positive rate | 0.613 | 0.290 | 0.129 | 0.000 | 0.333 | 0.433 |
| False negative rate | 0.000 | 0.000 | 0.333 | 0.613 | 0.300 | 0.000 |
| Positive predictive value | 0.136 | 0.250 | 0.333 | 1.000 | 0.955 | 0.188 |
| Negative predictive value | 1.000 | 1.000 | 0.964 | 0.136 | 0.182 | 1.000 |
|  |  |  |  |  |  |  |
|  |  |  |  |  |  |  |
|  |  |  |  |  |  |  |
| MSA versus PD | NFL | GFAP | P-tau 181 | Aβ40 | Aβ42 | Aβ42/40 |
| AUC (95% CI) | 0.758 (0.495, 1.021) | 0.606 (0.31, 0.902) | 0.6 (0.199, 1.001) | 0.909 (0.709, 1.11) | 0.636 (0.082, 1.191) | 0.788 (0.507, 1.069) |
| Cutoff | 26.062 | 121.200 | 1.293 | 76.910 | 4.520 | 0.070 |
| Sensitivity | 1.000 | 0.545 | 1.000 | 0.727 | 0.818 | 1.000 |
| Specificity | 0.636 | 1.000 | 0.300 | 1.000 | 0.667 | 0.636 |
| False positive rate | 0.364 | 0.000 | 0.700 | 0.000 | 0.333 | 0.364 |
| False negative rate | 0.000 | 0.455 | 0.000 | 0.273 | 0.182 | 0.000 |
| Positive predictive value | 0.429 | 1.000 | 0.300 | 1.000 | 0.900 | 0.429 |
| Negative predictive value | 1.000 | 0.375 | 1.000 | 0.500 | 0.500 | 1.000 |

Note: Considering the small sample size of MSA-C and MSA-P groups, we performed ROC analyses only in MSA versus HC, MSA versus SCA, and MSA versus PD groups.

Abbreviations: Αβ, amyloid-β; AUC, area under the curve; CI, confidence interval; GFAP, glial fibrillary acidic protein; HC, healthy control; MSA, multiple system atrophy; NfL, neurofilament light; PD, Parkinson's disease; p-tau181, phosphorylated tau at threonine 181; SCA, spinocerebellar ataxia.

**Supplementary Table 14: Receiver operating characteristic (ROC) analyses of neuroimaging indices**

| MSA-C versus SCA | Pons volume | Left cerebellum volume | Right cerebellum volume |
| --- | --- | --- | --- |
| AUC (95% CI) | 0.614 (0.405, 0.823) | 0.644 (0.42, 0.869) | 0.612 (0.379, 0.845) |
| Cutoff | 11528.510 | 30477.700 | 30691.800 |
| Sensitivity | 0.500 | 0.600 | 0.500 |
| Specificity | 0.814 | 0.837 | 0.860 |
| False positive rate | 0.186 | 0.163 | 0.140 |
| False negative rate | 0.500 | 0.400 | 0.500 |
| Positive predictive value | 0.385 | 0.462 | 0.455 |
| Negative predictive value | 0.875 | 0.900 | 0.881 |

| MSA-P versus PD | Left putamen volume | Right putamen volume |
| --- | --- | --- |
| AUC (95% CI) | 0.619 (0.307, 0.931) | 0.746 (0.449, 1.043) |
| Cutoff | 4377.200 | 4715.600 |
| Sensitivity | 0.857 | 0.857 |
| Specificity | 0.556 | 0.778 |
| False positive rate | 0.444 | 0.222 |
| False negative rate | 0.143 | 0.143 |
| Positive predictive value | 0.600 | 0.750 |
| Negative predictive value | 0.833 | 0.875 |

Note: For patients with available data of brain volumes, we performed ROC analyses in MSA-C versus SCA and MSA-P versus PD groups.

Abbreviations: AUC, area under the curve; CI, confidence interval; MSA-C, multiple system atrophy-cerebellar type; MSA-P, multiple system atrophy-parkinsonian type; PD, Parkinson's disease; SCA, spinocerebellar ataxia.

**Supplementary Table 15: Results of post-hoc power analysis**

| Plasma NfL | Number of groups | Average sample number of the two groups | Effect size | Significance level | Calculated power |
| --- | --- | --- | --- | --- | --- |
| MSA versus HC | 2 | 86.5 | 244.312 | 0.00625 (0.05/8) | 1 |
| MSA-C versus HC | 2 | 79 | 219.7682 | 0.00625 (0.05/8) | 1 |
| MSA-P versus HC | 2 | 57.5 | 58.14301 | 0.00625 (0.05/8) | 1 |
| MSA-C versus MSA-P | 2 | 36.5 | 2.616284 | 0.00625 (0.05/8) | 1 |
| MSA versus PD | 2 | 50.5 | 19.89417 | 0.00625 (0.05/8) | 1 |
| MSA versus SCA | 2 | 51 | 1.694515 | 0.00625 (0.05/8) | 1 |
| MSA-P versus PD | 2 | 21.5 | 4.267351 | 0.00625 (0.05/8) | 1 |
| MSA-C versus SCA | 2 | 43.5 | 2.354245 | 0.00625 (0.05/8) | 1 |
|  |  |  |  |  |  |
|  |  |  |  |  |  |
| Plasma GFAP | Number of groups | Average sample number of the two groups | Effect size | Significance level | Calculated power |
| MSA versus HC | 2 | 86.5 | 16.79616 | 0.00625 (0.05/8) | 1 |
| MSA-C versus HC | 2 | 79 | 19.77655 | 0.00625 (0.05/8) | 1 |
| MSA-P versus HC | 2 | 57.5 | 0.5207354 | 0.00625 (0.05/8) | 0.9970794 |
| MSA-C versus MSA-P | 2 | 36.5 | 4.377141 | 0.00625 (0.05/8) | 1 |
| MSA versus PD | 2 | 50.5 | 3.458569 | 0.00625 (0.05/8) | 1 |
| MSA versus SCA | 2 | 51 | 4.380657 | 0.00625 (0.05/8) | 1 |
| MSA-P versus PD | 2 | 21.5 | 6.632106 | 0.00625 (0.05/8) | 1 |
| MSA-C versus SCA | 2 | 43.5 | 5.54801 | 0.00625 (0.05/8) | 1 |
|  |  |  |  |  |  |
|  |  |  |  |  |  |
|  |  |  |  |  |  |
| Plasma p-tau181 | Number of groups | Average sample number of the two groups | Effect size | Significance level | Calculated power |
| MSA versus HC | 2 | 86.5 | 26.91087 | 0.00625 (0.05/8) | 1 |
| MSA-C versus HC | 2 | 79 | 28.13753 | 0.00625 (0.05/8) | 1 |
| MSA-P versus HC | 2 | 57.5 | 3.879778 | 0.00625 (0.05/8) | 1 |
| MSA-C versus MSA-P | 2 | 36.5 | 1.53779 | 0.00625 (0.05/8) | 1 |
| MSA versus PD | 2 | 50.5 | 2.889239 | 0.00625 (0.05/8) | 1 |
| MSA versus SCA | 2 | 51 | 0.5847802 | 0.00625 (0.05/8) | 0.9988925 |
| MSA-P versus PD | 2 | 21.5 | 0.01411311 | 0.00625 (0.05/8) | 0.006453612 |
| MSA-C versus SCA | 2 | 43.5 | 1.123114 | 0.00625 (0.05/8) | 1 |
|  |  |  |  |  |  |
|  |  |  |  |  |  |
| Plasma Aβ40 | Number of groups | Average sample number of the two groups | Effect size | Significance level | Calculated power |
| MSA versus HC | 2 | 86.5 | 10.05009 | 0.00625 (0.05/8) | 1 |
| MSA-C versus HC | 2 | 79 | 6.520897 | 0.00625 (0.05/8) | 1 |
| MSA-P versus HC | 2 | 57.5 | 5.129857 | 0.00625 (0.05/8) | 1 |
| MSA-C versus MSA-P | 2 | 36.5 | 1.458015 | 0.00625 (0.05/8) | 1 |
| MSA versus PD | 2 | 50.5 | 14.10916 | 0.00625 (0.05/8) | 1 |
| MSA versus SCA | 2 | 51 | 1.747348 | 0.00625 (0.05/8) | 1 |
| MSA-P versus PD | 2 | 21.5 | 13.37081 | 0.00625 (0.05/8) | 1 |
| MSA-C versus SCA | 2 | 43.5 | 1.236379 | 0.00625 (0.05/8) | 1 |
|  |  |  |  |  |  |
|  |  |  |  |  |  |
|  |  |  |  |  |  |
| Plasma Aβ42 | Number of groups | Average sample number of the two groups | Effect size | Significance level | Calculated power |
| MSA versus HC | 2 | 86.5 | 3.103842 | 0.00625 (0.05/8) | 1 |
| MSA-C versus HC | 2 | 79 | 1.268133 | 0.00625 (0.05/8) | 1 |
| MSA-P versus HC | 2 | 57.5 | 3.817272 | 0.00625 (0.05/8) | 1 |
| MSA-C versus MSA-P | 2 | 36.5 | 3.014134 | 0.00625 (0.05/8) | 1 |
| MSA versus PD | 2 | 50.5 | 13.96248 | 0.00625 (0.05/8) | 1 |
| MSA versus SCA | 2 | 51 | 0.6140151 | 0.00625 (0.05/8) | 0.9995949 |
| MSA-P versus PD | 2 | 21.5 | 11.68478 | 0.00625 (0.05/8) | 1 |
| MSA-C versus SCA | 2 | 43.5 | 0.2287045 | 0.00625 (0.05/8) | 0.2585369 |
|  |  |  |  |  |  |
|  |  |  |  |  |  |
| Plasma Aβ42/40 | Number of groups | Average sample number of the two groups | Effect size | Significance level | Calculated power |
| MSA versus HC | 2 | 86.5 | 10.74686 | 0.00625 (0.05/8) | 1 |
| MSA-C versus HC | 2 | 79 | 10.66375 | 0.00625 (0.05/8) | 1 |
| MSA-P versus HC | 2 | 57.5 | 1.571625 | 0.00625 (0.05/8) | 1 |
| MSA-C versus MSA-P | 2 | 36.5 | 0.4590013 | 0.00625 (0.05/8) | 0.8607052 |
| MSA versus PD | 2 | 50.5 | 0.09885589 | 0.00625 (0.05/8) | 0.03935244 |
| MSA versus SCA | 2 | 51 | 0.918615 | 0.00625 (0.05/8) | 1 |
| MSA-P versus PD | 2 | 21.5 | 0.000376274 | 0.00625 (0.05/8) | 0.006250144 |
| MSA-C versus SCA | 2 | 43.5 | 1.517365 | 0.00625 (0.05/8) | 1 |

Note: Power analyses were performed using the “pwr.anova.test” function in R software.
